# Supplementary material for: Capivasertib plus fulvestrant in patients with HR-positive/HER2-negative advanced breast cancer: phase 3 CAPItello-291 study extended Chinese cohort
Source: Nat Commun. 2025 May 9;16:4324. doi: 10.1038/s41467-025-59210-6 (PMC12064754; doi:10.1038/s41467-025-59210-6)
Supplement: Supplementary file 1 — Supplementary information [file 41467_2025_59210_MOESM1_ESM.pdf]

# Supplementary Information: Capivasertib plus fulvestrant in patients with HR-positive/HER2-negative advanced breast cancer: phase 3 CAPItello-291 study extended Chinese cohort

**Supplementary Table 1 | *PIK3CA/AKT1/PTEN* tumor alteration status by NGS in the Chinese cohort**

| **Alteration, *n* (%)** | | **Capivasertib–fulvestrant (*n* = 71)** | **Placebo–fulvestrant (*n* = 63)** |
|---|---|---|---|
| **Any *PIK3CA/AKT1/PTEN* alteration** | | 24 (33.8) | 22 (34.9) |
| *PIK3CA* | *PIK3CA* only | 17 (23.9) | 15 (23.8) |
| | *PIK3CA* and *PTEN* | 1 (1.4) | 2 (3.2) |
| *AKT1* only | | 3 (4.2) | 3 (4.8) |
| *PTEN* only | | 3 (4.2) | 2 (3.2) |
| ***PIK3CA/AKT1/PTEN* non-altered** | | 47 (66.2) | 41 (65.1) |
| *PIK3CA/AKT1/PTEN* alteration not detected | | 35 (49.3) | 33 (52.4) |
| Unknown | | 12 (16.9) | 8 (12.7) |
| No sample available | | 0 | 1 (1.6) |
| Preanalytical failure | | 6 (8.5) | 3 (4.8) |
| Postanalytical failure | | 6 (8.5) | 4 (6.3) |

*AKT1*, Akt serine/threonine kinase 1; NGS, next-generation sequencing; *PIK3CA*, catalytic subunit alpha of phosphatidylinositol-3-kinase; *PTEN*, phosphatase and tensin homolog.

**Supplementary Table 2 | Summary of tumor response by investigator assessment and blinded independent central review in the Chinese cohort**

| | Overall population | | Patients with *PIK3CA/AKT1/PTEN*-altered tumors | |
|---|---|---|---|---|
| | **Capivasertib–fulvestrant (*n* = 71)** | **Placebo–fulvestrant (*n* = 63)** | **Capivasertib–fulvestrant (*n* = 24)** | **Placebo–fulvestrant (*n* = 22)** |
| **Investigator assessment** | | | | |
| Patients with measurable disease at baseline, *n* | 68 | 60 | 23 | 21 |
| Objective response rate, *n* (%) | 20 (29.4) | 5 (8.3) | 9 (39.1) | 2 (9.5) |
| Odds ratio (95% CI)[a] | 4.58 (1.60–13.15) | | NC (NC) | |
| Median duration of response, months | 5.6 | 2.1 | 4.4 | 1.2 |
| Best overall response in all patients, *n* (%) | 20 (28.2) | 5 (7.9) | 9 (37.5) | 2 (9.1) |
| Complete response<br>Partial response<br>Stable disease (≥ 8 weeks)<br>Progressive disease<br>Non-evaluable | 1 (1.4)<br>19 (26.8)<br>36 (50.7)<br>13 (18.3)<br>2 (2.8) | 0<br>5 (7.9)<br>25 (39.7)<br>30 (47.6)<br>3 (4.8) | 0<br>9 (37.5)<br>11 (45.8)<br>4 (16.7)<br>0 | 0<br>2 (9.1)<br>7 (31.8)<br>12 (54.5)<br>1 (4.5) |
| Clinical benefit rate,[b] *n* (%) | 41 (57.7) | 18 (28.6) | 14 (58.3) | 5 (22.7) |
| **Blinded independent central review** | | | | |
| Best overall response in all patients, *n* (%) | 20 (28.2) | 3 (4.8) | 8 (33.3) | 0 |
| Complete response<br>Partial response<br>Stable disease (≥ 8 weeks)<br>Progressive disease<br>Non-evaluable | 1 (1.4)<br>19 (26.8)<br>34 (47.9)<br>15 (21.1)<br>2 (2.8) | 0<br>3 (4.8)<br>27 (42.9)<br>29 (46.0)<br>4 (6.3) | 0<br>8 (33.3)<br>11 (45.8)<br>5 (20.8)<br>0 | 0<br>0<br>7 (31.8)<br>14 (63.6)<br>1 (4.5) |

[a]Analysis was performed using unadjusted logistic regression in patients with measurable disease at baseline. NC for patients with *PIK3CA/AKT1/PTEN*-altered tumors due to insufficient number of responders (<20 across both treatment groups). [b]Clinical benefit rate at 24 weeks—a secondary objective—was defined as the percentage of patients who have a complete or partial response or

stable disease per RECIST v1.1 (without subsequent cancer therapy) for ≥ 23 weeks after treatment initiation. *AKT1*, Akt serine/threonine kinase 1; CI, confidence interval; NC, non-calculable; *PIK3CA*, catalytic subunit alpha of phosphatidylinositol-3-kinase; *PTEN*, phosphatase and tensin homolog; RECIST, Response Evaluation Criteria in Solid Tumors.

**Supplementary Table 3 | Subsequent anticancer therapies**

| Anticancer therapy, n (%) | Overall population | | Patients with *PIK3CA/AKT1/PTEN*-altered tumors | |
|---|---|---|---|---|
| | **Capivasertib–fulvestrant (*n* = 71)** | **Placebo–fulvestrant (*n* = 63)** | **Capivasertib–fulvestrant (*n* = 24)** | **Placebo–fulvestrant (*n* = 22)** |
| Total number of patients | 40 (56.3) | 45 (71.4) | 14 (58.3) | 18 (81.8) |
| Cytotoxic chemotherapy | 29 (40.8) | 36 (57.1) | 12 (50.0) | 14 (63.6) |
| Targeted therapy | 16 (22.5) | 16 (25.4) | 7 (29.2) | 7 (31.8) |
| Hormonal therapy | 15 (21.1) | 14 (22.2) | 5 (20.8) | 6 (27.3) |
| Antiangiogenic therapy | 7 (9.9) | 1 (1.6) | 3 (12.5) | 0 |
| Biologic therapy | 1 (1.4) | 0 | 0 | 0 |
| PARP inhibitor | 1 (1.4) | 0 | 0 | 0 |
| Immunotherapy | 0 | 1 (1.6) | 0 | 1 (4.5) |
| Other | 3 (4.2) | 3 (4.8) | 0 | 2 (9.1) |

Patients may have received ≥ 1 subsequent anticancer therapy.

PARP, poly(ADP-ribose) polymerase.

**Supplementary Table 4 | SAEs in the Chinese cohort overall population (safety population)[a]**

| SAE, n (%) | Capivasertib–fulvestrant (*n* = 71) | Placebo–fulvestrant (*n* = 62) |
|---|---|---|
| Patients with any SAE | 20 (28.2) | 3 (4.8) |
| Rash[b] | 3 (4.2) | 0 |
| Erythema multiform | 2 (2.8) | 0 |
| Vomiting | 2 (2.8) | 0 |
| Blood creatinine increased | 1 (1.4) | 0 |
| Bone tuberculosis | 1 (1.4) | 0 |
| COVID-19 | 1 (1.4) | 0 |
| Dermatitis | 1 (1.4) | 0 |
| Drug reaction with eosinophilia and systemic symptoms | 1 (1.4) | 0 |
| Endometrial cancer | 1 (1.4) | 0 |
| Granulocytopenia | 1 (1.4) | 0 |
| Hepatitis B reactivation | 1 (1.4) | 0 |
| Hemorrhoids | 1 (1.4) | 0 |
| Hordeolum | 1 (1.4) | 0 |
| Hydronephrosis | 1 (1.4) | 0 |
| Hypercalcemia | 1 (1.4) | 0 |
| Hyperglycemia | 1 (1.4) | 0 |
| Hypokalemia | 1 (1.4) | 0 |
| Nausea | 1 (1.4) | 0 |
| Platelet count decreased | 1 (1.4) | 0 |
| Pneumonia | 1 (1.4) | 0 |
| Pyrexia | 1 (1.4) | 0 |
| Renal failure | 1 (1.4) | 0 |
| Supraventricular extrasystoles | 1 (1.4) | 0 |
| Uterine leiomyoma | 1 (1.4) | 0 |
| Vertigo | 1 (1.4) | 0 |
| Anemia | 0 | 1 (1.6) |
| Drug-induced liver injury | 0 | 1 (1.6) |
| Hepatic function abnormal | 0 | 1 (1.6) |
| Pericardial effusion | 0 | 1 (1.6) |

[a]The safety population included all the patients who received at least one dose of capivasertib, fulvestrant or placebo. All reported SAEs are listed. AEs are reported regardless of the relationship to the study drugs. [b]Group term of rash includes the preferred terms of rash, rash macular, rash maculopapular, rash papular and rash pruritic. All three SAEs reported were for rash maculopapular. AE, adverse event; COVID-19, coronavirus disease 2019; SAE, serious adverse event.

**Supplementary Table 5 | Summary of AEs leading to treatment discontinuation, dose reduction or dose interruption in the Chinese cohort overall population (safety population)**

| n (%) | Capivasertib–fulvestrant (*n* = 71) | Placebo–fulvestrant (*n* = 62) |
|---|---|---|
| **Any AE leading to discontinuation** | 8 (11.3) | 2 (3.2) |
| Discontinuation of capivasertib/placebo only | 6 (8.5) | 0 |
| Discontinuation of both capivasertib/placebo and fulvestrant | 2 (2.8) | 2 (3.2) |
| **Any AE leading to dose reduction** | 13 (18.3) | 0 |
| Dose reduction of capivasertib/placebo only | 13 (18.3) | 0 |
| **Any AE leading to dose interruption** | 37 (52.1) | 18 (29.0) |
| Dose interruption of capivasertib/placebo only | 29 (40.8) | 10 (16.1) |
| Dose interruption of fulvestrant only | 1 (1.4) | 0 |
| Dose interruption of both capivasertib/placebo and fulvestrant | 7 (9.9) | 8 (12.9) |

AE, adverse event.

**Supplementary Fig. 1 | Study overview (CONSORT diagram)**

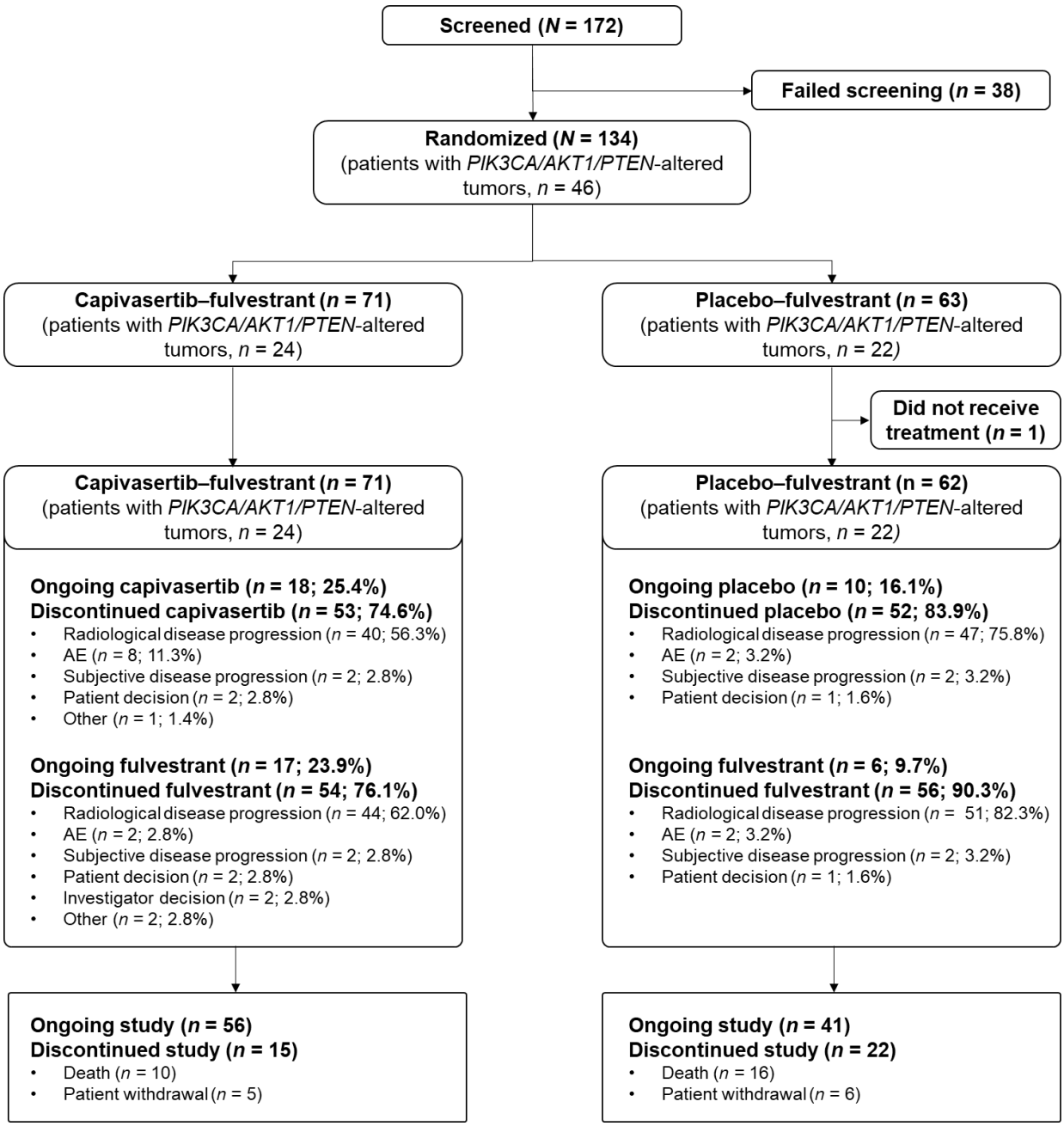


Deaths were obtained from public records or survival follow-up. AE, adverse event; *AKT1*, Akt serine/threonine kinase 1; *PIK3CA*, catalytic subunit alpha of phosphatidylinositol-3-kinase; *PTEN*, phosphatase and tensin homolog.

**Supplementary Fig. 2 | Investigator-assessed PFS in the Chinese cohort patients with *PIK3CA/AKT1/PTEN*-non-altered tumors a, including unknown NGS result, b, excluding unknown NGS result, and c, only with unknown NGS result (*post-hoc*)**

**a**

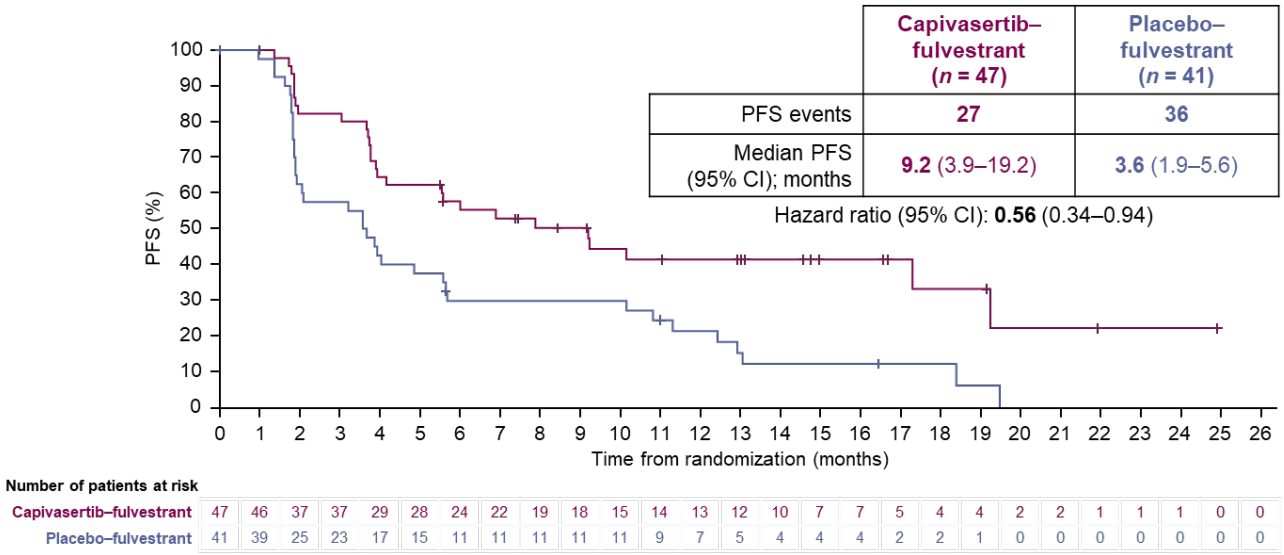


**b**

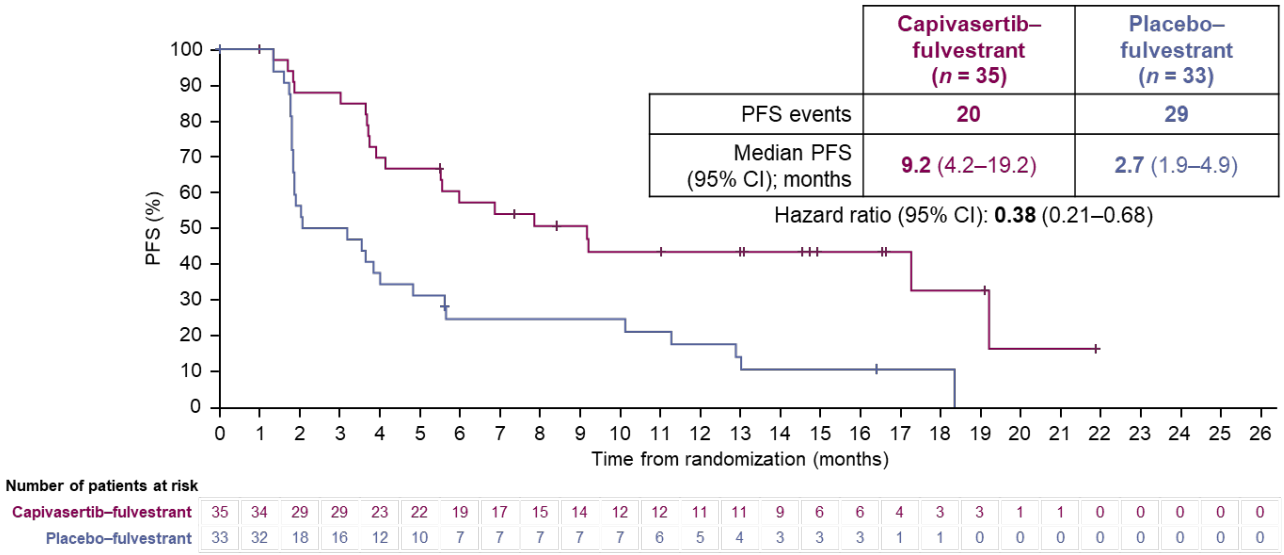

**c**

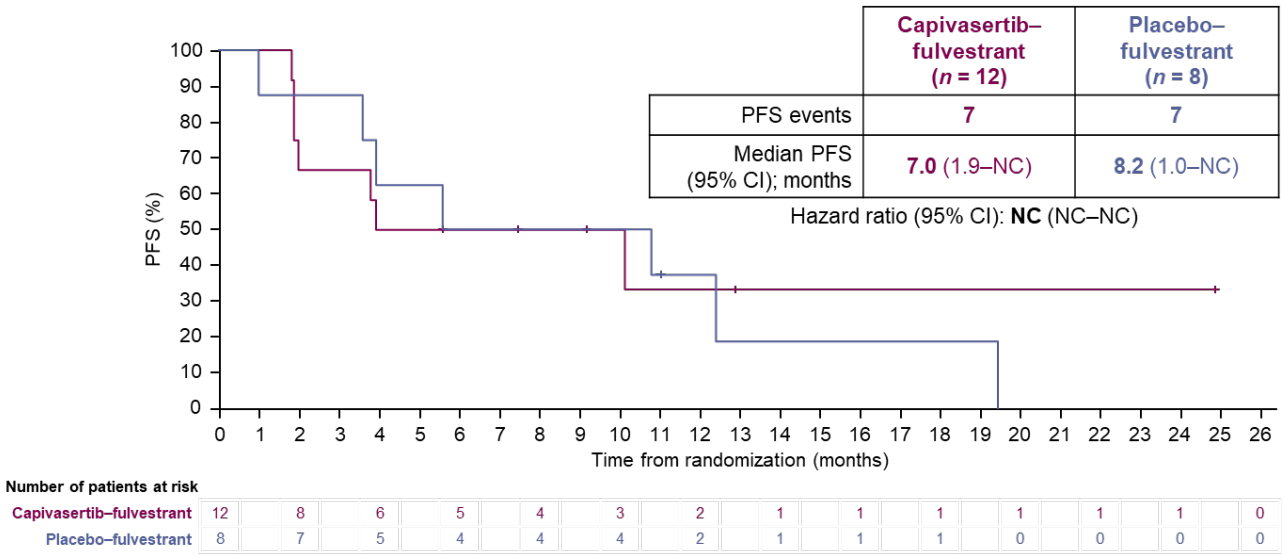


The hazard ratio of the capivasertib–fulvestrant (purple) and placebo–fulvestrant (blue) curves was estimated with the use of an unstratified Cox proportional hazards model. Tick marks indicate censored data. Results for the population of patients with tumors with unknown NGS results are not presented, due to an insufficient number of events (<20 across treatment groups). *AKT1*, Akt serine/threonine kinase 1; CI, confidence interval; NC, not calculated; NGS, next-generation sequencing; PFS, progression-free survival; *PIK3CA*, catalytic subunit alpha of phosphatidylinositol-3-kinase; *PTEN*, phosphatase and tensin homolog.

**Supplementary Fig. 3 | Subgroup analysis of investigator-assessed PFS in the Chinese cohort patients with *PIK3CA/AKT1/PTEN*-altered tumors**

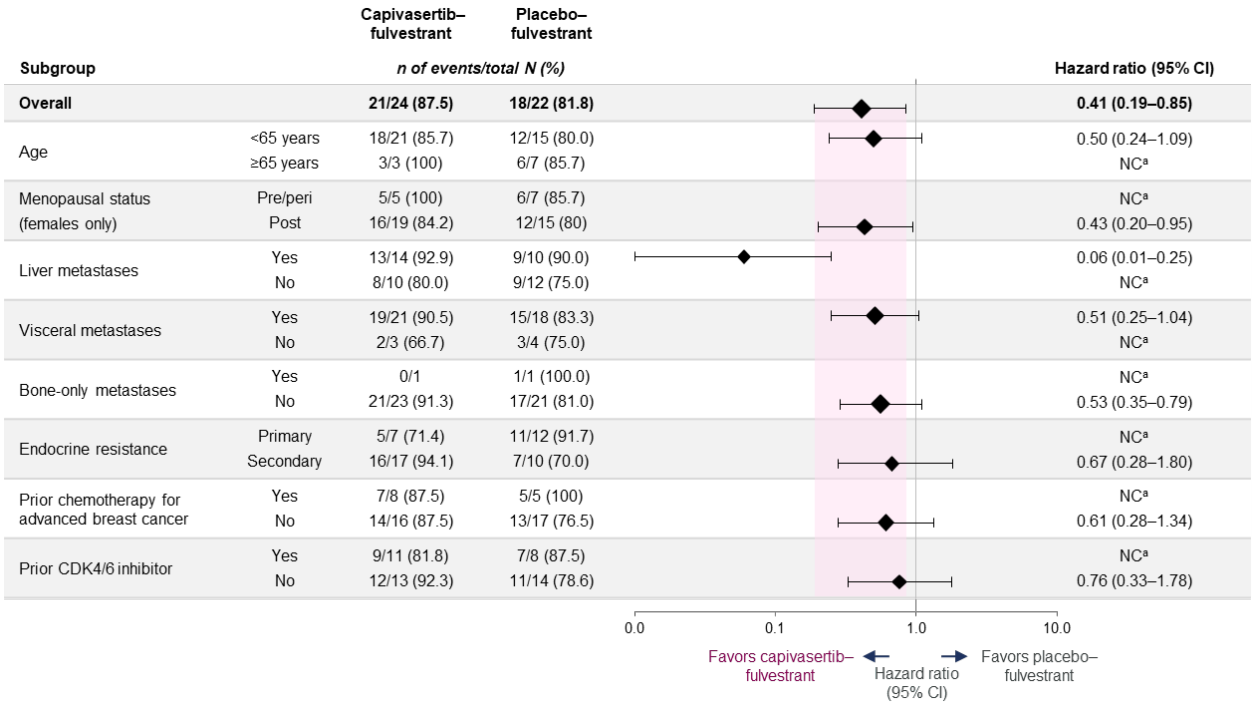


Subgroup analysis within the population of patients with *PIK3CA/AKT1/PTEN*-altered tumors was performed at each subgroup level with the use of a Cox proportional hazards model, including the trial-group term only. Data are presented as unstratified HRs and 95% CI. Selected subgroups of interest are shown. Menopausal status was assessed in women only. [a]Hazard ratio and 95% CI were not calculated, due to an insufficient number of events (<20 across treatment groups). *AKT1*, Akt serine/threonine kinase 1; CDK4/6, cyclin-dependent kinase 4/6; CI, confidence interval; NC, not calculated; PFS, progression-free survival; *PIK3CA*, catalytic subunit alpha of phosphatidylinositol-3-kinase; *PTEN*, phosphatase and tensin homolog.

**Supplementary Fig. 4 | PFS by blinded independent central review in a, the Chinese cohort overall population and b, patients with *PIK3CA/AKT1/PTEN*-altered tumors**

**a**

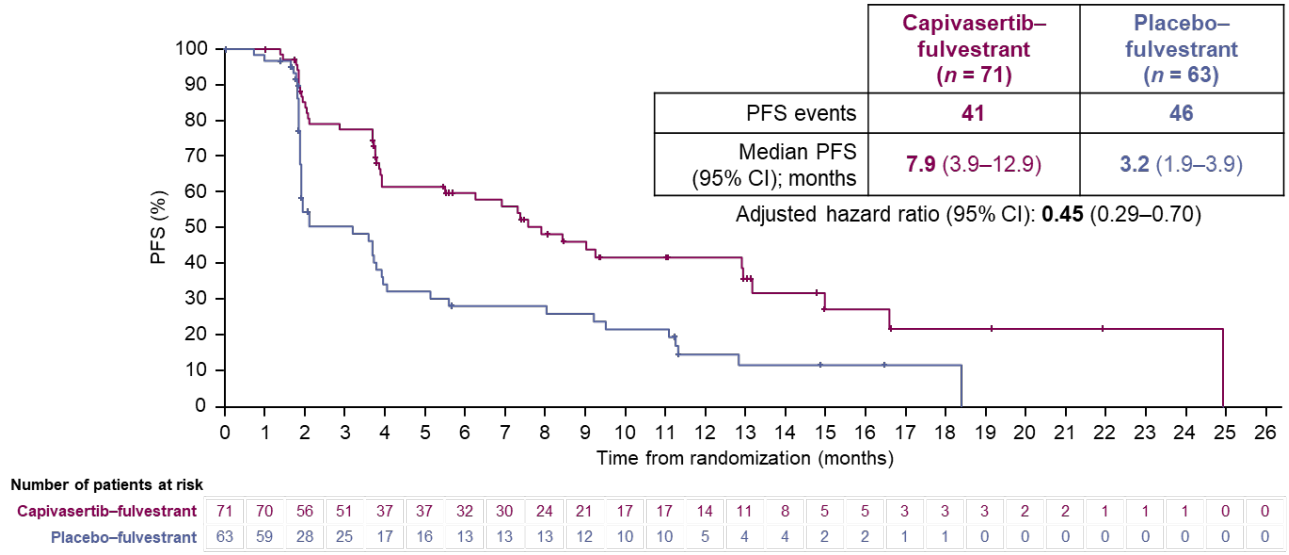


**b**

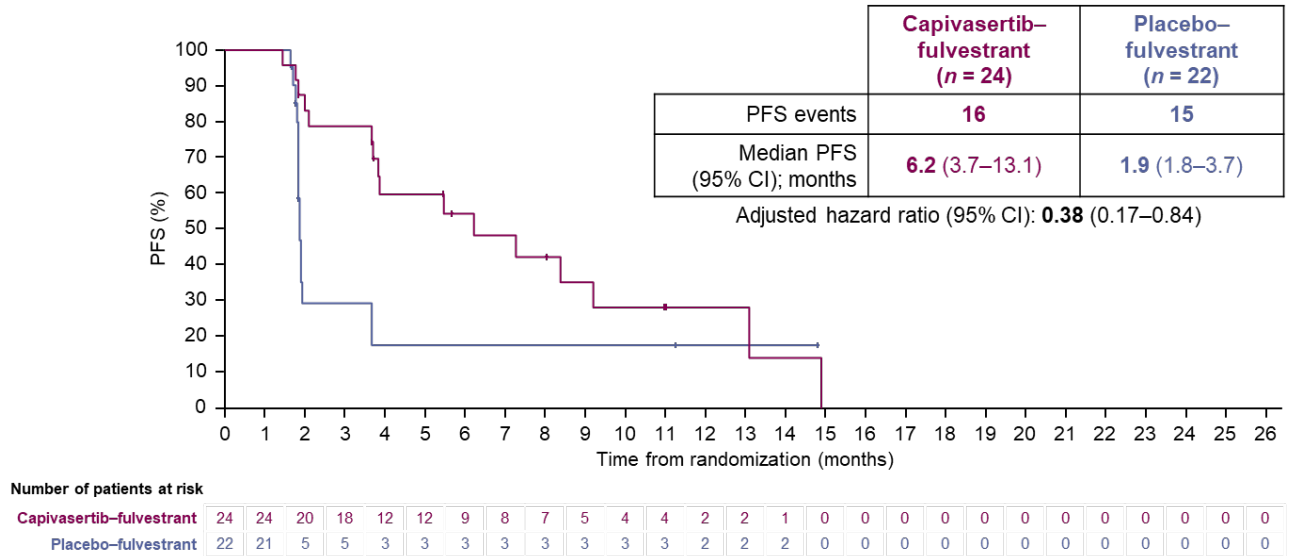


The hazard ratio of the capivasertib–fulvestrant (purple) and placebo–fulvestrant (blue) curves was estimated with the use of the Cox proportional hazards model, with stratification according to the presence or absence of liver metastases and previous use of a CDK4/6 inhibitor (yes or no) in the overall population, and according to previous CDK4/6 inhibitor use in the population of patients with *PIK3CA/AKT1/PTEN*-altered tumors. Tick marks indicate censored data. *AKT1*, Akt serine/threonine kinase 1; CDK4/6, cyclin-dependent kinase 4/6; CI, confidence interval; PFS,

progression-free survival; *PIK3CA*, catalytic subunit alpha of phosphatidylinositol-3-kinase; *PTEN*, phosphatase and tensin homolog.

**Supplementary Fig. 5 | Investigator-assessed PFS in the Chinese extended cohort in a, the overall population and b, patients with *PIK3CA/AKT1/PTEN*-altered tumors**

**a**

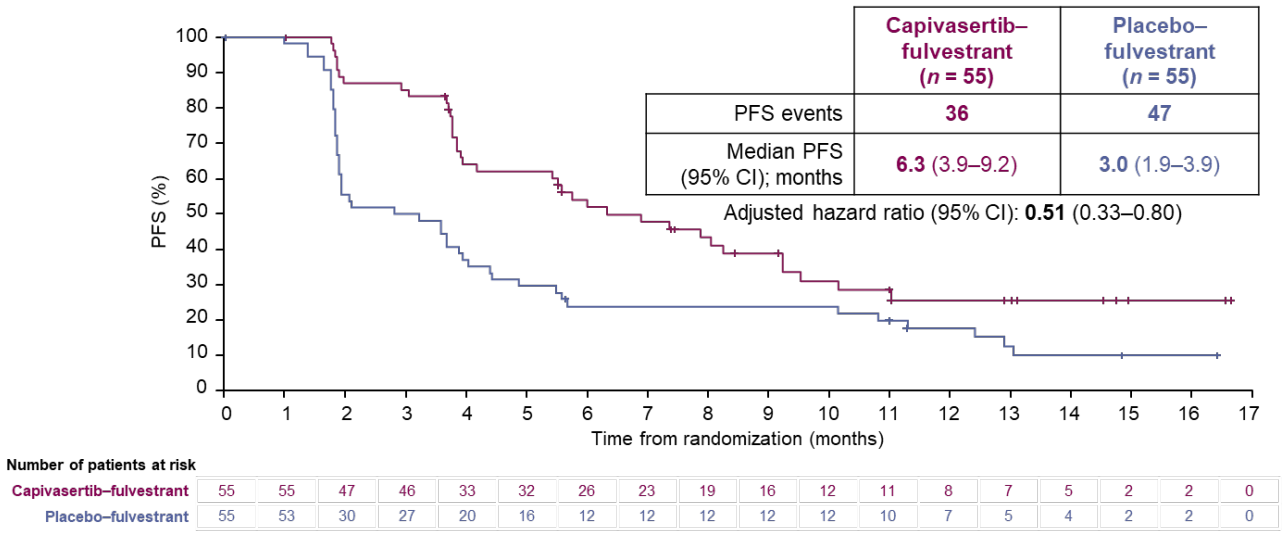


**b**

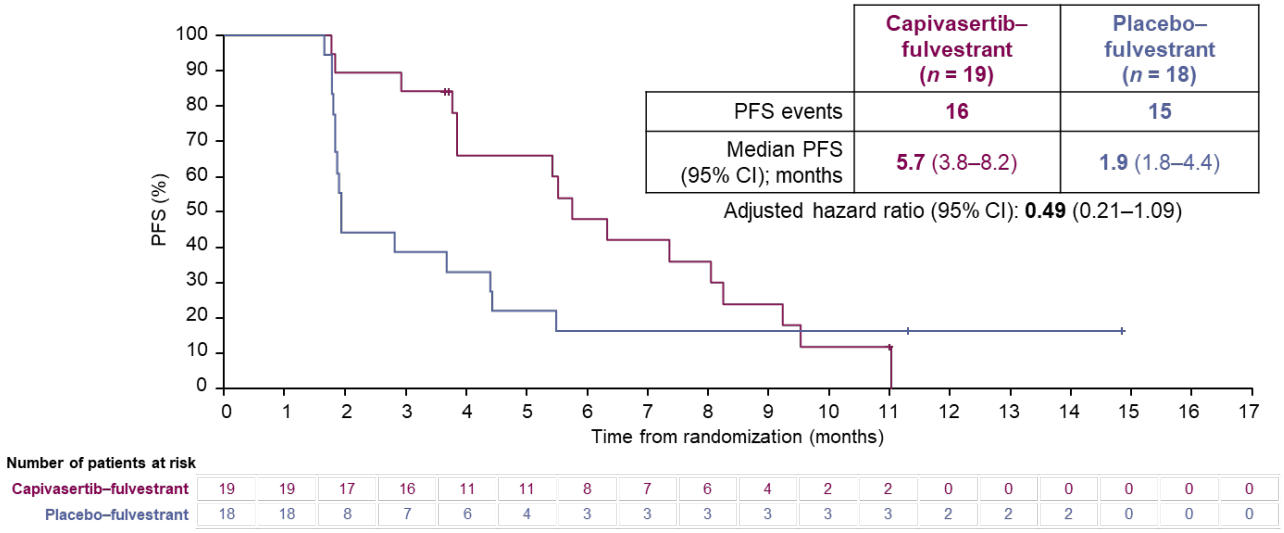


The hazard ratio of the capivasertib–fulvestrant (purple) and placebo–fulvestrant (blue) curves was estimated with the use of the Cox proportional hazards model, with stratification according to the presence or absence of liver metastases and previous use of a CDK4/6 inhibitor (yes or no) in the overall population, and according to previous CDK4/6 inhibitor use in the population of patients with *PIK3CA/AKT1/PTEN*-altered tumors. Tick marks indicate censored data. *AKT1*, Akt serine/threonine kinase 1; CDK4/6, cyclin-dependent kinase 4/6; CI, confidence interval; *PIK3CA*,

catalytic subunit alpha of phosphatidylinositol-3-kinase; PFS, progression-free survival; *PTEN*, phosphatase and tensin homolog.

**Supplementary Fig. 6 | Investigator-assessed PFS2 in a, the Chinese cohort overall population and b, patients with *PIK3CA/AKT1/PTEN*-altered tumors**

**a**

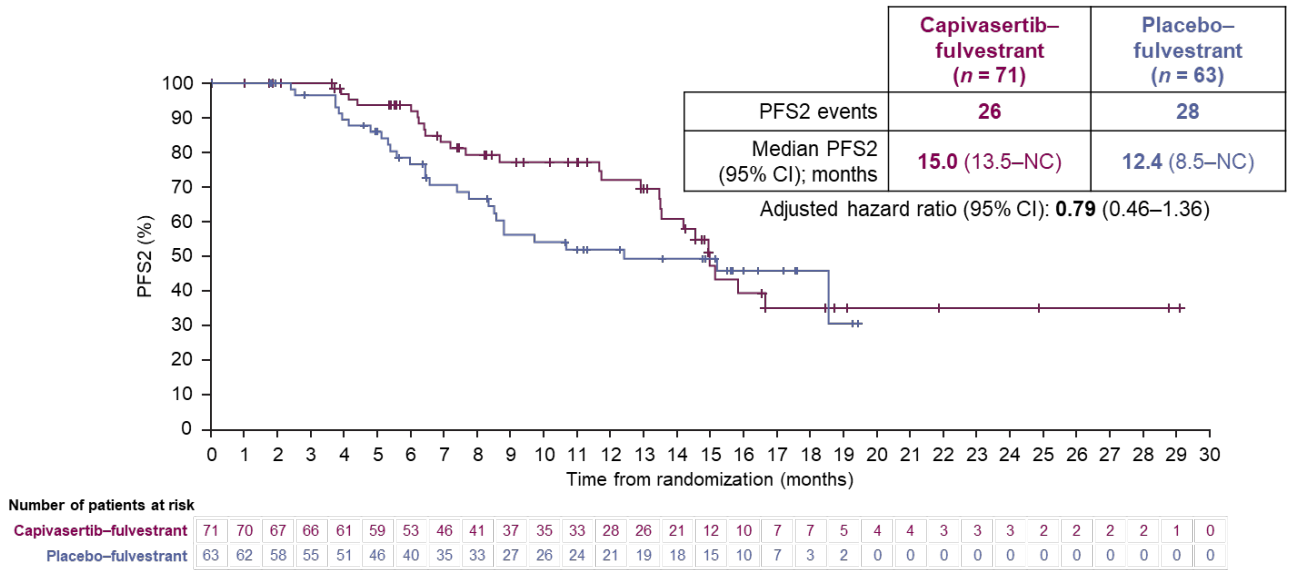


**b**

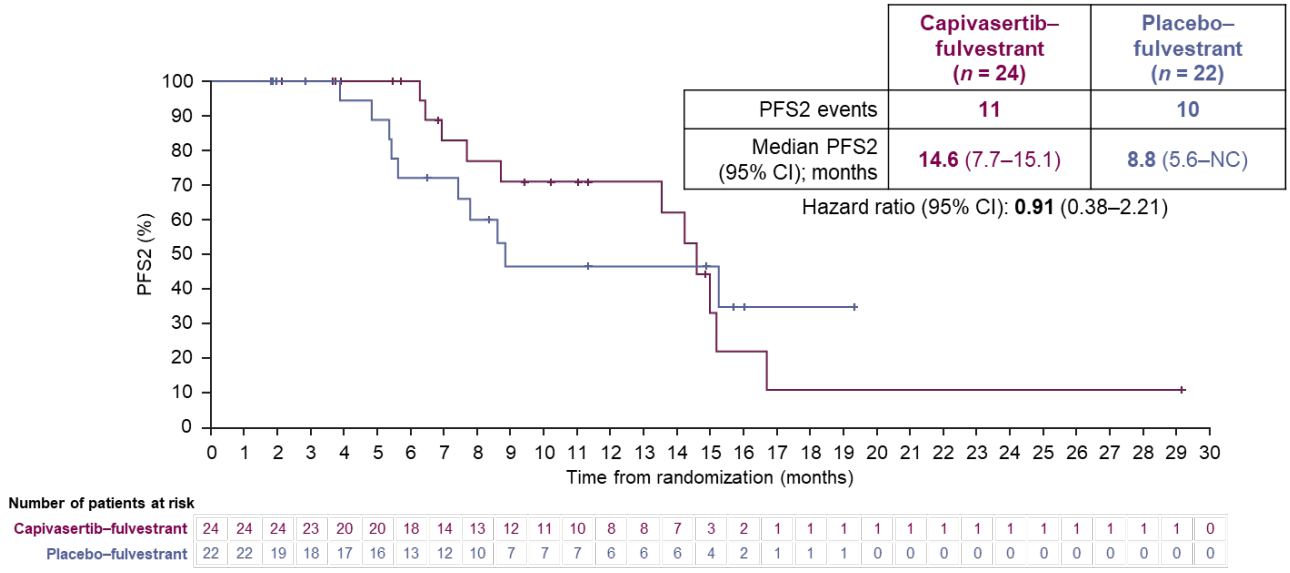


The hazard ratio of the capivasertib–fulvestrant (purple) and placebo–fulvestrant (blue) curves was estimated with the use of the Cox proportional hazards model, with stratification according to the presence or absence of liver metastases and previous use of a CDK4/6 inhibitor (yes or no) in the overall population. Tick marks indicate censored data. *AKT1*, Akt serine/threonine kinase 1; CDK4/6, cyclin-dependent kinase 4/6; CI, confidence interval; *PIK3CA*, catalytic subunit alpha of

phosphatidylinositol-3-kinase; PFS2, progression-free survival 2; *PTEN*, phosphatase and tensin homolog.

**Supplementary Fig. 7 | Change from baseline in EORTC QLQ-C30, by treatment visit**

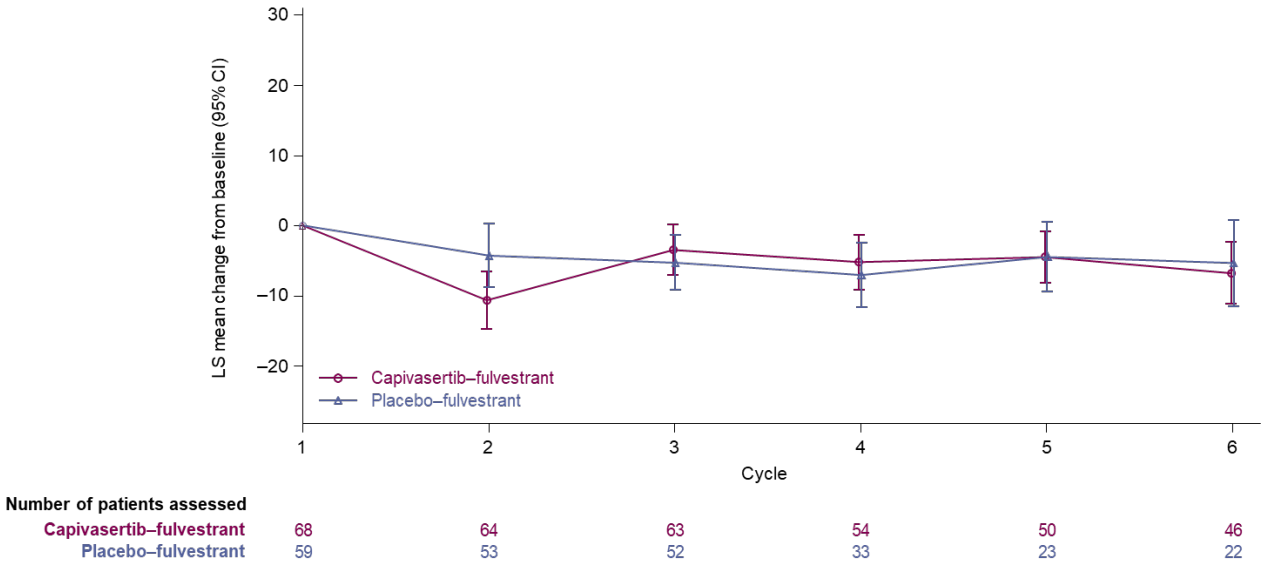


| Number of patients assessed | | | | | | |
|---|---|---|---|---|---|---|
| Capivasertib–fulvestrant | 68 | 64 | 63 | 54 | 50 | 46 |
| Placebo–fulvestrant | 59 | 53 | 52 | 33 | 23 | 22 |

Data are represented as LS mean changes from baseline (95% CI) for cycles with data for capivasertib–fulvestrant (purple) and placebo–fulvestrant (blue) treatment arms presented. Change from baseline was analyzed using a mixed effects model for all postbaseline scores for each visit. The model included treatment arm, visit, and treatment by visit interaction and stratification factors as explanatory variables, and the baseline score and baseline score by visit as covariates. Visits at each Cycle occurred on Day 1. Baseline was defined as the first assessment collected on Cycle 1 Day 1, prior to start of study treatment. For each treatment arm, summary statistics were presented at timepoints where in the respective treatment arm, ≥ 20 patients had an assessment and over one-third of patients had received treatment. CI, confidence interval; EORTC QLQ-C30, European Organisation for Research and Treatment of Cancer Quality of Life Questionnaire Core 30; LS, least squares.

| **Clinical Study Protocol** | |
|---|---|
| Drug Substance | Capivasertib (AZD5363) |
| Study Code | D3615C00001 |
| Version | 4.0 |
| Date | 07 February 2022 |

# A Phase III Double-blind Randomised Study Assessing the Efficacy and Safety of Capivasertib + Fulvestrant Versus Placebo + Fulvestrant as Treatment for Locally Advanced (Inoperable) or Metastatic Hormone Receptor Positive, Human Epidermal Growth Factor Receptor 2 Negative (HR+/HER2−) Breast Cancer Following Recurrence or Progression On or After Treatment with an Aromatase Inhibitor (CAPItello-291)

**Sponsor:**

AstraZeneca AB, S-151 85 Södertälje, Sweden.

AstraZeneca K.K., 3-1, Ofuka-cho, Kita-ku, Osaka 530-0011, Japan.

**Regulatory Agency Identifying Number(s):**

EudraCT number: 2019-003629-78

IND number: 118046

# VERSION HISTORY

| **Version 4.0, 08 February 2022** |
|---|
| **Sections 1.2, 1.3, 4.1, 9.2, 9.4, 9.5, 9.5.1 and 9.6**: Removal of the interim PFS analysis for the overall population to align with the change to the dual primary endpoints of PFS in the overall population and *PIK3CA/AKT1/PTEN*-altered sub-group. |
| **Sections 1.2, 3.1, 4.1 and 9.2** : Clarification of primary objectives. The study was designed with an alpha split between the overall population and the *PIK3CA/AKT1/PTEN*-altered subgroup, however, only the overall population was defined as the primary endpoint. To render the nomenclature consistent with the intent of the multiple testing procedure, the primary objective has been renamed as dual primary, i.e. PFS in the overall population and in the *PIK3CA/AKT1/PTEN*-altered sub-group. |
| **Section 1.2 and 3.2:** Duplication of all secondary objectives (except PK), i.e. to be assessed in both the overall population and the *PIK3CA/AKT1/PTEN*-altered subgroup, to align with the dual primary endpoints. |
| **Section 1.2:** ORR added to exploratory objective and definition added to endpoint |
| **Section 1.2, 4.4, 9.2, 9.4 and 9.5.1**: Clarification of the data cut-off trigger for primary PFS analysis as well as OS interim and final analysis; to align with the dual primary endpoints, the triggers of these DCOs are to be based on reaching the prespecified maturity both in the overall population and the *PIK3CA/AKT1/PTEN*-altered subgroup. |
| **Section 1.2 and 9.4.4:** Update of MTP. With the removal of PFS interim analysis, the 0.1% alpha originally reserved for the interim PFS analysis is now allocated to test PFS in the overall population. To maximise the chance of success in PFS in the *PIK3CA/AKT1/PTEN*-altered subgroup, the MTP has been updated to allow alpha recycling from PFS in the overall population to the *PIK3CA/AKT1/PTEN*-altered subgroup, with Spiessen Debois approach applied to the *PIK3CA/AKT1/PTEN*-altered subgroup using the correlation between overall population and the *PIK3CA/AKT1/PTEN*-altered subgroup. The testing sequence of the key secondary endpoints has been amended to follow the following hierarchical order: OS in the *PIK3CA/AKT1/PTEN*-altered subgroup, OS in the overall population, ORR in the overall population, ORR in the *PIK3CA/AKT1/PTEN*-altered subgroup. |
| **Section 9.2:** Updates to study design characteristics table 13 and table 14 to reflect the updates to the MTP. |

| |
|---|
| **Section 9.6:** The definition of altered subgroup China FAS and China altered subgroup safety analysis set has been added along with an updated timing for the China efficacy analysis to align with the updated planned analysis for the global cohort. |
| **Version 3.0, 29 June 2021** |
| **Sections 1.2, 3.2, and 9.4.1.8, Appendix J:** Clarified definition of $t_{max}$. |
| **Sections 1.2 and 9.3:** Clarified the FAS is to comprise patients randomised into the study, excluding patients randomised in China after the global cohort LPFV. |
| **Section 9.6:** Removed reference to Asian population analyses. |
| **Version 2.0, 03 June 2021** |
| **Section 1.1:** Included smoking history at screening in Schedule of assessments |
| **Section 1.1:** Added further clarification of visit windows in Schedule of assessments and throughout the protocol, where applicable. Updated the visit window for PRO assessments for the discontinuation of study treatment visit and the progression visit. |
| **Sections 1.1 and 8.1.7.8:** Clarified that PRO devices must be functional at baseline visit to ensure the assessments can be completed. Clarified text throughout on use of ePRO devices and how PRO questionnaires should be completed. |
| **Sections 1.1 and 8.7.1.3:** Clarified that results of central testing of the optional tumour biopsy collected at disease progression will be provided to the investigator, if available. |
| **Section 1.1:** Added collection of circulating soluble factor, whole blood DNA sample, and whole blood RNA samples at discontinuation of IMP if it falls on a different visit/time than progression. |
| **Sections 1.1, 1.2, 5.1, and 6.5.1:** Updated administration of LHRH agonist in pre- or peri-menopausal women must start prior to or on Cycle 1, Day 1. |
| **Section 1.1:** Clarified that if fulvestrant and capivasertib/placebo are both discontinued at the same time as progression, the discontinuation visits are combined and the optional biopsy is completed, if applicable. |
| **Sections 1.2 and 3.2:** Clarified secondary objective for PK to evaluate the PK of capivasertib (when given in combination with fulvestrant). |

| |
|---|
| **Sections 1.2 and 9.3:** Clarified FAS to comprise patients randomised into the study prior to global cohort LPFV; clarified the Safety Analysis Set will also include patients who receive only fulvestrant and these patients will be included in the treatment arm to which they were randomised; clarified the Altered Subgroup FAS will include patients with a result from a valid biomarker test as pre-specified in the SAP. |
| **Sections 1.2 and 9.6:** Clarified the China cohort. |
| **Sections 2.3.3 and 4.5:** Added text for impact of COVID-19 on the study conduct. |
| **Section 5.1:** Clarified Inclusion criterion for confirmation of breast cancer HER2- |
| **Section 5.1:** Clarified Inclusion criterion of metastatic or locally advanced disease with radiological or objective evidence of disease progression (the cancer should have shown progression during or after most recent therapy). |
| **Section 5.2:** Clarified Exclusion criterion for cardiac criteria as QT interval corrected by Fridericia's formula. |
| **Section 5.2:** Clarified Exclusion criterion for severe or uncontrolled systemic diseases (as judged by the investigator) to include those with confirmed COVID-19, for example. |
| **Section 5.2:** Clarified Exclusion criterion for participation in another clinical study and to clarify vaccines for COVID-19 are not considered investigational agents. |
| **Sections 1.1, 5.3.3, and 8.2.1:** Updated that FSH and oestradiol will be monitored at screening, at Day 1 of each cycle, and when clinically indicated in pre- and peri-menopausal women (per Inclusion criteria) and only at screening and when clinically indicated in some post-menopausal women. |
| **Section 6.1.1:** Clarified the treatment administered to dosing of capivasertib/placebo 400 mg BD (2 tablets of 200 mg taken twice a day = total daily dose 800 mg) |
| **Sections 5.2 and 6.5.1:** Added clarification that authorised/approved COVID-19 vaccines are allowed concomitant therapy. |
| **Section 8.2.1:** Removed testosterone from safety laboratory variables testing as this is no longer a safety requirement for capivasertib. |
| **Section 8.2.3:** Updated Vital signs subsection to include respiratory rate. |

| |
|---|
| **Section 8.3.10:** Added a subsection for Adverse Events of Special Interest. |
| **Section 8.7.1:** Clarified text regarding tumour tissue sampling, including a clarification for archival bone biopsies and analysis results of the optional tumour biopsy will be provided to the investigator, if available. |
| **Section 9.4.4:** Added text to specify the hierarchical testing order to test secondary endpoints after OS. |
| **Appendix D and Sections 5.2, 6.5.2:** Added MATE1 and OCT2 transporter substrates under the appendix of drugs that may be influenced by capivasertib. Removed the restriction of CYP2D6 and CYP2C9 substrates. |
| **Appendix K and L:** Added new appendices for changes related to mitigation of study disruptions and guidance for the COVID-19 outbreak. |
| **Version 1.0, 04 November 2019** |
| Initial creation |

This Clinical Study Protocol has been subjected to peer review according to AstraZeneca Standard procedures. The Clinical Study Protocol is publicly registered and the results are disclosed and/or published according to the AstraZeneca Global Policy on Bioethics and in compliance with prevailing laws and regulations.

# TABLE OF CONTENTS

TITLE PAGE ..... 1
VERSION HISTORY ..... 2
TABLE OF CONTENTS ..... 6
1 PROTOCOL SUMMARY ..... 11
1.1 Schedule of Activities (SoA) ..... 11
1.2 Synopsis ..... 21
1.3 Schema ..... 32
2 INTRODUCTION ..... 33
2.1 Study rationale ..... 33
2.2 Background ..... 33
2.3 Benefit/risk assessment ..... 37
2.3.1 Risks with capivasertib ..... 37
2.3.2 Overall benefit-risk and ethical assessment ..... 38
2.3.3 Impact on Benefit/Risk from Study Disruptions due to Coronavirus Disease 2019 ..... 39
3 OBJECTIVES AND ENDPOINTS ..... 40
3.1 Primary objectives ..... 40
3.2 Secondary objectives ..... 40
3.3 Exploratory objectives ..... 42
4 STUDY DESIGN ..... 44
4.1 Overall design ..... 44
4.1.1 Study oversight considerations ..... 48
4.2 Scientific rationale for study design ..... 48
4.3 Justification for dose ..... 52
4.3.1 Capivasertib ..... 52
4.3.2 Fulvestrant ..... 53
4.4 End of study definition ..... 53
4.5 Study Conduct Mitigation During Study Disruptions Due to COVID-19 – Temporary Measures ..... 54
5 STUDY POPULATION ..... 55
5.1 Inclusion criteria ..... 55
5.2 Exclusion criteria ..... 58
5.3 Lifestyle restrictions ..... 61
5.3.1 Meals and dietary restrictions ..... 61
5.3.2 Caffeine, alcohol, and tobacco ..... 62
5.3.3 Contraception ..... 62
5.3.3.1 Females ..... 62
5.3.3.2 Males ..... 63

5.3.4 Other restrictions ..... 63
5.4 Screen failures ..... 63
6 STUDY TREATMENTS ..... 63
6.1 Treatments administered ..... 64
6.1.1 Capivasertib/placebo ..... 64
6.1.2 Fulvestrant ..... 65
6.1.3 Duration of treatment ..... 66
6.2 Preparation/handling/storage/accountability ..... 66
6.3 Measures to minimise bias: randomisation and blinding ..... 67
6.4 Treatment compliance ..... 68
6.5 Concomitant therapy ..... 68
6.5.1 Allowed concomitant therapy ..... 68
6.5.2 Restricted concomitant therapy ..... 70
6.5.3 Other concomitant treatment ..... 70
6.6 Dose modifications and delay ..... 70
6.7 Treatment after the end of the study ..... 71
7 DISCONTINUATION OF TREATMENT AND PATIENT WITHDRAWAL . 72
7.1 Discontinuation of study treatment ..... 72
7.1.1 Temporary discontinuation ..... 73
7.1.2 Procedures for discontinuation of study treatment ..... 73
7.2 Lost to follow-up ..... 73
7.3 Withdrawal from the study ..... 74
8 STUDY ASSESSMENTS AND PROCEDURES ..... 74
8.1 Efficacy assessments ..... 75
8.1.1 RECIST v1.1 assessments ..... 75
8.1.1.1 Progression-free survival (PFS) ..... 76
8.1.1.2 Clinical benefit rate (CBR) ..... 76
8.1.1.3 Duration of response (DoR) ..... 76
8.1.1.4 Objective response rate (ORR) ..... 76
8.1.2 Overall survival (OS) ..... 77
8.1.3 PFS2 ..... 77
8.1.4 Time to definitive deterioration of the ECOG performance status ..... 78
8.1.5 Time to first subsequent chemotherapy or death (TFSC) ..... 78
8.1.6 Bone scan ..... 78
8.1.7 Patient-reported outcomes ..... 78
8.1.7.1 EORTC QLQ-C30 ..... 79
8.1.7.2 EORTC QLQ-BR23 ..... 79
8.1.7.3 EQ-5D-5L ..... 79
8.1.7.4 PGIS ..... 80
8.1.7.5 PGIC ..... 80
8.1.7.6 PGI-TT ..... 80
8.1.7.7 PRO-CTCAE ..... 80
8.1.7.8 Administration of patient-reported outcome questionnaires ..... 80

8.2 Safety assessments ..... 82
8.2.1 Clinical safety laboratory assessments ..... 82
8.2.1.1 Glucose and glycosylated haemoglobin (HbA1c) ..... 84
8.2.1.2 Serum creatinine ..... 84
8.2.2 Physical examinations ..... 84
8.2.3 Vital signs ..... 84
8.2.4 Electrocardiograms ..... 85
8.2.5 MUGA scan/Echocardiogram ..... 85
8.3 Collection of adverse events ..... 86
8.3.1 Method of detecting AEs and SAEs ..... 86
8.3.2 Time period and frequency for collecting AE and SAE information ..... 86
8.3.3 Follow-up of AEs and SAEs ..... 87
8.3.4 Adverse event data collection ..... 87
8.3.5 Causality collection ..... 88
8.3.6 Adverse events based on signs and symptoms ..... 88
8.3.7 Adverse events based on examinations and tests ..... 88
8.3.8 Hy's law ..... 89
8.3.9 Disease progression ..... 89
8.3.10 Adverse Events of Special Interest ..... 89
8.4 Safety reporting and medical management ..... 90
8.4.1 Reporting of serious adverse events ..... 90
8.4.1.1 Regulatory Reporting Requirements for SAEs ..... 91
8.4.2 Pregnancy ..... 91
8.4.2.1 Maternal exposure ..... 91
8.4.2.2 Paternal exposure ..... 92
8.4.3 Overdose ..... 92
8.4.4 Medication error ..... 93
8.4.5 Management of capivasertib-related toxicities ..... 93
8.4.5.1 Capivasertib/placebo dose modification and guidance ..... 93
8.4.5.2 Dose modifications due to general capivasertib-related toxicities ..... 93
8.4.5.3 Dose modifications due to specific capivasertib-related toxicities ..... 94
8.5 Pharmacokinetics ..... 99
8.5.1 Determination of drug concentration ..... 100
8.5.2 Storage and destruction of pharmacokinetic samples ..... 100
8.5.2.1 Samples collected in China ..... 100
8.5.2.2 Samples collected outside of China ..... 100
8.6 Genetics ..... 101
8.6.1 Optional exploratory genetic sample ..... 101
8.6.2 Storage and destruction of genetic samples ..... 101
8.7 Biomarkers ..... 101
8.7.1 Tumour tissue sample ..... 101
8.7.1.1 Mandatory tumour tissue samples for biomarker analysis ..... 101
8.7.1.2 Optional tumour tissue samples for exploratory biomarker research ..... 102
8.7.1.3 Collection, analysis and reporting of tumour samples ..... 103
8.7.2 Mandatory blood samples for exploratory biomarker analysis ..... 103

8.7.3 Storage, re-use and destruction of biomarker samples ..... 104
8.8 Healthcare resource use ..... 104
9 STATISTICAL CONSIDERATIONS ..... 105
9.1 Statistical hypotheses ..... 105
9.2 Sample size determination ..... 105
9.3 Populations for analyses ..... 108
9.4 Statistical analyses ..... 109
9.4.1 Efficacy analyses ..... 109
9.4.1.1 Primary endpoint: PFS in the overall population ..... 109
9.4.1.2 Primary endpoint: PFS in the *PIK3CA/AKT1/PTEN*-altered subgroup ..... 110
9.4.1.3 Secondary endpoint: OS in the overall population ..... 110
9.4.1.4 Secondary endpoint: OS in the *PIK3CA/AKT1/PTEN*-altered subgroup ..... 110
9.4.1.5 Secondary endpoint: PFS2 in the overall and altered FAS population ..... 111
9.4.1.6 Secondary endpoint: ORR in the overall and altered FAS population ..... 111
9.4.1.7 Secondary endpoint: DoR in the overall and altered FAS population ..... 111
9.4.1.8 Secondary endpoint: CBR in the overall and altered FAS population ..... 111
9.4.1.9 Secondary endpoint: PK ..... 111
9.4.1.10 Secondary endpoints: EORTC QLQ-C30 and EORTC QLQ-BR23 ..... 112
9.4.1.11 Secondary endpoint: time to definitive deterioration of the ECOG performance status in the overall population and the *PIK3CA/AKT1/PTEN*-altered population ..... 113
9.4.2 Safety analyses ..... 113
9.4.2.1 Adverse events ..... 114
9.4.2.2 Other safety analysis ..... 115
9.4.3 Other analyses ..... 115
9.4.4 Methods for multiplicity control ..... 115
9.5 Interim analyses ..... 116
9.5.1 Independent data monitoring committee (IDMC) ..... 116
9.6 China cohort ..... 116
10 REFERENCES ..... 118
11 SUPPORTING DOCUMENTATION AND OPERATIONAL CONSIDERATIONS ..... 125

## LIST OF TABLES

Table 1 Schedule of assessments (SoA) ..... 12
Table 2 Primary objectives ..... 40
Table 3 Secondary objectives ..... 40
Table 4 Exploratory objectives ..... 42
Table 5 Capivasertib/placebo ..... 64
Table 6 Fulvestrant ..... 65

Table 7 Dose levels for capivasertib/placebo and fulvestrant ..... 71
Table 8 Laboratory safety variables ..... 83
Table 9 Dose modifications for general capivasertib-related toxicities ..... 94
Table 10 Dose modifications for capivasertib-related hyperglycaemia[a] ..... 95
Table 11 Dose modifications for capivasertib-related maculo-papular rash ..... 97
Table 12 Dose modifications for capivasertib-related diarrhoea ..... 99
Table 13 Statistical considerations for dual primary PFS endpoints ..... 106
Table 14 Statistical considerations for OS endpoint ..... 107
Table 15 Analysis populations ..... 108
Table 16 Summary of imaging modalities for tumour assessment ..... 126
Table 17 RECIST v1.1 evaluation of target lesions ..... 133
Table 18 RECIST v1.1 evaluation of non-target lesions ..... 134
Table 19 RECIST v1.1 overall visit response ..... 134
Table 20 ECOG performance status scale (Oken et al 1982) ..... 137
Table 21 CYP3A4-interacting medication that must be avoided or used with caution ..... 144
Table 22 CYP3A4 substrates that must be avoided or used with caution ..... 146
Table 23 MATE1 and/or OCT2 transporter substrates that should be used with caution ..... 146

## LIST OF FIGURES

Figure 1 Study design ..... 32
Figure 2 Study flow chart ..... 47
Figure 3 Treatment schedule ..... 65
Figure 4 Illustration of data cut-offs and associated treatment comparisons ..... 115

## LIST OF APPENDICES

**Appendix A** Guidelines for evaluation of objective tumour response using RECIST v1.1 Criteria (Response Evaluation Criteria in Solid Tumours) 126
**Appendix B** ECOG/WHO Performance Status ..... 137
**Appendix C** Regulatory, ethical and study oversight considerations ..... 138
**Appendix D** Guidance regarding potential interactions of capivasertib with concomitant medications ..... 144

**Appendix E** Adverse event definitions and additional safety information ..... 148
**Appendix F** Handling of human biological samples ..... 153
**Appendix G** Genetics..... 156
**Appendix H** Actions required in cases of increases in liver biochemistry and evaluation of Hy's law ..... 160
**Appendix I** Patient-reported outcomes – questionnaires..... 164
**Appendix J** Abbreviations ..... 175
**Appendix K** Changes Related to Mitigation of Study Disruptions Due to COVID-19 Outbreak - Temporary Measures..... 179
**Appendix L** Guidance during the COVID-19 Outbreak ..... 182

# 1 PROTOCOL SUMMARY

## 1.1 Schedule of Activities (SoA)

The schedule of study assessments is provided in Table 1.

## Table 1 Schedule of assessments (SoA)

| | Screen | Cycle 1[a] | | | Cycle 2[a, c] | | Cycle 3[a, c] → | Discontinuation[v] | | Post-treatment follow-up | Progres-sion | Survival follow-up | Details in CSP section or appendix |
|---|---|---|---|---|---|---|---|---|---|---|---|---|---|
| | | | | | | | | Fulvestrant | Capivasertib/ placebo | 30 days after last dose | | Every 8 weeks | |
| **Week** | | 1 | 3 | | 1 | 3 | 1 | | | | | | |
| **Day of week** | -28 to 0 | 1 | 1 | 4 | 1 | 1 | 1 | | | | | | |
| **Visit window (days)[b]** | | 0 | -1/+3 | -1/+3 | -1/+3 | -1/+3 | -1/+3 | +7 | +7 | +7 | +7 | ±7 | |
| Informed consent | X | | | | | | | | | | | | Section 5.1, Appendix C |
| Inclusion/exclusion criteria | X | | | | | | | | | | | | Sections 5.1 & 5.2 |
| Prepare ePRO device[d] | | X | | | | | | | | | | | Section 8.1.7.8 |
| Train patient on ePRO device[e] | | X | | | | | | | | | | | Section 8.1.7.8 |
| **Routine clinical procedures[f]** | | | | | | | | | | | | | |
| Demography/baseline characteristics | X | | | | | | | | | | | | |
| Medical/surgical history | X | | | | | | | | | | | | Sections 5.1 & 5.2 |
| Smoking history | X | | | | | | | | | | | | |
| Previous cancer therapy | X | | | | | | | | | | | | Section 5.2 |
| *BRCA1/2* status (local testing), if available | X | | | | | | | | | | | | |
| Concomitant medication | X | At every study visit | | | | | | | | | | | Section 6.5 |
| ECOG/WHO performance status | X | X | X | | X | X | X | X | X | X | | | Appendix B |

| | Screen | Cycle 1[a] | | | Cycle 2[a, c] | | Cycle 3[a, c] → | Discontinuation[v] | | Post-treatment follow-up | Progres-sion | Survival follow-up | Details in CSP section or appendix |
|---|---|---|---|---|---|---|---|---|---|---|---|---|---|
| | | | | | | | | Fulvestrant | Capivasertib/ placebo | 30 days after last dose | | Every 8 weeks | |
| **Week** | | **1** | **3** | | **1** | **3** | **1** | | | | | | |
| **Day of week** | **-28 to 0** | **1** | **1** | **4** | **1** | **1** | **1** | | | | | | |
| **Visit window (days)[b]** | | **0** | **-1/+3** | **-1/+3** | **-1/+3** | **-1/+3** | **-1/+3** | **+7** | **+7** | **+7** | **+7** | **±7** | |
| Physical examination | X | X | | | X | | X | X | X | X | | | Section 8.2.2 |
| Vital signs | X | Pre (within 30m) and 1-2h post (±30m) | X | | X | X | X | X | X | X | | | Section 8.2.3 |
| Height | X | | | | | | | | | | | | Section 8.2.2 |
| Weight | X | X | | | X | | X | X | X | X | | | Section 8.2.2 |
| 12-lead ECG | X | Pre (within 30m) and 1h post (±30m) | | | Cycle 2, Week 1, Day 1 and every 12 weeks thereafter | | | | X | | | | Section 8.2.4 |
| MUGA/ECHO (LVEF)[g] | X | As clinically indicated during study treatment | | | | | | | | | | | Section 8.2.5 |
| **Routine safety measurements[f]** | | | | | | | | | | | | | |
| Adverse events | X | At every study visit | | | | | | | | | | | Section 8.3, Appendix E |
| Pregnancy test (peri-/pre-menopausal only) | X | X | As clinically indicated and at treatment discontinuation | | | | | | | | | | Section 8.4.2 |
| Oestradiol and FSH[h] | X | X | | | X | | X | | | | | | Section 8.2.1 |
| International Normalised Ratio (INR) | X | As clinically indicated | | | | | | | | | | | Section 8.2.1 |
| Haematology[c] | X | Pre | Pre | | Pre | Pre | X | X | X | | | | Section 8.2.1 |

| | Screen | Cycle 1[a] | | | Cycle 2[a, c] | | Cycle 3[a, c] → | Discontinuation[v] | | Post-treatment follow-up | Progression | Survival follow-up | Details in CSP section or appendix |
|---|---|---|---|---|---|---|---|---|---|---|---|---|---|
| | | | | | | | | Fulvestrant | Capivasertib/ placebo | 30 days after last dose | | Every 8 weeks | |
| **Week** | | **1** | **3** | | **1** | **3** | **1** | | | | | | |
| **Day of week** | **-28 to 0** | **1** | **1** | **4** | **1** | **1** | **1** | | | | | | |
| **Visit window (days)[b]** | | **0** | **-1/+3** | **-1/+3** | **-1/+3** | **-1/+3** | **-1/+3** | **+7** | **+7** | **+7** | **+7** | **±7** | |
| Clinical chemistry[c,i] | X | Pre | Pre | | Pre | Pre | X | X | X | | | | Section 8.2.1 |
| Urinalysis[c] | X | Pre | Pre | | Pre | Pre | X | X | X | | | | Section 8.2.1 |
| Glycosylated haemoglobin (fasting)[c,j] | X | Every 12 weeks from Cycle 1, Week 1, Day 1 | | | | | | X | X | | | | Section 8.2.1 |
| Lipids (fasting)[c,j] | X | Every 12 weeks from Cycle 1, Week 1, Day 1 | | | | | | | X | | | | Section 8.2.1 |
| Glucose (fasting)[c,j] | X | Pre (within 3h) and 4h post (±30m) | Pre | | Pre | | X | | X | | | | Section 8.2.1 |
| **Biomarker analysis** | | | | | | | | | | | | | |
| FFPE tumour sample[k] | X | | | | | | | | | | | | Section 8.7.1.1 |
| Single biopsy at disease progression (optional)[l] | | | | | | | | | | | X | | Section 8.7.1.2 |
| Paired biopsies (optional)[l,m] | X | | | X | | | | | | | | | Section 8.7.1.2 |
| ctDNA blood samples[c,l] | X | Pre | Pre | | Pre | | Pre | X | X | | X | | Section 8.7.2 |
| Circulating soluble factors blood draw[c,l,n] | X | Pre | Pre | | Pre | | Pre, C3 only | X | X | | X | | Section 8.7.2 |

| | Screen | Cycle 1[a] | | | Cycle 2[a, c] | | Cycle 3[a, c] → | Discontinuation[v] | | Post-treatment follow-up | Progres-sion | Survival follow-up | Details in CSP section or appendix |
|---|---|---|---|---|---|---|---|---|---|---|---|---|---|
| | | | | | | | | Fulvestrant | Capivasertib/ placebo | 30 days after last dose | | Every 8 weeks | |
| **Week** | | **1** | **3** | | **1** | **3** | **1** | | | | | | |
| **Day of week** | **-28 to 0** | **1** | **1** | **4** | **1** | **1** | **1** | | | | | | |
| **Visit window (days)[b]** | | **0** | **-1/+3** | **-1/+3** | **-1/+3** | **-1/+3** | **-1/+3** | **+7** | **+7** | **+7** | **+7** | **±7** | |
| Whole blood DNA sample (genomic markers)[c,l,n] | X | Pre | Pre | | Pre | | Pre, C3 only | X | X | | X | | Section 8.7.2 |
| Whole blood RNA sample (genomic markers)[c,l,n] | X | Pre | Pre | | Pre | | Pre, C3 only | X | X | | X | | Section 8.7.2 |
| **Genetic sample (optional)** | | | | | | | | | | | | | |
| Blood sample for pharmacogenetics DNA (optional)[l] | X | | | | | | | | | | | | Section 8.6 |
| **Pharmacokinetic measurements[c]** | | | | | | | | | | | | | |
| Capivasertib PK plasma samples | | 1h (±10m) and 4h (±30m) post-dose | Pre (<30 m) | | Pre (<30 m) | Pre (<30 m) | | | | | | | Section 8.5 |
| Capivasertib PK plasma samples for 12 Japanese patients only | | Pre (within 30m) and 0.5 (±5m), 1 (±10m), 2 (±10m), 4 (±30m), 6 (±30m), 8 (±30m), 12h (±1h) post-dose | Pre (<30 m) | | Pre (<30 m) | Pre (<30 m) | | | | | | | Section 8.5 |

| | Screen | Cycle 1[a] | | | Cycle 2[a, c] | | Cycle 3[a, c] → | Discontinuation[v] | | Post-treatment follow-up | Progression | Survival follow-up | Details in CSP section or appendix |
|---|---|---|---|---|---|---|---|---|---|---|---|---|---|
| | | | | | | | | Fulvestrant | Capivasertib/ placebo | 30 days after last dose | | Every 8 weeks | |
| **Week** | | **1** | **3** | | **1** | **3** | **1** | | | | | | |
| **Day of week** | **-28 to 0** | **1** | **1** | **4** | **1** | **1** | **1** | | | | | | |
| **Visit window (days)[b]** | | **0** | **-1/+3** | **-1/+3** | **-1/+3** | **-1/+3** | **-1/+3** | **+7** | **+7** | **+7** | **+7** | **±7** | |
| **Imaging and other assessments** | | | | | | | | | | | | | |
| Bone scan | X | As clinically indicated | | | | | | | | | | | Section 8.1.6 |
| RECIST v1.1 tumour assessments[o] | X | Every 8 weeks (±7 days) for the first 18 months and every 12 weeks thereafter, from randomisation to radiological progression. Patients who discontinue treatment prior to progression should continue to be scanned until progression. | | | | | | | | | | | Section 8.1.1 & Appendix A |
| Survival status[p] | | | | | | | | | | X | | X | Section 8.1.2 |
| PFS2[q] | | | | | | | | | | X | | X | Section 8.1.3 |
| Subsequent cancer therapy following discontinuation of study treatment[q] | | | | | | | | | | X | | X | Section 6.7 |
| **Patient-reported outcomes and healthcare resource utilisation** | | | | | | | | | | | | | |
| EORTC QLQ-C30 | | Cycle 1, Week 1, Day 1 (-3 days) and every 4 weeks (±3 days) until PFS2. Also at discontinuation of study treatment visit (+3 days) and, for those who discontinue for reasons other than progression, also at progression visit (+3 days). If PROs have been completed up to 3 days prior to the discontinuation or progression visit, they do not need to be repeated. | | | | | | | | | | | Section 8.1.7.1 |
| EORTC QLQ-BR23 | | | | | | | | | | | | | Section 8.1.7.2 |
| EQ-5D-5L | | | | | | | | | | | | | Section 8.1.7.3 |
| PGIS | | | | | | | | | | | | | Section 8.1.7.4 |

| | Screen | Cycle 1[a] | | | Cycle 2[a, c] | | Cycle 3[a, c] → | Discontinuation[v] | | Post-treatment follow-up | Progres-sion | Survival follow-up | Details in CSP section or appendix |
|---|---|---|---|---|---|---|---|---|---|---|---|---|---|
| | | | | | | | | Fulvestrant | Capivasertib/ placebo | 30 days after last dose | | Every 8 weeks | |
| **Week** | | **1** | **3** | | **1** | **3** | **1** | | | | | | |
| **Day of week** | **-28 to 0** | **1** | **1** | **4** | **1** | **1** | **1** | | | | | | |
| **Visit window (days)[b]** | | **0** | **-1/+3** | **-1/+3** | **-1/+3** | **-1/+3** | **-1/+3** | **+7** | **+7** | **+7** | **+7** | **±7** | |
| PGIC | | | | | Every 4 weeks (±3 days) after Cycle 1, Week 1, Day 1 until PFS2. Also at discontinuation of study treatment visit (+3 days) and, for those who discontinue for reasons other than progression, also at progression visit (+3 days). If PROs have been completed up to 3 days prior to discontinuation or progression visit, they do not need to be repeated. | | | | | | | | Section 8.1.7.5 |
| PGI-TT | | Cycle 1, Week 1, Day 1 (-3 days) and every 2 weeks (±2 days) up to and including Week 12 and then every 4 weeks (±3 days) until discontinuation of study treatment, at discontinuation of study treatment visit (+3 days) and at 4 weeks (±3 days) post discontinuation of study treatment visit. If PROs have been completed up to 3 days prior to discontinuation visit, they do not need to be repeated. | | | | | | | | | | | Section 8.1.7.6 |
| PRO-CTCAE | | | | | | | | | | | | | Section 8.1.7.7 |
| Healthcare resource use (HOSPAD)[r] | | Healthcare resource use module is event driven and should be populated as required at every study visit | | | | | | | | | | | Section 8.8 |
| **Study treatment administration** | | | | | | | | | | | | | |
| Randomisation[s] | | X | | | | | | | | | | | Section 6.3 |
| Fulvestrant dosing | | X | X | | X | | X | | | | | | Section 6.1.2 |
| Capivasertib/placebo dosing[t] | | Twice daily on days 1 to 4 every week (ie, 4 days on and 3 days off). A minimum interval of 3 days is required between the last dose of the previous week of treatment and the first dose of the following week of treatment | | | | | | | | | | | Section 6.1.1 |

| | Screen | Cycle 1[a] | | | Cycle 2[a, c] | | Cycle 3[a, c] → | Discontinuation[v] | | Post-treatment follow-up | Progres-sion | Survival follow-up | Details in CSP section or appendix |
|---|---|---|---|---|---|---|---|---|---|---|---|---|---|
| | | | | | | | | Fulvestrant | Capivasertib/ placebo | 30 days after last dose | | Every 8 weeks | |
| **Week** | | **1** | **3** | | **1** | **3** | **1** | | | | | | |
| **Day of week** | **-28 to 0** | **1** | **1** | **4** | **1** | **1** | **1** | | | | | | |
| **Visit window (days)[b]** | | **0** | **-1/+3** | **-1/+3** | **-1/+3** | **-1/+3** | **-1/+3** | **+7** | **+7** | **+7** | **+7** | **±7** | |
| LHRH agonist dosing (for pre-/peri-menopausal women only; both treatment arms) | X[u] | Sub-cutaneous administration every 28 days or as per manufacturer's instructions | | | | | | | | | | | Sections 5.1, 5.3.3.1, and 6.5.1 |

NOTE: Unscheduled visits may be initiated as needed. Only required assessments are to be performed as per investigator's discretion.

[a] Specified times (eg, 'Pre', '1-2h post', '4h post', etc) refer to capivasertib/placebo dosing.

[b] Study visits are scheduled relative to Week 1, Day 1 of each cycle. As per protocol, the visit window is -1/+3 days for most visits, **however. a -1 day window cannot be used for study treatment dosing and should only be used for labs and other assessments**.

[c] Screening laboratory assessments taken within 3 days of Cycle 1, Day 1 do not need to be repeated and can be utilised for Cycle 1, Day 1/randomisation. Laboratory safety assessments, the collection of blood samples for biomarker analysis (ctDNA sample, circulating soluble factors sample, whole blood DNA sample and whole blood RNA sample) and rich PK sampling should be performed on the day of the scheduled study visit. However, from Cycle 2 onwards, the laboratory safety assessments and biomarker sampling may be performed 1 day before the scheduled study visit. These samples must be collected pre-dosing.

[d] The handheld device must be charged and fully functional at the beginning of the baseline visit to ensure that the PROs can be completed at the start of the visit.

[e] The patient should be trained on the use of the device and the importance of completing the PRO questionnaires in accordance with the schedule throughout the study.

[f] Routine safety measurements and routine clinical procedures should be performed before dosing of study treatment unless otherwise specified.

[g] Bidimensional ECHO is the preferred modality because of the global technetium [Tc-99m] shortage (but MUGA can be used alternatively). The modality of the cardiac function assessments must be consistent within patient ie, if ECHO is used for the screening assessment and a follow-up assessment if clinically indicated, then ECHO should also be used for subsequent scans if required. Patients should also be examined using the same machine and operator whenever possible.

[h] Female pre- and peri-menopausal patients starting an LHRH agonist (either starting or continuing this treatment) must have oestradiol and FSH measurements done at screening (before Cycle 1, Day 1) and after at least 4 weeks after commencing LHRH agonist treatment to confirm post-menopausal levels; assessments after screening will be at Day 1 of each cycle and when clinically indicated. Female patients <60 years who are considered post-menopausal per wording of inclusion criterion 5, must have oestradiol and FSH levels confirmed as being within the standard laboratory reference range for post-menopausal females at screening (before Cycle 1, Day 1) only and if clinically indicated (see Section 8.2.1 for all details when and for whom FSH and oestradiol must be checked).

[i] For patients taking concomitant metformin, please refer to Sections 8.2.1.2 and 8.4.5.3.

[j] 'Fasting' is defined as no caloric intake for ≥4 hours before sampling. Glucose samples should be done under fasting conditions with the exception of the 4-hour post dose sample on Cycle 1, Week 1, Day 1 which can be fasting or non-fasting.

[k] *FFPE tumour sample*: Tumour tissue will be required at baseline for determination of *PIK3CA/AKT1/PTEN* status at a central laboratory to allow monitoring of the prevalence of the *PIK3CA/AKT1/PTEN*-altered subgroup. Tumour tissue samples will be collected as detailed in the Laboratory Manual and Diagnostic Testing Manual. The most recently collected tumour tissue, from primary or recurrent cancer is required. FFPE blocks are strongly preferred. If not possible, preferably 30 (minimum 20) freshly-cut unstained serial tumour tissue sections are accepted provided they met the specifications described in the Diagnostic Testing Manual. Local pathology QC must be completed prior to randomisation to ensure the sample is suitable for NGS analysis, based on the requirements described in the Diagnostic Testing Manual.

[l] These samples will not be collected in China. Results can be provided to the investigator upon request, if available.

[m] *Paired biopsies (optional)*: Baseline sample: at screening OR pre-dose on Cycle 1, Week 1, Day 1. On-treatment sample: at Cycle 1, Week 3, Day 4 (any day between Cycle 1, Week 3, Day 2 and Cycle 1, Week 3, Day 4 is allowed) >4 hours post-dose.

[n] *Circulating soluble factor, whole blood DNA sample, and whole blood RNA sample draws*: To be taken at screening, pre-dose on Cycle 1, Week 1, Day 1; Cycle 1, Week 3, Day 1; Cycle 2, Week 1, Day 1; Cycle 3, Week 1, Day 1, at progression, and at discontinuation of IMP if this falls on a different visit/time than progression.

[o] Baseline RECIST v1.1 assessments will be performed using CT scans of the chest, abdomen and pelvis (or MRI where CT is contraindicated) and should be performed as close as possible to the start of treatment. RECIST v1.1 follow-up assessments will include CT scans of thorax, abdomen and pelvis (or MRI where CT is contraindicated) for all patients. Any other sites at which new disease is suspected should also be appropriately imaged.

[p] In addition to regular contacts at ≤8-week intervals, patients will be contacted in the 7 days following a specified date (data cut-off date) for survival analysis.

[q] Patients will enter the PFS2 follow-up period once the patient has discontinued study treatment due to progressive disease by RECIST v1.1. Progression on second-line treatment will be documented by site personnel at the 30-day follow-up visit, every 8 weeks (±7 days) for the first 2 years and every 12 weeks (±7 days) thereafter until second progression. Survival status and subsequent cancer therapies will be documented by site personnel, following objective disease progression or treatment discontinuation, at the 30-day follow-up, every 8 weeks (±7 days) for the first 2 years and every 12 weeks (±7 days) thereafter until end of study, study withdrawal or death.

[r] Assessments include: number of hospitalisations and attendances; primary symptom/reason associated with hospitalisation or attendance; length of stay, including time in intensive care; and concomitant medication and procedures undertaken.

[s] Randomisation must occur within 28 days of the start of screening. Randomisation and Cycle 1, Week 1, Day 1 should ideally occur on the same day.

[t] Day 1 of each cycle is defined by fulvestrant dosing and fulvestrant should not be delayed due to capivasertib/placebo toxicity. In the case of a capivasertib/placebo dosing delay at the beginning of the cycle, the same routine safety measurements and routine clinical procedures must be repeated on the next planned date of dosing. Cycles are 28-day long, dosing outside of the prespecified window will be considered as overdose except for a dose delay situation.

[u] Administration of LHRH agonist in pre- or peri-menopausal women must start prior to or on Cycle 1, Day 1.

[v] If both drugs are discontinued at the same time as progression, visits are combined and the optional biopsy is completed, if applicable.

→, onwards*; BRCA1/2*, breast cancer gene 1/2; CT, computed tomography; ctDNA, circulating tumour DNA; DNA, deoxyribonucleic acid; ECG, electrocardiogram; ECHO, echocardiography; ECOG, Eastern Cooperative Oncology Group; EORTC, European Organisation for Research and Treatment of Cancer; EORTC QLQ-BR23, EORTC Quality of Life Questionnaire breast cancer specific module; EORTC QLQ-C30, EORTC Quality of Life Questionnaire – Core 30 items; ePRO, electronic patient-reported outcome; EQ-5D-5L, European Quality of Life 5-Domain 5-Level Scale; FFPE, formalin-fixed paraffin-embedded; FSH, follicle stimulating hormone; HOSPAD, Hospital Admission module; LHRH, luteinising-hormone releasing hormone; LVEF, left ventricular ejection fraction; MRI, magnetic resonance imaging; MUGA, multiple-gated acquisition scan; PFS2, time from randomisation to second progression or death; PGIC, Patient Global Impression–Change; PGIS, Patient Global Impression–Severity; PGI-TT, Patient Global Impression–Treatment Tolerability; PK, pharmacokinetics; *PIK3CA*, phosphatidylinositol-4,5-bisphosphate 3-kinase catalytic subunit alpha gene; PRO, patient-reported outcome; PRO-CTCAE, Patient-Reported Outcomes version of the Common Terminology Criteria for Adverse Events; PTEN, phosphatase and tensin homolog; RECIST v1.1, Response Evaluation Criteria in Solid Tumours version 1.1; RNA, ribonucleic acid; WHO, World Health Organisation.

## 1.2 Synopsis

**International co-ordinating investigator**

Professor Nicholas Turner, MA FRCP PhD
Breast Unit, The Royal Marsden NHS Foundation Trust
Breast Cancer Now Research Centre, The Institute of Cancer Research
London, SW3 6JJ
United Kingdom

**Protocol Title:** A Phase III Double-blind Randomised Study Assessing the Efficacy and Safety of Capivasertib + Fulvestrant Versus Placebo + Fulvestrant as Treatment for Locally Advanced (Inoperable) or Metastatic Hormone Receptor Positive, Human Epidermal Growth Factor Receptor 2 Negative (HR+/HER2−) Breast Cancer Following Recurrence or Progression On or After Treatment with an Aromatase Inhibitor (CAPItello-291)

**Short Title:** Phase III Study of Capivasertib + Fulvestrant vs Placebo + Fulvestrant as Treatment for Locally Advanced (Inoperable) or Metastatic HR+/HER2− Breast Cancer (CAPItello-291)

**Rationale:**

Numerous preclinical studies have shown that endogenous AKT activity promotes breast cancer cell survival and resistance to chemotherapy or endocrine therapy (Frogne et al 2005, Ghayad et al 2010, van der Hage et al 2004). This can be overcome by down-regulation or inhibition of the PI3K/AKT pathway, as has been shown in several in vitro and in vivo breast cancer models (Frogne et al 2005, Ghayad et al 2010, van der Hage et al 2004). In addition, long-term estrogen deprivation (LTED) of human breast cancer cells in vitro (to mimic the low estrogen levels observed in aromatase inhibitor [AI] treated patients) has revealed hyperactivation of the PI3K/mTOR signalling pathway (Miller et al 2010). Consistently, increased PI3K/AKT signalling in clinical tumour samples is associated with poor anti-proliferative response to AI in breast cancer patients (Tokunaga et al 2006, Gao et al 2014). Inhibition of the PI3K pathway in the LTED cells induced apoptosis, indicating dependence on the PI3K pathway upon hormone deprivation (Miller et al 2010). Reciprocal feedback between the estrogen receptor (ER) and PI3K signalling suggests that the combination of endocrine therapy and inhibitors of the PI3K/AKT pathway may have clinical utility in the treatment of HR+ breast cancer, with responses expected both in patients with tumours harbouring a genetic alteration leading to an activated PI3K pathway as well as in patients whose tumours do not harbour such genetic alterations, but become dependent on the PI3K pathway upon treatment with ER downregulating/degrading agents, eg, AIs or selective ER degraders (SERDs).

A recent investigator-sponsored, randomised, double-blind, placebo-controlled Phase Ib/II study (NCT01992952, FAKTION) evaluated the combination of capivasertib (an AKT

inhibitor) + fulvestrant (a selective estrogen down-regulator) compared with placebo + fulvestrant in post-menopausal patients with estrogen receptor positive, human epidermal growth factor receptor 2 negative (ER+/HER2−) advanced breast cancer (ABC) (140 patients, randomised 1:1) after relapse or disease progression on an AI therapy (Jones et al 2019). The study met its primary endpoint of progression-free survival (PFS), showing a statistically significant improvement in PFS with the addition of capivasertib to fulvestrant. At the time of the PFS analysis, overall survival (OS) data were not mature; however, the data suggest an improvement with the addition of capivasertib to fulvestrant. The combination of capivasertib + fulvestrant was generally well tolerated, with a manageable safety profile.

This Phase III study (CAPItello-291) is being conducted in response to the positive results from the FAKTION study and aims to evaluate the efficacy and safety of capivasertib + fulvestrant versus (vs) placebo + fulvestrant in patients with locally advanced (inoperable) or metastatic hormone receptor positive (HR+)/HER2− breast cancer following recurrence or progression on or after third generation AI therapy in an unselected population (hereafter referred to as the "overall population") and a molecularly defined subgroup with tumours harbouring at least one *PIK3CA/AKT1/PTEN*-qualifying alteration detected in tissue (hereafter referred to as the "altered subgroup").

## Objectives and Endpoints:

| Primary objective | Endpoint |
|---|---|
| To compare the effect of capivasertib + fulvestrant relative to placebo + fulvestrant by assessment of PFS in the overall population and in the *PIK3CA/AKT1/PTEN*-altered subgroup. | PFS is defined as the time from randomisation until progression per RECIST v1.1, as assessed by the investigator at the local site, or death due to any cause. |

PFS, progression-free survival; RECIST v1.1, Response Evaluation Criteria in Solid Tumours version 1.1; vs, versus.

| Secondary objectives | Endpoint |
|---|---|
| To compare the effect of capivasertib + fulvestrant relative to placebo + fulvestrant by assessment of OS in the overall population and in the *PIK3CA/AKT1/PTEN*-altered subgroup. | OS is length of time from randomisation until the date of death due to any cause. |
| To compare the effect of capivasertib + fulvestrant relative to placebo + fulvestrant by assessment of PFS2 in the overall population and in the *PIK3CA/AKT1/PTEN*-altered subgroup. | PFS2 is defined as the time from randomisation until second progression on next-line treatment, as assessed by the investigator at the local site, or death due to any cause. |
| To compare the effect of capivasertib + fulvestrant relative to placebo + fulvestrant by assessment of ORR in the overall population and in the *PIK3CA/AKT/PTEN*-altered subgroup. | ORR is defined as the percentage of patients with at least one CR or PR per RECIST v1.1, as assessed by the investigator at the local site. |

| Secondary objectives | Endpoint |
|---|---|
| To compare the effect of capivasertib + fulvestrant relative to placebo + fulvestrant by assessment of DoR in the overall population and in the *PIK3CA/AKT/PTEN*-altered subgroup. | DoR is defined as the time from the date of first documented response until date of documented progression or death in the absence of disease progression. |
| To compare the effect of capivasertib + fulvestrant relative to placebo + fulvestrant by assessment of CBR in the overall population and in the *PIK3CA/AKT/PTEN*-altered subgroup. | CBR is defined as the percentage of patients who have a CR, PR or stable disease per RECIST v1.1 (without subsequent cancer therapy) maintained ≥24 weeks after randomisation. |
| To assess the safety and tolerability of capivasertib + fulvestrant as compared to placebo + fulvestrant in the overall population and in the *PIK3CA/AKT/PTEN*-altered subgroup. | Safety and tolerability will be evaluated in terms of AEs/SAEs, vital signs, clinical chemistry/haematology/glucose metabolism parameters, and ECG parameters.<br>Assessments related to AEs cover:<br>• Occurrence/frequency<br>• Relationship to capivasertib and fulvestrant as assessed by investigator<br>• CTCAE grade<br>• Seriousness<br>• Death<br>• AEs leading to discontinuation of capivasertib/placebo<br>• AEs leading to discontinuation of fulvestrant<br>• AEs leading to dose interruption of capivasertib/placebo<br>• AEs leading to dose interruption of fulvestrant<br>• AEs leading to dose reduction of capivasertib/placebo<br>• AEs of special interest<br>• Other significant AEs<br>Vital signs parameters include systolic and diastolic blood pressure, pulse, respiratory rate, body temperature and weight.<br>Assessments cover:<br>• Observed value<br>• Absolute and change from baseline values over time |

| Secondary objectives | Endpoint |
|---|---|
| To evaluate the PK of capivasertib when given in combination with fulvestrant. | Plasma concentration of capivasertib pre-dose ($C_{trough}$) and post-dose ($C_{1h}$ and $C_{4h}$) in the overall population (patients randomised to capivasertib + fulvestrant).<br><br>$AUC_{0-12h}$, $C_{max}$ and $t_{max}$ in a subpopulation of approximately 6 Japanese patients with rich PK sampling. |
| To assess the impact of capivasertib + fulvestrant vs placebo + fulvestrant on patients' disease-related symptoms, function and HRQoL in the overall population and in the *PIK3CA/AKT/PTEN*-altered subgroup where applicable. | Evaluation of EORTC QLQ-C30, EORTC QLQ-BR23, scale/item scores including change from baseline and time to deterioration. |
| To compare the effect of capivasertib + fulvestrant relative to placebo + fulvestrant by assessment of time to definitive deterioration of ECOG performance status from baseline in the overall population and in the *PIK3CA/AKT/PTEN*-altered subgroup. | Time to definitive deterioration of ECOG performance status is defined as time from randomisation to the earlier of the date of the first definitive deterioration or death due to any cause. |

AE, adverse event; $AUC_{0-12h}$, area under the plasma concentration-time curve from zero to 12 hours; C, concentration; CBR, clinical benefit rate; $C_{max}$, maximum observed plasma (peak) concentration; CR, complete response; CTCAE, Common Terminology Criteria for Adverse Event; ctDNA, circulating tumour DNA; DoR, duration of response; ECG, electrocardiogram; ECOG, Eastern Cooperative Oncology Group; EORTC, European Organisation for Research and Treatment of Cancer; EORTC QLQ-BR23, EORTC Quality of Life Questionnaire breast cancer specific module; EORTC QLQ-C30, EORTC Quality of Life Questionnaire-Core 30 items; HRQoL health-related quality of life; ORR, objective response rate; OS, overall survival; PFS, progression-free survival; PFS2, time from randomisation to second progression or death; *PIK3CA*, phosphatidylinositol-4,5-bisphosphate 3-kinase catalytic subunit alpha gene; PK, pharmacokinetics; PR, partial response; PTEN, phosphatase and tensin homolog; RECIST v1.1, Response Evaluation Criteria in Solid Tumours version 1.1; SAE, serious adverse event; $t_{max}$, time to reach peak or maximum observed concentration following drug administration; vs, versus.

| **Exploratory objectives**[a] | **Endpoint** |
|---|---|
| To compare the effect of capivasertib + fulvestrant relative to placebo + fulvestrant on OS, PFS and ORR in additional populations which may include, but are not limited to:<br>• Patients with *PIK3CA/AKT1/PTEN*-qualifying alterations detected in tissue and/or ctDNA<br>• Patients without *PIK3CA/AKT1/PTEN*-qualifying alterations in tissue (non-altered subgroup)<br>• Patients with *PIK3CA/AKT1/PTEN*-qualifying alterations detected in ctDNA only<br>• Patients with *PIK3CA*-qualifying alterations detected in tissue<br>• Patients with PTEN low/absent as determined by IHC<br>• Patients with or without *BRCA1/2* mutations as determined by local testing and/or central NGS | OS is length of time from randomisation until the date of death due to any cause.<br>PFS is defined as the time from randomisation until progression per to RECIST v1.1, as assessed by the investigator at the local site, or death due to any cause.<br>ORR is defined as the percentage of patients with at least one CR or PR per RECIST v1.1, as assessed by the investigator at the local site. |
| To compare the effect of capivasertib + fulvestrant relative to placebo + fulvestrant by assessment of time to first subsequent chemotherapy or death (TFSC). | TFSC is defined as time from randomisation to the earlier of start date of subsequent chemotherapy after discontinuation of randomised treatment or death due to any cause. |
| To explore exposure-response relationships for efficacy and safety/tolerability variables. | Data-driven model parameters for the relationship between capivasertib exposure and efficacy and safety/tolerability variables, as applicable. |
| To compare healthcare resource use associated with capivasertib + fulvestrant vs placebo + fulvestrant. | Healthcare Resource Use Module. |
| To generate ctDNA data for use in regulatory submission with view to approval or IVD tests. | Evaluation of pre-treatment plasma samples for ctDNA analysis. |
| To investigate the effect of capivasertib + fulvestrant vs placebo + fulvestrant on genetic alterations including but not limited to *PIK3CA/AKT1/PTEN* alterations that may be detected in ctDNA. | Evaluation of pre- and on-treatment plasma samples for genetic alterations in ctDNA. |
| To investigate predictive markers of response and acquired resistance to capivasertib + fulvestrant vs placebo + fulvestrant including but not limited to biomarkers of AKT inhibitor sensitivity/resistance, immunological biomarkers, changes in gene expression and breast cancer subtyping. | Analysis of ctDNA and/or tumour tissue. |

| Exploratory objectives[a] | Endpoint |
|---|---|
| To investigate the effect of capivasertib + fulvestrant or placebo + fulvestrant on biomarkers that may be observed in paired pre- and on-treatment tumour tissue samples. | Analysis of matched pre- and post-treatment biopsy samples. |
| To assess patient-reported treatment tolerability of capivasertib + fulvestrant vs placebo + fulvestrant. | Patient-reported outcomes version of the CTCAE (PRO-CTCAE) and a single item on overall treatment tolerability using PGI-TT. |
| To assess patient-reported overall change in health status since the start of the study treatment and current severity of cancer symptoms in capivasertib + fulvestrant vs placebo + fulvestrant. | Evaluation of patient global impression of severity of cancer symptoms and change in health status using PGIS and PGIC, respectively. |
| To explore the impact of treatment and disease state on health state utility. | Health state utility data for economic evaluation will be derived by using the EQ-5D-5L health state utility index. |
| To perform future exploratory research into genes/genetic variation that may influence response (ie, distribution, safety, tolerability and efficacy) to capivasertib + fulvestrant vs placebo + fulvestrant and/or susceptibility to cancer. | Collection and storage of DNA for future analysis. |
| To conduct potential future exploratory research into factors that may influence the progression of cancer and/or response (efficacy, tolerability or safety) to capivasertib + fulvestrant vs placebo + fulvestrant. | Collection and storage of plasma, blood and tissue and/or analysis of surplus plasma, blood or tissue including patient-specific archival tumour tissue, if available. This may include but is not limited to the analysis of tumour and circulating biomarkers, such as DNA, RNA, proteins or metabolites. |

[a] China will be excluded from exploratory objectives requiring the provision of additional tumour or blood samples.

*BRCA1/2*, breast cancer gene 1/2; ctDNA, circulating tumour DNA; DNA, deoxyribonucleic acid; EQ-5D-5L, European Quality of Life 5-Domain 5-Level Scale; IHC, immunohistochemistry; IVD, in vitro diagnostic; NGS, next-generation sequencing; OS, overall survival; PFS, progression-free survival; PGIC, Patient Global Impression–Change; PGIS, Patient Global Impression–Severity; PGI-TT, Patient Global Impression–Treatment Tolerability; PRO-CTCAE, Patient-Reported Outcomes version of the Common Terminology Criteria for Adverse Events; RECIST v1.1, Response Evaluation Criteria in Solid Tumours version 1.1; RNA, ribonucleic acid; TFSC, time to first subsequent chemotherapy or death; vs, versus.

**Overall design:**

This is a Phase III, double-blind, randomised study assessing the efficacy of capivasertib + fulvestrant vs placebo + fulvestrant for the treatment of patients with locally advanced (inoperable) or metastatic HR+/HER2− breast cancer following recurrence or progression on or after AI therapy.

Enrolment will be open to all eligible patients irrespective of the *PIK3CA/AKT1/PTEN* status of their tumour(s); however, adequate tumour tissue collected before study entry will be required for central retrospective analysis to monitor the prevalence of *PIK3CA/AKT1/PTEN* mutation status. The list of eligible alterations is curated on the basis of the currently published literature for causal associations between pathogenicity and the alterations, using the AstraZeneca strategy for early-stage clinical studies as described by Carr et al 2016. The list of eligible variants will be defined and specified in the statistical analysis plan (SAP) prior to analysis.

The target patient population is adult males and pre-menopausal or post-menopausal adult females (aged ≥18 years [≥20 years in Japan]) with histologically confirmed, locally advanced (inoperable) or metastatic, HR+/HER2− ABC with recurrence or progression while receiving or within 12 months of the end of (neo)adjuvant treatment with an AI, or progression while on prior AI for locally advanced or metastatic breast cancer (although this does not need to be the most recent therapy). In accordance with these criteria, the study intends to recruit patients with AI resistance.

HR+/HER2− breast cancer must be histologically determined from the most recent tumour sample (primary or metastatic), as per the American Society of Clinical Oncology-College of American Pathologists guideline recommendations (Hammond et al 2010, Wolff et al 2018). To fulfil the requirement of HR+ disease, a breast cancer must express ER with or without co-expression of progesterone receptor (Hammond et al 2010, Wolff et al 2018).

Patients must be candidates to receive further endocrine-based therapy (ie, fulvestrant monotherapy). Eligible patients may have received up to 2 prior endocrine therapy lines for metastatic disease and up to 1 line of chemotherapy in the metastatic setting. A minimum of 51% of patients recruited to the study should have been previously treated with a CDK4/6 inhibitor.

> **NOTE:** CDK4/6 inhibitors (palbociclib, ribociclib, abemaciclib) are, in certain markets, approved treatment options in combination with an aromatase inhibitor or fulvestrant (± luteinising-hormone releasing hormone (LHRH) agonist depending on menopausal status) for patients with advanced or metastatic HR+HER2- breast cancer who have received prior endocrine therapy and/or as initial endocrine-based therapy. Some of these agents have been shown to improve overall survival in this patient population and, where

available in the country in which a patient is being enrolled, must be considered and excluded as treatment options before the patient can be considered for entering this study. Prior CDK4/6 inhibition will be a stratification factor; the actual proportions will be monitored during the course of the study (Section 6.3). Reasons why a patient has not received a CDK4/6 inhibitor will be collected.

Patients with prior exposure to fulvestrant, other SERDs or PI3K/AKT/mTOR inhibitors are not permitted to enter the study. Concomitant treatment with a CDK4/6 inhibitor is not permitted.

Pre-menopausal patients with adequate ovarian suppression are eligible for the study. Pre-menopausal patients without ovarian suppression could be eligible if concomitant treatment with a LHRH agonist is started prior to or on Cycle 1, Day 1.

Patients must have radiological evidence of recurrence or progression, an Eastern Cooperative Oncology Group (ECOG) Performance Status of 0 or 1, and measurable lesion(s) according to RECIST v1.1 or, in the absence of measurable disease, lytic or mixed bone lesions that can be assessed by computerised tomography (CT) or magnetic resonance imaging (MRI).

Patients with visceral metastases may be eligible unless the visceral involvement is associated with organ dysfunction (ie, visceral crisis) in which case, such patients are excluded.

A recently collected formalin-fixed paraffin embedded (FFPE) tumour tissue block (strongly preferred) or 30 (minimum 20) freshly-cut unstained, serial slides from the most recently collected tumour tissue (primary or recurrent cancer) will be required from all patients. If tissue is inadequate for testing, the study site may collect a fresh sample before the start of treatment.

Adequate haematology, glucose metabolism, renal and liver function tests are required at screening; patients with type 2 diabetes are eligible if HbA1c at screening is less than 8.0% (63.9 mmol/mol), and if they do not require insulin treatment. Patients with type 1 diabetes are not eligible.

Approximately 700 patients will be randomised (1:1) with approximately 350 included in each arm. Patients will receive capivasertib (400 mg or placebo, oral, twice daily; 4 days on and 3 days off, weekly) and fulvestrant (500 mg, intramuscular injection on Day 1 of Weeks 1 and 3 of Cycle 1, and then on Day 1, Week 1 of each cycle thereafter). Study treatment will continue until objective radiological disease progression as defined by RECIST v1.1.

Randomisation will occur as soon as possible after commencement of screening and should be within 28 days of screening. The randomisation scheme will be stratified on the following factors:

- Liver metastases (yes vs no)
- Prior use of CDK4/6 inhibitors (yes vs no)
- Geographic location:
  - Region 1: United States, Canada, Western Europe, Australia, and Israel
  - Region 2: Latin America, Eastern Europe and Russia
  - Region 3: Asia

The study schema is depicted in Figure 1.

**Study Period:**

Estimated date of first patient enrolled Q1 2020

Estimated date of last patient completed Q2 2024

**Number of Patients:**

It is expected that an estimated 930 patients will be screened and approximately 700 patients will be randomised, with half of the patients included in each treatment arm. The China cohort will consist of approximately 134 randomised patients from National Medical Product Administration (NMPA)-certified sites. In the event that recruitment of China cohort is not completed during global recruitment of approximately 700 patients, the recruitment of China cohort will continue until approximately 134 patients have been randomised from NMPA-certified sites.

**Treatments and treatment duration:**

Capivasertib/placebo will be administered orally twice daily on an intermittent dosing schedule with treatment on Days 1 to 4 each week of a 28-day treatment cycle. The starting dose is 400 mg twice daily (BD) (4 days on and 3 days off). Capivasertib/placebo dose reductions are permitted: the initial dose reduction can be to 320 mg BD and the second dose reduction can be to 200 mg BD.

Fulvestrant will be administered as 2 × 5 mL intramuscular injections (250 mg/5 mL = 500 mg in total) on Day 1 of Weeks 1 and 3 of Cycle 1, and then on Day 1, Week 1 of each cycle thereafter. Dose reductions for fulvestrant are not allowed.

Study treatment will be continued until disease progression unless there is evidence of unacceptable toxicity, or if the patient requests to stop the study treatment.

If capivasertib/placebo is discontinued for reasons other than disease progression, the patient may continue on fulvestrant alone at the investigator's discretion. Likewise, if fulvestrant is discontinued for reasons other than disease progression, the patient may continue capivasertib/placebo alone at the investigator's discretion.

Cross-over from placebo to capivasertib is not allowed.

**Data Monitoring Committee:**

An Independent Data Monitoring Committee (IDMC) will be established in order to assess the progress of the clinical study at regular intervals prior to the PFS primary analysis database lock. This will include reviewing unblinded safety and tolerability data.

**Statistical methods**

The Full Analysis Set (FAS) will be used as the primary population for reporting efficacy data and to summarise baseline characteristics. This comprises all patients randomised into the study, excluding patients randomised in China after the global cohort last patient first visit (LPFV), and will be analysed according to randomised treatment regardless of the treatment received (intention-to-treat [ITT] principle). Any important deviations from randomised treatment will be listed and considered when interpreting the efficacy data. The Safety Analysis Set will be used as the primary population for reporting safety data. This comprises all patients included in the FAS, who received at least 1 dose of study drug (fulvestrant, capivasertib, placebo) and will be analysed according to the treatment received. If a patient receives at least 1 dose of capivasertib they will be summarised in the capivasertib arm for safety summaries.

The primary objective of this study is to assess the efficacy of capivasertib +fulvestrant vs placebo + fulvestrant by assessment of PFS; the dual primary endpoints are PFS in the overall population and PFS in the *PIK3CA/AKT1/PTEN*-altered population. This will be analysed using a stratified log-rank test adjusting for the stratification factors. From the stratified Cox proportional hazards model (ties = Efron), the hazard ratio (HR) (capivasertib + fulvestrant vs placebo + fulvestrant) together with its corresponding 95% CI (calculated using a profile likelihood approach) will be presented. An HR <1 will favour capivasertib + fulvestrant.

The key secondary endpoints are OS and objective response rate (ORR) in the overall and the *PIK3CA/AKT1/PTEN*-altered populations. OS will be assessed as described above for the primary endpoint. ORR will be presented with two-sided 95% CIs using the Clopper-Pearson method (Clopper and Pearson 1934).

To preserve the overall type 1 error (familywise error rate) in the strong sense, a multiple testing procedure (MTP) including the primary and secondary endpoints will be implemented. The PFS Analysis (primary) will take place after PFS reaches approximately 77% maturity in both the overall and the *PIK3CA/AKT1/PTEN*-altered populations. The OS Interim Analysis is expected to occur when approximately 394 OS events have been observed in the overall population (80% information fraction) and the *PIK3CA/AKT1/PTEN*-altered population has reached similar maturity. The OS Final Analysis will take place when the approximately 70%

maturity has been observed in both the overall and the *PIK3CA/AKT1/PTEN*-altered populations.

Safety and tolerability data will be summarised using appropriate descriptive statistics.

## 1.3 Schema

The general study design is summarised in Figure 1.

**Figure 1 Study design**

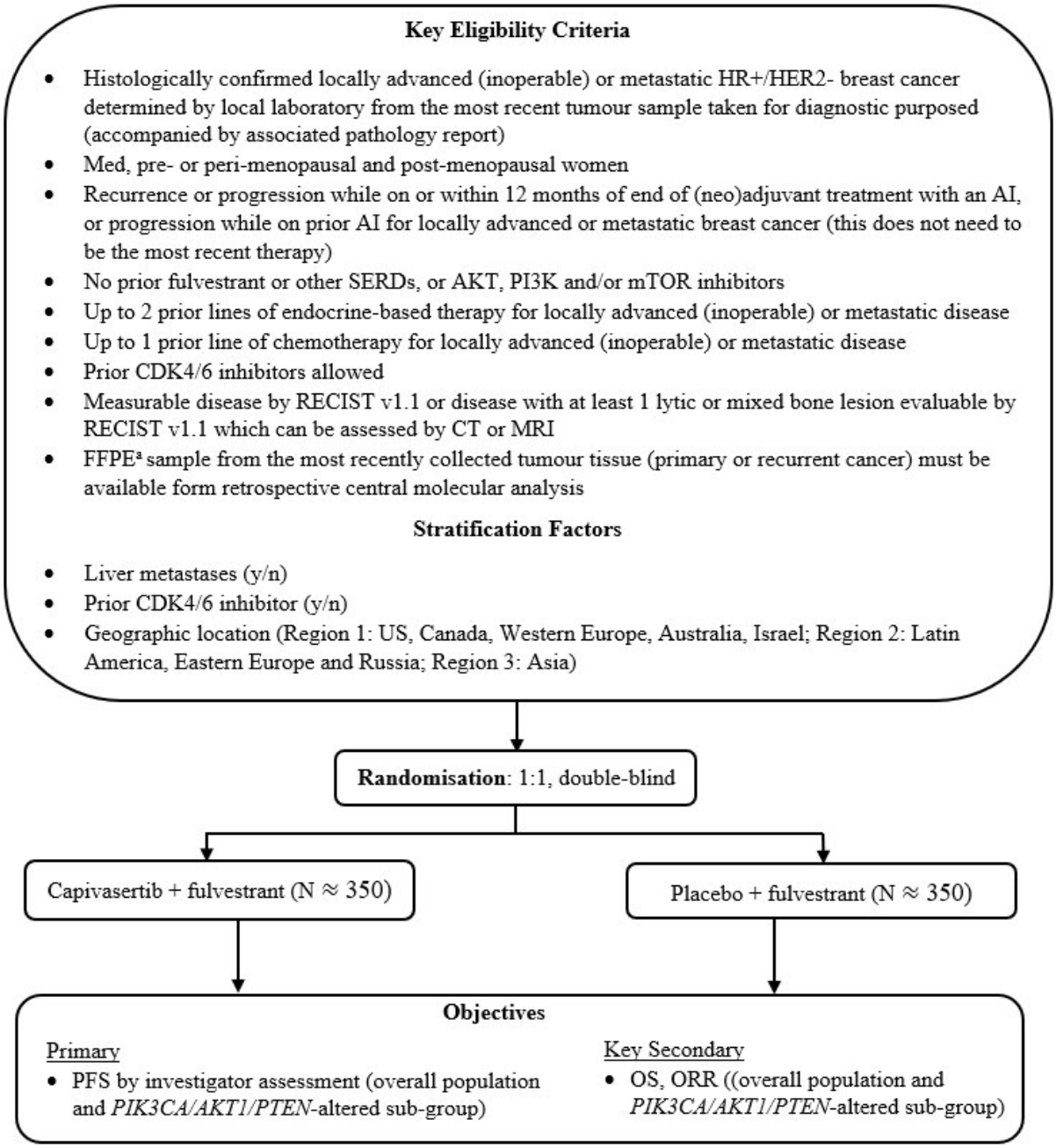


[a] FFPE (formalin-fixed, paraffin-embedded) blocks are strongly preferred, or if not possible, preferably 30 [minimal 20]) freshly-cut unstained serial tumour tissue sections are acceptable provided that they meet the specifications described in the Diagnostic Testing Manual.

AI, aromatase inhibitor; AKT, serine/threonine specific protein kinase; CBR, clinical benefit rate; CDK, cyclin dependent kinase; CT, computerised tomography; FFPE, formalin-fixed paraffin-embedded; HER2, human epidermal growth factor receptor 2; HR, Hormone receptor; MRI, magnetic resonance imaging; mTOR, mammalian target of rapamycin; ORR, objective response rate; OS, overall survival; PFS, progression-free survival; PI3K, phosphatidylinositol-3-kinase; RECIST v1.1, Response Evaluation Criteria in Solid Tumours version 1.1; SERD, selective estrogen response degrader; US, United States.

# 2 INTRODUCTION

Capivasertib, a novel pyrrolopyrimidine-derived compound, is a potent and selective oral, intermittently dosed inhibitor of all 3 isoforms of AKT (Davies et al 2012).

Further details can be found in the current capivasertib (AZD5363) Investigator's Brochure.

## 2.1 Study rationale

This Phase III study is designed to provide confirmatory evidence of the findings from the previous Phase Ib/II FAKTION study (NCT01992952), which evaluated the combination of capivasertib + fulvestrant compared with placebo + fulvestrant in post-menopausal patients with estrogen receptor positive, human epidermal growth factor receptor 2 negative (ER+/HER2−) advanced breast cancer (ABC) [140 patients, randomised 1:1] after relapse or disease progression on an aromatase inhibitor (AI) therapy (Jones et al 2019). The study met its primary endpoint of progression-free survival (PFS), showing a 5.5-month PFS improvement with the addition of capivasertib to fulvestrant, regardless of the PI3K/AKT/PTEN (phosphatidylinositol-3-kinase/AKT/phosphatase and tensin homolog) pathway activation status. At the time of the PFS analysis, overall survival (OS) data were not mature (37% maturity); however, the data suggest an improvement of approximately 6 months from the combination treatment (not statistically significant). The combination of capivasertib + fulvestrant was generally well tolerated, with a manageable safety profile. Further details on the FAKTION study are available in Section 2.2.

This Phase III study (CAPItello-291) aims to evaluate the efficacy and safety of capivasertib + fulvestrant versus (vs) placebo + fulvestrant in patients with locally advanced (inoperable) or metastatic hormone receptor positive (HR+)/HER2− breast cancer following recurrence or progression on or after third-generation AI therapy in an unselected population (hereafter referred to as the "overall population") and a molecularly defined subgroup with tumours harbouring at least one *PIK3CA/AKT1/PTEN*-qualifying alteration (hereafter referred to as the "altered subgroup"), as described in Section 4.1.

## 2.2 Background

In women, breast cancer is the most frequently diagnosed malignancy and the leading cause of cancer mortality worldwide (GLOBOCAN 2018, NCCN 2019). ABC comprises both locally advanced (inoperable) and metastatic disease. Although it can be treated, metastatic breast

cancer remains incurable with a median survival of approximately 3 years and a 5-year survival rate of around 25% (Cardoso et al 2018). Several prognostic indicators for breast cancer have been identified including HER2 and the ER (here, also referred to as hormone receptor [HR]). Most of the improvements in survival rates achieved in the last decade have been due to advances in the treatment of patients with HER2+ tumours. Conversely, survival rates for patients with HR+/HER2− tumours, which account for approximately 70% of all breast cancers (Howlader et al 2014), have not shown much improvement over the same period (Gobbini et al 2018).

In the majority of cases, endocrine-based therapy is the initial treatment for HR+/HER2− breast cancer. The exceptions are patients with visceral crisis or in whom endocrine-based alternatives have been exhausted or for whom there is evidence of endocrine resistance; in such instances, chemotherapy is required. In women, endocrine therapy is recommended for both post- and pre-menopausal patients where, in the latter setting, it is provided in conjunction with the suppression or ablation of ovarian function (Cardoso et al 2018). In men, the treatment approach for ABC is similar to that of women, with some particularities: tamoxifen is the preferred treatment in HR+ metastatic disease; in addition, AIs should be used in combination with luteinising hormone-releasing hormone (LHRH) agonist or surgical orchidectomy due to hypothalamic-pituitary negative feedback and in some case reports, fulvestrant has also shown efficacy for the treatment of metastatic disease (Cardoso et al 2017). Many patients with HR+/HER2− breast cancer benefit from sequential use of endocrine therapies and those who respond to endocrine therapy with either tumour shrinkage or long-term stabilisation (ie, clinical benefit) should be offered additional endocrine therapy at subsequent disease progression (NCCN 2019). The optimal sequence and integration of the available endocrine agents is not established and is influenced by choice of initial therapy, the response obtained, as well as individual patient and disease characteristics (Cardoso et al 2018). Several novel, effective agents have become available in recent years for combination with endocrine therapies. These include cyclin-dependent kinase (CDK) 4/6 inhibitors and everolimus in combination with exemestane (an inhibitor of the mammalian target of the rapamycin receptor [mTOR]). Available second or further lines of therapies are limited either with regard to the population able to gain benefit, or the minimal magnitude of benefit (Cardoso et al 2018). A Phase III study (BOLERO-2) of everolimus plus exemestane in patients with HR+/HER2- ABC post-AI progression showed that the combination therapy improved PFS compared with exemestane alone from 3.2 to 7.8 months irrespective of *PIK3CA* genotype, albeit without significant OS improvement (Yardley et al 2013). More recently, the Phase III SOLAR-1 study demonstrated an improvement in PFS of 5.7 to 11 months with the addition of the alpha-specific PI3K inhibitor alpelisib to fulvestrant in patients with HR+/HER2− ABC with tumours harbouring a *PIK3CA* mutation, who had relapsed or progressed on an AI (André et al 2019), based on which the FDA granted approval. Of note, a clinically relevant benefit was not seen in the *PIK3CA* non-mutant cohort and is not included in the approval. Despite these advances, these tumours eventually develop

endocrine resistance necessitating the use of chemotherapy and thus, HR+/HER2− ABC remains an area of considerable unmet medical need.

The AKT serine/threonine protein kinases (AKT1, AKT2, AKT3) are key downstream effectors of the PI3K/AKT/mTOR pathway, mediating cell proliferation and resistance to apoptosis, and are activated in a wide range of solid and haematologic malignancies (Brown and Banerji 2017, Lindsley 2010, Liu et al 2019). AKT activation in tumours is largely due to input from other signalling pathways upstream of AKT including loss of PTEN function and activating mutations in the catalytic subunit of PI3K (*PIK3CA*) (Yi and Lauring 2016). Numerous preclinical studies show that endogenous AKT activity promotes breast cancer cell survival and resistance to chemotherapy or endocrine therapy (Frogne et al 2005, Ghayad et al 2010, van der Hage et al 2004). This can be overcome by down-regulation or inhibition of the PI3K/AKT pathway, as has been shown in several in vitro and in vivo breast cancer models (Frogne et al 2005, Ghayad et al 2010, van der Hage et al 2004).

Capivasertib is under investigation by AstraZeneca for a range of therapeutic indications (Banerji et al 2018, Tamura et al 2016) including triple-negative breast cancer (TNBC), ER+/HER2− breast cancer and prostate cancer. Capivasertib inhibits the proliferation of 25 out of 180 tumour cell lines with a concentration causing 50% inhibition of cell growth ($GI_{50}$) of <1 μM. Breast cancer cell lines show the highest frequency of sensitivity to capivasertib, with a significant correlation between the presence of *PIK3CA* or *PTEN* mutations and sensitivity (Davies et al 2012). Capivasertib inhibits the growth of several human breast cancer xenograft models, including the TNBC xenograft model HCC1187 and the HER2+, *PIK3CA* mutant xenograft models BT474 and HCC1954.

Long-term estrogen deprivation (LTED) of human breast cancer cells in vitro (to mimic the low estrogen levels observed in AI-treated patients) revealed hyperactivation of the PI3K/mTOR signalling pathway (Miller et al 2010). Consistently, increased PI3K/AKT signalling in clinical tumour samples is associated with poor anti-proliferative response to AI in breast cancer patients (Tokunaga et al 2006, Gao et al 2014). Inhibition of the PI3K pathway in the LTED cells induced apoptosis, indicating dependence on the PI3K pathway upon hormone deprivation (Miller et al 2010). Furthermore, modest reduction of PTEN expression in cell lines and xenograft models led to endocrine resistance which could be overcome by treatment with capivasertib in combination with fulvestrant (Fu et al 2014). Capivasertib synergises with fulvestrant in models of breast cancer irrespective of their sensitivity to endocrine therapy; compared with either agent alone, the combined treatment with capivasertib and fulvestrant resulted in a greater anti-tumour response in human breast cancer HBCx22 OvaR xenografts growing in ovariectomised mice (Ribas et al 2015).

A recent investigator-sponsored Phase Ib/II randomised, double-blind, placebo-controlled study (FAKTION) evaluated the combination of capivasertib (400 mg BD, 4 days on 3 days

off) with fulvestrant compared with fulvestrant alone in post-menopausal women with locally advanced (inoperable) or metastatic breast cancer previously treated with a third-generation AI therapy (Jones et al 2019). Eligible patients were post-menopausal with locally advanced or metastatic ER+/HER2- breast cancer that was not amenable to surgical resection. Patients had to be suitable for endocrine treatment but were to have received no more than 3 previous lines of endocrine treatment for ABC and up to 1 line of chemotherapy for ABC. Patients were also to have experienced disease progression during treatment with a third-generation AI for metastatic disease (although this did not need to be the most recent therapy) or have relapsed on an AI in the adjuvant setting. Previous treatment with fulvestrant or PI3K/mTOR/AKT inhibitors was not allowed. PI3K/AKT/PTEN pathway activation status was determined prior to randomisation; the protocol-specified definition of activated PI3K/AKT/PTEN pathway was: an activating *PIK3CA* mutation detected in either tissue and/or circulating tumour DNA, or low/absent PTEN by immunohistochemistry (primary or metastatic tumour). Patients (N=140) were randomised 1:1; of these, 69 received capivasertib + fulvestrant and 71 received placebo + fulvestrant. Baseline patient and disease characteristics were largely balanced between treatment arms. The analysis at the time of the primary endpoint data cut-off (DCO) [after 112 events; 80% maturity] showed that the study met its primary endpoint (Jones et al 2019). In the intention-to-treat analysis, median PFS was 10.3 months in the capivasertib + fulvestrant group compared with 4.8 months for the placebo + fulvestrant group (hazard ratio [HR] = 0.58; 95% confidence interval [CI]: 0.39 to 0.84; two-sided p=0.004). The overall survival (OS) data were not mature (52 deaths reported; 37% maturity), but the addition of capivasertib to fulvestrant resulted in an observed OS improvement of approximately 6 months (26 vs 20 months, respectively), though this was not statistically significant (HR 0.59; 95% CI: 0.34 to 1.05; two-sided p=0.071). Of note, the PFS benefit appeared to be independent of the PI3K/AKT/PTEN pathway activation status based on the available data at the time of primary analysis: in patients with pathway activated tumours (N=59) the HR was 0.59 (95% CI 0.34 to 1.03; two-sided p=0.064) and in those with pathway non-active tumours (N=81) it was 0.56 (95% CI 0.33 to 0.96, two-sided p=0.035). In addition, and in keeping with other studies in the clinical development program, the combination of capivasertib + fulvestrant demonstrated an overall manageable safety profile with a low rate of discontinuation of capivasertib due to adverse events (AEs) (11.6% [8 from 69]). The most frequent grade ≥3 AEs that occurred in ≥10% of the population, irrespective of causality, in the capivasertib vs placebo treatment groups, respectively, were: diarrhoea (14.5% vs 4.2%), fatigue (1.4% vs 4.2%), rash (20.3% vs 0%), hyperglycaemia (4.3% vs 0%), vomiting (2.9% vs 0%), and infections (5.8% vs 2.8%). Two patients died during the study, 1 in the placebo arm (grade 5 haemorrhage) and 1 death without progression on treatment with capivasertib, possibly related to treatment (atypical pulmonary infection).

The combination of capivasertib (400 mg, 4 days on 3 days off) with fulvestrant in ER+ metastatic breast cancer is also being evaluated in the AstraZeneca-sponsored Study D3610C00001 (Parts E [*AKT1*-mutant, completed] and F [*PTEN*-mutant, ongoing] and

in 2 ongoing investigator-sponsored studies (plasmaMATCH- and Hyman AKT mutant), adopting the recommended combination dose regimen of capivasertib with fulvestrant established in the FAKTION study. Interim efficacy data from Study D3610C00001 Part E have shown clinical activity of capivasertib plus fulvestrant in heavily pre-treated patients with *AKT1*-mutant ER+ metastatic breast cancer, including those with prior resistance to fulvestrant, with an overall manageable safety profile (Smyth et al 2017). Pooled AE data from Study D3610C00001 Parts E and F (DCO of 4 October 2018) indicate that the most common CTCAE grade ≥3 events (>5% of patients) were diarrhoea (5.4%), hyperglycaemia (5.4%), and rash maculo-papular (10.8%). The discontinuation rate for toxicity was low: 7 (9.5%) and 3 (4.1%) patients had at least 1 AE that led to discontinuation of capivasertib treatment, irrespective of causality and causally-related, respectively. The only AE leading to discontinuation that affected more than 1 patient was fatigue (reported in 2 patients).

Taken together, the overall benefit/risk of capivasertib + fulvestrant appears to be favourable for patients with HR+/HER2− locally advanced/metastatic breast cancer but requires confirmation in this pivotal Phase III study.

A detailed description of the chemistry, pharmacology, efficacy, and safety of capivasertib is provided in the Investigator's Brochure.

## 2.3 Benefit/risk assessment

### 2.3.1 Risks with capivasertib

Based on non-clinical and clinical data available to date, the risks associated with capivasertib are as follows:

- Identified Risks: nausea and vomiting, stomatitis, dry skin, pruritis, decreased appetite
- Important Identified Risks: diarrhoea, rash, hyperglycaemia, hypersensitivity
- Important Potential Risk: QT prolongation

Based on non-clinical data available (dog telemetry study), QT prolongation has been noted as an important potential risk. However, in AstraZeneca-sponsored clinical studies of capivasertib to date, there were no reports of sudden death, torsades de pointes, or seizures, and from a recent concentration-QT model analysis, capivasertib is not predicted to pose a clinically significant safety risk for QT prolongation at the therapeutic dosing regimen. However, it is recommended to avoid drugs known to prolong QT interval (see Section 6.5.3). Further details on the risks are available in 'Section 6: Summary of data and guidance for the investigators' of the Investigator's Brochure.

Overall in the monotherapy intermittent dosage pool (N=229), 42.4% patients had serious adverse events (SAEs), 17% were causally related and 3.1% patients were reported with SAEs leading to death. There were no capivasertib treatment-related deaths in this pool. A total of

17.5% patients were discontinued due to SAEs, and 11.4% were discontinued due to SAEs related to capivasertib treatment.

Based on data from the D3610C00001 and FAKTION studies (Section 2.2), the combination of capivasertib with fulvestrant has a tolerability profile that is manageable with dose modifications and supportive care.

There are 16 externally sponsored research studies which are planned, have recently commenced recruitment or are ongoing. Of these 16, 10 are investigating capivasertib specifically and 6 are "umbrella/basket" studies in which capivasertib is an option in a panel of potential investigational drugs. AstraZeneca is not aware of any new and significant safety information from externally sponsored research.

### 2.3.2 Overall benefit-risk and ethical assessment

Although endocrine therapy administered with or without either a CDK4/6 inhibitor, everolimus or alpelisib (in *PIK3CA* mutant tumours) are effective options for at least some patients with HR+/HER2− ABC, all tumours eventually develop endocrine resistance, and from that point will require sequential chemotherapies given until disease progression or unacceptable toxicity, with the intent of reducing disease burden and symptoms, and delaying disease progression. Thus, HR+/HER2− ABC still represents an area of considerable unmet medical need for new therapies and/or combinations that can delay or overcome resistance.

Clinical and non-clinical data with capivasertib (see Section 2.2) support the hypothesis that AKT inhibition in combination with an endocrine therapy such as fulvestrant may be a valid treatment strategy for HR+/HER2− ABC. Capivasertib has also demonstrated clinical activity in several settings including patients with metastatic TNBC in combination with first-line paclitaxel (Schmid et al 2018), patients with advanced *AKT1* mutant solid tumours in monotherapy or combination with fulvestrant (ER+ breast cancer) (Hyman et al 2017, Smyth et al 2017) and patients with ER+/HER2- ABC in combination with fulvestrant after AI therapy (Jones et al 2019).

The non-clinical profile and emerging clinical safety profile from the early clinical studies with capivasertib have not identified risks that would preclude investigation in this setting. The study design of this randomised clinical trial aims to minimise potential risks in several ways. First, appropriate inclusion and exclusion criteria will be included in the study protocol to protect patient safety. For example, in order to be enrolled, patients must be eligible for fulvestrant treatment (as per local investigator assessment) which is administered in both arms of the study, and patients with symptomatic visceral disease or any disease burden that makes the patient ineligible for endocrine therapy per the investigator's best judgement will be excluded. Of note, patients with uncontrolled and/or insulin dependent diabetes are also excluded from the study. Second, the protocol includes safety monitoring in excess of

standard of care monitoring, with the intent of protecting patients involved in the study. Furthermore, a dose modification strategy for the management of toxicity and monitoring is in place for those risks deemed to be most likely or serious. Thus, based upon the clinical and non-clinical safety profile, the limited life expectancy of patients due to malignant disease, and the strength of the scientific hypothesis under evaluation, the benefit/risk assessment for this study supports the treatment of capivasertib and fulvestrant in patients with HR+/HER2− ABC.

More detailed information about the known and expected benefits and risks and reasonably expected AEs of capivasertib may be found in the Investigator's Brochure.

See Section 9.5.1 and Appendix C for information regarding the Data Monitoring Committee.

### 2.3.3 Impact on Benefit/Risk from Study Disruptions due to Coronavirus Disease 2019

The emergence of the novel coronavirus disease 2019 (SARS-CoV-2/COVID-19) pandemic presents a potential safety risk for patients and therefore several risk mitigation factors have been implemented in this study (see Section 4.5, Appendix K, and Appendix L).

# 3 OBJECTIVES AND ENDPOINTS

## 3.1 Primary objectives

The primary objectives for the study and associated outcome measures are summarised in Table 2.

**Table 2 Primary objectives**

| Primary objective | Endpoint |
|---|---|
| To compare the effect of capivasertib + fulvestrant relative to placebo + fulvestrant by assessment of PFS in the overall population and in the *PIK3CA/AKT/PTEN*-altered subgroup.. | PFS is defined as the time from randomisation until progression per RECIST v1.1, as assessed by the investigator at the local site, or death due to any cause. |

PFS, progression-free survival; RECIST v1.1, Response Evaluation Criteria in Solid Tumours version 1.1; vs, versus.

## 3.2 Secondary objectives

The secondary objectives of the study and associated outcome measures are summarised in Table 3.

**Table 3 Secondary objectives**

| Secondary objectives | Endpoint |
|---|---|
| To compare the effect of capivasertib + fulvestrant relative to placebo + fulvestrant by assessment of OS in the overall population and in the PIK3CA/AKT/PTEN-altered subgroup. | OS is length of time from randomisation until the date of death due to any cause. |
| To compare the effect of capivasertib + fulvestrant relative to placebo + fulvestrant by assessment of PFS2 in the overall population and in the *PIK3CA/AKT/PTEN*-altered subgroup. | PFS2 is defined as the time from randomisation until second progression on next-line treatment, as assessed by the investigator at the local site, or death due to any cause. |
| To compare the effect of capivasertib + fulvestrant relative to placebo + fulvestrant by assessment of ORR in the overall population and in the *PIK3CA/AKT/PTEN*-altered subgroup. | ORR is defined as the percentage of patients with at least one CR or PR per RECIST v1.1, as assessed by the investigator at the local site. |
| To compare the effect of capivasertib + fulvestrant relative to placebo + fulvestrant by assessment of DoR in the overall population and in the *PIK3CA/AKT/PTEN*-altered subgroup. | DoR is defined as the time from the date of first documented response until date of documented progression or death in the absence of disease progression. |
| To compare the effect of capivasertib + fulvestrant relative to placebo + fulvestrant by assessment of CBR in the overall population and in the *PIK3CA/AKT/PTEN*-altered subgroup. | CBR is defined as the percentage of patients who have a CR, PR or stable disease per RECIST v1.1 (without subsequent cancer therapy) maintained ≥24 weeks after randomisation. |
| To assess the safety and tolerability of capivasertib + fulvestrant as compared to placebo + fulvestrant in | Safety and tolerability will be evaluated in terms of AEs/SAEs, vital signs, clinical |

| Secondary objectives | Endpoint |
|---|---|
| the overall population and in the *PIK3CA/AKT/PTEN*-altered subgroup. | chemistry/haematology/glucose metabolism parameters, and ECG parameters.<br><br>Assessments related to AEs cover:<br>• Occurrence/frequency<br>• Relationship to capivasertib and fulvestrant as assessed by investigator<br>• CTCAE grade<br>• Seriousness<br>• Death<br>• AEs leading to discontinuation of capivasertib/placebo<br>• AEs leading to discontinuation of fulvestrant<br>• AEs leading to dose interruption of capivasertib/placebo<br>• AEs leading to dose interruption of fulvestrant<br>• AEs leading to dose reduction of capivasertib/placebo<br>• AEs of special interest<br>• Other significant AEs<br><br>Vital signs parameters include systolic and diastolic blood pressure, pulse, respiratory rate, body temperature and weight.<br><br>Assessments cover:<br>• Observed value<br>• Absolute and change from baseline values over time |
| To evaluate the PK of capivasertib when given in combination with fulvestrant. | Plasma concentration of capivasertib pre-dose ($C_{trough}$) and post-dose ($C_{1h}$ and $C_{4h}$) in the overall population (patients randomised to capivasertib + fulvestrant).<br><br>AUC $_{0\text{-}12h}$, $C_{max}$ and $t_{max}$ in a subpopulation of approximately 6 Japanese patients with rich PK sampling. |
| To assess the impact of capivasertib + fulvestrant vs placebo + fulvestrant on patients' disease-related symptoms, function and HRQoL in the overall population and in the *PIK3CA/AKT/PTEN*-altered subgroup where applicable. | Evaluation of EORTC QLQ-C30, EORTC QLQ-BR23, scale/item scores including change from baseline and time to deterioration. |

| Secondary objectives | Endpoint |
|---|---|
| To compare the effect of capivasertib + fulvestrant relative to placebo + fulvestrant by assessment of time to definitive deterioration of ECOG performance status from baseline in the overall population and in the *PIK3CA/AKT/PTEN*-altered subgroup. | Time to definitive deterioration of ECOG performance status is defined as time from randomisation to the earlier of the date of the first definitive deterioration or death due to any cause. |

AE, adverse event; AUC $_{0\text{-}12h}$, area under the plasma concentration-time curve from zero to 12 hours; C, concentration; CBR, clinical benefit rate; $C_{max}$, maximum observed plasma (peak) concentration; CR, complete response; CTCAE, Common Terminology Criteria for Adverse Event; ctDNA, circulating tumour DNA; DoR, duration of response; ECG, electrocardiogram; ECOG, Eastern Cooperative Oncology Group; EORTC, European Organisation for Research and Treatment of Cancer; EORTC QLQ-BR23, EORTC Quality of Life Questionnaire breast cancer specific module; EORTC QLQ-C30, EORTC Quality of Life Questionnaire-Core 30 items; HRQoL health-related quality of life; ORR, objective response rate; OS, overall survival; PFS, progression-free survival; PFS2, time from randomisation to second progression or death; *PIK3CA*, phosphatidylinositol-4,5-bisphosphate 3-kinase catalytic subunit alpha gene; PK, pharmacokinetics; PR, partial response; PTEN, phosphatase and tensin homolog; RECIST v1.1, Response Evaluation Criteria in Solid Tumours version 1.1; SAE, serious adverse event; $t_{max}$, time to reach peak or maximum observed concentration following drug administration; vs, versus.

## 3.3 Exploratory objectives

The exploratory objectives of the study and associated outcome measures are summarised in Table 4. These exploratory objective analyses may be reported separately from the clinical study report (CSR).

**Table 4 Exploratory objectives**

| Exploratory objectives[a] | Endpoint |
|---|---|
| To compare the effect of capivasertib + fulvestrant relative to placebo + fulvestrant on OS, PFS and ORR in additional populations which may include, but are not limited to:<br>• Patients with *PIK3CA/AKT1/PTEN*-qualifying alterations detected in tissue and/or ctDNA<br>• Patients without *PIK3CA/AKT1/PTEN*-qualifying alterations in tissue (non-altered subgroup)<br>• Patients with *PIK3CA/AKT1/PTEN*-qualifying alterations detected in ctDNA only<br>• Patients with *PIK3CA*-qualifying alterations detected in tissue<br>• Patients with PTEN low/absent as determined by IHC<br>• Patients with or without *BRCA1/2* mutations as determined by local testing and/or central NGS | OS is length of time from randomisation until the date of death due to any cause.<br>PFS is defined as the time from randomisation until progression per to RECIST v1.1, as assessed by the investigator at the local site, or death due to any cause.<br>ORR is defined as the percentage of patients with at least one CR or PR per RECIST v1.1, as assessed by the investigator at the local site. |

| **Exploratory objectives**[a] | **Endpoint** |
|---|---|
| To compare the effect of capivasertib + fulvestrant relative to placebo + fulvestrant by assessment of time to first subsequent chemotherapy or death (TFSC). | TFSC is defined as time from randomisation to the earlier of start date of subsequent chemotherapy after discontinuation of randomised treatment or death due to any cause. |
| To explore exposure-response relationships for efficacy and safety/tolerability variables. | Data-driven model parameters for the relationship between capivasertib exposure and efficacy and safety/tolerability variables, as applicable. |
| To compare healthcare resource use associated with capivasertib + fulvestrant vs placebo + fulvestrant. | Healthcare Resource Use Module. |
| To generate ctDNA data for use in regulatory submission with view to approval or IVD tests. | Evaluation of pre-treatment plasma samples for ctDNA analysis. |
| To investigate the effect of capivasertib + fulvestrant vs placebo + fulvestrant on genetic alterations including but not limited to *PIK3CA/AKT1/PTEN* alterations that may be detected in ctDNA. | Evaluation of pre- and on-treatment plasma samples for genetic alterations in ctDNA. |
| To investigate predictive markers of response and acquired resistance to capivasertib + fulvestrant vs placebo + fulvestrant including but not limited to biomarkers of AKT inhibitor sensitivity/resistance, immunological biomarkers, changes in gene expression and breast cancer subtyping. | Analysis of ctDNA and/or tumour tissue. |
| To investigate the effect of capivasertib + fulvestrant or placebo + fulvestrant on biomarkers that may be observed in paired pre- and on-treatment tumour tissue samples. | Analysis of matched pre- and post-treatment biopsy samples. |
| To assess patient-reported treatment tolerability of capivasertib + fulvestrant vs placebo + fulvestrant. | Patient-reported outcomes version of the CTCAE (PRO-CTCAE) and a single item on overall treatment tolerability using PGI-TT. |
| To assess patient-reported overall change in health status since the start of the study treatment and current severity of cancer symptoms in capivasertib + fulvestrant vs placebo + fulvestrant. | Evaluation of patient global impression of severity of cancer symptoms and change in health status using PGIS and PGIC, respectively. |
| To explore the impact of treatment and disease state on health state utility. | Health state utility data for economic evaluation will be derived by using the EQ-5D-5L health state utility index. |
| To perform future exploratory research into genes/genetic variation that may influence response (ie, distribution, safety, tolerability and efficacy) to capivasertib + fulvestrant vs placebo + fulvestrant and/or susceptibility to cancer. | Collection and storage of DNA for future analysis. |

| Exploratory objectives[a] | Endpoint |
|---|---|
| To conduct potential future exploratory research into factors that may influence the progression of cancer and/or response (efficacy, tolerability or safety) to capivasertib + fulvestrant vs placebo + fulvestrant. | Collection and storage of plasma, blood and tissue and/or analysis of surplus plasma, blood or tissue including patient-specific archival tumour tissue, if available. This may include but is not limited to the analysis of tumour and circulating biomarkers, such as DNA, RNA, proteins or metabolites. |

[a] China will be excluded from exploratory objectives requiring the provision of additional tumour or blood samples.

BRCA1/2, breast cancer gene 1/2; ctDNA, circulating tumour DNA; DNA, deoxyribonucleic acid; EQ-5D-5L, European Quality of Life 5-Domain 5-Level Scale; IHC, immunohistochemistry; IVD, in vitro diagnostic; NGS, next-generation sequencing; OS, overall survival; PFS, progression-free survival; PGIC, Patient Global Impression–Change; PGIS, Patient Global Impression–Severity; PGI-TT, Patient Global Impression–Treatment Tolerability; PRO-CTCAE, Patient-Reported Outcomes version of the Common Terminology Criteria for Adverse Events; RECIST v1.1, Response Evaluation Criteria in Solid Tumours version 1.1; RNA, ribonucleic acid; TFSC, time to first subsequent chemotherapy or death; vs, versus.

# 4 STUDY DESIGN

## 4.1 Overall design

This is a Phase III, double-blind, randomised study assessing the efficacy of capivasertib + fulvestrant vs placebo + fulvestrant for the treatment of patients with locally advanced (inoperable) or metastatic HR+/HER2− breast cancer following recurrence or progression on or after AI therapy. The study is powered to show a statistically significant difference between capivasertib + fulvestrant and placebo + fulvestrant in PFS in the overall population and the *PIK3CA/AKT/PTEN*-altered sub-population (dual primary endpoints) and OS (key secondary endpoint) in the overall population and OS in the *PIK3CA/AKT/PTEN*-altered sub-population will also be assessed.

The reciprocal feedback between the ER and PI3K signalling (see Section 2.2) suggests that the combination of endocrine therapy and inhibitors of the PI3K/AKT pathway may have clinical utility in the treatment of ER+ breast cancer, and responses would be expected both in patients whose tumours become dependent on the PI3K pathway upon treatment with fulvestrant, as well as patients whose tumours harbour a genetic alteration leading to an activated PI3K pathway. Therefore, PFS will be assessed in patients with qualifying activating mutations in the *PIK3CA* or *AKT1* genes and/or loss of function mutations or deletion of the *PTEN* gene (referred to as *PIK3CA/AKT1/PTEN*-altered) as dual primary endpoints. The list of eligible alterations is curated on the basis of the currently published literature for causal associations between pathogenicity and the alterations, using the AstraZeneca strategy for early-stage clinical studies as described by Carr et al 2016. The list of eligible variants will be defined and specified in the SAP prior to analysis.

It is expected that an estimated 930 patients will be screened so that approximately 700 patients can be randomised 1:1, with approximately 350 included in each arm. Of these

700 randomised patients, based on a prevalence of 40% to 45% for *PIK3CA/AKT1/PTEN* alterations (Cristofanilli et al 2016, Curtis et al 2012, Di Leo et al 2018, Hortobagyi et al 2016, Pereira et al 2016, Spoerke et al 2016), and a test failure rate of 20%, it is expected that a minimum of approximately 224 patients will test positive for tumours with these alterations and will be assigned to the *PIK3CA/AKT1/PTEN*-altered subgroup. The prevalence of *PIK3CA*/*AKT1*/*PTEN* mutation status will be monitored post-randomisation by central testing of formalin-fixed paraffin-embedded (FFPE) tumour samples collected before study entry. Given the proposed sample size (approximately 700 patients overall), it is expected that randomisation will be sufficient to ensure a balance between treatment arms with respect to mutational status. Enrolment will be open to all eligible patients irrespective of the *PIK3CA/AKT1/PTEN* status of their tumour(s); however adequate tumour tissue collected before study entry will be required for a central retrospective analysis.

In China, recruitment will continue until approximately 134 Chinese patients have been randomised, irrespective of whether or not the overall study enrolment has been reached. This is to ensure adequate participation of Chinese patients to satisfy China Regulatory Authority requirements.

Patients will receive weekly capivasertib (400 mg or placebo, oral, twice daily; 4 days on and 3 days off) and fulvestrant (at the approved dose regimen [500 mg intramuscular injections on Day 1 of Weeks 1 and 3 of Cycle 1, and then on Day 1, Week 1 of each cycle thereafter]).

All patients will attend a screening visit a maximum of 28 days prior to the start of study treatment.

Day 1 is defined as the randomisation date; study treatment should begin as soon as possible after randomisation, ideally the same day. Randomised patients will continue study treatment until objective radiological disease progression as defined by Response Evaluation Criteria in Solid Tumours version 1.1 (RECIST v1.1), unacceptable toxicity occurs, the patient withdraws consent or death. Following objective disease progression, further treatment options will be at the discretion of the investigator. If a patient discontinues treatment (and/or receives a subsequent cancer therapy) prior to progression, the patient should still continue to be followed until objective disease progression as defined by RECIST v1.1. Cross-over from placebo to capivasertib is not allowed.

The randomisation scheme will be stratified on the following factors:

- Liver metastases (yes vs no)
- Prior use of CDK4/6 inhibitors (yes vs no)
  - Patients may have received prior treatment with CDK4/6 inhibitors as part of standard treatment or within clinical trials (in the latter scenario, written confirmation

of exposure to the investigational agent rather than placebo is required to allow stratification at randomisation)

- Geographic location:
  - Region 1: United States, Canada, Western Europe, Australia, and Israel
  - Region 2: Latin America, Eastern Europe and Russia
  - Region 3: Asia

The dual primary endpoints, PFS in overall and *PIK3CA/AKT1/PTEN*-altered subgroup, are defined as the time from randomisation until disease progression based on the investigator's assessment according to RECIST v1.1, or death due to any cause regardless of whether the patient withdraws from therapy or receives another anticancer therapy prior to progression. The key secondary endpoint of OS is defined as the time from the date of randomisation until death due to any cause. A sensitivity analysis will be conducted using PFS assessed by blinded, independent central review (BICR) and defined using RECIST v1.1 criteria.

A study flow chart is illustrated in Figure 2 and the study design is summarised in Figure 1.

For details on what is included in the efficacy and safety endpoints, see Section 3. For details on treatments given during the study, see Section 6.1.

**Figure 2 Study flow chart**

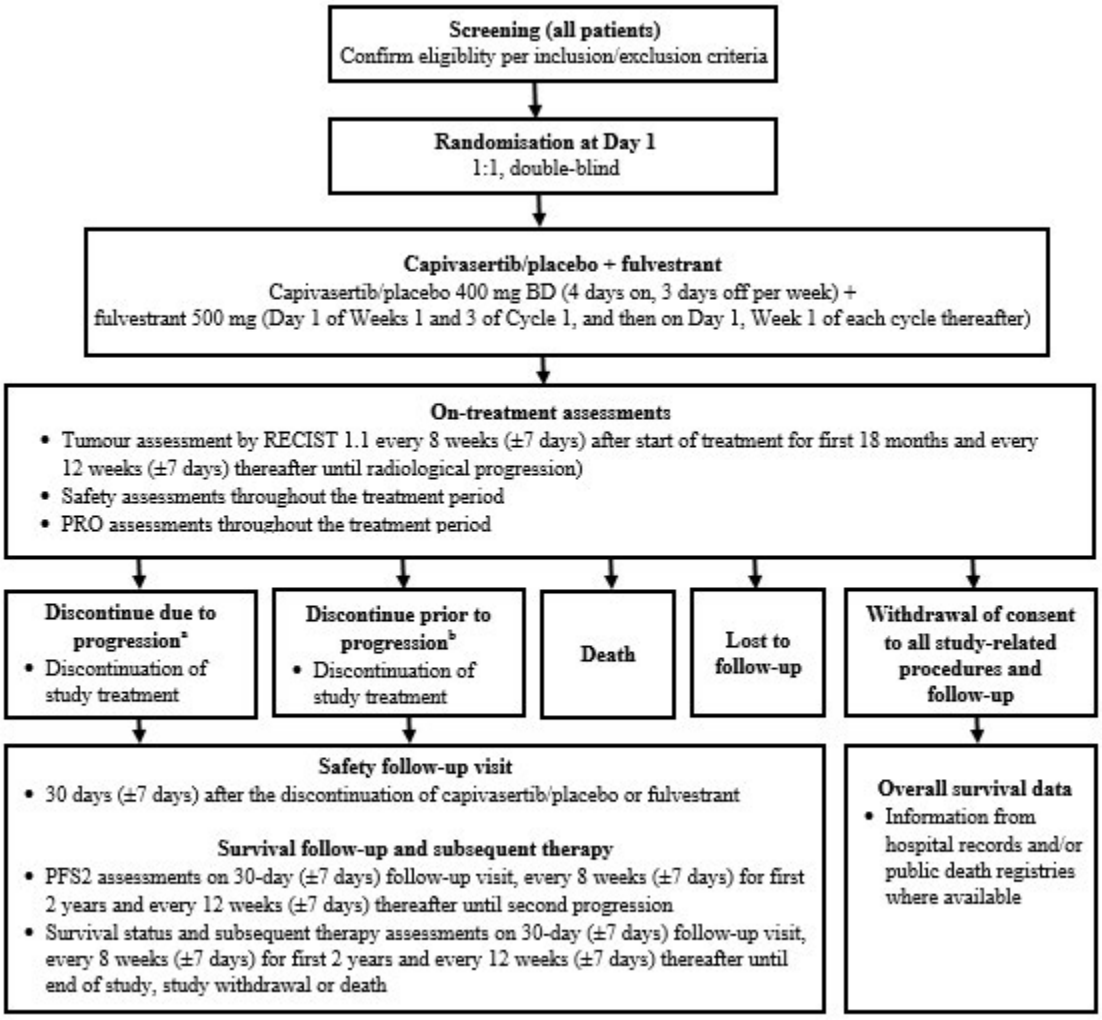


BD, twice daily; PFS2, time from randomisation to second progression or death; PRO, patient-reported outcome; RECIST v1.1, Response Evaluation Criteria in Solid Tumours version 1.1.

[a] If the patient discontinues due to progression, PROs (not including PGI-ITT and PRO-CTCAE) should be assessed at progression and every 4 weeks (±3 days) post discontinuation until PFS2.

[b] Patients who discontinue treatment prior to progression should continue to be scanned by RECIST v1.1 every 8 weeks (± 7days) for the first 18 months and every 12 weeks (±7 days) thereafter until progression, regardless of reason for treatment discontinuation. If the patient discontinues due to toxicity but does not progress, PROs (not including PGI-ITT and PRO-CTCAE) should be assessed every 4 weeks (±3 days) until progression, at progression and every 4 weeks (±3 days) post progression until PFS2.

### 4.1.1 Study oversight considerations

The study will use a Trial Steering Committee (TSC) and an external independent data monitoring committee (IDMC). Please see Section 9.5.1 and Appendix C for additional information.

## 4.2 Scientific rationale for study design

This randomised, double-blind, placebo-controlled, Phase III study will serve as confirmatory evidence of the clinical benefit, safety and tolerability of capivasertib + fulvestrant vs placebo + fulvestrant for the treatment of patients with locally advanced or metastatic HR+/HER2− breast cancer. Mandatory tissues samples collected from patients at screening will be used to retrospectively identify patients with qualifying *PIK3CA/AKT1/PTEN* alterations to investigate treatment with capivasertib + fulvestrant in this subgroup. The randomised, double-blind design of this study reduces potential for bias when assessing whether the combination of capivasertib + fulvestrant shows improved efficacy compared with fulvestrant monotherapy.

This is a Phase III study in adults (pre-menopausal women and post-menopausal women, and men) with HR+/HER2− locally advanced or metastatic breast cancer, after recurrence or progression on or after treatment with an AI. This represents a population that may have received up to 2 prior lines of endocrine therapy for metastatic disease, including combination therapy with a CDK4/6 inhibitor, and up to 1 line of chemotherapy in the metastatic setting; all patients will have received hormone (endocrine)-based therapy with an AI.

This study has been designed to also include pre- or peri-menopausal women, and men as per recent international guidelines (Cardoso et al 2018). These state that all treatment recommendations for post-menopausal patients should be extended to the pre-menopausal setting (with associated ovarian function suppression or ablation), encouraging the inclusion of patients who are initially pre- or peri-menopausal in clinical studies to avoid separate and duplicate trials. Studies evaluating new endocrine-based strategies should be designed to allow enrolment of both pre- and post-menopausal women, and men.

Hormone (or endocrine)-based therapy is the preferred initial treatment for ER+/HER2- or luminal ABC (Cardoso et al 2018) in the majority of cases, excluding only those with visceral crisis or concern for (or proof of) endocrine resistance, for whom chemotherapy is required. Current international treatment guidelines state that the preferred first-line endocrine therapy depends on the type and duration of adjuvant endocrine therapy as well as the disease-free interval. Available endocrine agents include but are not limited to AIs, tamoxifen, or fulvestrant, for pre- and peri-menopausal women (with ovarian function suppression/ablation), men (preferably with a LHRH agonist), and post-menopausal women. Of note, CDK4/6 inhibitors in combination with an AI or fulvestrant (±LHRH agonist depending on menopausal status) have substantially improved PFS compared with endocrine therapy alone.

These agents are, in certain markets, approved for advanced or metastatic HR+/HER2- breast cancer as initial endocrine-based therapy and/or after disease progression following endocrine therapy. As this class of agents has started to show a survival benefit (Im et al 2019, Slamon 2019, Sledge 2019), it is expected that their already wide use will continue to increase, especially in the first-line setting. Therefore, if they are available in the country in which a patient is being enrolled, they must be considered and excluded as treatment options before the patient can be considered for entering the study. For the above reasons and to make sure the enrolled population is representative of the general AI resistant HR+/HER2- advanced or metastatic breast cancer population, the study intends to enroll a minimum of 51% of patients previously treated with CDK4/6 inhibitor. Regarding additional endocrine-based options, the mTOR inhibitor everolimus is also approved in combination with the AI exemestane in patients with previous failure of treatment with letrozole or anastrozole, but the European Society for Medical Oncology (ESMO) guidelines state that the decision to treat must take into account the toxicities associated with this combination, lack of statistically significant OS benefit, cost and availability.

There is no single standard of care following endocrine therapy with or without a CDK4/6 inhibitor (as first- or second-line treatment). When patients progress, the choice of therapy depends on several factors: prior treatments (and response), disease burden, patient preference, and treatment availability, which varies in different countries around the world. Regardless of first- or second-line CDK4/6 inhibitor treatment, and despite multiple treatment options (eg, fulvestrant, everolimus plus exemestane), median post-progression treatment duration is limited, ranging from 4 to 8 months (Lupichuk et al 2019, Turner et al 2017). Patients eventually experience disease progression (endocrine resistance) and require chemotherapy. Thus, regardless of prior CDK4/6 inhibitor use, a considerable unmet medical need remains in this population for improved second- and additional-line treatment.

Available second or further lines of therapies are limited either with regard to the population able to gain benefit, or the magnitude of benefit. A Phase III study (BOLERO-2) of everolimus plus exemestane in patients with HR+/HER2- ABC post-AI progression, showed that the combination therapy improved PFS compared with exemestane alone from 3.2 to 7.8 months irrespective of *PIK3CA* genotype, albeit without significant OS improvement (Yardley et al 2013). More recently, the Phase III SOLAR-1 study demonstrated an improvement in PFS of 5.7 to 11 months with the addition of the alpha-specific PI3K inhibitor alpelisib to fulvestrant in patients with HR+/HER2− ABC with tumours harbouring a *PIK3CA* mutation, who had relapsed or progressed on an AI (André et al 2019), based on which the FDA granted approval. Of note, a clinically relevant benefit was not seen in the *PIK3CA* non-mutant cohort and is not included in the approval.

The purpose of this Phase III study is to further characterise and confirm the efficacy and safety of capivasertib + fulvestrant observed in the Phase II randomised FAKTION study

where the addition of capivasertib to fulvestrant resulted in a 5.5-month improvement in median PFS from 4.8 months on fulvestrant alone to 10.3 months (HR 0.57 [95% CI: 0.393, 0.841]) regardless of PI3K/AKT/PTEN pathway activation status. At the time of the PFS analysis, OS data were not mature (37%; 52 deaths reported); however, the data suggest an improvement of approximately 6 months on the combination treatment. The positive efficacy data are supported by the finding that the addition of capivasertib to fulvestrant appears overall well tolerated with no marked impact on the dose intensity of fulvestrant (see Section 2.1 and Section 2.2 for further information on the FAKTION study and the study rationale).

In this Phase III study, patients qualifying for the altered subgroup will be identified by post-randomisation central testing of tumour tissue collected prior to enrolment based on a prespecified list of molecular alterations, using a validated assay as described in Section 8.7.1. By enrolment of both the overall population and a minimum number of patients in the altered subgroup, the study design (including the sample size and planned statistical analysis approach described in Section 9) will allow for further investigation of outcomes in the 2 populations tested.

In light of the demonstrated activity of the combination of capivasertib + fulvestrant in the Phase II FAKTION study and the common usage of single agent fulvestrant in the management of advanced/metastatic HR+/HER2- breast cancer, fulvestrant is considered an appropriate comparator for this Phase III study. In addition, deploying fulvestrant as the control agent will enable a robust assessment of the contribution of components in the doublet regimen under study.

The rationale for the choice of stratification factors in this study is as follows:

- Liver metastases (yes vs no): The pattern and extent of metastatic disease are considered to be important factors with prognostic impact in HR+/HER2− (Solomayer et al 2000). Patients with HR+ ABC with non-visceral metastases and visceral non-liver metastases have significantly better outcomes from endocrine therapy than patients with liver metastases (Robertson et al 2019).
- Prior use of CDK4/6 inhibitors (yes vs no): Recent changes in the treatment landscape for HR+/HER2- ABC include the addition of CDK4/6 inhibitors to endocrine agents in the first- and second-line settings. Although limited and retrospective, currently available data suggest that patients who have received prior CDK4/6 inhibitors do not appear to have a different outcome obtained from subsequent standard of care therapies (Lupichuk et al 2019, Turner et al 2017). However, as the FAKTION study recruited at a time when CDK4/6 inhibitors were not available in the enrolling sites, it remains unclear whether prior treatment with a CDK4/6 inhibitor would influence the magnitude of benefit from treatment with capivasertib plus fulvestrant. The total number of CDK4/6 naïve patients will be limited to 49% of the total number of patients (Section 6.3).

- Geographic location (Region 1: US, Canada, Western Europe, Australia, Israel; Region 2: Latin America, Eastern Europe and Russia; Region 3: Asia): Considering there is no single standard of care for the intended population, geographic region has been included to account for possible differences in treatment paradigms and patient demographics in different territories.

Despite the documented benefits of endocrine-based therapy in HR+/HER2- ABC, intrinsic and acquired resistance remains a common feature that limits the success of this strategy, and lack of sensitivity (or primary resistance) to endocrine therapy appears associated with worst survival (Cristofanilli et al 2018). For the purpose of this study, the following definitions for endocrine resistance (Cardoso et al 2018) will be used for data collection:

- Primary endocrine resistance is defined as relapse while on the first 2 years of adjuvant endocrine therapy, or progressive disease within the first 6 months of first-line endocrine therapy for ABC, while on endocrine therapy
- Secondary endocrine resistance is defined as relapse while on adjuvant endocrine therapy but after the first 2 years, or relapse within 12 months of completing adjuvant endocrine therapy, or progressive disease ≥6 months after initiating endocrine therapy for ABC, while on endocrine therapy

**NOTE:** Patients who meet any criteria of secondary endocrine resistance are classified as such, regardless of whether they meet any aspect of the definition of primary resistance; patients who do not meet any of the criteria of secondary endocrine resistance are defined as having primary resistance.

In recent studies with novel therapies in the HR+/HER2- ABC setting, estimation of the effect on OS has been jeopardised by limited statistical power, and therefore, suboptimal assessment of their benefit. The proposed study is intended to address this issue by providing adequate power to show a statistically significant difference between capivasertib + fulvestrant and placebo + fulvestrant in both PFS and OS. The dual primary endpoints of this study are PFS in the overall population and PFS in the *PIK3CA/AKT1/PTEN*-altered population, as described in Section 3.1 and Section 9. Key secondary efficacy endpoints (see Section 3.2 and Section 9) are OS and objective response rate (ORR) in the overall and the *PIK3CA/AKT1/PTEN* -altered populations. Other secondary endpoints of this study that are appropriate include clinical benefit rate (CBR), duration of response (DoR), time to definitive deterioration of ECOG performance status from baseline, time from randomisation to second progression or death (PFS2), EORTC Quality of Life Questionnaire-Core 30 items (EORTC QLQ-C30) and EORTC Quality of Life Questionnaire-breast cancer specific module (EORTC QLQ-BR23) and will be assessed in both the overall and the *PIK3CA/AKT1/PTEN*-altered populations. The EORTC QLQ-C30 and EORTC QLQ-BR23 capture relevant symptoms and functional impacts for patients with cancer and breast cancer, respectively. Examination of change from baseline and time to deterioration in these concepts will allow for an assessment of the ability of the investigational treatment to preserve overall health-related

quality of life (HRQoL) and delay worsening of disease symptoms and functional limitations in the target population. Pharmacokinetic (PK) characterisation of capivasertib as well as the safety and tolerability of capivasertib + fulvestrant and placebo + fulvestrant will also be assessed as secondary endpoints.

Several exploratory endpoints will be evaluated (see Section 3.3). These include comparisons of capivasertib + fulvestrant vs placebo + fulvestrant on HRQoL (using patient-reported outcomes [PROs]) and resource utilisation (eg, hospital visits), the relationship between PK and selected endpoints and tumour/blood samples and biomarkers (eg, ctDNA for the assessment of biomarkers). Tumour and blood samples will be evaluated for additional exploratory biomarkers; these may include, but not be limited to, predictive markers of response and acquired resistance to study treatment and the *PIK3CA/AKT1/PTEN* alterations in ctDNA.

## 4.3 Justification for dose

### 4.3.1 Capivasertib

Several doses and schedules of capivasertib have been studied clinically (all twice daily [BD]). Monotherapy has been given as continuous (80 mg to 600 mg), intermittent 4 days on, 3 days off (480 mg to 640 mg) and intermittent 2 days on, 5 days off (640 mg to 800 mg) schedules. Dose- and/or concentration-response relationships have been demonstrated for pharmacodynamic biomarkers of AKT inhibition in tumour tissue and for the most important AEs (diarrhoea, rash, and hyperglycaemia of CTCAE grade 3-4) in clinical studies.

The recommended regimen for monotherapy (480 mg BD, 4 days on 3 days off) was selected based on pharmacodynamic parameters from tumour biopsies, PK data, and clinical safety. The recommended Phase II dose of capivasertib in combination with fulvestrant was determined by the run-in part (Ib) of the Phase Ib/II FAKTION study. The aim of Phase Ib of the study was to determine the maximum tolerated dose (MTD) for capivasertib given in combination with the licensed dose of fulvestrant (500 mg intramuscular on Days 1, 15, 29 and once monthly thereafter). The toxicity profile in patients who received capivasertib 400 mg BD, 4 days on 3 days off was consistent with prior capivasertib studies. Although no dose-limiting toxicities were observed, the Safety Review Committee concluded that 480 mg BD was unlikely to be tolerated for long-term dosing in combination with fulvestrant, and the 4 days on, 3 days off 400 mg BD schedule was selected as the combination dose with fulvestrant. This dose regimen was also supported by safety data from Study D3610C00001 and preclinical PK-pharmacodynamic efficacy mathematical modelling (Yates et al 2015).

Results from the randomised part of the FAKTION study (Phase II) provide evidence that the selected dose and schedule are efficacious with acceptable safety and tolerability in patients with ER+ ABC. Therefore, the dose regimen for this Phase III study is capivasertib

400 mg BD, 4 days on 3 days off in combination with fulvestrant at the approved dose regimen.

### 4.3.2 Fulvestrant

Fulvestrant will be used within its marketing authorisation at a monthly dose regimen of 500 mg (2 × 5 mL intramuscular injections), with an additional loading dose in Cycle 1. This regimen is commonly used in clinical practice and considered as standard of care for the treatment of HR+/HER2– metastatic breast cancer in participating countries. Monthly fulvestrant at the approved dose regimen showed an expected and manageable safety profile when combined with capivasertib in the FAKTION study (Jones et al 2019) and in Study D3610C00001 (Sections 2.2 and 2.3.1).

## 4.4 End of study definition

The end of the study is defined as the last visit/contact of the last patient undergoing the study (ie, 30-day follow-up visit if the last patient continues treatment until progression). A patient is considered to have completed the study when he/she has completed his/her last scheduled visit or his/her last scheduled procedure in the SoA (Section 1.1).

As the study is event driven, the accrual of the predetermined number of events included in the study endpoints will determine the duration of the data collection phase of the study. There will be a final DCO, defined by the time of the OS Final Analysis, which will take place after OS reaches approximately 70% maturity of events in the overall population and the *PIK3CA/AKT1/PTEN*-altered population (see details in Section 9.2). At this time the clinical database will close to new data with the exception of China or Japan if the number of patients is judged insufficient and an additional DCO is required to address regulatory requirements in China or Japan.

Patients are permitted to continue to receive treatment beyond the closure of the database if, in the opinion of the investigator, they are continuing to receive benefit. For patients who continue to receive treatment beyond the time of the final DCO, investigators will continue to report all SAEs only (no other data will be captured) to AstraZeneca Patient Safety until 30 days after study treatment is discontinued, in accordance with Section 8.3. It is recommended to continue observing ongoing patients at the frequency indicated within the study plans as described in the SoA (Table 1). Restrictions regarding concomitant medications (Section 6.5) will be followed while the patient is receiving capivasertib/placebo ± fulvestrant. A change in capivasertib/placebo ± fulvestrant dose should only occur for safety reasons, based on the investigator's judgement, and should generally follow the approach for dose reduction and discontinuation as described in this protocol. If an investigator learns of any SAEs, including death, at any time after a patient has completed the study, and he/she considers there is reasonable possibility that the event is causally related to the investigational product, the investigator should notify AstraZeneca Patient Safety.

Additionally, as stated in Section 8.3.3, any SAE or non-serious AE that is ongoing at this DCO must be followed up to resolution unless the event is considered by the investigator to be unlikely to be resolved, or the patient is lost to follow-up.

The study may be terminated at individual centres if the study procedures are not being performed to Good Clinical Practice (GCP) or if recruitment is slow. AstraZeneca may also terminate the entire study prematurely for operational reasons or if concerns for safety arise within this study, or in any other study, with capivasertib.

See Appendix C for guidelines for the dissemination of study results.

## 4.5 Study Conduct Mitigation During Study Disruptions Due to COVID-19 – Temporary Measures

The guidance given below supersedes instructions provided elsewhere in this CSP and should be implemented temporarily only during the COVID-19 outbreak (eg, during quarantines and resulting site closures, regional travel restrictions, and considerations if site personnel or study patients become infected with COVID-19) which would prevent the conduct of study-related activities at study sites, thereby compromising the study site staff or the patient's ability to conduct the study. The investigator or designee should contact the study Sponsor to discuss whether the mitigation plans below should be implemented.

To ensure continuity of the clinical study during the COVID-19 outbreak, temporary changes may be implemented to ensure the safety of study patients, maintain compliance with GCP, and minimize risks to study integrity. Where allowable by local health authorities, ethics committees, healthcare provider guidelines (eg, hospital policies) or local government, these temporary changes may include the following options:

- Obtaining reconsent for the mitigation procedures (note, in the case of verbal reconsent, the ICF should be signed at the patient's next contact with the study site).
- Rescreening: Additional rescreening for screen failure and to confirm eligibility to participate in the clinical study can be performed in previously screened participants. The investigator should confirm this with the designated study physician.
- Home or Remote visit: Performed by a site qualified Health Care Professional or Health Care Professional provided by a third party vendor.
- Telemedicine visit: Remote contact with the patients using telecommunications technology including phone calls, virtual or video visits, and mobile health devices.
- At-home study treatment administration: Performed by a site qualified Health Care Professional, or a Health Care Professional provided by a third party vendor, or by the patients or the patient's caregiver, if possible. Additional information related to the visit can be obtained via telemedicine.
- At-home or Remote Delivery of oral study treatment (capivasertib/placebo).

For further details on study conduct during COVID-19, refer to Appendix K. For further guidance during the COVID-19 pandemic, refer to Appendix L.

# 5 STUDY POPULATION

Prospective approval of protocol deviations to recruitment and enrolment criteria, also known as protocol waivers or exemptions, is not permitted.

Each patient should meet all of the inclusion criteria and none of the exclusion criteria for this study in order to be assigned/randomised to a study intervention. Under no circumstances can there be exceptions to this rule. Patients who do not meet the entry requirements are screen failures, refer to Section 5.4.

In this protocol, "enrolled" patients are defined as those who sign informed consent. "Randomised" patients are defined as those who undergo randomisation and receive a randomisation number.

For procedures for withdrawal of incorrectly enrolled patients see Section 7.3.

## 5.1 Inclusion criteria

Patients are eligible to be included in the study only if all of the following inclusion criteria and none of the exclusion criteria apply:

**Informed consent**

1 Capable of giving signed informed consent, which includes compliance with the requirements and restrictions listed in the informed consent form (ICF) and in the clinical study protocol (CSP)

2 Provision of signed and dated, written ICF prior to any mandatory study specific procedures, sampling, and analyses

3 For inclusion in the optional exploratory genetic and/or biomarker research, provision of signed and dated written genetic and/or biomarker informed consents, respectively, prior to collection of sample(s)

If a patient declines to participate in the optional exploratory genetic research and/or the optional biomarker research, there will be no penalty or loss of benefit to the patient. The patient will not be excluded from other aspects of the study.

The ICF process is described in Appendix C 3.

**Age**

4 Patients must be aged ≥18 years (aged ≥20 years in Japan) at the time of signing the ICF

**Type of patient and disease characteristics**

5 Adult females, pre- and/or post-menopausal, and adult males

- Pre-menopausal (and peri-menopausal, ie, those that do not meet the criteria for post-menopausal defined below) women can be enrolled if amenable to treatment with an LHRH agonist. Patients are to have commenced concomitant treatment with LHRH agonist prior to or on Cycle 1, Day 1 and must be willing to continue on it for the duration of the study
- Post-menopausal women are defined as:
  - aged ≥60 years of age, OR
  - aged <60 years of age and amenorrhoeic for at least 12 months following cessation of all exogenous hormonal treatments/chemotherapy/ovarian suppression/tamoxifen or similar. These patients should also have serum oestradiol and follicle stimulating hormone (FSH) levels confirmed as being within the standard laboratory reference range for post-menopausal females, OR
  - documented bilateral oophorectomy

6 Histologically confirmed HR+/HER2− breast cancer determined from the most recent tumour sample (primary or metastatic), as per the American Society of Clinical Oncology and College of American Pathologists guideline recommendations (Hammond et al 2010, Wolff et al 2018). To fulfil the requirement of HR+ disease, a breast cancer must express ER with or without co-expression of progesterone receptor. Therefore, tumours must be:

- ER+ defined as ≥1% of tumour cells stain positive for ER on immunohistochemistry (IHC) or, if no percentage is available, then an Allred IHC score of ≥3/8,
- Progesterone receptor positive defined as ≥1% of tumour cells stain positive for progesterone receptor on IHC or, if no percentage is available, then an Allred IHC score of ≥3/8; or progesterone receptor negative defined as <1% of tumour cells stain positive for progesterone receptor on IHC or, if no percentage is available, then an Allred IHC score of ≤2/8; or progesterone receptor unknown, and
- HER2− defined as 0 or 1+ intensity on IHC, or 2+ intensity on IHC and no evidence of amplification on in situ hybridisation (ISH), or if IHC not done, no evidence of amplification on ISH.

7 Metastatic or locally advanced disease with radiological or objective evidence of recurrence or progression (the cancer should have shown progression during or after most recent therapy); locally advanced disease must not be amenable to resection with curative intent (patients who are considered suitable for surgical or ablative techniques following potential down-staging with study treatment are not eligible)

8 Patients are to have received treatment with an AI (aromatase inhibitor) containing regimen (single agent or in combination) and have:

(a) Radiological evidence of breast cancer recurrence or progression while on, or within 12 months of the end of (neo)adjuvant treatment with an AI, OR

(b) Radiological evidence of progression while on prior AI administered as a treatment line for locally advanced or metastatic breast cancer (this does not need to be the most recent therapy)

9 Patients must have:

- at least 1 lesion, not previously irradiated, that can be measured accurately at baseline as ≥10 mm in the longest diameter (except lymph nodes which must have short axis ≥15 mm) with CT or MRI which is suitable for accurate repeated measurements, OR
- in absence of measurable disease as defined above, at least 1 lytic or mixed (lytic + sclerotic) bone lesion that can be assessed by CT or MRI; patients with sclerotic/osteoblastic bone lesions only in the absence of measurable disease are not eligible

10 Patients must be eligible for fulvestrant therapy as per local investigator assessment

11 Consent to submit and provide a mandatory FFPE tumour sample for central testing. A FFPE tissue block from the most recently collected pre-randomisation tumour sample (primary or recurrent cancer) is preferred. If it is not possible to provide a tissue block, 30 (minimum 20) freshly-cut unstained serial tumour slides are to be provided. Local pathology QC must be completed prior to randomisation to ensure the sample is suitable for next-generation sequencing (NGS) analysis, based on the requirements described in the Diagnostic Testing Manual

12 Patients must be able to swallow and retain oral medication

13 Eastern Cooperative Oncology Group (ECOG)/ World Health Organisation (WHO) performance status 0 or 1 with no deterioration over the previous 2 weeks and life expectancy of ≥12 weeks

**Reproduction**

14 Pre-menopausal patients with ovarian suppression induced by LHRH agonist should agree to use 2 forms of highly effective methods of accepted contraception (see Section 5.3.3.1) to prevent pregnancy during the study and for 2 years after the last dose of fulvestrant, or until 16 weeks after discontinuing capivasertib/placebo whichever occurs later

15 Male patients should use barrier contraception (ie, condoms) from the time of screening until 2 years after the last dose of fulvestrant or until 16 weeks after discontinuation of capivasertib/placebo, whichever occurs later. Male patients wishing to father children in the future should be advised to arrange for the freezing of sperm prior to the start of study treatment

**NOTE:** Female partners should be advised to use accepted contraception during their partner's participation in the study and for 2 years after the last dose of fulvestrant or until 16 weeks after discontinuing capivasertib/placebo, whichever occurs later.

## 5.2 Exclusion criteria

Patients meeting **any of the following exclusion criteria** are not to be enrolled in the study:

**Medical conditions**

1 A disease burden that makes the patient ineligible for endocrine therapy per the investigator's best judgement (eg, symptomatic visceral disease that is potentially life-threatening in the short-term)

2 Malignancies other than breast cancer within 5 years prior to study treatment initiation (except for appropriately treated carcinoma in situ of the cervix, non-melanoma skin carcinoma or Stage I endometrioid uterine cancer)

3 Radiotherapy with a wide field of radiation within 4 weeks prior to study treatment initiation (capivasertib/placebo) and/or radiotherapy with a limited field of radiation for palliation within 2 weeks prior to study treatment initiation (capivasertib/placebo)

4 Major surgery (excluding placement of vascular access) within 4 weeks prior to study treatment initiation

5 With the exception of alopecia, any unresolved toxicities from prior therapy greater than CTCAE grade 1 at the time of starting study treatment

6 Spinal cord compression or brain metastases unless asymptomatic, treated and stable and not requiring steroids within 4 weeks prior to study treatment initiation

7 Leptomeningeal metastases

8 Past medical history of interstitial lung disease, drug-induced interstitial lung disease, radiation pneumonitis which required steroid treatment, or any evidence of clinically active interstitial lung disease

9 Any of the following cardiac criteria:
   - Mean resting QT interval corrected by Fridericia's formula (QTcF) >470 msec obtained from 3 consecutive ECGs
   - Any clinically important abnormalities in rhythm, conduction or morphology of resting ECG (eg, complete left bundle branch block, third degree heart block)
   - Any factors that increase the risk of corrected QT interval (QTc) prolongation or risk of arrhythmic events such as heart failure, hypokalaemia, potential for torsades de pointes, congenital long QT syndrome, family history of long QT syndrome or unexplained sudden death under 40 years of age or any concomitant medication known to prolong the QT interval

- Experience of any of the following procedures or conditions in the preceding 6 months: coronary artery bypass graft, angioplasty, vascular stent, myocardial infarction, angina pectoris, congestive heart failure New York Heart Association (NYHA) grade ≥2
- Uncontrolled hypotension – systolic blood pressure <90 mmHg and/or diastolic blood pressure <50 mmHg
- Cardiac ejection fraction outside institutional range of normal or <50% (whichever is higher) as measured by echocardiogram (or multiple-gated acquisition [MUGA] scan if an echocardiogram cannot be performed or is inconclusive)

10 Clinically significant abnormalities of glucose metabolism as defined by any of the following:
- Patients with diabetes mellitus type 1 or diabetes mellitus type 2 requiring insulin treatment
- HbA1c ≥8.0% (63.9 mmol/mol)

11 Inadequate bone marrow reserve or organ function as demonstrated by any of the following laboratory values:
- Absolute neutrophil count $<1.5 \times 10^9$/L
- Platelet count $<100 \times 10^9$/L
- Haemoglobin <9 g/dL (<5.59 mmol/L). [**NOTE:** any blood transfusion must be >14 days prior to the determination of a haemoglobin ≥9 g/dL (≥5.59 mmol/L)]
- Alanine aminotransferase (ALT) and Aspartate aminotransferase (AST) >2.5 times upper limit of normal (ULN) if no demonstrable liver metastases or >5 × ULN in the presence of liver metastases
- Total bilirubin >1.5 × ULN (Patients with confirmed Gilbert's syndrome may be included in the study)
- Creatinine >1.5 × ULN concurrent with creatinine clearance <50 mL/min (measured or calculated by Cockcroft and Gault equation); confirmation of creatinine clearance is only required when creatinine is >1.5 × ULN

12 As judged by the investigator, any evidence of severe or uncontrolled systemic diseases, including uncontrolled hypertension, or active infection including hepatitis B, hepatitis C and human immunodeficiency virus (HIV), including those who have confirmed COVID-19. Screening for chronic conditions is not required

13 Known abnormalities in coagulation such as bleeding diathesis, or treatment with anticoagulants precluding intramuscular injections of fulvestrant or LHRH agonist (if applicable)

14 Refractory nausea and vomiting, malabsorption syndrome, chronic gastrointestinal diseases, inability to swallow the formulated product or previous significant bowel resection, or other condition that would preclude adequate absorption of capivasertib

15 Previous allogenic bone marrow or solid organ transplant

16 Known immunodeficiency syndrome

17 History of hypersensitivity to active or inactive excipients of capivasertib, fulvestrant and LHRH agonists (if applicable, ie, concomitant LHRH agonist required in this study) or drugs with a similar chemical structure or class to capivasertib, fulvestrant or LHRH agonists (if applicable, ie, concomitant LHRH agonist required in this study)

18 Evidence of dementia altered mental status or any psychiatric condition that would prohibit understanding or rendering of informed consent

19 Any other disease, metabolic dysfunction, physical examination finding, or clinical laboratory finding that, in the investigator's opinion, gives reasonable suspicion of a disease or condition that contraindicates the use of an investigational drug, may affect the interpretation of the results, render the patient at high risk from treatment complications or interferes with obtaining informed consent

**Prior/concomitant therapy**

20 More than 2 lines of endocrine therapy for inoperable locally advanced or metastatic disease

**NOTE**: If an attempt to downstage a locally advanced tumour with endocrine therapy was made in the absence of metastatic breast cancer (neoadjuvant), and the tumour operated upon, then this does not count as a line of therapy for ABC. In contrast, if the tumour remained inoperable, this treatment should be included as a line of therapy for ABC. Adjuvant endocrine therapy is not considered a line of therapy for ABC.

In addition, switching drugs within a line of therapy to manage toxicities in the absence of progressive disease does NOT count as a new line of therapy.

21 More than 1 line of chemotherapy for inoperable locally advanced or metastatic disease. Adjuvant and neoadjuvant chemotherapy are not classed as lines of chemotherapy for ABC

22 Prior treatment with any of the following:

- AKT, PI3K and mTOR inhibitors
- Fulvestrant, and other SERDs
- Nitrosourea or mitomycin C within 6 weeks prior to study treatment initiation
- Any other chemotherapy, immunotherapy, immunosuppressant medication (other than corticosteroids) or anticancer agents within 3 weeks prior to study treatment

initiation. A longer washout period may be required for drugs with a long half-life (eg, biologics) as agreed by the sponsor

- Potent inhibitors or inducers of CYP3A4 within 2 weeks prior to the first dose of study treatment (3 weeks for St John's wort) or drugs that are sensitive to CYP3A4 inhibition within 1 week prior to study treatment initiation. For details, see Appendix D
- Any concomitant medication that may interfere with fulvestrant safety and efficacy based on the prescribing information of fulvestrant and local clinical guidelines

**Prior/concurrent clinical study experience**

23 Previous randomisation in this study

24 Participation in another clinical study with a non-marketed investigational medicinal product (IMP) administered in the last 30 days or 5 half-lives, whichever is longer (for IMPs which are a previously marketed drug, regardless of indication, only Exclusion Criterion 22 applies). Vaccines for COVID-19 with emergency use authorization are not considered investigational agents.

**Other exclusions**

25 Involvement in the planning and/or conduct of the study (applies to both AstraZeneca staff and/or staff at the study site)

26 Judgment by the investigator that the patient should not participate in the study if the patient is unlikely to comply with study procedures, restrictions and requirements

27 Pregnant women (confirmed with positive pregnancy test) or breast-feeding women

## 5.3 Lifestyle restrictions

### 5.3.1 Meals and dietary restrictions

There is a potential for delayed and reduced absorption of capivasertib if it is administered with food. The clinical relevance of this is unknown. Until further information is available, it is recommended that patients fast from 2 hours before dosing to 1 hour after dosing, where possible. Additional fasting restrictions also apply on days where glucose is to be tested (Section 8.2.1.1).

In addition, patients should avoid herbal supplements (eg, St John's wort) and ingestion of large amounts of foods and beverages known to potently modulate CYP3A4 enzyme activity during study treatment. For example, no more than half a grapefruit, a small glass of grapefruit juice (approximately 125 mL) or 2 teaspoons of Seville orange marmalade should be consumed daily.

### 5.3.2 Caffeine, alcohol, and tobacco

No interactions with caffeine, alcohol or tobacco have been identified.

### 5.3.3 Contraception

#### 5.3.3.1 Females

Females of childbearing potential should use two forms of highly reliable methods of contraception from the time of screening until 2 years after the last dose of fulvestrant or until 16 weeks after discontinuing capivasertib/placebo, whichever occurs later. A permitted combination of acceptable and highly effective contraception methods includes the use of a barrier method of contraception with spermicidal combined with one of the highly effective methods of contraception described below.

The choice of contraception should be in line with international guidelines for ABC. Current guidelines advise against the use of hormonal contraception in patients with breast cancer (NCCN 2019).

Highly effective methods of contraception include:

- Intrauterine device
- Bilateral tubal occlusion
- Male partner sterilisation (with the appropriate post-vasectomy documentation of the absence of sperm in the ejaculate)
- True abstinence (ie, not engaging in sexual activity; however, periodic abstinence, the rhythm method, and the withdrawal method are not acceptable methods of birth control)

Acceptable methods of contraception include:

- Barrier methods of contraception: condom or occlusive cap (diaphragm or cervical/vault caps) with spermicidal foam/gel/film/cream/suppository

The investigator should discuss fertility preservation options for women of childbearing potential prior to the start of study treatment, in line with recommendations from international breast cancer guidelines for advanced disease.

Pre-menopausal and peri-menopausal women are required to have treatment for ovarian suppression with LHRH agonist in order to participate in the study. FSH and oestradiol will be monitored at screening, at Day 1 of each cycle, and when clinically indicated; due to the structural similarity of fulvestrant and oestradiol, fulvestrant may interfere with antibody-based oestradiol assays and may result in falsely increased levels of oestradiol (Fulvestrant SmPC 2018, Folkerd et al 2014). If deemed appropriate by the investigator, ad hoc monthly pregnancy tests can be performed.

#### 5.3.3.2 Males

Male patients should use barrier contraception (condoms) from the time of screening until 2 years after the last dose of fulvestrant or until 16 weeks after discontinuation of capivasertib/placebo, whichever occurs later. Male patients should refrain from donating sperm from the start of dosing until 2 years after the last dose of fulvestrant or until 16 weeks after discontinuation of capivasertib/placebo, whichever occurs later.

Female partners should be advised to use accepted contraception during their partners participation on the trial and for 2 years after the last dose of fulvestrant or until 16 weeks after discontinuation of capivasertib/placebo, whichever occurs later.

### 5.3.4 Other restrictions

Patients who are blood donors should not donate blood during the study. For blood donation after study participation, refer to WHO guidelines (WHO 2012).

## 5.4 Screen failures

Screen failures are defined as patients who signed the ICF to participate in the clinical study but are not subsequently randomised. A minimal set of screen failure information is required to ensure transparent reporting of screen failure patients to meet the Consolidated Standards of Reporting Trials (CONSORT) publishing requirements and to respond to queries from regulatory authorities. Minimal information includes demography, screen failure details, eligibility criteria, and any SAEs.

Individuals who do not meet the criteria for participation in this study (screen failure) may be rescreened once, upon the study physician's approval and agreement. However, rescreening should be documented so that its effect on study results, if any, can be assessed.

These patients should have the reason for study withdrawal, including failed inclusion/exclusion criteria, recorded in the electronic case report form (eCRF).

# 6 STUDY TREATMENTS

Study treatment is defined as any IMPs including marketed product comparator and placebo or medical device(s) intended to be administered to a study participant according to the CSP. Study treatment in this study refers to capivasertib, placebo and fulvestrant.

LHRH agonist is not an IMP and this should be prescribed in accordance with local guidelines, where applicable.

## 6.1 Treatments administered

### 6.1.1 Capivasertib/placebo

Capivasertib/placebo is considered an IMP and will be supplied by AstraZeneca. Details of capivasertib are shown in Table 5. Placebo tablets match the appearance of capivasertib.

A schematic presentation of the treatment schedule is presented in Figure 3.

**Table 5 Capivasertib/placebo**

| | |
|---|---|
| **Study treatment name:** | Capivasertib/placebo |
| **Dosage formulation:** | 160 mg and 200 mg film-coated tablets |
| **Route of administration:** | Oral |
| **Dosing instructions:** | 400 mg BD (2 tablets of 200 mg taken twice a day = total daily dose 800 mg) given on an intermittent weekly dosing schedule. Patients will be dosed on Days 1 to 4 in each week of a 28-day treatment cycle |
| **Packaging and labelling:** | Study treatment will be provided in white high-density polythene bottles. Each bottle will be labelled in accordance with Good Manufacturing Practice Annex 13 and per country regulatory requirement |

Where possible, all doses of capivasertib/placebo should be taken:

- At approximately the same time each day
- 12 hours apart
- In a fasted state (water to drink only) from at least 2 hours prior to the dose to at least 1-hour post-dose.

If vomiting occurs, a replacement dose should not be taken.

Should a patient miss a scheduled dose, the patient will be allowed to take the dose up to a maximum of 2 hours after the scheduled dose time, with a fasting state being maintained. If greater than 2 hours after the scheduled dose time, the missed dose should not be taken and the patient should take their allotted dose at the next scheduled time. If a patient needs to take the dose earlier for whatever reason, the patient can take the dose up to 2 hours earlier than the scheduled dose time. The patient should make every reasonable effort to take the capivasertib/placebo tablet(s) on time.

Capivasertib/placebo dose reductions are permitted (see Section 6.6).

Cross-over from placebo to capivasertib is not allowed.

**Figure 3 Treatment schedule**

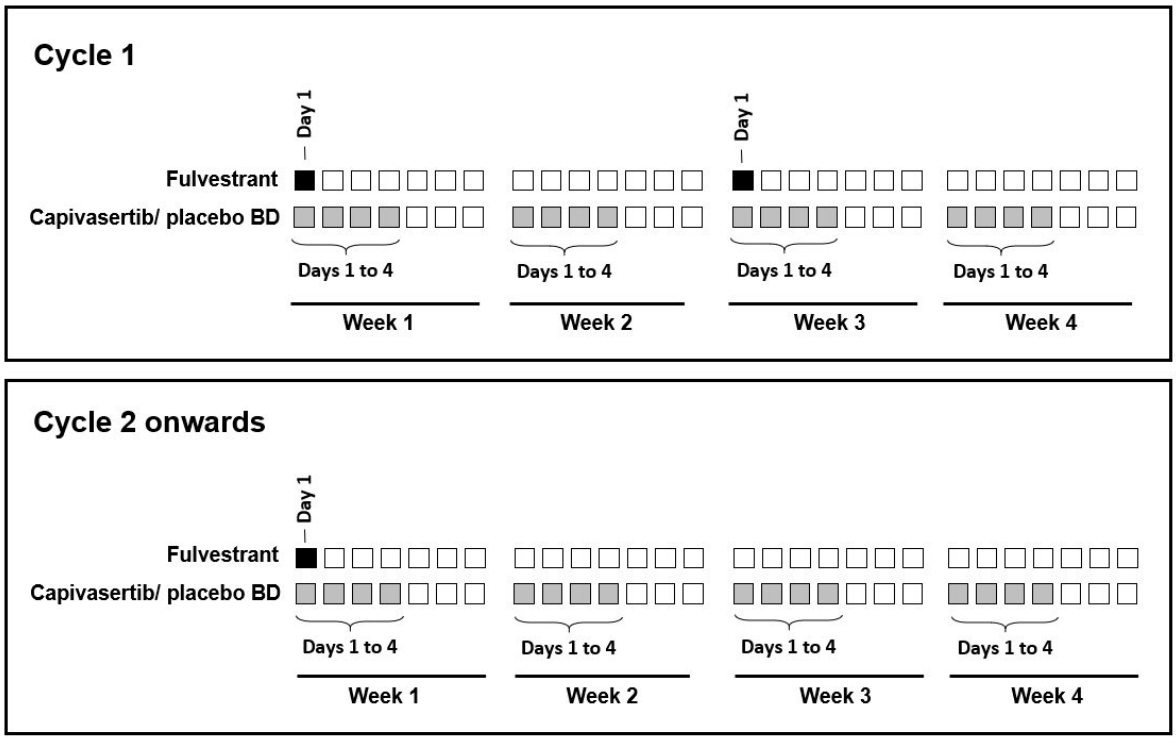


### 6.1.2 Fulvestrant

Fulvestrant is considered an IMP and commercial fulvestrant will be obtained centrally. Available stock will be used in keeping with the standard local practice. Details are shown in Table 6. Please refer to local prescribing information for information on formulation, preparation, and administration.

A schematic presentation of the treatment schedule is presented in Figure 3.

**Table 6 Fulvestrant**

| | |
|---|---|
| **Study treatment name:** | Fulvestrant |
| **Dosage formulation:** | 250 mg/ 5 mL solution for injection |
| **Route of administration:** | Intramuscular injection |
| **Dosing instructions:** | Patients will be administered 500 mg (2 injections) on Day 1 of Weeks 1 and 3 of Cycle 1, and then on Day 1, Week 1 of each cycle thereafter |
| **Packaging and labelling:** | Study treatment will be provided in a pre-filled syringe. Each syringe will be labelled in accordance with Good Manufacturing Practice Annex 13 and per local practice |

If injection-related symptoms occur, patients should be treated according to best medical practice and patients will be monitored until adequate resolution of signs and symptoms.

Dose reductions for fulvestrant are not permitted.

### 6.1.3 Duration of treatment

Study treatment will be continued until disease progression unless there is evidence of unacceptable toxicity, or if the patient requests to stop the study treatment.

If capivasertib/placebo is discontinued for reasons other than disease progression, the patient may continue on fulvestrant alone at the investigator's discretion. The patient must continue being scanned for RECIST v1.1 assessment every 8 weeks (±7 days) for the first 18 months and every 12 weeks (±7 days) thereafter, until objective disease progression (RECIST v1.1), administration of further anticancer therapy, withdrawal of consent or death whichever occurs first.

If fulvestrant is discontinued for reasons other than disease progression, the patient may continue on capivasertib/placebo alone at the investigator's discretion. The patient must continue being scanned for RECIST v1.1 assessment every 8 weeks (±7 days) for the first 18 months and every 12 weeks (±7 days) thereafter, until objective disease progression (even if further anticancer therapy is administered).

Patients with locally advanced disease who are considered suitable/planned for surgical or ablative techniques following potential down-staging with study treatment are not eligible. Cases in which a patient become amenable to surgery or ablative therapy must be communicated to the sponsor. Study drug must be discontinued prior to surgery. However, if, in the opinion of the investigator, the patient is continuing to receive benefit, they may be permitted to continue to restart treatment after surgery after discussion and agreement with the Sponsor. Patients should continue RECIST v1.1 assessment until progression.

## 6.2 Preparation/handling/storage/accountability

Fulvestrant must be stored in a refrigerator (2°C to 8°C) in the original packaging, to protect from light. Capivasertib must be stored in the pack provided, protected from light and at the recommended storage condition of below 30°C, and used according to the instructions on the label.

The investigator or designee must confirm appropriate temperature conditions have been maintained during transit for all study treatment received and any discrepancies are reported and resolved before use of the study treatment.

Only patients enrolled in the study may receive study treatment and only authorised site staff may supply or administer study treatment. All study treatments must be stored in a secure, environmentally controlled, and monitored (manual or automated) area in accordance with the labelled storage conditions with access limited to the investigator and authorised site staff.

The investigator, institution, or the head of the medical institution (where applicable) is responsible for study treatment accountability, reconciliation, and record maintenance (ie, receipt, reconciliation, and final disposition records).

Any study drug remaining at the end of the trial must be destroyed or returned according to the sites local standard operating procedures following authorisation by the sponsor.

## 6.3 Measures to minimise bias: randomisation and blinding

Capivasertib and placebo film-coated tablets will be identical in appearance and presented in the same packaging to ensure blinding of the capivasertib.

Capivasertib and placebo will be labelled using a unique kit identification (kit ID) number, which is linked to the randomisation scheme.

Fulvestrant will be allocated to be dispensed to each patient using the interactive web response system (IWRS) system (if centrally sourced) or via the local pharmacy (if locally sourced) at each patient dispensing visit. Routines for this will be described in the interactive voice response system (IVRS)/IWRS user manual that will be provided to each centre.

Eligible patients will be randomised in a 1:1 ratio (capivasertib + fulvestrant: placebo + fulvestrant). The planned treatment given to individual patients will be determined by a randomisation scheme that will be loaded into the IWRS database. The randomisation scheme will be produced by a process called AZRand (AZ Global Randomisation process) that incorporates a standard procedure for generating random numbers.

A blocked randomisation will be generated, and all centres will use the same list in order to minimise any imbalance in the number of patients assigned to each treatment group.

Eligibility will be established before treatment randomisation. It is recommended that patients commence study treatment as soon as possible after randomisation and ideally on the same day of randomisation.

In order to meet the required minimum of 51% of recruited patients pre-treated with CDK4/6 inhibitors, the actual proportions will be monitored during the course of the study. Selective recruitment of patients with prior CDK4/6 inhibitor treatment may be implemented in the event that approximately 343 CDK4/6 inhibitors naïve patients are randomised. This would be achieved via the IWRS system as prior CDK4/6 inhibition is a stratification factor.

If a patient withdraws from the study, then his enrolment/randomisation code cannot be reused. Withdrawn patients will not be replaced.

The randomisation code should not be broken except in medical emergencies when the appropriate management of the patient requires knowledge of the treatment randomisation.

The investigator must document and report the action to AstraZeneca, without revealing to the AstraZeneca staff the treatment given to the patient.

AstraZeneca retains the right to break the code for SAEs that are unexpected and are suspected to be causally related to an IMP and that potentially require expedited reporting to regulatory authorities. Randomisation codes will not be broken for the planned analyses of data until all decisions on the evaluability of the data from each individual patient have been made and documented.

In the event that the treatment allocation for a patient becomes known to the Investigator or other study staff involved in the management of study patients, or needs to be known to treat an individual patient for an AE, the Sponsor must be notified promptly by the Investigator and if possible, before unblinding.

## 6.4 Treatment compliance

Data regarding capivasertib/placebo dosing will be collected and entered into the eCRF along with reasons for missed dose(s) if applicable.

In addition, patients will be required to return all bottles of study medication. The number of tablets remaining will be counted by the research nurse/pharmacist/investigator, documented in the eCRF and returned to pharmacy. Site personnel are responsible for managing the IMP from receipt by the study site until the destruction or return of all unused IMP. The investigator is responsible for ensuring that the patient has returned all unused IMP.

## 6.5 Concomitant therapy

Any medication or vaccine, including over-the-counter or prescription medicines, vitamins, and/or herbal supplements, that the patient is receiving at the time of enrolment or receives during the study must be recorded along with:

- Reason for use
- Dates of administration including start and end dates
- Dosage information including dose and frequency

Guidance regarding potential interactions of capivasertib with concomitant medications is provided in Appendix D.

### 6.5.1 Allowed concomitant therapy

The following therapies are allowed during study participation, as applicable:

- No prophylactic anti-emetic therapy is planned for capivasertib/placebo but standard anti-emetic therapy including a 5-HT3-antagonist can be given as needed on a

prophylactic and treatment basis in compliance with the standards of the centre's local policy

- Anti-diarrhoeal therapy for symptomatic treatment of diarrhoea, as indicated in the toxicity management guideline (Section 8.4.5)
- Erythropoietin: patients already receiving erythropoietin at the time of screening for the study may continue it providing they have been receiving it for more than 1 month at the time study treatment is started. Prophylactic erythropoietin should not be started during Cycle 1 of the study but may be started during or after Cycle 2
- Blood transfusions are allowed at any time during the study

  **NOTE:** any blood transfusion before screening must be >14 days prior to the determination of a haemoglobin at screening; see Section 5.2].
- Patients may receive bisphosphonate or denosumab therapy for the treatment of bone metastases; however, these agents must be started prior to randomisation. Modification of the pre-study bisphosphonate or denosumab treatment regimen for bone metastasis requires the approval of the sponsor or study physician
- Patients may take corticosteroids; however, electrolyte and/or glucose levels should be monitored carefully due to the potential for corticosteroid-related metabolic disturbance
- Patients who need to begin anti-coagulant therapy while receiving study treatment may be treated, at the discretion of the investigator, with low molecular weight heparin (LMWH). The LMWH should be temporarily discontinued 12 to 24 hours prior to each injection of fulvestrant and then resumed 12 to 24 hours later (depending on the particular LMWH used). There is an increased risk of haemorrhage in these patients and the investigator should decide whether that risk is outweighed by the possible benefits of continued treatment. It is advised to apply direct pressure to the injection site in these patients
- Patients receiving antiplatelet therapy (acetylsalicylic acid, ticlopidine, clopidogrel, etc) may be at increased risk of bleeding from intramuscular injection. The investigator should decide whether that risk is outweighed by the possible benefits of continued treatment. It is advised to apply direct pressure to the injection site in these patients
- Supportive care and other medication, which is considered necessary for the patient's safety and wellbeing, may be given at the discretion of the investigator and recorded in the appropriate sections of the eCRF
- LHRH agonist: Pre- or peri-menopausal patients are to have commenced concomitant treatment with LHRH agonist prior to or on Cycle 1, Day 1 and must be willing to continue LHRH agonist treatment for the duration of the study. Male patients can receive concomitant LHRH agonist if deemed appropriate by the investigator
- Authorised/approved COVID-19 vaccines can be given to patients enrolled in CAPItello-291, following local prescribing information, and policies when considering if vaccination against COVID-19 is appropriate for the patients participating in the study. Investigators should apply their discretion assessing the risk benefit of COVID-19 vaccines for participants in clinical trials. The administration of any vaccine and any potential adverse events must be documented in the eCRF

Other concomitant medication may be given as medically indicated. Details (including doses, frequency, route and start and stop dates) of the concomitant medication given must be recorded in the patient's medical records and the eCRF.

### 6.5.2 Restricted concomitant therapy

The following concomitant therapies are restricted during study treatment:

- Other anticancer agents, other investigational agents and radiotherapy, although radiation for palliation at focal sites is permitted (as long as the radiation field covers less than 30% of the marrow and does not include whole pelvis or spine; disease progression in the bones has to be excluded by appropriate imaging investigations)
- Drugs or herbal supplements that are known to be potent inhibitors/inducers of CYP3A4 must be avoided, but moderate inhibitors/inducers may be used with caution. Drugs known to be sensitive to inhibition of CYP3A4 metabolism and/or MATE1 or OCT2 transport must be avoided or used with caution, depending on their therapeutic window. If co-administration is necessary, then additional monitoring for signs of toxicity related to increased exposure to the substrates is required
- Patients must not receive other investigational drugs while on study

Please refer to Appendix D for further guidance on potential PK interactions with capivasertib. For guidance regarding potential PK interactions with fulvestrant, please refer to the current local prescribing information (package insert).

Patients must be instructed not to take any medications, including herbal and dietary supplements, without first consulting with the investigator.

### 6.5.3 Other concomitant treatment

Medication other than that described in Section 6.5.2, which is considered necessary for the patient's safety and wellbeing, may be given at the discretion of the investigator and recorded in the appropriate sections of the eCRF. The concomitant administration of drugs known to prolong QT interval is restricted unless considered essential due to patient management, in which case, patients should be closely monitored. Information regarding drugs known to prolong the QT interval can be found on the CredibleMeds® website (URL: https://crediblemeds.org).

## 6.6 Dose modifications and delay

For capivasertib/placebo, dose reductions or holds are allowed as clinically indicated by the treating physician and in line with Table 7. For each patient, a maximum of 2 dose reductions will be allowed (Table 7). Dose re-escalations are not allowed for capivasertib/placebo. For guidance on capivasertib dose reductions for management of AEs, refer to Section 8.4.5.

Dose reductions for fulvestrant are not allowed.

Refer to locally approved prescribing information for guidance on fulvestrant monitoring and management of toxicities.

**Table 7 Dose levels for capivasertib/placebo and fulvestrant**

| Study treatment | Starting dose | Dose reduction levels |
|---|---|---|
| Capivasertib/placebo | 400 mg BD | Reduction level 1: 320 mg BD<br>Reduction level 2: 200 mg BD |
| Fulvestrant | 500 mg | Dose reductions not allowed |

In the event of an AE which the investigator considers to be related to the administration of study treatment, supportive therapy should be given at the discretion of the investigator. In addition, the investigator may decide that dosing of study treatment should be temporarily interrupted, a subsequent treatment cycle delayed, or study treatment permanently discontinued as per the guidelines outlined in Section 7.1.

Substantial acute toxicities should be managed as medically indicated and with temporary suspension of study drug, as appropriate.

A maximum break of 28 consecutive days for capivasertib/placebo dosing is allowed within each treatment cycle or between two consecutive cycles. A maximum delay of 35 days since a planned injection of fulvestrant is allowed.

Any further delays in capivasertib/placebo and/or fulvestrant treatment must be approved by the sponsor or study physician. Following a treatment break, the patient may resume treatment at the Day/Week in the cycle that they stopped.

## 6.7 Treatment after the end of the study

After the clinical study database closes, patients who continue to derive clinical benefit from study treatments in the opinion of the investigator may continue to receive study treatment until progression. These treatments will continue to be provided by AstraZeneca until the availability of either a local commercial supplier and reimbursement program or an early access program; at which point a change in supply will be requested. Treatment of patients beyond progression is not allowed.

Please refer to Section 4.4 for End of Study definition.

# 7 DISCONTINUATION OF TREATMENT AND PATIENT WITHDRAWAL

## 7.1 Discontinuation of study treatment

Patients will be discontinued from IMP in the following situations. Note that discontinuation from study treatment is NOT the same as a complete withdrawal from the study. Patients who discontinue study treatment should continue the study and be followed-up as outlined in the CSP.

- Objective disease progression assessed by RECIST v1.1
- Clinical disease progression/worsening of disease under investigation
- Patients incorrectly initiated on IMP
- Patient becomes pregnant
- Intercurrent illness that, in the judgment of the investigator, will affect assessments of clinical status to a significant degree or contraindicate further dosing
- AE or unacceptable toxicity
- Determination by the investigator that it is no longer safe for the patient or in the patient's best interest to continue therapy
- Patient request
- Severe non-compliance with study protocol in the judgement of the investigator and/or the sponsor
- Clinical need for concomitant or ancillary therapy (ie, non–protocol-specified anticancer therapy) that is not permitted in the study
- General or specific changes in the patient's condition that are unacceptable for further treatment in the judgment of the investigator

Patients found to be deriving benefit from treatment and tolerating treatment may continue therapy, subject to agreement between the treating and Sponsor physicians. The patients will be followed according to protocol-defined procedures and assessments. All protocol deviations will be reviewed and important protocol deviations will be identified prior to the unblinding of the study.

See the schedule of activities (SoA; Section 1.1) for data to be collected at the time of treatment discontinuation and follow-up and for any further evaluations that need to be completed.

All reasons for discontinuation of study treatment must be documented in the eCRF (see Section 7.1.2).

### 7.1.1 Temporary discontinuation

If study treatment will be interrupted for a period longer than permitted in the dose modification guidelines (Section 6.6), the investigator will need approval from the sponsor to restart the patient on study treatment.

### 7.1.2 Procedures for discontinuation of study treatment

The investigator should instruct the patient to contact the site before or at the time the patient decides to stop the study treatment. A patient who decides to discontinue study treatment must be asked about the reason(s) and the presence of any AEs. The date of last intake of study treatment should be documented in the eCRF. All study treatment should be returned by the patient at their next on-site study visit or unscheduled visit. Patients permanently discontinuing study treatment should be given locally available standard of care therapy, at the discretion of the investigator.

Discontinuation of study treatment, for any reason, does not impact on the patient´s participation in the study. The patient should continue attending subsequent study visits and data collection should continue according to the study protocol. If the patient does not agree to continue in-person study visits, a modified follow-up must be arranged to ensure the collection of endpoints and safety information. This could be a telephone contact with the patient, a contact with a relative or treating physician, or information from medical records. The approach taken should be recorded in the medical records. A patient that agrees to modified follow-up is not considered to have withdrawn consent or to have withdrawn from the study.

## 7.2 Lost to follow-up

A patient may be considered lost to follow-up if he or she fails to return for scheduled visits and is unable to be contacted by the study site.

The following actions must be taken if a patient fails to return to the clinic for a required study visit:

- The site must attempt to contact the patient and reschedule the missed visit as soon as possible and counsel the patient on the importance of maintaining the assigned visit schedule
- Before a patient is deemed lost to follow-up, the investigator or designee must make every effort to regain contact with the patient or next of kin by for example, repeat telephone calls, certified letter to the patient´s last known mailing address or local equivalent methods. These contact attempts should be documented in the patient's medical record

- Efforts to reach the patient should continue until the end of the study. Should the patient be unreachable at the end of the study the patient will be considered lost to follow-up, with unknown vital status at end of study and censored at the last follow-up contact

## 7.3 Withdrawal from the study

A patient may withdraw from the study (eg, withdraw consent) at any time at his/her own request, without prejudice to further treatment. If a patient withdraws consent, they will be specifically asked if they are withdrawing consent to all further participation in the study (treatment, assessments and follow-up) or withdrawing from treatment/assessments only and will allow further follow-up.

A patient who considers withdrawing from the study must be informed by the investigator about modified follow-up options (eg, telephone contact, a contact with a relative or treating physician, or information from medical records).

If the patient also withdraws consent for disclosure of future information, the sponsor may retain and continue to use any data collected before such a withdrawal of consent.

If a patient withdraws from the study, he/she may request destruction of any samples taken, and the investigator must document this in the site study records and inform the study monitor and study team.

A patient who withdraws consent will always be asked about the reason(s) and the presence of any AEs. The investigator will follow-up patients as medically indicated. The patient will return electronic patient-reported outcomes (ePRO) devices.

AstraZeneca or its delegate will request investigators to make every effort to collect information on patients' survival status (dead or alive; date of death when applicable) at the end of the study, including patients that withdrew consent or are classified as "lost to follow-up". Survival status can be obtained by site personnel from publicly available resources where it is possible, in accordance with local regulations. Knowledge of the survival status at study end in all patients is crucial for the integrity of the study.

See SoA (Section 1.1), for data to be collected at the time of study discontinuation and follow-up, and for any further evaluations that need to be completed. All study treatment should be returned by the patient.

# 8 STUDY ASSESSMENTS AND PROCEDURES

Study procedures and their timing are summarised in the SoA (see Section 1.1).

The investigator will ensure that data are recorded on the eCRFs. A Web Based Data Capture system will be used for data collection and query handling.

The investigator must ensure the accuracy and completeness for eCRFs which includes: legibility and timeliness of the data recorded and of the provision of answers to data queries according to the Clinical Study Agreement. The investigator will sign the completed eCRFs. A copy of the completed eCRFs will be archived at the study site.

Immediate safety concerns should be discussed with the sponsor immediately upon occurrence or awareness to determine if the patient should continue or discontinue study treatment.

Adherence to the study design requirements, including those specified in the SoA, is essential and required for study conduct.

All screening evaluations must be completed and reviewed to confirm that potential patients meet all eligibility criteria. The investigator will maintain a screening log to record details of all patients screened and to confirm eligibility or record reasons for screening failure, as applicable.

Procedures conducted as part of the patient's routine clinical management (eg, blood count) and obtained before signing of the ICF may be utilised for screening or baseline purposes provided the procedures met the protocol-specified criteria and were performed within the time frame defined in the SoA.

Repeat or unscheduled samples may be taken for safety reasons or for technical issues with the samples.

## 8.1 Efficacy assessments

### 8.1.1 RECIST v1.1 assessments

RECIST v1.1 tumour assessments will be performed using CT or MRI scans of the chest, abdomen and pelvis (with additional anatomy as clinically indicated by extent of disease) at baseline (no more than 28 days before the date of randomisation, as close as possible to the start of study treatment). Afterward, CT or MRI scans of the chest, abdomen and pelvis (with additional anatomy as clinically indicated by extent of disease) will be repeated every 8 weeks (±7 days) for the first 18 months and every 12 weeks (±7 days) thereafter, after start of treatment (Cycle 1, Week 1, Day 1) until objective radiological disease progression as defined by RECIST v1.1 (regardless of reason for treatment discontinuation). If an unscheduled assessment is performed (eg, to investigate clinical signs/symptoms of progression) and the patient has not progressed, every attempt should be made to perform the subsequent image acquisition at the next scheduled imaging visit. Patients who discontinue treatment prior to RECIST v1.1 progression (eg, discontinuation due to toxicity or clinical progression) should continue to be scanned until confirmed RECIST v1.1 progression. The same imaging modality and the same assessment (eg, the same contrast protocol for CT scans) should be performed at baseline and at all follow-up time-points. Guidelines on the valid methods of

assessment and the evaluation of objective tumour response using RECIST v1.1 are provided in Appendix A.

#### 8.1.1.1 Progression-free survival (PFS)

PFS is defined as the time from the date of randomisation until the date of disease progression, as defined by RECIST v1.1, or death (by any cause in the absence of progression) regardless of whether the patient withdraws from randomised therapy or receives another anticancer therapy prior to progression.

PFS will be assessed by investigator assessment. A supplementary analysis of PFS by BICR will be reported.

Patients who have not progressed or died at the time of analysis will be censored at the time of the latest date of assessment from their last evaluable RECIST v1.1 assessment. However, if the patient progresses or dies immediately after two or more consecutive missed visits, the patient will be censored at the time of the latest evaluable RECIST v1.1 assessment prior to the two missed visits.

#### 8.1.1.2 Clinical benefit rate (CBR)

CBR is defined as number of patients with complete or partial response or with stable disease maintained ≥24 weeks (as assessed by the investigator, using RECIST v1.1) divided by the number of patients in the analysis. Patients without a post-baseline tumour assessment will be considered to have no clinical benefit.

#### 8.1.1.3 Duration of response (DoR)

For patients who achieve complete or partial response per RECIST v1.1, DoR is defined as the time from the date of first documented response until date of documented progression (as assessed by the investigator, using RECIST v1.1) or death in the absence of disease progression. The end of response should coincide with the date of progression or death from any cause used for the PFS endpoint. The time of the initial response will be defined as the latest of the dates contributing towards the first visit response of complete or partial response.

If a patient does not progress following a response, then their DoR will use the PFS censoring time.

#### 8.1.1.4 Objective response rate (ORR)

ORR is defined as the percentage of patients with at least one investigator-assessed visit response of complete or partial response (as assessed by the investigator, using RECIST v1.1) and will be based on a subset of patients with measurable disease at baseline per the site investigator.

Data obtained up until progression, or last evaluable assessment in the absence of progression, will be included in the assessment of ORR. Patients who discontinue randomised treatment without progression, receive a subsequent anticancer therapy (note that for this analysis radiotherapy is not considered a subsequent anticancer therapy) and then respond will not be included as responders in the ORR.

### 8.1.2 Overall survival (OS)

OS is defined as the time from the date of randomisation until death due to any cause regardless of whether the patient withdraws from randomised therapy or receives another anticancer therapy.

Any patient not known to have died at the time of analysis will be censored based on the last recorded date on which the patient was known to be alive.

Assessments for survival will be conducted every 8 weeks for the first 2 years following objective disease progression or treatment discontinuation and then every 12 weeks.

Survival information may be obtained via telephone contact with the patient, patient's family, by contact with the patient's current physician, or local death registries as described in Section 7.3.

Survival calls will be made in the week following the date of DCO for each of the PFS or OS analyses, and if patients are confirmed to be alive or if the death date is post the DCO date, these patients will be censored at the date of DCO.

### 8.1.3 PFS2

Time from randomisation to second progression or death (PFS2) will be defined as the time from the date of randomisation to the earliest of the progression events subsequent to the first subsequent therapy, or death.

Patients alive and for whom a second disease progression has not been observed should be censored at date last known alive and without a second disease progression (ie, censored at the latest of the PFS or PFS2 assessment date if the patient has not had a second progression or death).

Following discontinuation of study treatment due to disease progression, as determined by investigator-based by RECIST v1.1 assessment, patients who started on subsequent cancer therapy post progression will continue to be followed at the 30-day follow-up visit, every 8 weeks (±7 days) for the first 2 years, and every 12 weeks (±7 days) thereafter for documentation of progression on second-line therapy. Determination of progressive disease for PFS2 will be by institutional call.

### 8.1.4 Time to definitive deterioration of the ECOG performance status

ECOG performance status will be used to assess physical health of patients. Time to definitive deterioration of ECOG performance status is defined as time from randomisation to the earlier of the date of the first definitive deterioration or death due to any cause. Deterioration is defined as a 1-point increase in ECOG score from baseline, and the deterioration is considered definitive if no improvements in the ECOG performance status are observed at a subsequent time of measurement during the treatment period, or at no further assessments following the time point where the deterioration is observed.

Patients who have not had definitive deterioration or have not died at the time of analysis will be censored at the time of the latest date of assessment from their last evaluable ECOG assessment. However, if the patient has definitive deterioration or dies immediately after two or more consecutive missed visits, the patient will be censored at the time of the latest evaluable ECOG assessment prior to the two missed visits.

### 8.1.5 Time to first subsequent chemotherapy or death (TFSC)

Time to first subsequent chemotherapy or death (TFSC) is defined as time from randomisation to the earlier of start date of the first subsequent chemotherapy after discontinuation of randomised treatment or death due to any cause.

Patients alive and not known to have had a first subsequent chemotherapy will be censored at the earliest of: date of study termination, date last known alive, DCO or, the last date that the patient was known not to have received a first subsequent chemotherapy.

### 8.1.6 Bone scan

An initial bone scan should be performed within 4 weeks prior to randomisation and repeated as necessary (see Appendix A [Isotopic bone scan]).

### 8.1.7 Patient-reported outcomes

PRO assessments will be used to examine the impact of treatment on disease-related symptoms, function, HRQoL, patient-perceived treatment tolerability, and overall health status. PROs have become increasingly important in evaluating the efficacy and tolerability of study treatments in clinical studies as part of the overall benefit/risk evaluation (Kluetz et al 2018). The PROs included in this study are as follows and will be administered in this order:

- EORTC QLQ-C30
- EORTC QLQ-BR23
- EuroQol 5-dimension, 5-level health state utility index (EQ-5D-5L)
- Patient-Reported Outcomes version of the Common Terminology Criteria for Adverse Events (PRO-CTCAE)

- Patient Global Impression–Severity (PGIS)
- Patient Global Impression–Change (PGIC)
- Patient Global Impression–Treatment Tolerability (PGI-TT)

The PROs will be assessed in accordance with the SoA (see Section 1.1) and Section 8.1.7.8. The individual questionnaires are provided in Appendix I.

#### 8.1.7.1 EORTC QLQ-C30

The EORTC QLQ-C30 was developed by the EORTC Quality of Life Group to assess HRQoL, functioning, and symptoms in cancer clinical trials. It has undergone extensive testing and validation as well as detailed cross-cultural testing and validation (Aaronson et al 1993). It is a 30-item self-administered questionnaire for all cancer types. Questions are grouped into 5 multi-item functional scales (physical, role, emotional, cognitive, and social), 3 multi-item symptom scales (fatigue, pain, and nausea/vomiting), a 2-item global QoL scale, 5 single items assessing additional symptoms commonly reported by cancer patients (dyspnea, loss of appetite, insomnia, constipation, and diarrhoea), and 1 item on the financial impact of the disease. All but 2 questions have 4-point scales: "Not at All," "A Little," "Quite a Bit," and "Very Much". The 2 questions concerning global health status and QoL have 7-point scales with ratings ranging from "Very poor" to "Excellent". For each of the 15 domains, final scores are transformed such that they range from 0 to 100, where higher scores indicate better functioning, better HRQoL, or greater level of symptoms (Aaronson et al 1993).

#### 8.1.7.2 EORTC QLQ-BR23

The EORTC QLQ-BR23 is a validated breast cancer-specific module used in conjunction with the core QLQ-C30 to assess breast cancer-specific HRQoL (Sprangers et al 1996). The self-administered instrument includes 23-items and yields 5 multi-item scores (body image, sexual functioning, arm symptoms, breast symptoms, and systemic therapy side effects). Items are scored on a 4-point verbal rating scale: "Not at All," "A Little," "Quite a Bit," and "Very Much". Scores are transformed to a 0 to 100 scale, where higher scores indicate better functioning, better HRQoL, or greater level of symptoms.

#### 8.1.7.3 EQ-5D-5L

The EQ-5D-5L will be used to explore the impact of treatment and disease state on health state utility.

The EQ-5D-5L, developed by the EuroQol Group, is a generic questionnaire that provides a simple descriptive profile of health and a single index value for health status for economic appraisal (van Reenen and Janssen 2015). The questionnaire comprises six questions that cover five dimensions of health (mobility, self-care, usual activities, pain/discomfort and anxiety/depression). Respondents also assess their health today using the EQ-VAS (visual

analogue scale), which ranges from 0 (worst imaginable health) to 100 (best imaginable health).

#### 8.1.7.4 PGIS

The PGIS item is included to assess how a patient perceives the overall severity of cancer symptoms over the past week. Patients will be asked to choose the response that best describes the severity of their overall cancer symptoms with options ranging from "No Symptoms" to "Very Severe".

#### 8.1.7.5 PGIC

The PGIC item is included to assess how a patient perceives their overall change in health status since the start of study treatment. This is a single-item questionnaire, and patients will choose from response options ranging from "Much Better" to "Much Worse".

#### 8.1.7.6 PGI-TT

The PGI-TT item is included to assess how a patient perceives the overall tolerability of the study treatment. This is a single-item questionnaire, and patients will rate the bother associated with any treatment-related symptoms using response options ranging from "Not at all" to "Very much".

#### 8.1.7.7 PRO-CTCAE

The PRO-CTCAE, developed by the National Cancer Institute (NCI) is included to address tolerability from the patients' perspective. The PRO-CTCAE will only be administered in those countries where a linguistically validated version is available. All applicable translations available during the study will be used. PRO-CTCAE is an item library of symptoms experienced by patients while undergoing treatment of their cancer. The items pre-selected for this study are based on a review of the treatment-related symptoms of capivasertib and fulvestrant and in consideration of symptoms that are already captured in the other PRO instruments with a view to minimise burden. The free text item in the PRO-CTCAE instrument is not included in the study, as the utility of this information and the analysis method have not been established.

#### 8.1.7.8 Administration of patient-reported outcome questionnaires

The PRO instruments will be self-administered by patients using a handheld electronic device in accordance with the SoA (see Section 1.1). PROs will be provided in the language of the country in which it will be administered, except for the PRO-CTCAE, which will only be administered in the languages where a linguistically validated version is available.

Patients will complete PRO assessments at home or at the study sites if the assessment timepoint coincides with a scheduled site visit. Similarly, during the post-progression period, patients will complete PROs at home or at the study site if a scheduled visit coincides with the

timepoint. If patients have had scans or other tests at an outside facility or missed a scheduled data collection site visit, PRO questionnaires should still be completed by patients at home according to the PRO completion schedule.

While PROs may be completed at home or site visits, patients should always bring the handheld electronic device to all site visits. It will take approximately 20 to 30 minutes for patients to complete the questionnaires.

If screening and randomisation are on the same day, ePRO questionnaire do not need to be repeated.

The following instructions should be followed when collecting PRO data via an electronic device:

- Following informed consent, PRO questionnaires completed at site visits must be completed prior to treatment administration and ideally before any discussions of health status to avoid biasing the patient's responses to the questions. As feasible, site staff should also ensure PRO questionnaires are completed prior to other study procedures, such as collection of laboratory samples, to further minimise bias.
- For PROs collected at site visits, PRO questionnaires must be completed by the patient in a quiet and private location and the patient given enough time to complete the PRO questionnaires at their own speed.
- The research nurse or appointed site staff must explain to patients the value and relevance of study participation and inform them that these questions are being asked to find out, directly from them, how they feel. The research nurse or appointed site staff should also stress that the information is not routinely shared with study staff. Therefore, if patients have any medical problems, they should discuss them with the doctor or research nurse separately from the PRO assessment.
- The research nurse or appointed site staff must train the patient on how to use the PRO device, using the materials and training provided by the PRO vendor, and provide guidance on whom to call if there are problems with the device if the patient is completing the PRO at home. All PRO questionnaires are to be completed using an ePRO device. If technical or other issues prohibit completion on the device, an appropriate back-up option may be considered with prior approval from AstraZeneca.
- The research nurse or appointed site staff must remind patients that there are no right or wrong answers and avoid introducing bias by not interpreting or clarifying items.
- The patient must not receive help from relatives, friends, or clinic staff deciding on answers to the PRO questionnaires. The responses are the patient's alone. If a patient uses visual aids (eg, glasses or contact lenses) for reading and does not have them when he or she visits the site, the patient will be exempted from completing the PROs at the visit.
- Site staff must not read or complete the PRO questionnaires on behalf of the patient. If the patient is unable to read the questionnaire (eg, is blind, illiterate, or not fluent in the available language), that patient is exempted from completing PRO questionnaires but

may still participate in the study. If the patient cannot complete the PRO questionnaires due to reasons other than being blind, illiterate, or fluent in language, the AstraZeneca study team must be contacted to determine if they can be exempted. Patients exempted in this regard should be flagged appropriately by the site staff in the source documents and the Review of PRO/Questionnaire/Diary eCRF.

- Site staff must administer questionnaires available in the language that the patient speaks and understands. Questions should not be read in an available language and translated into another language for the patient.
- It is vital that the PRO reporting is initiated at the baseline visit (Cycle 1, Week 1, Day 1), as specified in the study plan to capture the effect of study treatment. The handheld device must be charged and fully functional at the beginning of the baseline visit to ensure that the PROs can be completed at the start of the visit.
- Finally, the research nurse or appointed site staff will review the completion status of questionnaires during site visits and document the reason(s) why a patient could not complete assessments in the eCRF. The research nurse or appointed site staff must monitor compliance since minimising missing data is a key aspect of study success. It is important that the PRO device is charged and fully functional at the baseline visit. Compliance must be checked at each study visit and should be checked more frequently to identify problems early. If compliance drops to 85% or below, the patient will be flagged in the routine compliance report generated by the ePRO system, and a check-in call from the study site to ask the patient if he or she has any difficulties is highly recommended. A solution to enhance/resolve compliance should be discussed with the patient. Discussions and compliance review should be reflected in source documents.

## 8.2 Safety assessments

Planned time points for all safety assessments are provided in the SoA (see Section 1.1).

### 8.2.1 Clinical safety laboratory assessments

See Table 8 for the list of clinical safety laboratory tests to be performed and the SoA (Section 1.1) for the timing, frequency and additional information.

The date and time of each collection will be recorded in the appropriate eCRF.

Laboratory values that meet the criteria for CTCAE grade 3 or have changed significantly from baseline and are considered to be of clinical concern will be repeated/confirmed within 7 days and followed up as appropriate.

The investigator should make an assessment of the available results with regard to clinically relevant abnormalities. The laboratory results should be signed and dated and retained at centre as source data for laboratory variables. For information on how AEs based on laboratory tests should be recorded and reported, see Section 8.3.7.

Additional safety samples may be collected if clinically indicated at the discretion of the investigator. The date, time of collection and results (values, units and reference ranges) will be recorded on the appropriate eCRF.

The clinical chemistry, haematology and urinalysis will be performed at a local laboratory at or near to the investigator site. Sample tubes and sample sizes may vary depending on laboratory method used and routine practice at the site.

**Table 8 Laboratory safety variables**

| **Haematology/Haemostasis (whole blood)** | **Clinical Chemistry (serum or plasma)** |
|---|---|
| B-Haemoglobin | S/P-Albumin |
| B-Leukocyte (white blood cells [WBC]) | S/P-ALT |
| B-Leukocyte (WBC) % differential or absolute count:[a] | S/P-AST |
| B-Neutrophils | S/P-Alkaline phosphatase |
| B-Lymphocytes | S/P-Bilirubin, total |
| B-Eosinophils | S/P-Calcium, total |
| B-Platelet count | S/P-Creatinine |
| **Coagulation** | S/P-FSH (females only)[d] |
| **INR**[b] | S/P-Glucose |
| **Urinalysis** | S/P-Glycosylated haemoglobin (HbA1c) |
| U-Glucose | S/P-Magnesium |
| U-Protein | S/P-Oestradiol (females only)[d] |
| U-Blood | S/P-Potassium |
| U-Ketones | S/P-Total Protein |
| U-Microscopy[c] (red blood cells and white blood cells, bacteria, casts and crystals) | S/P Free T4[b] |
| | S/P TSH[b] |
| | S/P Troponin[e] I or T |
| | S/P-Sodium |
| | S/P-Urea nitrogen and/or Urea |
| | S/P-Lipids (total cholesterol, HDL, LDL, and triglycerides) |

[a] For Exclusion Criterion 11 (Section 5.2), if sites only collected % differential of neutrophils, the absolute neutrophil count will need to be calculated for eligibility, based on leukocytes (WBC) and % of neutrophils.
[b] Only at screening and as clinically indicated.
[c] Only perform if urinalysis is abnormal.
[d] FSH and oestradiol will be performed only in:
- Pre- and peri-menopausal women (ie, all women <60 years of age unless considered post-menopausal per Inclusion Criterion 5 [Section 5.1]) – at screening, at Day 1 Week 1 of each cycle, and when clinically indicated.

- Post-menopausal women <60 years of age – only at screening to confirm post-menopausal status and when clinically indicated.

All other women (ie, ≥60 years of age or with documented bilateral oophorectomy) do not need to have a check of FSH and oestradiol (refer to Inclusion Criterion 5 in Section 5.1).

[e] Test will only be performed when clinically indicated.

NOTE: In case a patient shows an AST or ALT ≥3 × ULN or total bilirubin ≥2 x ULN please refer to Appendix H 'Actions required in cases of increases in liver biochemistry and evaluation of Hy's Law', for further instructions.

ALT, alanine aminotransferase; AST, aspartate aminotransferase; B, blood; FSH, follicle-stimulating hormone; INR, International Normalised Ratio; P, plasma; S, serum; TSH, thyroid-stimulating hormone; U, urine.

#### 8.2.1.1 Glucose and glycosylated haemoglobin (HbA1c)

Blood glucose and HbA1c will be assessed according to the SoA (see Section 1.1). On blood glucose assessment days (incorporating clinical chemistry and glucose) it is requested that patients fast and refrain from caloric intake for ≥4 hours prior to the morning dose of study treatment.

- Glucose pre-dose (fasting) and 4 hours post dose (fasting or non-fasting) in Cycle 1, Week 1, Day 1
- Glucose (fasting) in Cycle 1, Week 3, Day 1; Cycle 2, Week 1, Day 1; and in Cycle 3 onwards on Day 1 (Week 1)
- Further glucose monitoring may be performed at the investigator's discretion as clinically indicated
- HbA1c to be measured at screening, pre-dose every 12 weeks, and at discontinuation

#### 8.2.1.2 Serum creatinine

Patients taking capivasertib/placebo and metformin in combination should have creatinine assessments conducted as part of the routine clinical chemistry with additional monitoring of creatinine at the discretion of the investigator (see Section 8.4.5.3).

### 8.2.2 Physical examinations

The physical examination will be performed at timelines as specified in the SoA (Section 1.1) and will include an assessment of the following: general appearance, respiratory, cardiovascular, abdomen skin, head and neck (including ears, eyes, nose and throat), lymph nodes, thyroid, musculoskeletal (including spine and extremities) and neurological systems.

Weight and height will be assessed at timelines as specified in the SoA (Section 1.1).

Investigators should pay special attention to clinical signs related to previous serious illnesses, new or worsening abnormalities may qualify as AEs, see Section 8.3.7 for details.

### 8.2.3 Vital signs

Vital signs (including blood pressure, pulse rate, respiratory rate, and body temperature) will be assessed at timelines as specified in the SoA (Section 1.1).

Blood pressure and pulse measurements will be assessed with a completely automated device. Manual techniques will be used only if an automated device is not available.

Blood pressure, pulse, and respiratory rate measurements should be preceded by at least 5 minutes of rest for the patient in a quiet setting without distractions (eg, television, cell phones).

Vital signs will be measured in a supine, semi-supine, or sitting position after 5 minutes rest and will include temperature, systolic and diastolic blood pressure, pulse, and respiratory rate. Three readings of blood pressure and pulse will be taken with a ≥1-minute interval in between and should be reported in the patient notes. The first reading should be rejected. The second and third readings should be averaged to give the measurement to be recorded in the eCRF. Respiratory rate will be counted only once.

### 8.2.4 Electrocardiograms

Triplicate 12-lead ECG will be obtained as outlined in the SoA (see Section 1.1).

All ECGs to be conducted as triplicate measurements, within approximately 5 minutes of starting (the 3 ECGs separated by approximately 2 minutes). Assessments should be performed as close as possible to, but within 30 minutes of the nominal time point.

The timing and number of ECGs may be altered depending on emerging safety information. Additional ECGs may be taken at the discretion of the investigator.

Twelve-lead ECGs will be obtained after the patient has been resting semi-supine for at least 10 minutes prior to times indicated. All ECGs should be recorded with the patient in the same physical position. A standardised ECG machine should be used and the patient should be examined using the same machine throughout the study if possible.

After paper ECGs have been recorded, the investigator or designated physician will review each of the ECGs and may refer to a local cardiologist if appropriate. A paper copy should be filed in the patient's medical records.

If an abnormal ECG finding at screening or baseline is considered to be clinically significant by the investigator, it should be reported as a concurrent condition. For all ECGs details of intervals PR, R-R, QRS, QT, and QTcF and an overall evaluation will be recorded (normal; abnormal and non-clinically significant; or abnormal and clinically significant).

### 8.2.5 MUGA scan/Echocardiogram

Assessments will be performed at screening and thereafter as clinically indicated. Bidimensional echocardiography (ECHO) is the preferred modality because of the global technetium [Tc-99m] shortage (but MUGA can be used alternatively).

The modality of the cardiac function assessments must be consistent within patient ie, if echocardiogram is used for the screening assessment and a follow-up assessment if clinically indicated, then echocardiogram should also be used for subsequent scans if required. The patients should also be examined using the same machine and operator whenever possible.

## 8.3 Collection of adverse events

The principal investigator is responsible for ensuring that all staff involved in the study are familiar with the content of this section.

The definitions of an AE or SAE can be found in Appendix E.

AEs will be reported by the patient (or, when appropriate, by a caregiver, surrogate, or the patient's legally authorised representative).

The investigator and any designees are responsible for detecting, documenting, and recording events that meet the definition of an AE or SAE. For information on how to follow/up AEs see Section 8.3.3.

All confirmed or suspected COVID-19 infection events must be recorded in the eCRF.

### 8.3.1 Method of detecting AEs and SAEs

Care will be taken not to introduce bias when detecting AEs and/or SAEs. Open-ended and non-leading verbal questioning of the patient is the preferred method to inquire about AE occurrences.

### 8.3.2 Time period and frequency for collecting AE and SAE information

AEs and SAEs will be collected from time of signature of the ICF, throughout the treatment period and including the 30-day follow-up period after discontinuation of study drug.

For patients who continue to receive treatment beyond the time of the final DCO, investigators will continue to report all SAEs to AstraZeneca Patient Safety until 30 days after study treatment is discontinued (see Section 4.4).

All SAEs will be recorded and reported to the sponsor or designee within 24 hours, as indicated in Appendix E. The investigator will submit any updated SAE data to the sponsor within 24 hours of it being available.

Investigators are not obligated to actively seek AEs or SAEs in patients who have already completed follow-up. However, if the investigator learns of any SAE, including a death, at any time after a patient’s last visit and he/she considers the event to be reasonably related to the study treatment or study participation, the investigator may notify the sponsor.

The method of recording, evaluating, and assessing causality of AEs and SAEs and the procedures for completing and transmitting SAE reports are provided in Appendix E.

### 8.3.3 Follow-up of AEs and SAEs

After the initial AE/SAE report, the investigator is required to proactively follow each patient at subsequent visits/contacts. All SAEs/non-serious AEs/AEs of special interest (as defined in Appendix E), will be followed until resolution, stabilisation, the event is otherwise explained, or the patient is lost to follow-up.

A post-study assessment will be performed preferably within 7 days of the time that the IMP is permanently discontinued.

Safety follow-up should be carried out with the patient 30 days (± 7 days) after the discontinuation of study treatment to follow-up any SAEs/AEs and concomitant medications (including any subsequent cancer therapy).

Any AE/SAE/abnormal laboratory findings that are ongoing at the time of study treatment discontinuation or any new treatment related events within 30 days of study treatment, must be followed up to resolution or until the event becomes stable (or returns to baseline) or is unlikely to resolve further in the opinion of the investigator. Any AEs that are unresolved at the patient's last AE assessment or other assessment/visit as appropriate in the study are followed up by the investigator for as long as medically indicated, but without further recording in the eCRF. AstraZeneca retains the right to request additional information for any patient with ongoing AE(s)/SAE(s) at the end of the study, if judged necessary.

### 8.3.4 Adverse event data collection

The following variables will be collected for each AE:

- AE (verbatim)
- The date when the AE started and stopped
- CTCAE grade and changes in CTCAE grade
- Whether the AE is serious or not
- Investigator causality rating against the IMP(s) (yes or no)
- Action taken with regard to IMP(s)
- Outcome

In addition, the following variables will be collected for SAEs:

- Date AE met criteria for SAE
- Date investigator became aware of SAE
- AE is serious due to
- Date of hospitalisation

- Date of discharge
- Probable cause of death
- Date of death
- Autopsy performed
- Causality assessment in relation to study procedure(s)
- Causality assessment to other medication

### 8.3.5 Causality collection

The investigator will assess causal relationship between IMP and each AE, and answer 'yes' or 'no' to the question 'Do you consider that there is a reasonable possibility that the event may have been caused by the IMP?'.

For SAEs, causal relationship will also be assessed for other medication and study procedures. Note that for SAEs that could be associated with any study procedure the causal relationship is implied as 'yes'.

A guide to the interpretation of the causality question is found in Appendix E to the CSP.

### 8.3.6 Adverse events based on signs and symptoms

All AEs spontaneously reported by the patient or reported in response to the open question from the study site staff (eg, "Have you had any health problems since the previous visit/you were last asked?") or revealed by observation will be collected and recorded in the eCRF. When collecting AEs, the recording of diagnoses is preferred (when possible) to recording a list of signs and symptoms. However, if a diagnosis is known and there are other signs or symptoms that are not generally part of the diagnosis, the diagnosis and each sign or symptom will be recorded separately.

### 8.3.7 Adverse events based on examinations and tests

The results from the CSP mandated laboratory tests and vital signs will be summarised in the CSR. Deterioration as compared to baseline in protocol-mandated laboratory values, vital signs and ECG abnormalities should therefore only be reported as AEs if they fulfil any of the SAE criteria or are the reason for discontinuation of treatment with the IMP or if they are considered to be clinically relevant as judged by the investigator (which may include but is not limited to consideration as to whether treatment or non-planned visits were required or other action was taken with the study treatment, eg, dose adjustment or drug interruption).

If deterioration in a laboratory value/vital sign is associated with clinical signs and symptoms, the sign or symptom will be reported as an AE and the associated laboratory result/vital sign will be considered as additional information. Wherever possible the reporting investigator uses the clinical, rather than the laboratory term (eg, anaemia vs low haemoglobin value). In

the absence of clinical signs or symptoms, clinically relevant deteriorations in non-mandated parameters should be reported as AE(s).

Deterioration of a laboratory value, which is unequivocally due to disease progression, should not be reported as an AE/SAE.

Any new or aggravated clinically relevant abnormal medical finding at a physical examination as compared with the baseline assessment will be reported as an AE unless unequivocally related to the disease under study (see Section 8.3.9).

### 8.3.8 Hy's law

Cases where a patient shows elevations in liver biochemistry may require further evaluation and occurrences of AST or ALT ≥ 3 × ULN together with total bilirubin ≥ 2 × ULN, without any findings of cholestasis (as indicated by elevated serum alkaline phosphatase [ALP]), may need to be reported as SAEs. Please refer to Appendix H for further instruction on cases of increases in liver biochemistry and evaluation of Hy's Law.

### 8.3.9 Disease progression

Disease progression can be considered as a worsening of a patient's condition attributable to the disease for which the IMP is being studied. It may be an increase in the severity of the disease under study and/or increases in the symptoms of the disease. The development of new or progression of existing metastasis to the primary cancer under study should be considered as disease progression and not an AE. Events, which are unequivocally due to disease progression, should not be reported as an AE during the study.

### 8.3.10 Adverse Events of Special Interest

Adverse Events of Special Interest (AESIs) are events of scientific and medical interest specific to the further understanding of capivasertib safety profile and require close monitoring and rapid communication by the investigators to AstraZeneca. An AESI can be serious or non-serious. All AESIs will be recorded in the eCRF. Serious AESIs will be recorded and reported as per Section 8.4.1.

The AESIs for this study are:

- Urinary tract infection
- Hyperglycaemia
- Non-infectious diarrhoea
- Infective pneumonia
- Rash
- Stomatitis

- QT Prolongation

## 8.4 Safety reporting and medical management

Investigators should refer to locally approved prescribing information for guidance on fulvestrant monitoring and the management of fulvestrant-related toxicities.

### 8.4.1 Reporting of serious adverse events

All SAEs have to be reported, whether or not considered causally related to the IMP, or to the study procedure(s). All SAEs will be recorded in the eCRF.

If any SAE occurs in the course of the study, then investigators or other site personnel inform the appropriate AstraZeneca representatives within one day ie, immediately but **no later than 24 hours** of when he or she becomes aware of it.

The designated AstraZeneca representative works with the investigator to ensure that all the necessary information is provided to the AstraZeneca Patient Safety data entry site **within 1 calendar day** of initial receipt for fatal and life-threatening events **and within 5 calendar days** of initial receipt for all other SAEs.

For fatal or life-threatening AEs where important or relevant information is missing, active follow-up is undertaken immediately. Investigators or other site personnel inform AstraZeneca representatives of any follow-up information on a previously reported SAE within 1 calendar day ie, immediately but **no later than 24 hours** of when he or she becomes aware of it.

Once the investigators or other site personnel indicate an AE is serious in the electronic data capture (EDC) system, an automated email alert is sent to the designated AstraZeneca representative.

If the EDC system is not available, then the investigator or other study site staff reports a SAE to the appropriate AstraZeneca representative by telephone, email or fax.

The AstraZeneca representative will advise the investigator/study site staff how to proceed investigators or other site personnel send relevant eCRF modules by fax to the designated AstraZeneca representative.

For further guidance on the definition of a SAE, see Appendix E of the Clinical Study Protocol.

#### 8.4.1.1 Regulatory Reporting Requirements for SAEs

Prompt notification by the investigator to the sponsor of a SAE is essential so that legal obligations and ethical responsibilities towards the safety of participants and the safety of a study intervention under clinical investigation are met.

The sponsor has a legal responsibility to notify both the local regulatory authority and other regulatory agencies about the safety of a study intervention under clinical investigation. The sponsor will comply with country-specific regulatory requirements relating to safety reporting to the regulatory authority, Institutional Review Boards (IRB)/Independent Ethics Committees (IEC), and investigators.

For all studies except those utilizing medical devices investigator safety reports must be prepared for suspected unexpected serious adverse reactions (SUSAR) according to local regulatory requirements and sponsor policy and forwarded to investigators as necessary.

An investigator who receives an investigator safety report describing a SAE or other specific safety information (eg, summary or listing of SAEs) from the sponsor will review and then file it along with the Investigator's Brochure or and will notify the IRB/IEC, if appropriate according to local requirements.

### 8.4.2 Pregnancy

A pregnancy test will be locally administered to female patients at screening, on Day 1 of the study (Cycle 1, Week 1, Day 1), at discontinuation of capivasertib, and as clinically indicated. Confirmation of absence of pregnancy is strongly recommended in case of delayed menstrual period (including infrequent or irregular menstrual cycles).

All pregnancies and outcomes of pregnancy should be reported to AstraZeneca (see Section 8.4.2.1) except where the pregnancy is discovered before the study patient has received any study treatment.

Abnormal pregnancy outcomes (eg, spontaneous abortion, foetal death, stillbirth, congenital anomalies, ectopic pregnancy) are considered SAEs.

#### 8.4.2.1 Maternal exposure

If a patient becomes pregnant during the course of the study, study treatment should be discontinued immediately.

Pregnancy itself is not regarded as an AE unless there is a suspicion that the IMP under study may have interfered with the effectiveness of a contraceptive medication. Congenital abnormalities/birth defects and spontaneous miscarriages should be reported and handled as SAEs. Elective abortions without complications should not be handled as AEs. The outcome of all pregnancies (spontaneous miscarriage, elective termination, ectopic pregnancy, normal

birth or congenital abnormality) should be followed up and documented even if the patient was discontinued from the study.

If any pregnancy occurs in the course of the study, then the investigator or other site personnel informs the appropriate AstraZeneca representatives within 1 day ie, immediately but **no later than 24 hours** of when he or she becomes aware of it.

The designated AstraZeneca representative works with the investigator to ensure that all relevant information is provided to the AstraZeneca Patient Safety data entry site within 1 or 5 calendar days for SAEs and within 30 days for all other pregnancies.

The same timelines apply when outcome information is available.

#### 8.4.2.2 Paternal exposure

Male patients should refrain from fathering a child or donating sperm during the study and for 2 years after the last dose of fulvestrant or until 16 weeks after discontinuing capivasertib/placebo, whichever occurs later. Patients wishing to father children should be advised to arrange for freezing of sperm samples prior to the start of study treatment.

Pregnancy of a patient's partner is not considered to be an AE. However, the outcome of all pregnancies (spontaneous miscarriage, elective termination, ectopic pregnancy, normal birth or congenital abnormality), occurring from the date of the first dose until 2 years after the last dose of fulvestrant or until 16 weeks after discontinuing capivasertib/placebo whichever occurs later, should, if possible, be followed up and documented.

### 8.4.3 Overdose

There is currently no specific treatment in the event of an overdose with capivasertib and possible symptoms of overdose are not established. Capivasertib must only be used in accordance with the relevant CSP. AEs associated with overdose should be treated in response to symptoms. Any dose, or frequency of dosing, that exceeds the dose regimen specified in the CSP should be reported as an overdose. The MTD for capivasertib is 400 mg BD in combination with fulvestrant and 480 mg BD as monotherapy. Fulvestrant must be used according to local prescribing information.

Adverse reactions associated with overdose should be treated symptomatically and should be managed appropriately.

- An overdose with associated AEs is recorded as the AE diagnosis/symptoms on the relevant AE modules in the eCRF and on the Overdose eCRF module
- An overdose without associated symptoms is only reported on the Overdose eCRF module

If an overdose on an AstraZeneca study drug occurs in the course of the study, then the investigator or other site personnel inform appropriate AstraZeneca representatives immediately, or no later than 24 hours of when he or she becomes aware of it.

The designated AstraZeneca representative works with the investigator to ensure that all relevant information is provided to the AstraZeneca Patient Safety data entry site.

- For overdoses associated with a SAE, the standard reporting timelines apply, see Section 8.3.2. For other overdoses, reporting must occur within 30 days

### 8.4.4 Medication error

If a medication error occurs in the course of the study, then the investigator or other site personnel should inform the appropriate AstraZeneca representatives within 1 day ie, immediately but no later than 24 hours of when he or she becomes aware of it.

The designated AstraZeneca representative works with the investigator to ensure that all relevant information is completed within 1 (initial fatal/life-threatening or follow-up fatal/life-threatening) or 5 (other serious initial and follow-up) calendar days if there is an SAE associated with the medication error (see Section 8.3.2) and within 30 days for all other medication errors.

The definition of a medication error can be found in Appendix E.

### 8.4.5 Management of capivasertib-related toxicities

#### 8.4.5.1 Capivasertib/placebo dose modification and guidance

Please refer to Section 6.6 for guidance on dose modifications.

#### 8.4.5.2 Dose modifications due to general capivasertib-related toxicities

Treatment with capivasertib/placebo should be temporarily interrupted for any intolerable AE regardless of grade or for any AE grade ≥3, that is not attributable to the disease under investigation, where the investigator considers the AE of concern to be specifically associated with capivasertib. Dose modification guidelines for capivasertib-related toxicities are shown in Table 9. Appropriate and optimal treatment of the toxicity is assumed prior to considering dose modifications. The study physician may be consulted prior to discontinuation of study drug due to toxicities. Please see Section 8.4.5.3 for the management of capivasertib specific toxicities including hyperglycaemia, maculo-papular rash and other skin reactions, and diarrhoea.

**Table 9 Dose modifications for general capivasertib-related toxicities**

| NCI CTCAE v5 Toxicity Grade: | Actions: |
|---|---|
| **Grade 1 or 2 clinically significant or intolerable**<br>• Resolves to baseline or clinically tolerable within 21 days of onset<br>• Does not resolve or remains intolerable within 21 days of onset | Hold dosing and follow guidance below, depending on outcome<br>• Resume dosing at same dose or one reduced dose level as clinically appropriate<br>• Discontinue study drug and observe patient until resolution |
| **Grade ≥3**<br>• Grade ≥3 toxicity for ≤21 days and resolves to ≤ grade 2 or baseline within 21 days of onset<br>• Grade ≥3 toxicity for >21 days | Hold dosing and follow guidance below, depending on outcome<br>• Resume dosing at one reduced dose or same dose level as clinically appropriate<br>• Discontinue study drug and observe patient until resolution |

NCI CTCAE v5, National Cancer Institute Common Terminology Criteria for Adverse Events Version 5.0.

#### 8.4.5.3 Dose modifications due to specific capivasertib-related toxicities

**Hyperglycaemia**

These are general recommendations therefore due consideration should be given to baseline values and fasting condition (and time since food if applicable) when interpreting glucose results. In diabetic patients, it may be beneficial to rule out concomitant aetiologies that could be associated with hyperglycaemia (eg, infections, dehydration, vascular events, glucocorticoids).

Patients should be made aware of symptoms of hyperglycaemia (eg, polydipsia and polyuria).

Dose modification guidelines for capivasertib-related hyperglycaemia are shown in Table 10. In addition, for all grades, patients should receive education on lifestyle changes (eg, a diabetic diet) and consider beginning home glucose monitoring (eg, fasting self-blood glucose monitoring [SBGM] once-daily) at the discretion of the investigator. If glucose home monitoring is instituted, the capivasertib/placebo treatment decision should be based on the morning fasting glucose value obtained prior to the dose of capivasertib/placebo.

It is recommended that approaches to the management of capivasertib-induced hyperglycaemia include advice from a diabetologist where appropriate (eg, diabetic patients). Metformin is currently the preferred oral antidiabetic recommended for the management of hyperglycaemia occurring in patients participating in studies of capivasertib (see below for further guidance). If a second agent is required, consideration should be given to the intermittent schedule of capivasertib and the pattern of glucose changes (eg, sulphonylureas should be avoided due to their risk of hypoglycaemia secondary to their mechanism of action).

**Table 10 Dose modifications for capivasertib-related hyperglycaemia[a]**

| NCI CTCAE v5 Toxicity Grade: | Actions |
|---|---|
| **Grade 1**<br>(Abnormal glucose above baseline with no medical intervention) | Maintain same capivasertib/placebo dose level |
| **Grade 2**<br>(Change in daily management from baseline for a diabetic; oral anti-glycaemic agent initiated; workup for diabetes) | Asymptomatic:<br>• Maintain same capivasertib/placebo dose level<br>• Treatment as per local guidelines, consider the use of oral antidiabetic (eg, metformin) on capivasertib/placebo dosing days only (see further guidance on choice of antidiabetic agents on text above and below the table)<br>Symptomatic: Appropriate clinical management as per local guidelines<br>• Interrupt capivasertib/placebo until resolution of symptoms and fasting blood glucose ≤160 mg/dL or ≤8.9 mmol/L (treatment can be interrupted up to 21 days)<br>• Restart at same dose level maintaining appropriate antidiabetic treatment (eg addition of/higher dose of oral metformin)<br>• Consider consult with the diabetologist |
| **Grade 3**<br>(Insulin therapy initiated; hospitalisation indicated) | Hold capivasertib/placebo up to 21 days until resolution of symptoms. Consult with diabetologist<br>• If fasting blood glucose decreases ≤160 mg/dL or ≤8.9 mmol/L within 21 days following appropriate antidiabetic treatment, resume capivasertib/placebo at 1 lower dose level<br>• If fasting blood glucose does not decrease to ≤160 mg/dL or ≤8.9 mmol/L within 21 days following appropriate antidiabetic treatment, permanently discontinue Capivasertib/placebo |
| **Grade 4**<br>(Life-threatening consequences; urgent intervention indicated) | • Appropriate clinical management as per local guidelines<br>• Consider consult with the diabetologist<br>• Consider permanent cessation of capivasertib/placebo |

[a] Patients may receive education on lifestyle changes (eg, a diabetic diet) and consider beginning home glucose monitoring (eg, fasting self-blood glucose monitoring [SBGM] once-daily) at the discretion of the investigator. If glucose home monitoring is instituted, the capivasertib/placebo treatment decision should be based on the morning fasting glucose value obtained prior to the dose of capivasertib/placebo.

NCI CTCAE v5, National Cancer Institute Common Terminology Criteria for Adverse Events Version 5.0; SBGM, self-blood glucose monitoring

*Use of metformin*

Metformin is currently the preferred oral antidiabetic recommended for the management of hyperglycaemia occurring in patients participating in studies of capivasertib. Investigators should exercise caution in the dosing and management of patients receiving the metformin and capivasertib/placebo combination and must be vigilant for signs of renal impairment and metformin toxicity, such as lactic acidosis and hypoglycaemia, namely: lethargy, hypotension, poor urine output, drowsiness, irritation, tachypnoea, sweating, diarrhoea, and vomiting.

Due to the potential interaction of metformin and capivasertib (caused by the inhibition of renal transporters [eg, OCT2] involved in the excretion of metformin), when taking both capivasertib and metformin concurrently, it is recommended weekly monitoring of creatinine after initiation of metformin, for up to 3 weeks and then on Day 1 of each cycle thereafter.

Metformin should only be given on the days when capivasertib/placebo is also administered (the half-life of capivasertib is approximately 7-15 hours) and should be withdrawn when treatment with capivasertib/placebo is withdrawn, unless otherwise clinically indicated.

Consider withholding of metformin on the days patients are due to have imaging with contrast (in order to reduce the already low risk of lactic acidosis) as per local guidelines.

### Maculo-papular rash

Dose modifications for capivasertib-related maculo-papular rash, which is the most frequent skin toxicity observed in patients treated with capivasertib, are provided in Table 11. However, these management guidelines can be used for other skin toxicities at the discretion of the investigator and/or following consultation with the dermatologist.

**Table 11 Dose modifications for capivasertib-related maculo-papular rash**

| NCI CTCAE v5 Toxicity Grade: | Actions: |
|---|---|
| **Grade 1 or 2** | Continue dosing at current dose and initiate dermatological treatment:<br>• Topical steroid of moderate strength twice-daily<br>• Non-sedating oral antihistamines |
| **Grade ≥3 or any grade clinically intolerable** | Withhold dosing for up to 28 days and initiate dermatological treatment (topical steroid of moderate strength and non-sedating oral antihistamines) with oral steroid for a short course (eg, up to 2 weeks). Consultation with dermatologist is advised |
| • Improves to grade ≤1 and tolerable within 28 days from onset | • Continue dermatological treatment[a] and restart dosing at same dose |
| • Improves to grade 2 and tolerable within 28 days from onset | • Continue dermatological treatment[a] and restart dosing at reduced dose (1 dose level reduction) |
| • Does not improve to grade 2 and tolerable within 28 days from onset | • Continue dermatological treatment[a] and discontinue capivasertib/placebo |
| **Recurrence of Grade 3, or Grade 4 (eg, severe bullous, blistering or exfoliating skin conditions), or any % BSA associated with extensive superinfection, with IV antibiotics indicated; life-threatening consequences** | Discontinue capivasertib/placebo |

[a] In patients with persistent rash or previous occurrence of grade 3 rash, consider secondary prophylaxis by continuing topical steroids and/or non-sedating oral antihistamines.

BSA, body surface area; NCI CTCAE v5, National Cancer Institute Common Terminology Criteria for Adverse Events Version 5.0.

### Hypersensitivity

In the case of hypersensitivity reactions, capivasertib/placebo should be discontinued and symptomatic/supportive therapy should be initiated (including with antihistamines and/or steroids) as considered appropriate by the investigator/treating physician. Drug re-challenge is not recommended; any subsequent consideration on re-challenge with capivasertib at the same or a lower dose, with its potential for recurrence of such or more severe events should be carefully considered against the potential benefits to the individual patient from continuation

of capivasertib therapy and agreed with the sponsor. Further management should follow local guidelines on management of hypersensitivity reactions.

**Diarrhoea**

Patients should be instructed to promptly contact investigators if they develop diarrhoea. **Alternative aetiologies should be ruled out prior to initiating the dose modifications.** Investigators are recommended to prescribe anti-diarrhoeal treatment at the first visit so that patients can start treatment at the first sign of diarrhoea, should it occur.

Loperamide is the preferred anti-diarrhoea agent for the management of diarrhoea occurring in patients participating in studies of capivasertib. Dose modifications for capivasertib-related diarrhoea are provided in Table 12.

If diarrhoea is reported, additional details regarding this AE will be collected in the eCRF.

**Table 12 Dose modifications for capivasertib-related diarrhoea**

| NCI CTCAE v5 Toxicity Grade: | Actions: |
|---|---|
| **Grade 1** | Maintain same capivasertib/placebo dose. Anti-diarrhoeal treatment (eg, loperamide) should be initiated at first report of diarrhoea. Maximise the supportive care (eg, dietary modifications, appropriate hydration therapy and electrolyte supplements as clinically indicated). |
| **Grade 2** | Interrupt capivasertib/placebo dose (up to 21 days) until recovery to grade ≤1 and resume dosing at same dose level. Anti-diarrhoeal (eg, loperamide) treatment should be initiated at first report of diarrhoea. Maximise the supportive care (eg, dietary modifications, appropriate hydration therapy and electrolyte supplements as clinically indicated).<br>Consider starting secondary prophylaxis[a]. |
| **Grade ≥3** | Interrupt capivasertib/placebo dose (up to 21 days) and institute appropriate anti-diarrhoeal treatment |
| • Improves to grade ≤1 within 21 days | • Resume dosing at same dose level or 1 reduced dose level as clinically appropriate, maintaining treatment for toxicity as necessary and/or start secondary prophylaxis[a] |
| • Does not improve to grade ≤1 after 21 days | • Discontinue capivasertib/placebo |
| **Recurrence of grade ≥2 or clinically significant or intolerable toxicity despite secondary prophylaxis** | Interrupt capivasertib/placebo dose (up to 21 days) maintaining appropriate anti-diarrhoeal treatment |
| • Improves to grade ≤1 or becomes clinically tolerable within 21 days | • Resume dosing up to 2 reduced dose levels maintaining treatment for toxicity as necessary and/or maintaining secondary prophylaxis[a] |
| • Does not improve to grade ≤1 or remains clinically significant/intolerable after 21 days | • Discontinue capivasertib/placebo |

[a] In patients with persistent grade 1 diarrhoea (eg, loperamide 2 mg, 2 to 4 times daily).

NCI CTCAE v5, National Cancer Institute Common Terminology Criteria for Adverse Events Version 5.0.

## 8.5 Pharmacokinetics

Blood samples of approximately 2 mL will be collected from each patient for measurement of plasma concentration of capivasertib as per the SoA (Section 1.1). Samples from patients dosed with placebo will not be analysed (for exceptions, see Section 8.5.1). The randomisation list will be supplied to the bioanalytics team before sample analysis.

Results will only be reported for samples shipped within a timeframe for which the stability of capivasertib in the samples has been validated and shown to be acceptable.

Samples may be collected at additional time points during the study if warranted and agreed upon between the investigator and the sponsor. Instructions for the collection and handling of biological samples will be provided by the sponsor or analytical test site. The actual date and time (24-hour clock time) of each sample will be recorded.

Samples will be used to evaluate the PK of capivasertib. Samples collected for analyses of capivasertib plasma concentration may also be used to evaluate safety or efficacy aspects related to concerns arising during or after the study.

Drug concentration information that may unblind the study will not be reported to investigative sites or blinded personnel until the study has been unblinded.

Incurred sample reproducibility analysis, if any, will be performed alongside the bioanalysis of the test samples. The results from the evaluation will not be reported in the CSR but separately in a bioanalytical report.

### 8.5.1 Determination of drug concentration

Samples for determination of capivasertib concentrations in plasma will be analysed by Covance on behalf of Clinical Bioanalysis Alliance, AstraZeneca R&D, using an appropriate bioanalytical method. Full details of the analytical method used will be described in a separate bioanalytical report.

For each placebo patient, samples will only be analysed on a 'for cause' basis, eg, if no quantifiable concentrations were observed in a patient's samples when the drug was expected to be present.

### 8.5.2 Storage and destruction of pharmacokinetic samples

#### 8.5.2.1 Samples collected in China

PK samples will be disposed of after the bioanalytical report finalisation or 6 months after issuance of the draft bioanalytical report (whichever is earlier).

#### 8.5.2.2 Samples collected outside of China

PK samples will be disposed of after the bioanalytical report finalisation or six months after issuance of the draft bioanalytical report (whichever is earlier), unless requested for future analyses.

PK samples may be disposed of or destroyed and anonymised by pooling. Additional analyses may be conducted on the anonymised, pooled pharmacokinetic samples to further evaluate and validate the analytical method. Any results from such analyses may be reported separately from the CSR.

## 8.6 Genetics

### 8.6.1 Optional exploratory genetic sample

A blood sample will be collected at screening for DNA isolation from patients who have consented to participate in the genetic analysis component of the study. Participation is optional. Patients who do not wish to participate in the genetic research may still participate in the study.

In the event that the original genetic sample cannot be analysed, a replacement genetic blood sample may be requested from the patient. Signed informed consent will be required to obtain a replacement sample unless it was included in the original consent.

See Appendix G for information regarding genetic research. Details on processes for collection and shipment and destruction of these samples can be found in Appendix G or in the Laboratory Manual.

This sample will not be collected in China.

### 8.6.2 Storage and destruction of genetic samples

The processes adopted for the coding and storage of samples for genetic analysis are important to maintain patient confidentiality. Samples may be stored for a maximum of 15 years or as per local regulations from the date of the Last Patient's Last Visit, after which they will be destroyed. DNA is a finite resource that may be used up during analyses. The results of any further analyses will be reported either in the CSR itself or as an addendum, or separately in a scientific report or publication.

No personal details identifying the individual will be available to AstraZeneca or designated organisations working with the DNA.

## 8.7 Biomarkers

Mandatory collection of tumour tissue and blood samples for biomarker research is required as a part of this study. Optional tumour tissue samples should be collected from consenting patients only. The following samples will be collected from all patients in this study as specified in the SoA (Section 1.1). See Appendix F for additional information on the handling of biological samples.

### 8.7.1 Tumour tissue sample

#### 8.7.1.1 Mandatory tumour tissue samples for biomarker analysis

All randomised patients must provide an archival or newly collected FFPE tumour sample (see Section 1.1) collected as part of routine clinical practice, for central testing. This should preferably be a tissue block to enable the analyses described below. If a block is not possible,

then 30 (minimum 20) freshly-cut, unstained, serial tumour slides from the most recently collected tumour tissue. For archival bone biopsies: samples arising from skeletal deposits are not accepted unless the sample has been taken from an associated soft tissue component and has not required or undergone decalcification. If tissue is inadequate for testing, the study site may collect a fresh sample before the start of treatment. Further details on tissue specifications are outlined in the Laboratory Manual and the Diagnostic Testing Manual. The primary purpose for mandating tumour tissue is determination of patient tumours' *PIK3CA*, *AKT1* and *PTEN* mutation status, to allow monitoring of the prevalence of the qualifying *PIK3CA/AKT1/PTEN* alterations to ensure an appropriate number of patients are recruited into the altered subgroup.

Additional exploratory analyses may be undertaken on the data generated from tumour tissue to identify other genomic biomarkers of sensitivity and resistance to treatment, and to increase our understanding of the disease.

Based on availability of tissue, further additional exploratory biomarkers may be evaluated, which may include but are not limited to, PTEN protein expression and immunological biomarkers such as PD-L1 expression and tumour infiltrating lymphocyte, and gene-expression based tumour subtyping.

Samples collected in China will not be used for additional exploratory biomarker studies, beyond the initial tumour profiling.

#### 8.7.1.2 Optional tumour tissue samples for exploratory biomarker research

Patients will sign an informed consent for the optional tumour tissue biopsies. Where possible, every effort should be made to collect optional samples for biomarker research from consenting patients in the study, as outlined in the SoA (see Section 1.1) and as follows:

- Paired biopsies: a baseline biopsy taken before the initial dosing of study drug at screening or pre-dose on Cycle 1, Week 1, Day 1, and one taken on-treatment biopsy. The on-treatment- biopsy should be taken at least 4 hours post-dose between Cycle 1, Week 3, Day 2 and Cycle 1, Week 3, Day 4. Biopsies should preferably be collected from the same tumour site. The exact day, time and site of biopsy collection should be clearly noted in the associated documentation.
- An additional biopsy obtained at termination of treatment/disease progression, if applicable.

The tumour biopsy procedure will be performed by core needle, under radiological guidance, or surgically if the site of disease is superficial and palpable or visible. It is mandated that the core biopsy be removed directly from the tumour in situ and not cored from a surgically removed tumour. This is to ensure the best possible quality of the biopsy, so the blood/nutrient supply to the tumour is not disrupted prior to biopsy collection. Collection of tumour cells from fluid such as ascites or pleural effusion is not permitted.

The optional tumour samples may be analysed using the same central test as used for the mandatory tumour tissue samples and for biomarkers including, but not limited to, markers of AKT inhibition such as phosphorylated GSK3β, phosphorylated PRAS40, FOXO3 and treatment-induced changes in immunological markers such as CD8 expressing T-cells and PD-L1 expression.

These samples will not be collected in China.

#### 8.7.1.3 Collection, analysis and reporting of tumour samples

Mandatory tumour samples will be collected as detailed in the Laboratory Manual and the Diagnostic Testing Manual. Patients must consent to provide an archival or newly FFPE tumour sample, collected as part of routine clinical practice, for analysis.

Mandatory tumour samples meeting the minimum tumour content and tissue volume, as specified in the Laboratory Manual and Diagnostic Testing Manual, will be tested with the primary intent to detect tumour DNA alterations in the following genes; *AKT1*, *PIK3CA*, and *PTEN*. If the first sample submitted for testing is inconclusive due to technical test failure, a further sample may be submitted for testing. Submission and testing of additional samples can only be performed if the original testing failed due to technical reasons (ie, failed test result). Please refer to the Laboratory Manual and the Diagnostic Testing Manual for further details regarding retesting procedures. Central testing may generate results on genes other than *AKT1, PIK3CA,* and *PTEN*, and the results may be provided at the time of treatment discontinuation upon individual request by the investigator in order to avoid bias during the treatment period.

Optional tumour biopsy samples will be collected from consenting patients only. The optional tumour biopsies collected at disease progression meeting the minimum tumour content and tissue volume, as specified in the Laboratory Manual and Pathology and Genomic Testing Manual, will be tested centrally by NGS using the same central test as described above for the mandatory tumour samples. The primary intent is to detect changes in the tumour DNA alterations that may be associated with treatment resistance. The results of this central testing of the optional tumour biopsy obtained at disease progression will be provided to the investigator.

Test results will be reported to AstraZeneca.

### 8.7.2 Mandatory blood samples for exploratory biomarker analysis

Blood samples will be collected for analysis of ctDNA (see Section 1.1). ctDNA samples collected at screening will be analysed for predictive biomarkers of response to treatment and may be used to develop and validate future in vitro diagnostic (IVD) tests to identify patients most likely to response to the treatment. The sample is requested prior to treatment in order to

maximise the probability of detecting ctDNA where the tumour burden is relatively high. Several ctDNA samples will be taken during treatment and a final sample will be taken at disease progression. These samples will be used for additional exploratory research which may include but is not limited to interrogation of changes in genetic alterations as well as the dynamics of the biomarkers on treatment and potential mechanisms of resistance to treatment.

For exploratory biomarker research, blood samples will be collected to provide plasma for circulating soluble factors and whole blood samples will be collected for DNA and RNA biomarkers. These samples will be taken at multiple timepoints and analysed for a range of oncology and immunological biomarkers that may correlate with drug response. These biomarkers may include but are not limited to panels of cytokines, chemokines and other soluble biomarkers; T-cell receptor repertoire analysis and analysis of gene expression biomarkers associated with immunomodulatory effects.

Details of sample collection, processing, shipping and storage are detailed in the Laboratory Manual.

These samples will not be collected in China.

### 8.7.3 Storage, re-use and destruction of biomarker samples

Biological samples will be stored for a maximum of 15 years from the date of the Last Patient's Last Visit, after which they will be destroyed. The results of this biomarker research will be reported either in the CSR itself or as an addendum, or separately in a scientific report or publication. The results of this biomarker research may be pooled with biomarker data from other studies to generate hypotheses to be tested in future research.

Any unused or partially used tumour samples collected in China for the purpose of *PIK3CA/AKT1/PTEN*-alternation assessment will be destroyed or repatriated maximally 5 years after the study drug is approved for marketing in China.

## 8.8 Healthcare resource use

The impact of treatment and disease on healthcare resource use will be captured in this study on an event-driven basis.

The Hospital Admission (HOSPAD) module will be used to collect information on key health care resource use beyond study mandated visits. The site should complete the HOSPAD form at the site at every scheduled clinic visit up to and including the post-study treatment discontinuation follow-up visit. If a patient discontinues study treatment for reasons other than RECIST v1.1 progression, the HOSPAD form should continue to be administered until progression has been confirmed. Study mandated visits should not be included as a hospital admission.

The data may be used as input to cost analyses for example cost utility analysis or cost effectiveness analysis.

# 9 STATISTICAL CONSIDERATIONS

## 9.1 Statistical hypotheses

The null hypotheses for the primary time to event endpoint (PFS) are that there is no difference between capivasertib + fulvestrant and placebo + fulvestrant in the probability of a progression event in the overall population and the *PIK3CA/AKT1/PTEN*-altered population. The intention of the study is to demonstrate the superiority of capivasertib + fulvestrant over placebo + fulvestrant in either or both of the overall and *PIK3CA/AKT1/PTEN*-altered populations.

## 9.2 Sample size determination

Assuming a significance level of 5%, a total of 492 OS events will be required to achieve 90% power to detect a treatment effect of an average HR 0.74 in the overall population, assuming a 12-month delay to a treatment effect and an HR of 0.64 after the delay. Assuming 70% maturity at the time of the final analysis, approximately 700 patients will need to be randomised. Of these 700 randomised patients, it is expected that a minimum of 280 patients will have a tumour harbouring an eligible *PIK3CA/AKT1/PTEN* alteration based on a prevalence of ~40-45% (Cristofanilli et al 2016, Curtis et al 2012, Di Leo et al 2018, Hortobagyi et al 2016, Pereira et al 2016, Spoerke et al 2016), and a minimum of approximately 224 patients will be in the *PIK3CA/AKT1/PTEN*-altered subgroup assuming a test failure rate of 20%.

The primary objective of this study is to assess the efficacy of capivasertib + fulvestrant vs placebo + fulvestrant by assessment of PFS in the overall and the *PIK3CA/AKT1/PTEN*-altered populations. The PFS Primary Analysis will take place after PFS reaches approximately 77% maturity (542 events) in the overall population, and approximately 77% of PFS events have occurred in patients whose tumours harbour an eligible *PIK3CA/AKT1/PTEN* alteration, based on a prevalence of ~40-45% (and 174 events will have been observed if a test failure rate is 20%).

After all the pre-defined PFS endpoints have been tested, the remaining alpha will be used for testing OS in the *PIK3CA/AKT1/PTEN*-altered population and subsequently for OS in the in overall population. ORR in the *PIK3CA/AKT1/PTEN*-altered population and the overall population will be tested sequentially only if OS in the overall population is statistically significant.

The OS Interim Analysis is expected to occur when approximately 394 OS events have been observed in the overall population and similar maturity has been reach in the altered

population (56% maturity, 80% information fraction). If the time between the PFS analysis and the OS interim analysis is approximately 3 months or less, then the PFS DCO may be delayed and the analyses combined. The OS Final Analysis will take place when approximately 70% maturity has been observed in both the overall and the *PIK3CA/AKT1/PTEN*-altered populations. The exact significance level will be determined according to the O'Brien & Fleming method (Lan and DeMets 1983) based on the actual number of events observed at the OS Interim Analysis.

**Table 13** and **Table 14** show the critical values at various significance levels for PFS and OS testing.

**Table 13 Statistical considerations for dual primary PFS endpoints**

| **Analysis** | **Significance level** | **Number of events (assuming 20% test failure rate)** | **Critical value in HR [corresponding median PFS[a]]** | **Power (Assuming HR=0.64)** |
|---|---|---|---|---|
| PFS Analysis (altered subgroup, assuming 77% maturity) | 0.021[b] | 217 (174) | 0.73 (7.5 months) [0.71 (7.7 months)] | 83.7% (73.9%) |
| | 0.05[c] | 217 (174) | 0.77 (7.2 months) [0.74 (7.4 months)] | 90.8% (83.7%) |
| PFS Analysis (overall population, assuming 77% maturity) | 0.035 | 542 | 0.83 (6.6 months) | >99% |

HR, hazard ratio; PFS, progression-free survival.

[a] Assuming median PFS for placebo + fulvestrant arm is 5.5 months and PFS is exponentially distributed.

[b] Assuming the null hypothesis for the PFS Analysis in the overall population is not rejected at α=0.035 level and the observed ratio of the number of events between the altered subgroup and overall population is 0.4.

[c] Assuming the null hypothesis for the PFS Analysis in the overall population is rejected at α=0.035 level.

## Table 13 Statistical considerations for OS endpoint

| Analysis | Significance level | Number of events (assuming 20% test failure rate) | Critical value in HR [corresponding median OS[a]] | Power (Assuming HR=0.74) |
|---|---|---|---|---|
| OS Interim Analysis (altered subgroup, assuming 80% information fraction) | 0.0056[b] | 158 (127) | 0.642 (33.7 months) [0.610 (40.9 months)] | 13% (6%) |
| | 0.0244[c] | 158 (127) | 0.698 (30.8 months) [0.670 (35.2 months)] | 28% (16%) |
| OS Final Analysis (altered subgroup) | 0.0133[b] | 197 (158) | 0.702 (29.3 months) [0.673 (32.0 months)] | 36% (21%) |
| | 0.0429[c] | 197 (158) | 0.749 (27.9 months) [0.724 (29.7 months)] | 53% (36%) |
| OS Interim Analysis (overall population, assuming 80% information fraction) | 0.0056[b] | 394 | 0.756 (28.5 months) | 44% |
| | 0.0244[c] | 394 | 0.796 (27.2 months) | 64% |
| OS Final Analysis (overall population) | 0.0133[b] | 492 | 0.799 (26.5 months) | 80% |
| | 0.0429[c] | 492 | 0.833 (25.8 months) | 90% |

HR, hazard ratio; OS, overall survival.

[a] Assuming median OS for placebo + fulvestrant arm is 23 months and OS follows a Weibull distribution with $\beta$=1.5.

[b] Assuming the null hypothesis for the PFS Analysis in the overall population is not rejected at $\alpha = 0.035$ level.

[c] Assuming the null hypothesis for the PFS Analysis in the overall population is rejected at $\alpha = 0.035$ level.

## 9.3 Populations for analyses

For purposes of analyses, the populations described in Table 14 are defined.

**Table 14 Analysis populations**

| Population | Description |
|---|---|
| Full Analysis Set (FAS) | The FAS will be used as the primary population for reporting efficacy data and to summarise baseline characteristics.<br>This comprises all patients randomised into the study, excluding patients randomised in China after the global cohort last patient first visit (LPFV), and will be analysed according to randomised treatment regardless of the treatment received (intention-to-treat [ITT] principle). Any important deviations from randomised treatment will be listed and considered when interpreting the efficacy data. |
| Safety Analysis Set | The safety analysis set comprises all patients included in the FAS, who received at least 1 dose of study drug (fulvestrant, capivasertib, placebo) and will be analysed according to the treatment received. If a patient receives at least 1 dose of capivasertib, they will be summarised in the capivasertib arm for safety summaries (eg, capivasertib arm will include patients randomised to capivasertib who receive at least 1 dose of capivasertib, or placebo patients who receive at least 1 dose of capivasertib in error at any time). If a patient randomised to capivasertib receives only placebo treatment, then they will be summarised as part of the placebo arm. Patients who receive only fulvestrant will also be included in the safety analysis set and will be included in the treatment arm to which they were randomised. |
| Altered Subgroup FAS | This comprises all patients included in the FAS with a *PIK3CA/AKT1/PTEN*-altered result from a valid[a] biomarker test as determined by central testing. |
| Altered Subgroup Safety Analysis Set | This comprises all patients included in the Safety Analysis Set with a *PIK3CA/AKT1/PTEN*-altered tumour as determined by central testing. |
| Pharmacokinetic (PK) Analysis Set | This comprises all patients included in the FAS who received at least 1 dose of capivasertib with at least one reportable concentration. |

[a] The definition of a valid biomarker test will be pre-specified in the SAP prior to any efficacy analysis.

## 9.4 Statistical analyses

Analyses will be performed by AstraZeneca or its representative. A comprehensive SAP will be developed and finalised before the primary PFS analysis database lock and will describe the patient populations to be included in the analyses, and procedures for accounting for missing, unused, and spurious data. This section is a summary of the planned statistical analyses of the primary and secondary endpoints. Any deviations from this plan will be reported in the CSR.

### 9.4.1 Efficacy analyses

Efficacy analyses will be performed based on the Full Analysis Set (FAS) (intention-to-treat [ITT]) for the overall population and on the Altered Subgroup FAS for the *PIK3CA/AKT1/PTEN*-altered subgroup.

Depending on the extent of any impact, summaries of data relating to patients diagnosed with COVID-19, and impact of COVID-19 on study conduct (in particular missed visits, delayed or discontinued IP, and other protocol deviations) may be generated. More details will be provided in the SAP.

#### 9.4.1.1 Primary endpoint: PFS in the overall population

PFS is defined in Section 8.1.1.1 together with the details of censoring.

PFS in the overall population will be analysed using a stratified log-rank test adjusting for the stratification factors (Section 4.1).

The stratification variables in the statistical modelling will be based on the values entered into IVRS at randomisation. If there are insufficient events per strata, the strata will be pooled following a pooling strategy that will be prespecified in the SAP.

From the stratified Cox proportional hazards model (ties = Efron) the HR (capivasertib + fulvestrant vs placebo + fulvestrant) together with its corresponding 95% CI (calculated using a profile likelihood approach) will be presented. An HR less than 1 will favour capivasertib + fulvestrant.

Subgroup analyses will be conducted to assess PFS by the stratification factors (see Section 4.1) and the following (but not limited to) subgroups of the FAS:

- Age at randomisation (<65 vs ≥65 years of age)
- *PIK3CA/AKT1/PTEN* mutation status in tissue (*PIK3CA/AKT1/PTEN*-altered vs confirmed *PIK3CA/AKT1/PTEN*-non-altered + unknown)

Additional subgroup analyses (eg, menopausal status, type of endocrine resistance) may be conducted and the details outlined in the SAP.

Other baseline variables may also be assessed if there is clinical justification or an imbalance is observed between the treatment arms. The purpose of the subgroup analyses is to assess the consistency of treatment effect across expected prognostic and/or predictive factors. Forest plots will be presented.

No adjustment to the significance level for testing of the subgroup and sensitivity analyses will be made, since all these analyses will be considered supportive of the analysis of PFS.

For each subgroup level of a factor, the HRs and associated CIs will be calculated from a Cox proportional hazards model (ties = Efron) that only contains a term for treatment. The Cox models will be fitted using SAS® PROC PHREG with the Efron method to control for ties and using a BY statement for the subgroup factor. The HRs and 95% CIs will be presented on a forest plot.

Further sensitivity analyses will be described in the SAP.

**Supplementary analysis**

PFS defined by BICR in the overall population will be analysed as described for the investigator-assessed PFS endpoint (see above).

#### 9.4.1.2 Primary endpoint: PFS in the *PIK3CA/AKT1/PTEN*-altered subgroup

PFS in the *PIK3CA/AKT1/PTEN*-altered subgroup will be analysed as described for the investigator-assessed PFS endpoint (Section 9.4.1.1) and stratified by the stratification factors (Section 4.1).

Additional sensitivity and subgroup analyses may be performed (including outcomes based on BICR).

#### 9.4.1.3 Secondary endpoint: OS in the overall population

OS is defined in Section 8.1.2 together with details of censoring.

OS will be analysed as described for the investigator-assessed PFS endpoint (Section 9.4.1.1), including subgroup analyses, and stratified by the stratification factors (Section 4.1).

#### 9.4.1.4 Secondary endpoint: OS in the *PIK3CA/AKT1/PTEN*-altered subgroup

OS in the *PIK3CA/AKT1/PTEN*-altered subgroup will be analysed as described for the investigator-assessed PFS endpoint (Section 9.4.1.1) and stratified by the stratification factors (Section 4.1).

Additional sensitivity and subgroup analyses may be performed.

#### 9.4.1.5 Secondary endpoint: PFS2 in the overall and altered FAS population

PFS2 is defined in Section 8.1.3 together with details of censoring.

PFS2 in the overall and in the *PIK3CA/AKT/PTEN*-altered populations will be analysed as described for the investigator-assessed PFS endpoint (Section 9.4.1.1) and stratified by the stratification factors (Section 4.1).

#### 9.4.1.6 Secondary endpoint: ORR in the overall and altered FAS population

The ORR will be compared between capivasertib + fulvestrant vs placebo + fulvestrant, for both the overall and the *PIK3CA/AKT/PTEN*-altered populations, using logistic regression models adjusting for the stratification factors (Section 4.1). The results of the analysis will be presented in terms of an odds ratio (an odds ratio greater than 1 will favour capivasertib + fulvestrant) together with its associated profile likelihood 95% CI and p-value. If there are not enough responses for a meaningful analysis using logistic regression, then a Cochran-Mantel Haenszel (CMH) test will be presented. The CMH test will be stratified using the same stratification factors as the dual primary endpoints.

ORR will be presented with a two-sided 95% CIs using the Clopper-Pearson method (Clopper and Pearson 1934).

#### 9.4.1.7 Secondary endpoint: DoR in the overall and altered FAS population

DoR is defined in Section 8.1.1.3 together with details of censoring.

Descriptive data will be provided for the DoR in responding patients, including the associated Kaplan-Meier (KM) plots for both the overall and the *PIK3CA/AKT1/PTEN*-altered populations.

#### 9.4.1.8 Secondary endpoint: CBR in the overall and altered FAS population

CBR is defined in Section 8.1.1.2.

CBR in the overall and the *PIK3CA/AKT1/PTEN*-altered population will be analysed as described for the ORR analysis (Section 9.4.1.6).

#### 9.4.1.9 Secondary endpoint: PK

The plasma concentration-time data will be analysed by population PK methods using non-linear mixed-effects modelling. PK parameters, including variability parameters, will be estimated as data permits. The influence of intrinsic (eg, ethnicity, gender, age, weight, renal function and hepatic function) and extrinsic (eg, concomitant medication) factors will be evaluated and exposure-response relationships will be explored. Details will be outlined in a separate modelling analysis plan and results may be reported separately.

In the subgroup of approximately 6 Japanese patients with rich capivasertib PK data, the area under the plasma concentration-time curve from zero to 12 hours ($AUC_{0-12h}$), the maximum observed plasma (peak) concentration ($C_{max}$), and the time to reach peak or maximum observed concentration following drug administration ($t_{max}$) will be derived.

PK analyses will be performed based on the PK Analysis Set and any exclusion of data will be documented and justified.

Data from this study may be pooled with data from other studies.

#### 9.4.1.10 Secondary endpoints: EORTC QLQ-C30 and EORTC QLQ-BR23

The EORTC QLQ-C30 and EORTC QLQ-BR23 will be scored according to the EORTC Scoring Manual (Fayers et al 2011). An outcome variable consisting of a score from 0 to 100 will be derived for each of the symptom scales, each of the functional scales, and the global measure of health status scale according to the EORTC Scoring Manual. Higher scores on the global measure of health status and functional scales indicate better health status/function, but higher scores on symptom scales represent greater symptom severity. For each subscale, if <50% of the subscale items are missing, then the subscale score will be divided by the number of non-missing items and multiplied by the total number of items on the subscales (Fayers et al 2011). If at least 50% of the items are missing, then that subscale will be treated as missing. Missing single items are treated as missing. The reason for any missing questionnaire will be identified and recorded.

The main PRO measures identified in the secondary objectives are global health status/QoL, physical function, role function, fatigue, pain and appetite loss subscales of the EORTC QLQ-C30. However, separate analysis will be conducted for each EORTC QLQ-C30 and EORTC QLQ-BR23 scale/symptom score.

The primary assessment of global health status/QoL, physical function, role function, or symptoms will focus on comparing mean change from baseline between treatment arms. To examine the change from baseline and time to deterioration, the analysis population will be a modified FAS (ITT) which includes all randomised patients with an evaluable baseline assessment and at least one evaluable post-baseline assessment. Similarly, analyses may be repeated in a modified *PIK3CA/AKT1/PTEN*-altered population, which includes patients whose tumours harbour a qualifying *PIK3CA/AKT1/PTEN*-alteration and have an evaluable baseline assessment and at least one evaluable post-baseline assessment, where appropriate. Change from baseline will be analysed using a mixed model repeated measurements analysis of all the post-baseline scores. The model will include treatment arm, visit, and treatment by visit interaction and stratification factors as explanatory variables, and the baseline score and baseline score by visit as covariates. Adjusted mean change from baseline estimates per

treatment arm and corresponding 95% CIs will be presented, along with an overall estimate of the treatment difference, 95% CI, and p-value.

Time to deterioration will be analysed using the same approach as described for the PFS and OS endpoints (Section 9.4.1.1). Separate analyses will be conducted for global health status/QoL, physical function, role function, fatigue, pain and appetite loss. The effect of treatment arms will be estimated by the HR together with its corresponding 95% CI and p-value. KM plots will be presented by treatment arm. Summaries of the number and percentage of patients who have an event as well as who were censored will be provided along with the median time to deterioration for each treatment arm. The HR, p-value, and 95% CI estimates will be presented.

Summary tables of responses for each EORTC QLQ-C30 and EORTC QLQ-BR23 scale/item score (global health status/QoL, 5 functions, symptom scales) for each assessment (improvement, deterioration, and no change) will be presented by treatment arm.

Finally, summaries of absolute and unadjusted change from baseline values of each EORTC QLQ-C30 and EORTC QLQ-BR23 scale/item score will be reported by assessment timepoint for each treatment arm. Graphical presentations may also be produced as appropriate.

Full details of the statistical analyses and appropriate sensitivity analyses will be described in full in the SAP.

#### 9.4.1.11 Secondary endpoint: time to definitive deterioration of the ECOG performance status in the overall population and the *PIK3CA/AKT1/PTEN*-altered population

Time to definitive deterioration of the ECOG performance status is defined in Section 8.1.4 together with details of censoring.

Time to definitive deterioration of the ECOG performance status in the overall and the *PIK3CA/AKT1/PTEN*-altered populations will be analysed as described for the investigator-assessed PFS endpoint (Section 9.4.1.1) and stratified by the stratification factors (Section 4.1).

### 9.4.2 Safety analyses

Safety analyses will be performed based on the Safety Analysis Set for the overall population and key safety results will be presented for the Altered Subgroup Safety Analysis Set for the *PIK3CA/AKT1/PTEN*-altered subgroup.

Depending on the extent of any impact, summaries of data relating to patients diagnosed with COVID-19, and impact of COVID-19 on study conduct (in particular missed visits, delayed or

discontinued IP, and other protocol deviations) may be generated. More details will be provided in the SAP.

#### 9.4.2.1 Adverse events

Adverse events will be coded using the most recent version of the Medical Dictionary for Regulatory Activities (MedDRA) that will have been released for execution at AZ/designee.

Safety data will be presented using descriptive statistics unless otherwise specified.

AEs will be presented for each treatment group by SOC and PT covering number and percentage of patients reporting at least one event and number of events where appropriate.

AEs occurring prior to start of IMP, treatment emergent AEs and post-treatment AEs will be presented separately.

An overview of AEs will present for each treatment group the number and percentage of patients with any AE, AEs with outcome of death, serious AEs, and AEs leading to discontinuation of IMP, as well as AEs leading to IMP dose interruptions and AEs leading to IMP dose reductions.

Separate AE tables will be provided taken into consideration relationship as assessed by the investigator, CTCAE grade, seriousness, death and events leading to discontinuation of IMP as well as other action taken related to IMP, events of special interest and other significant adverse events.

An additional table will present number and percentage of patients with most common AEs.

In accordance with the requirements of the FDA, a separate table will present non-serious AEs occurring in more than 5% of patients in any treatment group.

Key patient information will be presented for patients with AEs with outcome of death, serious AEs, and AEs leading to discontinuation of IMP.

An AE listing for the safety analysis set will cover details for each individual AE.

Full details of AE analyses will be provided in the SAP.

Treatment emergent

The following events are considered treatment emergent:

- Adverse events with an onset date on or after first dose of IMP
- Worsening of pre-existing events on or after first dose of IMP

#### 9.4.2.2 Other safety analysis

Other safety data including physical examinations, clinical haematology, chemistry, urinalysis, vital signs and ECGs will be summarized using descriptive statistics. The analysis will be performed based on the Safety Analysis Set for the overall population and on the Altered Subgroup Safety Analysis Set for the *PIK3CA/AKT1/PTEN*-altered subgroup, as defined in the SAP.

### 9.4.3 Other analyses

Exploratory analyses will be described in an appropriate SAP before the analyses take place.

### 9.4.4 Methods for multiplicity control

To control the family-wise error rate in the strong sense at 5% for the treatment comparisons in OS and PFS, a predefined MTP with an alpha-exhaustive recycling strategy (Burman et al 2009) taking into account intrinsic correlation between test statistics (Spiessens and Debois 2010), will be applied. The MTP is outlined in Figure 4. According to alpha (test mass) splitting and alpha recycling, if the higher-level hypothesis in the MTP is rejected for superiority, then the next lower level hypothesis will be tested. The test mass that becomes available after each rejected hypothesis is recycled to lower level hypotheses not yet rejected.

Tests will be grouped into 2 main families: one for the comparisons in PFS, the other for the comparisons in OS. Within the PFS and OS families, there are 2 sets of tests, one in the overall population, the other in the altered subgroup. The PFS family will be tested first, the OS family will be tested later (Figure 4). This testing procedure stops when the entire test mass is allocated to non-rejected hypotheses. Implementation of this predefined testing procedure, including recycling, will control the family wise type I error in the strong sense at 5% (2-sided), among all key hypotheses.

**Figure 4 Illustration of data cut-offs and associated treatment comparisons**

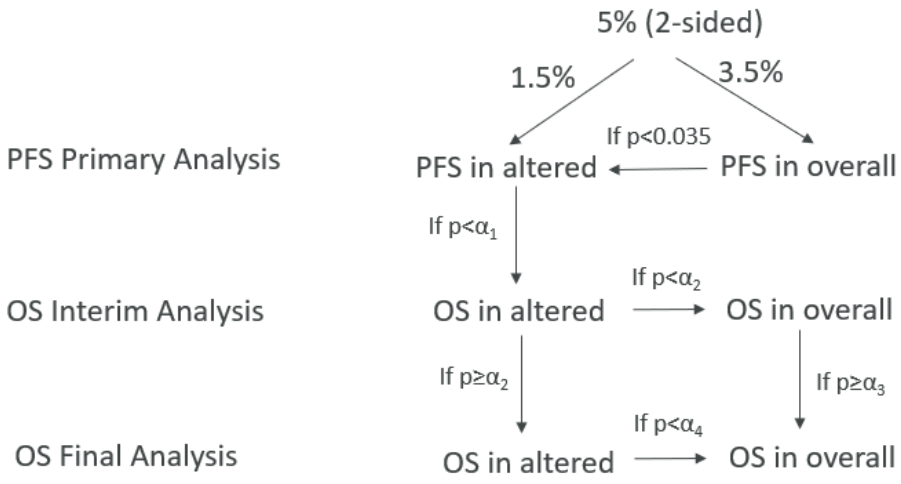

The significance level of $\alpha_1$ at PFS primary analysis in the altered subgroup will be determined as follows:

- If the p-value for PFS in the overall population is significant at 3.5% level, then the α level of 0.035 tested for the overall population will be recycled, making $\alpha_1$=0.05.
- If the p-value for PFS in the overall population is not significant at 3.5% level, then the $\alpha_1$ is determined by the observed ratio of #events in the altered subgroup and overall population using Spiessens and Debois method.

The significance level of $\alpha_2$ at OS interim analysis and $\alpha_4$ at OS final analysis for the altered subgroup will be determined by the observed information fraction (O'Brien & Fleming approach [Lan and DeMets 1983]) based on the remaining α available:

- If the p-value for PFS in the overall population is significant at 3.5% level, then the remaining α=0.05
- If the p-value for PFS in the overall population is not significant at 3.5% level, then the remaining α=0.015.

Similarly, if the p-value for OS in the altered subgroup is significant at $\alpha_2$ level at the OS interim analysis, OS in the overall population will be tested using the O'Brien & Fleming approach [Lan and DeMets 1983] based on the remaining α available. If the p-value for OS in the altered subgroup is significant at $\alpha_4$ level at the OS final analysis, all remaining α available after PFS primary analysis will be used to test OS in the overall population, i.e. either 1.5% or 5% based on the outcome of PFS hypothesis testing in the overall population.

If the OS endpoint is successful at either interim or final analysis in both the overall population and *PIK3CA/AKT1/PTEN*-altered subgroup, the remaining alpha will be recycled to test ORR in the *PIK3CA/AKT1/PTEN*-altered subgroup. If significant, the remaining alpha will be recycled to test ORR in the overall population. Data at PFS primary analysis DCO will be used to test ORR.

## 9.5 Interim analyses

The OS Interim Analysis is expected to occur when approximately 394 OS events have been observed in the overall population and similar maturity has been reached in the *PIK3CA/AKT1/PTEN*-altered population (56% maturity, 80% information fraction). The significance level for OS will be based on the significance level available after the PFS primary analysis and will be determined according to the O'Brien and Fleming method (Lan and DeMets 1983).

The Statistical Analysis Plan will describe the planned interim analysis in greater detail.

### 9.5.1 Independent data monitoring committee (IDMC)

An IDMC will be established in order to assess the progress of the clinical study at regular intervals prior to the PFS primary analysis database lock. This will include reviewing unblinded safety and tolerability data.

Appendix C provides more details on the rationale for and the remit of the committee.

## 9.6 China cohort

The global cohort will enrol approximately 930 patients to randomise approximately 700 patients. The China cohort will consist of approximately 134 randomised patients from National Medical Product Administration (NMPA)-certified sites. The global cohort will consist of patients recruited by the documented date of LPFV of the global cohort. In the

event that recruitment of China cohort is not completed during global recruitment of approximately 700 patients, the recruitment of China cohort will continue until approximately 134 Chinese patients have been randomised from NMPA-certified sites. Patients randomised in the China cohort prior to the LPFV of the global cohort enrolment will be included in both the FAS and the China FAS. A patient randomised in the China cohort after the LPFV of the global cohort enrolment will be included only in the China FAS.

Per NMPA guidance, in addition to the evaluation of the global cohort data for primary, secondary and safety objectives, evaluation of consistency in efficacy and safety in Chinese populations is required to facilitate the benefit-risk assessment for Chinese patients. Hence, the safety and efficacy data in the China cohort will be analysed separately where the same endpoint definitions (as described in Section 8.1) and the same analysis methods (as detailed in Section 9.4) are applied.

The China FAS will include all patients randomised in the China cohort including those who were recruited prior to the closure of the global cohort and are therefore included in the analyses of efficacy and safety for the main study. The China altered subgroup FAS will include all patients in the China FAS with a *PIK3CA/AKT1/PTEN*-altered result from a valid biomarker test as determined by central testing. Both China FAS and China altered subgroup FAS will be used for China-only efficacy analyses.

The China safety analysis set will consist of all patients included in the China FAS who received at least 1 dose of study treatment. The China altered subgroup safety analysis set will consist of all patients included in the China safety analysis set with a *PIK3CA/AKT1/PTEN*-altered result from a valid biomarker test as determined by central testing.

Efficacy analyses for the China cohort will be performed when the PFS and/or OS data from the patients from the China cohort are of similar maturity at which significant clinical efficacy is established in the global cohort, eg, if OS efficacy is established at the OS Interim Analysis, a similar maturity will be used for the consistency evaluation.

All statistical analyses will be considered exploratory and only performed if sufficient numbers of events or patients are available (eg, ≥20 OS or PFS events) unless specified, otherwise, descriptive statistics only will be presented. No adjustment for multiplicity will be made and the procedure for hierarchical testing detailed in Section 9.4 will not be followed.

Details of the China cohort analyses will be specified in the China SAP, which is to be finalised before the global cohort database lock for the PFS primary analysis.

# 10 REFERENCES

**Aaronson et al 1993**
Aaronson NK, Ahmedzai S, Bergman B, Bullinger M, Cull A, Duez NJ, et al. The European Organization for Research and Treatment of Cancer QLQ-C30: a quality-of-life instrument for use in international clinical trials in oncology. J Natl Cancer Inst 1993;85:365–76.

**André et al 2019**
André F, Ciruelos E, Rubovszky G, Campone M, Loibl S, Rugo HS, et al. Alpelisib for *PIK3CA*-Mutated, Hormone Receptor–Positive Advanced Breast Cancer. New Engl J Med 2019;380:1929-40.

**Banerji et al 2018**
Banerji U, Dean E, Perez-Fidalgo JA, Batist G, Bedard PL, You B, et al. A Phase 1 open-label study to identify a dosing regimen of the pan-AKT inhibitor AZD5363 for evaluation in solid tumors and in *PIK3CA*-mutated breast and gynecologic cancers. Clin Cancer Res 2018;24:2050–59.

**Brown and Banerji 2017**
Brown JS, Banerji U. Maximising the potential of AKT inhibitors as anti-cancer treatments. Pharmacol Ther 2017;172:101–115.

**Burman et al 2009**
Burman CF, Sonesson C, Guilbaud O. A recycling framework for the construction of Bonferroni-based multiple tests. Stat Med 2009;28:739–61.

**Cardoso et al 2017**
Cardoso F, Kataja V, Tjan-Heijnen (eds.). Breast Cancer: Essentials for Clinicians, 2017, ESMO.

**Cardoso et al 2018**
Cardoso F, Senkus E, Costa A, Papadopoulos E, Aapro M, Andre F, et al. 4th ESO–ESMO International Consensus Guidelines for Advanced Breast Cancer (ABC 4). Ann Oncol 2018;29:1634–57.

**Carr et al 2016**
Carr TH, McEwen R, Dougherty B, Johnson JH, Dry JR, Lai Z, et al. Defining actionable mutations for oncology therapeutic development. Nat Rev Cancer 2016 Apr 26;16(5):319-29.

**Clopper and Pearson 1934**
Clopper CJ, Pearson ES. The Use of Confidence or Fiducial Limits Illustrated in the Case of the Binomial. Biometrika 1934;26:404–413.

**Cristofanilli et al 2016**
Cristofanilli M, Turner NC, Bondarenko I, Ro J, Im SA, Masuda N, et al. Fulvestrant plus palbociclib versus fulvestrant plus placebo for treatment of hormone-receptor-positive, HER2-negative metastatic breast cancer that progressed on previous endocrine therapy (PALOMA-3): final analysis of the multicentre, double-blind, phase 3 randomised controlled trial. Lancet Oncol 2016;17:425-39.

**Cristofanilli et al 2018**
Cristofanilli M, Slamon DJ, Ro J, Bondarenko I, Im S, Masuda N, et al. Overall survival (OS) with palbociclib plus fulvestrant in women with hormone receptor-positive (HR+), human epidermal growth factor receptor 2-negative (HER2−) advanced breast cancer (ABC): Analyses from PALOMA-3. Ann Oncol 2018;9(Suppl 8). LBA2_PR. Available at URL: https://academic.oup.com/annonc/article/29/suppl_8/mdy424.009/5141510.

**Curtis et al 2012**
Curtis C, Shah SP, Chin SF, Turashvili G, Rueda OM, Dunning MJ, et al. The genomic and transcriptomic architecture of 2,000 breast tumours reveals novel subgroups. Nature 2012;486:346-52.

**Davies et al 2012**
Davies BR, Greenwood H, Dudley P, Crafter C, Yu DH, Zhang J, et al. Preclinical pharmacology of AZD5363, an inhibitor of AKT: pharmacodynamics, antitumor activity, and correlation of monotherapy activity with genetic background. Mol Cancer Ther 2012;114:873–87.

**Di Leo et al 2018**
Di Leo A, Johnston S, Lee KS, Ciruelos E, Lønning PE, Janni W, et al. Buparlisib plus fulvestrant in postmenopausal women with hormone-receptor-positive, HER2-negative, advanced breast cancer progressing on or after mTOR inhibition (BELLE-3): a randomised, double-blind, placebo-controlled, phase 3 trial. Lancet Oncol 2018;19:87-100.

**Fayers et al 2011**
Fayers PM, Aaronson NK, Bjordal K, Groenvold M, Curran D, Bottomley A, on behalf of the EORTC Quality of Life Group. The EORTC QLQ-C30 Scoring Manual. 3rd ed. Brussels: European Organisation for Research and Treatment of Cancer; 2001.

**Folkerd et al 2014**
Folkerd EJ, Lønning PE, Dowsett M. Interpreting plasma estrogen levels in breast cancer: caution needed. J Clin Oncol. 2014 May 10;32(14):1396-400.

**Frogne et al 2005**
Frogne T, Jepsen JS, Larsen SS, Brown JS, Banerji U. Antiestrogen-resistant human breast cancer cells require activated protein kinase B/Akt for growth. Endocr Relat Cancer 2005;12:599–614.

**Fu et al 2014**
Fu X, Creighton CJ, Biswal NC, Kumar V, Shea M, Herrera S, et al. Overcoming endocrine resistance due to reduced PTEN levels in estrogen receptor-positive breast cancer by co-targeting mammalian target of rapamycin, protein kinase B, or mitogen-activated protein kinase. Breast Cancer Res 2014;16:430.

**Fulvestrant SmPC 2018**
Fulvestrant Summary of Product Characteristics. Available from: https://www.medicines.org.uk/emc/product/68/smpc.

**Gao et al 2014**
Gao Q, Patani N, Dunbier AK, Ghazoui Z, Zvelebil M, Martin LA, et al. Effect of aromatase inhibition on functional gene modules in estrogen receptor–positive breast cancer and their relationship with antiproliferative response. Clin Cancer Res 2014;20:2485-94.

**Ghayad et al 2010**
Ghayad SE, Vendrell JA, Larbi SB, Dumontet C, Bieche I, Cohen P. Endocrine resistance associated with activated ErbB system in breast cancer cells is reversed by inhibiting MAPK or PI3K/Akt signaling pathways. Int J Cancer 2010;126:545–62.

**GLOBOCAN 2018**
GLOBOCAN 2018. Breast Cancer Factsheet. Available at: URL: http://gco.iarc.fr/today/data/factsheets/cancers/20-Breast-fact-sheet.pdf.

**Gobbini et al 2018**
Gobbini E, Ezzalfani M, Dieras V, Bachelot T, Brain E, Debled M, et al. Time trends of overall survival among metastatic breast cancer patients in the real-life ESME cohort. Eur J Cancer 2018;96:17-24.

**Hammond et al 2010**
Hammond ME, Hayes DF, Dowsett M, Allred DC, Hagerty KL, Badve S, et al. American Society of Clinical Oncology/College of American Pathologists guideline recommendations for immunohistochemical testing of estrogen and progesterone receptors in breast cancer. J Clin Oncol 2010;28:2784-95; erratum in J Clin Oncol 2010;28:3543.

**Hortobagyi et al 2016**
Hortobagyi GN, Chen D, Piccart M, Rugo HS, Burris HA III, Pritchard KI, et al. Correlative analysis of genetic alterations and everolimus benefit in hormone receptor-positive, human

epidermal growth factor receptor 2-negative advanced breast cancer: Results From BOLERO-2. J Clin Oncol 2016;34:419-26.

**Howlader et al 2014**
Howlader N, Altekruse SF, Li CI, Chen VW, Clarke CA, Ries LA, et al. US incidence of breast cancer subtypes defined by joint hormone receptor and HER2 status. JNCI J Natl Cancer Inst 2014;106(5):dju055,doi:10.1093/jnci/dju055.

**Hyman et al 2017**
Hyman DM, Smyth LM, Donoghue MTA, Westin SN, Bedard PL, Dean EJ, et al. AKT inhibition in solid tumors with AKT1 mutations. J Clin Oncol 2017;35:2251-59. Erratum in: J Clin Oncol 2019;37:360.

**Im et al 2019**
Im SA, Lu YS, Bardia A, Harbeck N, Colleoni M, Franke F, et al. Overall Survival with Ribociclib plus Endocrine Therapy in Breast Cancer. N Engl J Med 2019;381:307-316.

**Jones et al 2019**
Jones RH, Carucci M, Casbard AC, Butler R, Alchami F, Bale CJ, et al. Capivasertib (AZD5363) plus fulvestrant versus placebo plus fulvestrant after relapse or progression on an aromatase inhibitor in metastatic ER-positive breast cancer (FAKTION): A randomized, double-blind, placebo-controlled, phase II trial. J Clin Oncol 2019;37:Suppl;Abstract 1105. Available at URL: https://meetinglibrary.asco.org/record/170933/abstract.

**Kluetz et al 2018**
Kluetz PG, O'Connor DJ, Soltys K. Incorporating the patient experience into regulatory decision making in the USA, Europe, and Canada. Lancet Oncol 2018;19:e267–74.

**Lan and DeMets 1983**
Lan KKG, DeMets DL. Discrete sequential boundaries for clinical trials. Biometrika 1983;70:659–63.

**Lindsley 2010**
Lindsley CW. The AKT/PKB family of protein kinases: a review of small molecule inhibitors and progress towards target validation: a 2009 update. Curr Top Med Chem 2010;10:458-77.

**Liu et al 2019**
Liu P, Cheng H, Roberts TM, and Zhao JJ. Targeting the phosphoinositide 3-kinase pathway in cancer. Nat Rev Drug Discov 2009;8:627–44.

**Lupichuk et al 2019**
Lupichuk SM, Recaldin B, Nixon NA, Mututino A, Joy AA. Real-world experience using exemestane and everolimus in patients with hormone receptor positive/HER2 negative breast cancer with and without prior CDK4/6 inhibitor exposure. In: Proceedings of the 2018 San

Antonio Breast Cancer Symposium; 2018 Dec 4-8; San Antonio, TX. Philadelphia (PA): AACR; Cancer Res 2019;79(4 Suppl):Abstract P4-13-06.

**Miller et al 2010**
Miller TW, Hennessy BT, Gonzalez-Angulo AM, Fox EM, Mills GB, Chen H, et al. Hyperactivation of phosphatidylinositol-3 kinase promotes escape from hormone dependence in estrogen receptor-positive human breast cancer. J Clin Invest 2010;120:2406-13.

**NCCN 2019**
National Comprehensive Cancer Network (NCCN) Clinical Practice Guidelines in Oncology. Version 1.2019 (July 2, 2019).

**Pereira et al 2016**
Pereira B, Chin SF, Rueda OM, Vollan HK, Provenzano E, Bardwell HA, et al. The somatic mutation profiles of 2,433 breast cancers refines their genomic and transcriptomic landscapes. Nat Commun 2016;7:11479. doi: 10.1038/ncomms11479.

**Ribas et al 2015**
Ribas R, Pancholi S, Guest SK, Maragoni E, Gao Q, Thuleau A, et al. AKT antagonist AZD5363 influences estrogen receptor function in endocrine-resistant breast cancer and synergizes with fulvestrant (ICI182780) *in vivo*. Mol Cancer Ther 2015;14:2035-48.

**Robertson et al 2019**
Robertson JF, Paridaens R, Bogaerts J, Lichfield J, Bradbury I, Campbell C. Meta-analyses of visceral versus non-visceral metastases treated by SERM, AI & SERD agents as first-line endocrine therapy (ET) for HR+ breast cancer (BC). San Antonio Breast Cancer Symposium, 2018 Dec 4-8; San Antonio, TX. Philadelphia (PA): Cancer Res 2019;79(4 Suppl):Abstract P4-13-11. Available at URL: http://cancerres.aacrjournals.org/content/79/4_Supplement/P4-13-11.short?rss=1.

**Schmid et al 2018**
Schmid P, Abraham J, Chan S, Wheatley D, Brunt M, Nemsadze G, et al. AZD5363 plus paclitaxel versus placebo plus paclitaxel as first-line therapy for metastatic triple-negative breast cancer (PAKT): A randomised, double-blind, placebo-controlled, phase II trial. J Clin Oncol 2018;36:No15(suppl)1007–1007.

**Slamon 2019**
Slamon DJ, Neven P, Chia S, Fasching PA, De Laurentiis M, Im S, et al. Overall survival (OS) results of the Phase III MONALEESA-3 trial of postmenopausal patients (pts) with hormone receptor-positive (HR+), human epidermal growth factor 2-negative (HER2−) advanced breast cancer (ABC) treated with fulvestrant (FUL) ± ribociclib (RIB). ESMO 2019 [LBA 7].

**Sledge 2019**
Sledge G, Toi M, Neven P, Sohn J, Inoue K, Pivot X, et al. MONARCH 2: Overall survival of abemaciclib plus fulvestrant in patients with HR+, HER2 advanced breast cancer. ESMO [LBA 6].

**Smyth et al 2017**
Smyth LM, Oliveira M, Ciruelos E, Tamura K, El-Khoueiry A, Mita A, et al. AZD5363 in combination with fulvestrant in AKT1-mutant ER-positive metastatic breast cancer. SABCS 2017, P5-21-32. Available at URL: http://cancerres.aacrjournals.org/content/78/4_Supplement/P5-21-32.

**Solomayer et al 2000**
Solomayer EF, Diel IJ, Meyberg GC, Gollan C, Bastert G. Metastatic breast cancer: clinical course, prognosis and therapy related to the first site of metastasis. Breast Cancer Res Treat 2000;59:271–8.

**Spiessens and Debois 2010**
Spiessens B, Debois M. Adjusted significance levels for subgroup analyses in clinical trials. Contemp Clin Trials 2010;31:647-56.

**Spoerke et al 2016**
Spoerke JM, Gendreau S, Walter K, Qui J, Wilson TR, Savage H, et al. Heterogeneity and clinical significance of ESR1 mutations in ER-positive metastatic breast cancer patients receiving fulvestrant. Nat Commun 2016;7:11579. doi: 10.1038/ncomms11579.

**Sprangers et al 1996**
Sprangers MA, Groenvold M, Arraras JI, Franklin J, te Velde A, Muller M, et al. The European Organization for Research and Treatment of Cancer breast cancer-specific quality-of-life questionnaire module: first results from a three-country field study. J Clin Oncol 1996;14:2756–68.

**Tamura et al 2016**
Tamura K, Hashimoto J, Tanabe Y, Kodaira M, Yonemori K, Seto T, et al. Safety and tolerability of AZD5363 in Japanese patients with advanced solid tumors. Cancer Chemother Pharmacol 2016;77:787–95.

**Tokunaga et al 2006**
Tokunaga E, Kataoka A, Kimura Y, Oki E, Mashino K, Nishida K, et al. The association between Akt activation and resistance to hormone therapy in metastatic breast cancer. Eur J Cancer 2006;42:629-35.

**Turner et al 2017**
Turner NC, Andre F, Cristofanilli M, Verma S, Iwata H, Loi S, et al. Treatment post-progression in women with endocrine-resistant HR+/HER2- advanced breast cancer who

received palbociclib plus fulvestrant in PALOMA-3. In: Proceedings of the 2016 San Antonio Breast Cancer Symposium; 2016 Dec 6-10; San Antonio, TX. Philadelphia (PA): AACR; Cancer Res 2017;77(4 Suppl):Abstract P4-22-06.

**van der Hage et al 2004**
Van der Hage JA, van den Broek LJ, Legrand C, Clahsen PC, Bosch CJ, Robanus-Maandag EC, et al. Overexpression of p70 S6 kinase protein is associated with increased risk of loco-regional recurrence in node negative premenopausal early breast cancer patients. Br J Cancer 2004;90:1543–50.

**van Reenen and Janssen 2015**
van Reenen M, Janssen B. EQ-5D-5L User Guide. Version 2.1. (April 2015). EuroQol Research Foundation 2015. Available from URL: https://euroqol.org/wp-content/uploads/2016/09/EQ-5D-5L_UserGuide_2015.pdf.

**WHO 2012**
World Health Organization. Blood Donor Selection: Guidelines on Assessing Donor Suitability for Blood Donation. Geneva: World Health Organization; 2012. Available at URL: https://www.ncbi.nlm.nih.gov/books/NBK138218/.

**Wolff et al 2018**
Wolff AC, Hammond MEH, Allison KH, Harvey BE, Mangu PB, Bartlett JMS, et al. Human Epidermal Growth Factor Receptor 2 Testing in Breast Cancer: American Society of Clinical Oncology/College of American Pathologists Clinical Practice Guideline Focused Update. Arch Pathol Lab Med 2018;142:1364-82.

**Yardley et al 2013**
Yardley DA, Noguchi S, Pritchard KI, Burris HA III, Baselga J, Gnant M, et al. Everolimus plus exemestane in postmenopausal patients with HR+ breast cancer: BOLERO-2 final progression-free survival analysis. Adv Ther 2013;30:870-84.

**Yates et al 2015**
Yates JW, Dudley P, Cheng J, D'Cruz C, Davies BR. Validation of a predictive modeling approach to demonstrate the relative efficacy of three different schedules of the AKT inhibitor AZD5363. Cancer Chemother Pharmacol 2015;76:343–56.

**Yi and Lauring 2016**
Yi KH, Lauring J. Recurrent AKT mutations in human cancers: functional consequences and effects on drug sensitivity. Oncotarget 2016;7:4241–51.

# 11 SUPPORTING DOCUMENTATION AND OPERATIONAL CONSIDERATIONS

# Appendix A Guidelines for evaluation of objective tumour response using RECIST v1.1 Criteria (Response Evaluation Criteria in Solid Tumours)

## Introduction

This appendix details the implementation of Response Evaluation Criteria in Solid Tumours version 1.1 (RECIST v1.1) guidelines (Eisenhauer et al 2009). Investigator assessments will use the RECIST v1.1 guidelines described in this appendix.

## Imaging modalities and acquisition specifications for RECIST v1.1

A summary of the imaging modalities that can be used for tumour assessment of target lesions (TLs), non-target lesions (NTLs), and new lesions (NLs) is provided in Table 15.

**Table 15 Summary of imaging modalities for tumour assessment**

| Target lesions | Non-target lesions | New lesions |
|---|---|---|
| CT | CT | CT |
| MRI | MRI | MRI |
| | Plain X-ray | Plain X-ray |
| | Chest X-ray | Chest X-ray |
| | | Bone scan (Scintigraphy) |
| | | FDG-PET/CT |

CT Computed tomography; MRI Magnetic resonance imaging; FDG-PET/CT $^{18}$F-Fluoro-deoxyglucose positron emission tomography/CT

### CT and MRI

Computed tomography (CT) with intravenous (IV) contrast, is the preferred imaging modality, and magnetic resonance imaging (MRI) with IV contrast being acceptable should CT be contraindicated, to generate reproducible anatomical images for tumour assessments, ie, for measurement of TLs, assessment of NTLs, and identification of NLs. It is essential that the same correct imaging modality, image acquisition parameters (eg, anatomic coverage, imaging sequences, etc.), imaging facility, tumour assessor (eg, radiologist), and method of tumour assessment (eg, RECIST v1.1) are used consistently for each patient throughout the study. The use of the same scanner for serial scans is recommended, if possible. It is important to follow the image collection/tumour assessment schedule as closely as possible (refer to the clinical study protocol Schedule of Activities [Section 1.1], and this on-study imaging schedule MUST be followed regardless of any delays in dosing or missed imaging visits. If an unscheduled assessment is performed (eg, to investigate clinical signs/symptoms of progression), and the patient has not progressed, every attempt should be made to perform the subsequent scan acquisitions at the next scheduled imaging visit.

Due to its inherent rapid acquisition (seconds), CT is the imaging modality of choice. Body scans should be performed with breath-hold scanning techniques if possible. Therefore, CT of the chest is recommended over MRI due to significant motion artefacts (heart, major blood vessels, breathing) associated with MRI. MRI has excellent contrast and spatial and temporal resolutions; however, there are many image acquisition variables involved in MRI, which greatly impact image quality, lesion conspicuity and measurement. Furthermore, the availability of MRI is variable globally. The modality used at follow-up should be the same as was used at baseline, and the lesions should be measured/assessed on the same pulse sequence. In general, local oncology diagnostic imaging parameters are applied for scan acquisition. It is beyond the scope of this appendix to prescribe specific MRI pulse sequence parameters for all scanners, body parts, and diseases.

The most critical CT and MRI image acquisition parameters for optimal tumour evaluation are *anatomic coverage, contrast administration, slice thickness, and reconstruction interval.*

**a. Anatomic coverage:** Optimal anatomic coverage for most solid tumours is the chest, abdomen and pelvis. Coverage should encompass all areas of known predilection for metastases in the disease under evaluation and should additionally investigate areas that may be involved based on signs and symptoms of individual patients. Because a lesion later identified in a body part not scanned at baseline would be considered as a new lesion representing disease progression, careful consideration should be given to the extent of imaging coverage at baseline and at subsequent follow-up timepoints. This will enable better consistency not only of tumour measurements but also identification of a new disease.

Required anatomical regions to be imaged for assessment of tumour burden (TLs and/or NTLs) at baseline and follow-up visits vary according to the study, and these are specified in the main clinical study protocol (eg, Schedule of Activities [Section 1.1]). Examples include the following:

- IV contrast enhanced CT of chest-abdomen (including the entire liver and both adrenal glands) and pelvis
- Non-contrast CT of chest and IV contrast enhanced abdomen (including the entire liver and both adrenal glands) and pelvis
- IV contrast enhanced CT or MRI of the head and neck
- IV contrast enhanced MRI (preferred) or CT of the brain

**b. IV contrast administration**: Optimal visualisation and measurement of metastases in solid tumours require consistent administration (dose and rate) of IV contrast as well as timing of scanning. An adequate volume of a suitable contrast agent should be given so that the tumour lesions are demonstrated to best effect and a consistent method is used on subsequent

examinations for any given patient. Oral contrast is recommended to help visualize and differentiate structures in the abdomen and pelvis.

**c. Slice thickness and reconstruction interval:** It is recommended that CT or MRI scans be acquired/reconstructed as contiguous (no gap) with ≤5 mm slice thickness throughout the entire anatomic region of interest for optimal lesion measurements. Exceptionally, particular institutions may perform medically acceptable scans at slice thicknesses greater than 5 mm. If this occurs, the minimum size of measurable lesions at baseline should be twice the slice thickness of the baseline scans.

For CT scans, all window settings should be included in the assessment, particularly in the thorax where lung and soft tissue windows should be considered. When measuring lesions, the TL should be measured on the same window setting for repeated examinations throughout the study.

**Chest X-ray**

Chest X-ray assessment will not be used for assessment of TL. Chest X-ray can, however, be used to assess NTL and to identify the presence of NLs. However, there is preference that a higher resolution modality such as CT be used to confirm the presence of NLs.

**Plain X-ray**

Plain X-ray may be used as a method of assessment for bone NTL and to identify the presence of new bone lesions.

**Isotopic bone scan**

A bone scan should be performed at screening; thereafter, a bone scan should only be performed in the event of clinical suspicion of progression of existing bone lesions that cannot be visualised on CT or MRI, the development of new bone lesions or in the assessment of a complete response (CR), if any disease was evident at screening.

Isotopic bone scans may be used as a method of assessment to identify the presence of new bone lesions at follow-up visits. NLs may be recorded in case positive hot-spots appear on a bone scan that were not present on a previous bone scan; however, a newly observed equivocal hot-spot on a bone scan which cannot be verified with correlative imaging (CT, MRI, X-ray) of the same anatomical region shall not be the only trigger for a progressive disease (PD) assessment at that timepoint.

**FDG-PET/CT**

$^{18}$F-Fluoro-deoxyglucose positron emission tomography/computed tomography/CT (FDG-PET/CT) scans may be used as a method for identifying new extrahepatic lesions (but not intrahepatic lesions) for RECIST v1.1 assessments, according to the following algorithm:

NLs will be recorded where there is positive $^{18}$F-Fluoro-deoxyglucose uptake[1] not present on baseline or a prior FDG-PET scan or in a location corresponding to an NL on a companion CT/MRI collected close in time to the FDG-PET scan. The PET portion of the PET/CT introduces additional data that may bias an investigator if it is not routinely or serially performed. Therefore, if there is no baseline or prior FDG-PET scan available for comparison, and no evidence of NLs on companion CT/MRI scans, then follow-up CT/MRI assessments should continue as per the regular imaging schedule in order to verify the unequivocal presence of NLs.

At present, low dose or attenuation correction CT portions of a combined FDG-PET/CT scan are of limited use in anatomically-based efficacy assessments, and it is therefore suggested that they should not substitute for dedicated diagnostic contrast-enhanced CT scans for tumour measurements by RECIST v1.1. In exceptional situations, if a site can document that the CT performed, as part of a PET/CT examination, is of identical diagnostic quality (with intravenous contrast) to a dedicated diagnostic CT scan, then the CT portion of the PET/CT can be used for RECIST v1.1 tumour assessments. Caution that this is not recommended because the PET portion of the CT introduces additional (PET) data that may bias an investigator if it is not routinely or serially performed.

### Ultrasound

Ultrasound examination will not be used for RECIST v1.1 assessment of tumours as it is not a reproducible acquisition method (operator dependent), is patientive in interpretation and may not provide an accurate assessment of true tumour size. Tumours identified by ultrasound will need to be assessed by correlative CT or MRI anatomical scan.

## Other tumour assessments

### Clinical examination

Clinical examination of skin/surface lesions (by visual inspection or manual palpation) will not be used for RECIST v1.1 assessments. Tumours identified by clinical examination will need to be assessed by correlative CT or MRI anatomical scans.

### Endoscopy and laparoscopy

Endoscopy and laparoscopy will not be used for tumour assessments as they are not validated in the context of tumour assessment.

### Histology and cytology

[1] A positive FDG-PET scan lesion should be reported only when an uptake (eg, SUV) greater than twice that of the surrounding tissue or liver is observed.

Histology or tumour markers on tumour biopsy samples will not be used as part of the tumour response assessment as per RECIST v1.1.

Results of cytological examination for the neoplastic origin of any effusion (eg, ascites, pericardial effusion, pleural effusion) that appears or worsens during the study will not be used as part of the tumour response assessment as per RECIST v1.1.

Furthermore, an overall assessment of complete response (all other disease disappears/reverts to normal) would be changed to partial response if an effusion remains present radiologically.

## Measurability of tumour lesions at baseline

### RECIST v1.1 measurable lesions at baseline:

A tumour lesion that can be accurately measured at baseline as ≥10 mm in the longest diameter for non-nodal lesions or ≥15 mm in short axis[2] diameter for lymph node lesions with IV contrast-enhanced CT or MRI and that is suitable for accurate repeated measurements. Please see additional RECIST v1.1 guidance below on measurability of intrahepatic hepatocellular carcinoma (HCC) lesions and porta hepatis lymph nodes.

### Non-measurable lesions at baseline:

- Truly non-measurable lesions include the following:
  - Bone lesions (see exception below for soft tissue component)
  - Leptomeningeal disease
  - Ascites, pleural, or pericardial effusion
  - Inflammatory breast disease
  - Lymphangitic involvement of skin or lung
- All other lesions, including small lesions (longest diameter <10 mm or pathological lymph nodes with ≥10 mm to <15 mm short axis diameter at baseline[3])
- Previously irradiated lesions[4]
- Brain metastasis

[2] The short axis is defined as the longest in-plane axis perpendicular to long axis.

[3] Lymph nodes with <10 mm short axis diameter are considered non-pathological and should not be recorded or followed as NTLs.

[4] Localised post-radiation changes which affect lesion sizes may occur. Therefore, lesions that have been previously irradiated are typically considered non-measurable and as NTL at baseline and followed up as part of the NTL assessment.

**Special considerations regarding lesion measurability at baseline:**

- Bone lesions
  - Bone scan, PET scan or plain X-ray are not considered adequate imaging techniques to measure bone lesions; however, these techniques can be used to confirm the presence or disappearance of bone lesions
  - Lytic bone lesions or mixed lytic–blastic lesions, with identifiable soft tissue components, can be considered measurable if the soft tissue component meets the definition of measurability
  - Blastic bone lesions are considered non-measurable
- Cystic lesions thought to represent cystic metastases can be considered measurable lesions if they meet the criteria for measurability from a radiological point of view, but if non-cystic lesions are present in the same patient, these should be selected over cystic lesions as TLs

**RECIST v1.1 target lesion selection at baseline:**

A maximum of 5 measurable lesions, with a maximum of 2 lesions per organ (including lymph nodes collectively considered as a single organ), representative of all lesions involved should be identified as TLs at baseline. TLs should be selected on the basis of their size (longest diameter for non-nodal lesions or short axis diameter for nodal lesions), but in addition should be those that lend themselves to reproducible repeated measurements. It may be the case that, on occasion, the largest lesion does not lend itself to reproducible measurement in which circumstance the next largest lesion, which can be measured reproducibly, should be selected.

Lymph nodes, in any location (local/regional and distant), are collectively considered as a single organ, with a maximum of 2 lymph node locations as TLs. A bilateral organ (eg, adrenal glands), a segmented organ (eg, liver), or a multilobed organ (eg, lung) is each considered as a single organ.

The site and location of each TL should be documented as well as the longest axis diameter for non-nodal lesions (or short axis diameter for lymph nodes). All measurements should be recorded in millimetres. At baseline the sum of the diameters for all TL will be calculated and reported as the baseline sum of diameters. At follow-up visits the sum of diameters for all TL will be calculated and reported as the follow-up sum of diameters.

**Special cases for target lesion assessment at baseline:**

- For TL measurable in 2 or 3 dimensions, always report the longest diameter. For pathological lymph nodes measurable in 2 or 3 dimensions, always report the short axis diameter

- When lymph nodes are coalesced and no longer separable in a conglomerate mass, the vector of the longest diameter should be used to determine the perpendicular vector for the maximal short axis diameter of the coalesced mass. Non-nodal lesions that coalesce should similarly be assessed by the longest axis diameter
- If the CT/MRI slice thickness used is >5 mm, the minimum size of measurable disease at baseline should be twice the slice thickness of the baseline scan
- Tumour lesions selected for fresh screening biopsy should not be selected as TLs, unless imaging occurred at least ~2 weeks after biopsy, allowing time for healing

**RECIST v1.1 non-target lesion selection at baseline:**

All other lesions, including non-measurable lesions and surplus measurable lesions not recorded as TLs should be identified as NTLs at baseline. Measurements of these lesions are not required, but the presence or absence of each should be noted throughout follow-up.

## Evaluation of tumour response and progression

### RECIST v1.1 target lesion assessment at follow-up

This section defines the criteria used to determine objective tumour visit response for RECIST v1.1-defined TLs. The imaging modality, location, and scan date of each TL identified previously at baseline should be documented at follow-up visits with the long axis diameter for non-nodal lesions or short axis diameter for lymph node lesions. All measurements should be recorded in millimetres. The sum of the diameters for all TL at each follow-up visit will be compared to the baseline sum of diameters (for response or stable disease) or to the smallest prior (nadir) sum of diameters (for progression) (Table 16).

**Special cases for target lesion assessment at follow-up:**

- If a lesion has completely disappeared, the diameter should be recorded as 0 mm. If a lesion appears in the same location on a subsequent scan, it will be recorded as an NL
- If a TL splits into 2 or more parts, then record the sum of the diameters of those parts
- If 2 or more TLs merge, then the sum of the diameters of the combined lesion should be recorded for 1 of the lesions and 0 mm recorded for the other lesion(s). If the merged TLs are non-nodal lesions, record the long axis diameter of the merged lesion. If pathologic lymph nodes coalesce and are no longer individually separable within a conglomerate mass, the vector of the longest diameter of the coalesced mass should be used to determine the perpendicular vector for the maximal short axis diameter
- If a TL is believed to be present and is faintly seen but too small to measure, a default value of 5 mm should be assigned. If an accurate measure can be given, this should be recorded, even if it is below 5 mm
- If a TL cannot be measured accurately due to it being too large, provide an estimate of the size of the lesion. The choice of 'Too large to measure' in the case report form will trigger an overall visit response of PD

- When a TL has had any intervention e.g., definitive radiotherapy, embolisation, surgery, transcatheter arterial chemoembolization (TACE), etc., during the study, the size of the TL should still be provided where possible and the intervention recorded in the RECIST v1.1 case report form for the current imaging visit and all subsequent visits. If a TL has been completely removed (surgery) or disappears, the longest diameter should be recorded as 0 mm

**Table 16 RECIST v1.1 evaluation of target lesions**

| **Complete response (CR)** | **Disappearance of all TLs since baseline. Any pathological lymph nodes selected as TLs must have a reduction in short axis diameter to <10 mm.** |
|---|---|
| Partial response (PR) | At least a 30% decrease in the sum of the diameters of TL, taking as reference the baseline sum of diameters |
| Stable disease (SD) | Neither sufficient decrease in sum of diameters to qualify for PR nor sufficient increase to qualify for PD |
| Progression of disease (PD) | At least a 20% increase in the sum of diameters of TLs, taking as reference the smallest previous sum of diameters (nadir) – this includes the baseline sum if that is the smallest on study. In addition to the relative increase of 20%, the sum must also demonstrate an absolute increase of at least 5 mm from nadir |
| Not evaluable (NE) | Only relevant if any of the TLs at follow-up were not assessed or not evaluable (e.g., missing anatomy) or had a lesion intervention at this visit. NOTE: if the sum of diameters meets the progressive disease criteria, progressive disease overrides not evaluable as a TL response |
| Not applicable (NA) | Only relevant if no TLs present at baseline |

CR complete response; PR partial response; PD progression of disease; NE not evaluable; SD stable disease; TL target lesion.

### RECIST v1.1 non-target lesion assessment at follow-up

All other lesions (or sites of disease) not recorded as TL should be identified as NTL at baseline. Measurements are not required for these lesions, but their status should be followed at subsequent visits. At each visit an overall assessment of the NTL response should be recorded by the investigator.

To achieve ‘unequivocal progression’ on the basis of NTLs, there must be an overall level of substantial worsening in non-target disease such that, even in presence of stable disease or partial response in TLs, the overall tumour burden has increased sufficiently to merit unequivocal progression by NTLs. A modest ‘increase’ in the size of one or more NTLs is usually not sufficient to qualify for unequivocal progression status. The designation of overall progression solely on the basis of change in non-target disease in the face of stable disease (SD) or progressive disease (PD) of target disease will therefore be extremely rare (Table 17).

**Table 17 RECIST v1.1 evaluation of non-target lesions**

| Complete response (CR) | Disappearance of all NTLs since baseline. All lymph nodes must be non-pathological in size (<10 mm short axis). |
|---|---|
| Non-CR/Non-PD | Persistence of one or more NTL |
| Progression (PD) | Unequivocal progression of existing NTLs. Unequivocal progression may be due to an important progression in one lesion only or in several lesions. In all cases the progression MUST be clinically significant for the physician to consider changing (or stopping) therapy |
| Not evaluable (NE) | Only relevant when one or some of the NTLs were not assessed and, in the investigator's opinion, they are not able to provide an evaluable overall NTL assessment at this visit<br>NOTE: for patients without TLs at baseline, this is relevant if any of the NTLs were not assessed at this visit and the progression criteria have not been met |
| Not applicable (NA) | Only relevant if no NTLs present at baseline |

CR complete response; PR partial response; PD progression of disease; NE not evaluable; NTL non-target lesion; TL target lesion.

### RECIST v1.1 new lesion identification at follow-up

Details including the imaging modality, the date of scan, and the location of any NLs will be recorded in the case report form. The presence of 1 or more NLs is assessed as progression. The finding of an NL should be unequivocal: ie, not attributable to differences in scanning technique, change in imaging modality, or findings thought to represent something other than tumour. If an NL is equivocal, for example because of its small size, the treatment and tumour assessments should be continued until the previously (pre-existing) new lesion has been assessed as unequivocal at a follow-up visit, and then the progression date should be declared using the date of the initial scan when the NL first appeared.

A lesion identified at a follow-up assessment in an anatomical location that was not scanned at baseline is considered a NL and will indicate disease progression.

### RECIST v1.1 evaluation of overall visit response at follow-up

Derivation of overall visit response as a result of the combined assessment of TLs, NTLs, and NLs is identical between RECIST v1.1 and RECIST v1.1 using the algorithm shown in Table 18.

**Table 18 RECIST v1.1 overall visit response**

| Target lesions | Non-target lesions | New lesions | Overall visit response |
|---|---|---|---|
| CR | CR | No | CR |
| CR | NA | No | CR |

| Target lesions | Non-target lesions | New lesions | Overall visit response |
|---|---|---|---|
| NA | CR | No | CR |
| CR | Non-CR/Non-PD | No | PR |
| CR | NE | No | PR |
| PR | Non-PD or NE or NA | No | PR |
| SD | Non-PD or NE or NA | No | SD |
| NA | Non-CR/Non-PD | No | SD (Non-CR/Non-PD[a]) |
| NE | Non-PD or NE or NA | No | NE |
| NA | NE | No | NE |
| PD | Any | Yes or No | PD |
| Any | PD | Yes or No | PD |
| Any | Any | Yes | PD |

[a] Non-CR/Non-PD for overall response if only non-target lesions (no TLs) are present at baseline.
NOTE: An overall assessment of complete response (all other disease disappears/reverts to normal) would be changed to partial response if ascites remains present radiologically.
CR complete response; NA not applicable; NE not evaluable; PD progressive disease; PR partial response; (only relevant if there were no target lesions at baseline or no non-target lesions at baseline); SD stable disease; TL target lesion.

The following overall visit responses are possible depending on the extent of tumour disease at baseline:

- For patients with TLs (at baseline): CR, PR, SD, PD, or NE
- For patients with NTLs only (at baseline): CR, Non-CR/Non-PD, PD, or NE

### Central imaging

Images, including unscheduled visit scans, will be collected on an ongoing basis and sent to an AstraZeneca-appointed imaging Contract Research Organisation (iCRO) for QC, storage, and for Blinded Independent Central Review (BICR). Digital copies of all original scans should be stored at the investigator site as Source documents. Electronic image transfer from the sites to the iCRO is strongly encouraged. A BICR of images will be performed at the discretion of AstraZeneca. Results of these independent reviews will not be communicated to investigators, and results of investigator tumour assessments will not be shared with the central reviewers. The management of patients will be based in part upon the results of the tumour assessments conducted by the investigator. Further details of the BICR will be documented in an Independent Review Charter.

## REFERENCE

**Eisenhauer et al 2009**
Eisenhauer EA, Therasse P, Bogaerts J, Schwartz LH, Sargent D, Ford R, et al. New response evaluation criteria in solid tumors: revised RECIST guideline (version 1.1). Eur J Cancer 2009;45(2):228-47.

# Appendix B ECOG/WHO Performance Status

The Eastern Cooperative Oncology Group (ECOG)/ World Health Organisation (WHO) performance scale is presented in Table 19.

**Table 19 ECOG performance status scale (Oken et al 1982)**

| Grade | |
|---|---|
| 0 | Fully active and able to carry on all pre-disease performance without restriction (Karnofsky 90-100). |
| 1 | Restricted in physically strenuous activity but ambulatory and able to carry out work of a light or sedentary nature, eg, light house work, office work (Karnofsky 70-80). |
| 2 | Ambulatory and capable of all self-care but unable to carry out any work activities. Up and about more than 50% of waking hours (Karnofsky 50-60). |
| 3 | Capable of only limited self-care, confined to bed or chair more than 50% of waking hours (Karnofsky 30-40). |
| 4 | Completely disabled. Cannot carry out any self-care. Totally confined to bed or chair (Karnofsky 10-20). |

## REFERENCE

**Oken et al 1982**
Oken MM, Creech RH, Tormey DC, Horton J, Davis TE, McFadden ET, et al. Toxicity and response criteria of the Eastern Cooperative Oncology Group. Am J Clin Oncol.1982;5(6):649-655.

# Appendix C Regulatory, ethical and study oversight considerations

## C 1 Regulatory and ethical considerations

This study will be conducted in accordance with the protocol and with the following:

- Consensus ethical principles derived from international guidelines including the Declaration of Helsinki and Council for International Organisations of Medical Sciences (CIOMS) International Ethical Guidelines
- Applicable International Council for Harmonisation of Technical Requirements for Pharmaceuticals for Human Use (ICH) Good Clinical Practice (GCP) Guidelines
- Applicable laws and regulations

The protocol, protocol amendments, informed consent form (ICF), Investigator's Brochure, and other relevant documents (eg, advertisements) must be submitted to an institutional review board (IRB)/independent ethics committee (IEC) by the investigator and reviewed and approved by the IRB/IEC before the study is initiated.

Any amendments to the protocol will require IRB/IEC approval before implementation of changes made to the study design, except for changes necessary to eliminate an immediate hazard to study patients.

The investigator will be responsible for the following:

- Providing written summaries of the status of the study to the IRB/IEC annually or more frequently in accordance with the requirements, policies, and procedures established by the IRB/IEC
- Notifying the IRB/IEC of SAEs or other significant safety findings as required by IRB/IEC procedures
- Providing oversight of the conduct of the study at the site and adherence to requirements of 21 CFR (Code of Federal Regulations), ICH guidelines, the IRB/IEC, European regulation 536/2014 for clinical studies (if applicable), and all other applicable local regulations

The study will be performed in accordance with the AstraZeneca policy on Bioethics and Human Biological Samples.

## C 2 Financial disclosure

Investigators and sub-investigators will provide the sponsor with sufficient, accurate financial information as requested to allow the sponsor to submit complete and accurate financial certification or disclosure statements to the appropriate regulatory authorities. Investigators are responsible for providing information on financial interests during the course of the study and for 1 year after completion of the study.

## C 3 Informed consent process

The investigator or his/her representative will explain the nature of the study to the patient or his/her legally authorised representative and answer all questions regarding the study.

Patients must be informed that their participation is voluntary. Patients or their legally authorised representative will be required to sign a statement of informed consent that meets the requirements of 21 CFR 50, local regulations, ICH guidelines, Health Insurance Portability and Accountability Act (HIPAA) requirements, where applicable, and the IRB/IEC or study centre.

The medical record must include a statement that written informed consent was obtained before the patient was enrolled in the study and the date and time the written consent was obtained. The authorised person obtaining the informed consent must also sign the ICF.

Patients must be re-consented to the most current version of the ICF(s) during their participation in the study.

A copy of the ICF(s) must be provided to the patient or the patient's legally authorised representative.

If a patient declines to participate in any voluntary exploratory genetic research component of the study, there will be no penalty or loss of benefit to the patient and he/she will not be excluded from other aspects of the study.

A patient who is rescreened is not required to sign another ICF if the rescreening occurs within 28 days from the previous ICF signature date.

The ICF will contain a separate section that addresses the use of remaining mandatory samples for optional exploratory research. The investigator or authorised designee will explain to each patient the objectives of the exploratory research. Patients will be told that they are free to refuse to participate and may withdraw their consent at any time and for any reason during the storage period. The patient will give a separate agreement to allow any remaining specimens to be used for exploratory research. Patients who decline to participate in this optional research will indicate this in the ICF. If a patient withdraws consent to the use

of donated biological samples, the samples will be disposed of/destroyed, and the action documented. If samples already have been analysed at the time of the request, AstraZeneca will not be obliged to destroy the results of this research.

## C 4 Data protection

Each patient will be assigned a unique identifier by the sponsor. Any patient records or data sets transferred to the sponsor will contain only the identifier; patient names or any information which would make the patient identifiable will not be transferred.

The patient must be informed that his/her personal study-related data will be used by the sponsor in accordance with local data protection law. The level of disclosure must also be explained to the patient.

The patient must be informed that his/her medical records may be examined by Clinical Quality Assurance auditors or other authorised personnel appointed by the sponsor, by appropriate IRB/IEC members, and by inspectors from regulatory authorities.

## C 5 Committees structure

A Trial Steering Committee (TSC) that includes Principal Investigators for this study and that may include Principal Investigators from the other pivotal studies will provide:

- Advice on any aspect of the study design or conduct based on requests from the sponsor
- Review relevant research (completed, ongoing, and pending) which may impact upon the study, assure consistency across the entire capivasertib pivotal programme, and to support the study team with interpretation of study outcomes

A Steering Committee Charter will define the primary responsibilities of the steering committee, its members, and the purpose and timing of meetings.

An Independent Data Monitoring Committee (IDMC) will be established in order to assess the progress of the clinical study at regular intervals. This will include reviewing unblinded safety and tolerability data.

The safety of all AstraZeneca clinical studies is closely monitored on an ongoing basis by AstraZeneca representatives in consultation with Patient Safety. Issues identified will be addressed; for instance, this could involve amendments to the clinical study protocol and letters to investigators.

## C 6 Dissemination of clinical study data

A description of this clinical trial will be available on http://astrazenecaclinicaltrials.com and http://www.clinicaltrials.gov as will the summary of the D3615C00001 study results when they are available. The clinical trial and/or summary of D3615C00001 study results may also be available on other websites according to the regulations of the countries in which the D3615C00001 study is conducted.

## C 7 Data quality assurance

All patient data relating to the study will be recorded on an electronic case report form (eCRF) unless transmitted to the sponsor or designee electronically (eg, laboratory data). The investigator is responsible for verifying that data entries are accurate and correct by physically or electronically signing the eCRF.

The investigator must maintain accurate documentation (source data) that supports the information entered in the eCRF.

The investigator must permit study-related monitoring, audits, IRB/IEC review, and regulatory agency inspections and provide direct access to source data documents.

The sponsor or designee is responsible for the data management of this study including quality checking of the data.

Study monitors will perform ongoing source data verification to confirm that data entered into the eCRF by authorised site personnel are accurate, complete, and verifiable from source documents; that the safety and rights of patients are being protected; and that the study is being conducted in accordance with the currently approved protocol and any other study agreements, ICH GCP, and all applicable regulatory requirements.

Records and documents, including signed ICFs, pertaining to the conduct of this study must be retained by the investigator in accordance to local regulations after study completion unless local regulations or institutional policies require a longer retention period. No records may be destroyed during the retention period without the written approval of the sponsor. No records may be transferred to another location or party without written notification to the sponsor.

## C 8 Source documents

Source documents provide evidence for the existence of the patient and substantiate the integrity of the data collected. Source documents are filed at the investigator's site.

Data reported on the eCRF that are transcribed from source documents must be consistent with the source documents or the discrepancies must be explained. The investigator may need

to request previous medical records or transfer records, depending on the study. Also, current medical records must be available.

## C 9 Study and Site Closure

The sponsor designee reserves the right to close the study site or terminate the study at any time for any reason at the sole discretion of the sponsor. The study may be stopped if, in the judgment of AstraZeneca, trial subjects are placed at undue risk because of clinically significant findings that:

- Meet individual stopping criteria or are otherwise considered significant
- Are assessed as causally related to study drug
- Are not considered to be consistent with continuation of the study

Regardless of the reason for termination, all data available for the subject at the time of discontinuation of follow-up must be recorded in the CRF. All reasons for discontinuation of treatment must be documented.

In terminating the study, the sponsor will ensure that adequate consideration is given to the protection of the subjects' interests.

Study sites will be closed upon study completion. A study site is considered closed when all required documents and study supplies have been collected and a study-site closure visit has been performed.

The investigator may initiate study-site closure at any time, provided there is reasonable cause and sufficient notice is given in advance of the intended termination.

Reasons for the early closure of a study site by the sponsor or investigator may include but are not limited to:

- Failure of the investigator to comply with the protocol, the requirements of the IRB/IEC or local health authorities, the sponsor's procedures, or GCP guidelines
- Inadequate recruitment of participants by the investigator
- Discontinuation of further study intervention development

## C 10 Publication policy

The results of this study may be published or presented at scientific meetings. If this is foreseen, the investigator agrees to submit all manuscripts or abstracts to the sponsor before submission. This allows the sponsor to protect proprietary information and to provide comments.

The sponsor will comply with the requirements for publication of study results. In accordance with standard editorial and ethical practice, the sponsor will generally support publication of multicentre studies only in their entirety and not as individual site data. In this case, a coordinating investigator will be designated by mutual agreement.

Authorship will be determined by mutual agreement and in line with International Committee of Medical Journal Editors authorship requirements.

# Appendix D Guidance regarding potential interactions of capivasertib with concomitant medications

NOTE: Patients are not eligible to enter the study if they have received any of the medications specified in the Exclusion Criterion 22 or are unable to meet the cautions and restrictions described below.

## D 1 Drugs that may influence capivasertib pharmacokinetics

Based on results from in vitro studies, capivasertib is a substrate of CYP3A4, although data suggests that glucuronidation may be the major metabolic route. Co-administration of some CYP3A4 inhibitors may increase exposure to capivasertib and hence potentially affect toxicity, while CYP3A4 inducers may decrease the exposure to capivasertib and may potentially affect efficacy.

The following lists (Table 20) are not intended to be exhaustive and a similar restriction will apply to other agents that are known to modulate CYP3A4 activity. Appropriate medical judgment is required. Please contact AstraZeneca with any queries you have on this issue.

**Table 20 CYP3A4-interacting medication that must be avoided or used with caution**

| Medication | Recommendation | Rationale |
|---|---|---|
| Clarithromycin<br>Cobicistat<br>Itraconazole<br>Ketoconazole<br>Nefazodone[a]<br>Posaconazole<br>Protease inhibitors (ritonavir, saquinavir, indinavir, tipranavir, telaprevir, elvitegravir, lopinavir, nelfinavir, boceprevir)<br>Telithromycin<br>Troleandomycin<br>Voriconazole | **Must be avoided** 2 weeks prior to capivasertib administration and for 2 days following discontinuation of capivasertib. | Potent CYP3A4 inhibitors, which may increase the capivasertib exposure. |

| Medication | Recommendation | Rationale |
|---|---|---|
| Carbamazepine[a]<br>Enzalutamide<br>Phenobarbital<br>Phenytoin<br>Rifabutin<br>Rifampicin<br>Mitotane<br>St John's wort | **Must be avoided** 2 weeks (3 weeks for St John's Wort and 4 weeks for enzalutamide) prior to capivasertib administration. | Potent CYP3A4 inducers, which may reduce the capivasertib exposure. |
| Aprepitant<br>Diltiazem<br>Erythromycin<br>Fluconazole<br>Verapamil | May be used with caution[b]. | Moderate CYP3A4 inhibitors which might increase the capivasertib exposure. |

[a] Carbamazepine and nefazodone must be avoided prior to capivasertib administration and 1 week following discontinuation of capivasertib. Carbamazepine is a potent CYP3A4 inducer and a CYP3A4 substrate; nefazodone is a potent CYP3A4 inducer.

[b] Drugs are permitted but caution should be exercised and patients monitored closely for possible drug interactions. Please refer to full prescribing information for all drugs prior to co-administration with capivasertib.

## D 2 Drugs that may be influenced by capivasertib

There are currently no data confirming that there are any pharmacokinetic (PK) interactions between capivasertib and CYP3A4. Likewise, there are no confirmed interactions with MATE1 or OCT2 substrates. The potential interactions detailed below (Table 21 and Table 22) are considered on the basis of preclinical data and physiologically based pharmacokinetic (PBPK) modelling. The following list is not intended to be exhaustive, and a similar restriction will apply to other agents that are known to be sensitive to inhibition of CYP3A4 metabolism and/or MATE1 or OCT2 transport and have a narrow therapeutic window. Appropriate medical judgment is required. Please contact AstraZeneca with any queries you have on this issue.

**Table 21 CYP3A4 substrates that must be avoided or used with caution**

| Medication | Usage | Rationale |
|---|---|---|
| Alfentanil<br>Atorvastatin<br>Carbamazepine[a]<br>Cerivastatin<br>Cyclosporin<br>Diergotamine<br>Ergotamine<br>Fentanyl<br>Lovastatin<br>Simvastatin<br>Sirolimus<br>Tacrolimus | **Must be avoided** 1 week prior to capivasertib administration and for 1 week following discontinuation of capivasertib. | CYP3A4 substrates, whose exposure may be increased by capivasertib. |
| Alprazolam<br>Domperidone<br>Erythromycin<br>Felodipine<br>Haloperidol<br>Isradipine<br>Midazolam<br>Methylprednisolone<br>Nifedipine<br>Pimozide<br>Quinidine<br>Sertraline<br>Tamoxifen<br>Trazodone<br>Triazolam | May be used with caution[b]. | CYP3A4 substrates, whose exposure may be increased by capivasertib. |

[a] Carbamazepine must be avoided prior to capivasertib administration and 1 week following discontinuation of capivasertib. Carbamazepine is a potent CYP3A4 inducer and a CYP3A4 substrate.

[b] Drugs are permitted but caution should be exercised and patients monitored closely for possible drug interactions. Please refer to full prescribing information for all drugs prior to co-administration with capivasertib.

**Table 22 MATE1 and/or OCT2 transporter substrates that should be used with caution**

| Medication | Recommendation | Rationale |
|---|---|---|
| Dofetilide | May be used with caution[a] | MATE1 and OCT2 substrate with a narrow therapeutic window whose exposure may be increased by capivasertib |

| Medication | Recommendation | Rationale |
|---|---|---|
| Metformin | See Section 8.4.5.3 | MATE1 and OCT2 substrate whose exposure may be increased by capivasertib |
| Procainamide | May be used with caution[a] | OCT2 substrate with a narrow therapeutic window whose exposure may be increased by capivasertib |

[a] Drugs are permitted but caution should be exercised and patients monitored closely for possible drug interactions. Please refer to full prescribing information for all drugs prior to co-administration with capivasertib.

## D 3 Guidance for statins

Atorvastatin, cerivastatin, lovastatin, and simvastatin must be avoided due to the potential for increased exposure through inhibition of CYP3A4 by capivasertib (Table 21).

Fluvastatin, pravastatin, and rosuvastatin are minimally influenced by CYP3A4 inhibitors, conveying a relatively low potential for clinically significant drug-drug interactions via this mechanism.

Capivasertib also has a potential to inhibit the organic-anion-transporting polypeptide 1B1 (OATP-1B1) transporter, which is implicated in the distribution and clearance of many of the statins. The predicted increase in the area under the plasma concentration-time curve (AUC) is 1.3-fold for pravastatin and 1.5-fold for rosuvastatin. It is, therefore, recommended that doses of pravastatin be capped to 40 mg once daily and rosuvastatin be capped to 10 mg once daily when combined with capivasertib, including 1 week prior to capivasertib administration and for 1 week following discontinuation of capivasertib.

In summary, rosuvastatin (up to 10 mg once daily), pravastatin (up to 40 mg once daily) and fluvastatin are appropriate agents to be used in patients included in capivasertib studies who require statin therapy.

## D 4 Additional resources

For additional inhibitors, inducers and substrates please refer to:

https://drug-interactions.medicine.iu.edu/Clinical-Table.aspx

https://www.fda.gov/Drugs/DevelopmentApprovalProcess/DevelopmentResources/DrugInteractionsLabeling/ucm093664.htm

# Appendix E Adverse event definitions and additional safety information

## E 1 Definition of adverse events

An adverse event (AE) is the development of any untoward medical occurrence in a patient or clinical study patient administered a medicinal product and which does not necessarily have a causal relationship with this treatment. An AE can therefore be any unfavourable and unintended sign (eg, an abnormal laboratory finding), symptom (for example nausea, chest pain), or disease temporally associated with the use of a medicinal product, whether or not considered related to the medicinal product.

The term AE is used to include both serious and non-serious AEs and can include a deterioration of a pre-existing medical occurrence. An AE may occur at any time, including run-in or washout periods, even if no Study treatment has been administered.

AEs of special interest events will be identified and actively monitored during the study.

## E 2 Definitions of serious adverse event

A serious adverse event is an AE occurring during any study phase (ie, run-in, treatment, washout, follow-up), that fulfils one or more of the following criteria:

- Results in death
- Is immediately life-threatening
- Requires in-patient hospitalisation or prolongation of existing hospitalisation
- Results in persistent or significant disability or incapacity
- Is a congenital abnormality or birth defect
- Is an important medical event that may jeopardise the patient or may require medical treatment to prevent one of the outcomes listed above

AEs for malignant tumours reported during a study should generally be assessed as Serious AEs. If no other seriousness criteria apply, the 'Important Medical Event' criterion should be used. In certain situations, however, medical judgement on an individual event basis should be applied to clarify that the malignant tumour event should be assessed and reported as a Non-Serious AE. For example, if the tumour is included as medical history and progression occurs during the study, but the progression does not change treatment and/or prognosis of the malignant tumour, the AE may not fulfil the attributes for being assessed as Serious, although reporting of the progression of the malignant tumour as an AE is valid and should occur. Also, some types of malignant tumours, which do not spread remotely after a routine treatment that does not require hospitalization, may be assessed as Non-Serious; examples include Stage 1 basal cell carcinoma and Stage 1A1 cervical cancer removed via cone biopsy.

The above instruction applies only when the malignant tumour event in question is a new malignant tumour (ie, it is *not* the tumour for which entry into the study is a criterion and that is being treated by the investigational medicinal product under study and is not the development of new or progression of existing metastasis to the tumour under study). Malignant tumours that – as part of normal, if rare, progression – undergo transformation (eg, Richter's transformation of B cell chronic lymphocytic leukaemia into diffuse large B cell lymphoma) should not be considered a new malignant tumour.

## E 3 Life-threatening

'Life-threatening' means that the patient was at immediate risk of death from the AE as it occurred or it is suspected that use or continued use of the product would result in the patient's death. 'Life-threatening' does not mean that had an AE occurred in a more severe form it might have caused death (eg, hepatitis that resolved without hepatic failure).

## E 4 Hospitalisation

Outpatient treatment in an emergency room is not in itself a serious AE (SAE), although the reasons for it may be (eg, bronchospasm, laryngeal oedema). Hospital admissions and/or surgical operations planned before or during a study are not considered AEs if the illness or disease existed before the patient was enrolled in the study, provided that it did not deteriorate in an unexpected way during the study.

## E 5 Important medical event or medical treatment

Medical and scientific judgement should be exercised in deciding whether a case is serious in situations where important medical events may not be immediately life-threatening or result in death, hospitalisation, disability or incapacity but may jeopardise the patient or may require medical treatment to prevent one or more outcomes listed in the definition of serious. These should usually be considered as serious.

Simply stopping the suspect drug does not mean that it is an important medical event; medical judgement must be used.

- Angioedema not severe enough to require intubation but requiring intravenous hydrocortisone treatment
- Hepatotoxicity caused by paracetamol (acetaminophen) overdose requiring treatment with N-acetylcysteine
- Intensive treatment in an emergency room or at home for allergic bronchospasm
- Blood dyscrasias (eg, neutropenia or anaemia requiring blood transfusion, etc.) or convulsions that do not result in hospitalisation
- Development of drug dependency or drug abuse

## E 6 Intensity rating scale

The grading scales found in the revised National Cancer Institute CTCAE latest version will be utilised for all events with an assigned CTCAE grading. For those events without assigned CTCAE grades, the recommendation in the CTCAE criteria that converts mild, moderate and severe events into CTCAE grades should be used. A copy of the CTCAE can be downloaded from the Cancer Therapy Evaluation Program website (http://ctep.cancer.gov). The applicable version of CTCAE should be described clearly.

It is important to distinguish between serious and severe AEs. Severity is a measure of intensity whereas seriousness is defined by the criteria in Appendix E 2. An AE of severe intensity need not necessarily be considered serious. For example, nausea that persists for several hours may be considered severe nausea, but not a SAE unless it meets the criteria shown in Appendix E 2. On the other hand, a stroke that results in only a limited degree of disability may be considered a mild stroke but would be a SAE when it satisfies the criteria shown in Appendix E 2.

## E 7 A guide to interpreting the causality question

When making an assessment of causality consider the following factors when deciding if there is a 'reasonable possibility' that an AE may have been caused by the drug.

- Time Course. Exposure to suspect drug. Has the patient actually received the suspect drug? Did the AE occur in a reasonable temporal relationship to the administration of the suspect drug?
- Consistency with known drug profile. Was the AE consistent with the previous knowledge of the suspect drug (pharmacology and toxicology) or drugs of the same pharmacological class? Or could the AE be anticipated from its pharmacological properties?
- De-challenge experience. Did the AE resolve or improve on stopping or reducing the dose of the suspect drug?
- No alternative cause. The AE cannot be reasonably explained by another aetiology such as the underlying disease, other drugs, other host or environmental factors.
- Re-challenge experience. Did the AE reoccur if the suspected drug was reintroduced after having been stopped? AstraZeneca would not normally recommend or support a re-challenge.
- Laboratory tests. A specific laboratory investigation (if performed) has confirmed the relationship.

In difficult cases, other factors could be considered such as:

- Is this a recognised feature of overdose of the drug?
- Is there a known mechanism?

Causality of 'related' is made if following a review of the relevant data, there is evidence for a 'reasonable possibility' of a causal relationship for the individual case. The expression 'reasonable possibility' of a causal relationship is meant to convey, in general, that there are facts (evidence) or arguments to suggest a causal relationship.

The causality assessment is performed based on the available data including enough information to make an informed judgment. With limited or insufficient information in the case, it is likely that the event(s) will be assessed as 'not related'.

Causal relationship in cases where the disease under study has deteriorated due to lack of effect should be classified as no reasonable possibility.

## E 8 Medication error

For the purposes of this clinical study a medication error is an unintended failure or mistake in the treatment process for an AstraZeneca study drug that either causes harm to the participant or has the potential to cause harm to the participant.

A medication error is not lack of efficacy of the drug, but rather a human or process related failure while the drug is in control of the study site staff or participant.

Medication error includes situations where an error:

- Occurred
- Was identified and intercepted before the participant received the drug
- Did not occur, but circumstances were recognised that could have led to an error

Examples of events to be reported in clinical studies as medication errors include:

- Drug name confusion
- Dispensing error eg, medication prepared incorrectly, even if it was not actually given to the participant
- Drug not administered as indicated, for example, wrong route or wrong site of administration
- Drug not taken as indicated eg, tablet dissolved in water when it should be taken as a solid tablet
- Drug not stored as instructed eg, kept in the fridge when it should be at room temperature
- Wrong participant received the medication (excluding IVRS/IWRS errors)
- Wrong drug administered to participant (excluding IVRS/IWRS errors)

Examples of events that **do not** require reporting as medication errors in clinical studies:

- Errors related to or resulting from IVRS/IWRS - including those which lead to one of the above listed events that would otherwise have been a medication error
- Participant accidentally missed drug dose(s) (eg, forgot to take medication)
- Accidental overdose (will be captured as an overdose)
- Participant failed to return unused medication or empty packaging
- Errors related to background and rescue medication, or standard of care medication in open-label studies, even if an AstraZeneca product

Medication errors are not regarded as AEs but AEs may occur as a consequence of the medication error.

# Appendix F Handling of human biological samples

## F 1 Chain of custody of biological samples

A full chain of custody is maintained for all samples throughout their lifecycle.

The investigator at each centre keeps full traceability of collected biological samples from the patients while in storage at the centre until shipment or disposal (where appropriate).

The sample receiver keeps full traceability of the samples while in storage and during use until used or disposed of or until further shipment and keeps documentation of receipt of arrival.

AstraZeneca will keep oversight of the entire life cycle through internal procedures, monitoring of study sites, auditing or process checks, and contractual requirements of external laboratory providers.

Samples retained for further use will be stored in the AstraZeneca-assigned biobanks and will be registered by the AstraZeneca Biobank Team during the entire life cycle.

If required, AstraZeneca will ensure that remaining biological samples are returned to the site according to local regulations or at the end of the retention period, whichever is the sooner.

## F 2 Withdrawal of informed consent for donated biological samples

If a patient withdraws consent to the use of donated biological samples, the samples will be disposed of/destroyed, and the action documented. If samples are already analysed, AstraZeneca is not obliged to destroy the results of this research.

As collection of the biological samples is an integral part of the study, then the patient is withdrawn from further study participation.

The investigator:

- Ensures patients' withdrawal of informed consent to the use of donated samples is notified immediately to AstraZeneca
- Ensures that biological samples from that patient, if stored at the study site, are immediately identified, disposed of /destroyed, and the action documented
- Ensures the organisation(s) holding the samples is/are informed about the withdrawn consent immediately and that samples are disposed of/destroyed, the action documented and the signed document returned to the study site
- Ensures that the patient and AstraZeneca are informed about the sample disposal

AstraZeneca ensures the organisations holding the samples is/are informed about the withdrawn consent immediately and that samples are disposed of/destroyed and the action documented and returned to the study site.

## F 3 International Airline Transportation Association (IATA) 6.2 guidance document

**LABELLING AND SHIPMENT OF BIOHAZARD SAMPLES**

International Airline Transportation Association (IATA) classifies biohazardous agents into 3 categories (http://www.iata.org/whatwedo/cargo/dangerous_goods/infectious_substances.htm). For transport purposes the classification of infectious substances according to risk groups was removed from the Dangerous Goods Regulations in the 46th edition (2005). Infectious substances are now classified either as Category A, Category B or Exempt. There is no direct relationship between Risk Groups and Categories A and B.

The investigator ensures that samples are labelled and shipped in accordance with the Laboratory Manual and the Biological Substance, Category B Regulations.

**Category A Infectious Substances** are infectious substances in a form that, when exposure to it occurs, is capable of causing permanent disability, life-threatening or fatal disease in otherwise healthy humans or animals. Category A pathogens are eg, Ebola, Lassa fever virus:

- Are to be packed and shipped in accordance with IATA Instruction 602.

**Category B Infectious Substances** are infectious substances that do not meet the criteria for inclusion in Category A. Category B pathogens are eg, Hepatitis A, B, C, D, and E viruses, Human immunodeficiency virus types 1 and 2. They are assigned the following UN number and proper shipping name:

- UN 3373 – Biological Substance, Category B
- Are to be packed in accordance with UN3373 and IATA 650

**Exempt** - all other materials with minimal risk of containing pathogens

- Clinical trial samples will fall into Category B or exempt under IATA regulations
- Clinical trial samples will routinely be packed and transported at ambient temperature in IATA 650 compliant packaging (http://www.iata.org/whatwedo/cargo/dangerous_goods/infectious_substances.htm)
- Biological samples transported in dry ice require additional dangerous goods specification for the dry-ice content
- IATA compliant courier and packaging materials should be used for packing and transportation and packing should be done by an IATA certified person, as applicable

- Samples routinely transported by road or rail are subject to local regulations which require that they are also packed and transported in a safe and appropriate way to contain any risk of infection or contamination by using approved couriers and packaging/containment materials at all times. The IATA 650 biological sample containment standards are encouraged wherever possible when road or rail transport is used

# Appendix G Genetics

## G 1 Use/analysis of DNA

Genetic variation may impact a patient's response to therapy, susceptibility to, and severity and progression of disease. Variable response to therapy may be due to genetic determinants that impact drug absorption, distribution, metabolism, and excretion; mechanism of action of the drug; disease aetiology; and/or molecular subtype of the disease being treated. Therefore, where local regulations and institutional review board (IRB)/independent ethics committee (IEC) allow, a blood sample will be collected for DNA analysis from consenting patients.

AstraZeneca intends to collect and store DNA for genetic research to explore how genetic variations may affect clinical parameters, risk and prognosis of diseases, and the response to medications. Genetic research may lead to better understanding of diseases, better diagnosis of diseases or other improvements in health care and to the discovery of new diagnostics, treatments or medications.

In addition, collection of DNA samples from populations with well described clinical characteristics may lead to improvements in the design and interpretation of clinical trials and, possibly, to genetically guided treatment strategies.

Genetic research may consist of the analysis of the structure of the patient´s DNA (ie, the entire genome).

The results of genetic analyses may be reported in the clinical study report (CSR) or in a separate study summary.

The sponsor will store the DNA samples in a secure storage space with adequate measures to protect confidentiality.

The samples will be retained while research on study treatment or study treatments of this class or indication continues but no longer than 15 years or other period as per local requirements.

## G 2 Genetic research plan and procedures

**Selection of genetic research population**

*Study selection record*

All patients will be asked to participate in this genetic research. Participation is voluntary and if a patient declines to participate there will be no penalty or loss of benefit. The patient will not be excluded from any aspect of the main study.

*Inclusion criteria*

For inclusion in this genetic research, patients must fulfil all of the inclusion criteria described in the main body of the Clinical Study Protocol (CSP) and provide informed consent for the genetic sampling and analyses.

*Exclusion criteria*

Exclusion from this genetic research may be for any of the exclusion criteria specified in the main study.

**Withdrawal of consent for genetic research**

Patients may withdraw from this genetic research at any time, independent of any decision concerning participation in other aspects of the main study. Voluntary withdrawal will not prejudice further treatment. Procedures for withdrawal are outlined in Section 7.3 of the main CSP.

**Collection of samples for genetic research**

The blood sample for genetic research will be obtained from the patients at screening. Although DNA is stable, early sample collection is preferred to avoid introducing bias through excluding patients who may withdraw due to an adverse event (AE), such patients would be important to include in any genetic analysis. If for any reason the sample is not drawn at screening, it may be taken at any visit until the last study visit. Only one sample should be collected per patient for genetics during the study. Samples will be collected, labelled, stored, and shipped as detailed in the Laboratory Manual.

**Coding and storage of DNA samples**

The processes adopted for the coding and storage of samples for genetic analysis are important to maintain patient confidentiality. Samples will be stored for a maximum of 15 years, from the date of last patient last visit, after which they will be destroyed. DNA is a finite resource that is used up during analyses. Samples will be stored and used until no further analyses are possible or the maximum storage time has been reached.

An additional second code will be assigned to the blood sample either before or at the time of DNA extraction replacing the information on the sample tube. Thereafter, the sample will be identifiable only by the second, unique number. This number is used to identify the sample and corresponding data at the AstraZeneca genetics laboratories, or at the designated organisation. No personal details identifying the individual will be available to any person (AstraZeneca employee or designated organisations working with the DNA).

The link between the patient enrolment/randomisation code and the second number will be maintained and stored in a secure environment, with restricted access at AstraZeneca or designated organisations. The link will be used to identify the relevant DNA samples for analysis, facilitate correlation of genotypic results with clinical data, allow regulatory audit, and permit tracing of samples for destruction in the case of withdrawal of consent.

**Ethical and regulatory requirements**

The principles for ethical and regulatory requirements for the study, including this genetics research component, are outlined in Appendix G.

**Informed consent**

The genetic component of this study is optional and the patient may participate in other components of the main study without participating in the genetic component. To participate in the genetic component of the study the patient must sign and date both the consent form for the main study and the genetic component of the study. Copies of both signed and dated consent forms must be given to the patient and the original filed at the study centre. The investigator(s) is responsible for ensuring that consent is given freely and that the patient understands that they may freely withdrawal from the genetic aspect of the study at any time.

**Patient data protection**

AstraZeneca will not provide individual genotype results to patients, any insurance company, any employer, their family members, general physician unless required to do so by law.

Extra precautions are taken to preserve confidentiality and prevent genetic data being linked to the identity of the patient. In exceptional circumstances, however, certain individuals might see both the genetic data and the personal identifiers of a patient. For example, in the case of a medical emergency, an AstraZeneca physician or an investigator might know a patient's identity and also have access to his or her genetic data. In addition, regulatory authorities may require access to the relevant files, though the patient's medical information and the genetic files would remain physically separate.

**Data management**

Any genotype data generated in this study will be stored at a secure system at AstraZeneca and/or designated organisations to analyse the samples.

AstraZeneca and its designated organisations may share summary results (such as genetic differences from groups of individuals with a disease) from this genetic research with other researchers, such as hospitals, academic organisations or health insurance companies. This can be done by placing the results in scientific databases, where they can be combined with

the results of similar studies to learn even more about health and disease. The researchers can only use this information for health-related research purposes. Researchers may see summary results but they will not be able to see individual patient data or any personal identifiers.

Some or all of the clinical datasets from the main study may be merged with the genetic data in a suitable secure environment separate from the clinical database.

**Statistical methods and determination of sample size**

The number of patients that will agree to participate in the genetic research is unknown. It is therefore not possible to establish whether sufficient data will be collected to allow a formal statistical evaluation or whether only descriptive statistics will be generated. A statistical analysis plan may be prepared where appropriate.

# Appendix H Actions required in cases of increases in liver biochemistry and evaluation of Hy's law

## H 1 Introduction

This Appendix describes the process to be followed in order to identify and appropriately report Potential Hy's Law (PHL) cases and Hy's Law (HL) cases. It is not intended to be a comprehensive guide to the management of elevated liver biochemistries. Specific guidance on managing liver abnormalities can be found in Section 8.3.8.

During the course of the study the investigator will remain vigilant for increases in liver biochemistry. The investigator is responsible for determining whether a patient meets PHL criteria at any point during the study.

All sources of laboratory data are appropriate for the determination of PHL and HL events; this includes samples taken at scheduled study visits and other visits including central and all local laboratory evaluations even if collected outside of the study visits; for example, PHL criteria could be met by an elevated ALT from a central laboratory **and/or** elevated TBL from a local laboratory.

The investigator will also review adverse event (AE) data (for example, for AEs that may indicate elevations in liver biochemistry) for possible PHL events.

The investigator participates, together with AstraZeneca clinical project representatives, in review and assessment of cases meeting PHL criteria to agree whether HL criteria are met. HL criteria are met if there is no alternative explanation for the elevations in liver biochemistry other than drug induced liver injury (DILI) caused by the investigational medicinal product (IMP).

The investigator is responsible for recording data pertaining to PHL/HL cases and for reporting serious AEs (SAEs) and AEs according to the outcome of the review and assessment in line with standard safety reporting processes.

## H 2 Definitions

**Potential Hy's Law**

Aspartate aminotransferase (AST) or alanine aminotransferase (ALT) ≥3 × upper limit of normal (ULN) **together with** total bilirubin (TBL) ≥2 × ULN at any point during the study following the start of study medication irrespective of an increase in alkaline phosphatase (ALP).

**Hy's Law**

AST or ALT ≥3 × ULN **together with** TBL ≥2 × ULN, where no other reason, other than the IMP, can be found to explain the combination of increases, eg, elevated ALP indicating cholestasis, viral hepatitis, another drug.

For PHL and HL, the elevation in transaminases must precede or be coincident with (ie, on the same day) the elevation in TBL, but there is no specified time frame within which the elevations in transaminases and TBL must occur.

## H 3 Identification of potential Hy's Law cases

In order to identify cases of PHL it is important to perform a comprehensive review of laboratory data for any patient who meets any of the following identification criteria in isolation or in combination:

- ALT ≥3 × ULN
- AST ≥3 × ULN
- TBL ≥2 × ULN

The investigator will without delay review each new laboratory report and if the identification criteria are met will:

- Notify the AstraZeneca representative
- Determine whether the patient meets PHL criteria (see Appendix H 2 for definition) by reviewing laboratory reports from all previous visits
- Promptly enter the laboratory data into the laboratory eCRF

## H 4 Follow-up

### H 4.1 Potential Hy's Law criteria not met

If the patient does not meet PHL criteria the investigator will:

- Inform the AstraZeneca representative that the patient has not met PHL criteria.
- Perform follow-up on subsequent laboratory results according to the guidance provided in the clinical study protocol (CSP).

### H 4.2 Potential Hy's Law criteria met

If the patient does meet PHL criteria the investigator will:

- Notify the AstraZeneca representative who will then inform the central Study Team

- Within 1 day of PHL criteria being met, the investigator will report the case as an SAE of PHL; serious criteria 'important medical event' and causality assessment 'yes/related' according to CSP process for SAE reporting
- For patients that met PHL criteria prior to starting IMP, the investigator is not required to submit a PHL SAE unless there is a significant change in the patient's condition***

The study physician contacts the investigator to provide guidance, discuss and agree an approach for the study patients' follow-up (including any further laboratory testing) and the continuous review of data.

- Subsequent to this contact the investigator will:
  - Monitor the patient until liver biochemistry parameters and appropriate clinical symptoms and signs return to normal or baseline levels, or as long as medically indicated, and complete follow-up SAE form as required
  - Investigate the aetiology of the event and perform diagnostic investigations as discussed with the study physician
  - Complete the 3 liver eCRF modules as information becomes available

***A 'significant' change in the patient's condition refers to a clinically relevant change in any of the individual liver biochemistry parameters (ALT, AST or total bilirubin) in isolation or in combination, or a clinically relevant change in associated symptoms. The determination of whether there has been a significant change will be at the discretion of the Investigator, this may be in consultation with the study physician if there is any uncertainty.

## H 5 Review and assessment of potential Hy's Law cases

The instructions in this section should be followed for all cases where PHL criteria are met.

As soon as possible after the biochemistry abnormality was initially detected, the study physician contacts the investigator in order to review available data and agree on whether there is an alternative explanation for meeting PHL criteria other than DILI caused by the IMP, to ensure timely analysis and reporting to health authorities within 15 calendar days from date PHL criteria were met. The AstraZeneca Global Clinical Lead or equivalent and Global Safety Physician will also be involved in this review together with other patient matter experts as appropriate.

According to the outcome of the review and assessment, the investigator will follow the instructions below.

**Where there is an agreed alternative explanation** for the ALT or AST and TBL elevations, a determination of whether the alternative explanation is an AE will be made and subsequently whether the AE meets the criteria for a SAE:

- If the alternative explanation is **not** an AE, record the alternative explanation on the appropriate eCRF
- If the alternative explanation is an AE/SAE: update the previously submitted PHL SAE and AE CRFs accordingly with the new information (reassessing event term; causality and seriousness criteria) following the AstraZeneca processes

If it is agreed that there is **no** explanation that would explain the ALT or AST and TBL elevations other than the IMP:

- Send updated SAE (report term 'Hy's Law') according to AstraZeneca standard processes.
  - The 'Medically Important' serious criterion should be used if no other serious criteria apply
  - As there is no alternative explanation for the HL case, a causality assessment of 'related' should be assigned

If there is an unavoidable delay of over 15 calendar days in obtaining the information necessary to assess whether or not the case meets the criteria for HL, then it is assumed that there is no alternative explanation until such time as an informed decision can be made:

- Provide any further update to the previously submitted SAE of PHL (report term now 'Hy's Law case') ensuring causality assessment is related to IMP and seriousness criteria is medically important, according to CSP process for SAE reporting
- Continue follow-up and review according to agreed plan. Once the necessary supplementary information is obtained, repeat the review and assessment to determine whether HL criteria are still met. Update the previously submitted PHL SAE report following CSP process for SAE reporting, according to the outcome of the review amending the reported term if an alternative explanation for the liver biochemistry elevations is determined

# Appendix I Patient-reported outcomes – questionnaires

## I 1 EORTC QLQ-C30

### EORTC QLQ-C30 (version 3)

We are interested in some things about you and your health. Please answer all of the questions yourself by circling the number that best applies to you. There are no "right" or "wrong" answers. The information that you provide will remain strictly confidential.

Please fill in your initials: ____
Your birthdate (Day, Month, Year): ____
Today's date (Day, Month, Year): 31 ____

| | Not at All | A Little | Quite a Bit | Very Much |
|---|---|---|---|---|
| 1. Do you have any trouble doing strenuous activities, like carrying a heavy shopping bag or a suitcase? | 1 | 2 | 3 | 4 |
| 2. Do you have any trouble taking a long walk? | 1 | 2 | 3 | 4 |
| 3. Do you have any trouble taking a short walk outside of the house? | 1 | 2 | 3 | 4 |
| 4. Do you need to stay in bed or a chair during the day? | 1 | 2 | 3 | 4 |
| 5. Do you need help with eating, dressing, washing yourself or using the toilet? | 1 | 2 | 3 | 4 |

| During the past week: | Not at All | A Little | Quite a Bit | Very Much |
|---|---|---|---|---|
| 6. Were you limited in doing either your work or other daily activities? | 1 | 2 | 3 | 4 |
| 7. Were you limited in pursuing your hobbies or other leisure time activities? | 1 | 2 | 3 | 4 |
| 8. Were you short of breath? | 1 | 2 | 3 | 4 |
| 9. Have you had pain? | 1 | 2 | 3 | 4 |
| 10. Did you need to rest? | 1 | 2 | 3 | 4 |
| 11. Have you had trouble sleeping? | 1 | 2 | 3 | 4 |
| 12. Have you felt weak? | 1 | 2 | 3 | 4 |
| 13. Have you lacked appetite? | 1 | 2 | 3 | 4 |
| 14. Have you felt nauseated? | 1 | 2 | 3 | 4 |
| 15. Have you vomited? | 1 | 2 | 3 | 4 |
| 16. Have you been constipated? | 1 | 2 | 3 | 4 |

Please go on to the next page

Specimen

| During the past week: | Not at All | A Little | Quite a Bit | Very Much |
|---|---|---|---|---|
| 17. Have you had diarrhea? | 1 | 2 | 3 | 4 |
| 18. Were you tired? | 1 | 2 | 3 | 4 |
| 19. Did pain interfere with your daily activities? | 1 | 2 | 3 | 4 |
| 20. Have you had difficulty in concentrating on things, like reading a newspaper or watching television? | 1 | 2 | 3 | 4 |
| 21. Did you feel tense? | 1 | 2 | 3 | 4 |
| 22. Did you worry? | 1 | 2 | 3 | 4 |
| 23. Did you feel irritable? | 1 | 2 | 3 | 4 |
| 24. Did you feel depressed? | 1 | 2 | 3 | 4 |
| 25. Have you had difficulty remembering things? | 1 | 2 | 3 | 4 |
| 26. Has your physical condition or medical treatment interfered with your family life? | 1 | 2 | 3 | 4 |
| 27. Has your physical condition or medical treatment interfered with your social activities? | 1 | 2 | 3 | 4 |
| 28. Has your physical condition or medical treatment caused you financial difficulties? | 1 | 2 | 3 | 4 |

**For the following questions please circle the number between 1 and 7 that best applies to you**

29. How would you rate your overall health during the past week?

| 1 | 2 | 3 | 4 | 5 | 6 | 7 |
|---|---|---|---|---|---|---|
| Very poor | | | | | | Excellent |

30. How would you rate your overall quality of life during the past week?

| 1 | 2 | 3 | 4 | 5 | 6 | 7 |
|---|---|---|---|---|---|---|
| Very poor | | | | | | Excellent |

© Copyright 1995 EORTC Quality of Life Group. All rights reserved. Version 3.0

Specimen

# I 2 EORTC QLQ-BR23

ENGLISH

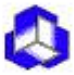

## EORTC QLQ - BR23

Patients sometimes report that they have the following symptoms or problems. Please indicate the extent to which you have experienced these symptoms or problems during the past week.

| During the past week: | Not at All | A Little | Quite a Bit | Very Much |
|---|---|---|---|---|
| 31. Did you have a dry mouth? | 1 | 2 | 3 | 4 |
| 32. Did food and drink taste different than usual? | 1 | 2 | 3 | 4 |
| 33. Were your eyes painful, irritated or watery? | 1 | 2 | 3 | 4 |
| 34. Have you lost any hair? | 1 | 2 | 3 | 4 |
| 35. Answer this question only if you had any hair loss: Were you upset by the loss of your hair? | 1 | 2 | 3 | 4 |
| 36. Did you feel ill or unwell? | 1 | 2 | 3 | 4 |
| 37. Did you have hot flushes? | 1 | 2 | 3 | 4 |
| 38. Did you have headaches? | 1 | 2 | 3 | 4 |
| 39. Have you felt physically less attractive as a result of your disease or treatment? | 1 | 2 | 3 | 4 |
| 40. Have you been feeling less feminine as a result of your disease or treatment? | 1 | 2 | 3 | 4 |
| 41. Did you find it difficult to look at yourself naked? | 1 | 2 | 3 | 4 |
| 42. Have you been dissatisfied with your body? | 1 | 2 | 3 | 4 |
| 43. Were you worried about your health in the future? | 1 | 2 | 3 | 4 |

| During the past four weeks: | Not at All | A Little | Quite a Bit | Very Much |
|---|---|---|---|---|
| 44. To what extent were you interested in sex? | 1 | 2 | 3 | 4 |
| 45. To what extent were you sexually active? (with or without intercourse) | 1 | 2 | 3 | 4 |
| 46. Answer this question only if you have been sexually active: To what extent was sex enjoyable for you? | 1 | 2 | 3 | 4 |

Please go on to the next page

SPECIMEN

ENGLISH

| During the past week: | Not at All | A Little | Quite a Bit | Very Much |
|---|---|---|---|---|
| 47. Did you have any pain in your arm or shoulder? | 1 | 2 | 3 | 4 |
| 48. Did you have a swollen arm or hand? | 1 | 2 | 3 | 4 |
| 49. Was it difficult to raise your arm or to move it sideways? | 1 | 2 | 3 | 4 |
| 50. Have you had any pain in the area of your affected breast? | 1 | 2 | 3 | 4 |
| 51. Was the area of your affected breast swollen? | 1 | 2 | 3 | 4 |
| 52. Was the area of your affected breast oversensitive? | 1 | 2 | 3 | 4 |
| 53. Have you had skin problems on or in the area of your affected breast (e.g., itchy, dry, flaky)? | 1 | 2 | 3 | 4 |

SPECIMEN

© Copyright 1994 EORTC Quality of Life Group. All rights reserved. Version 1.0

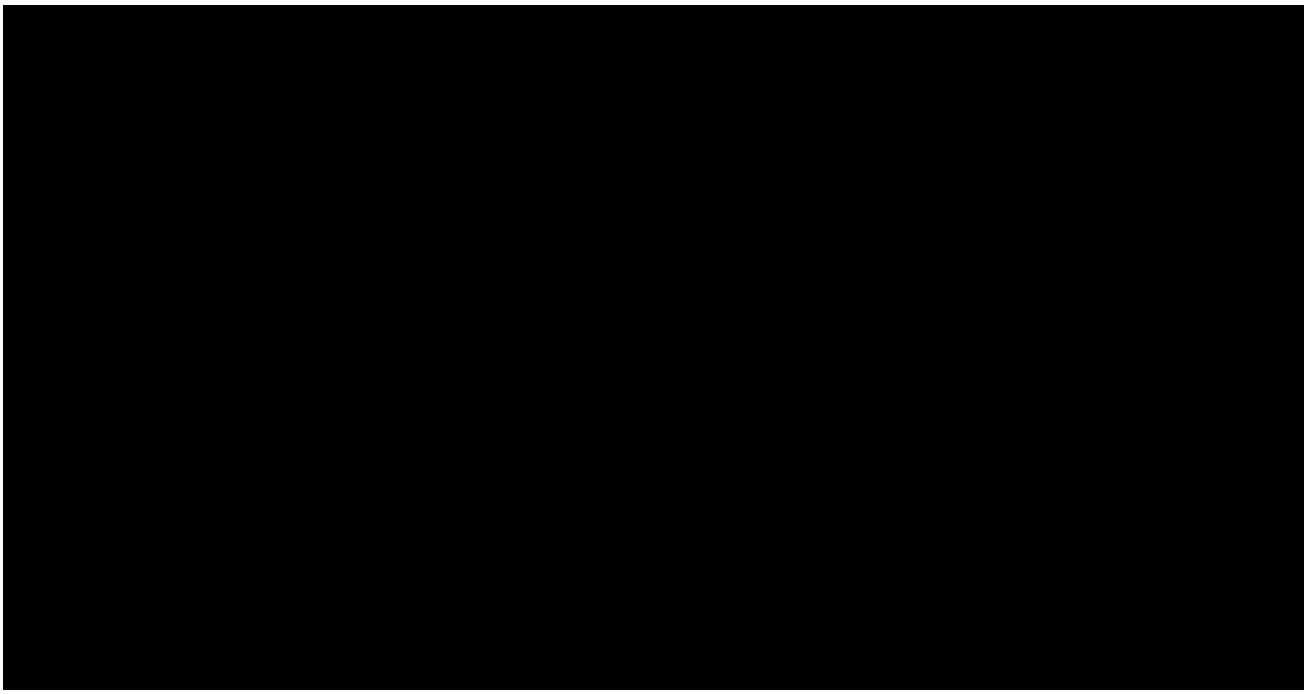

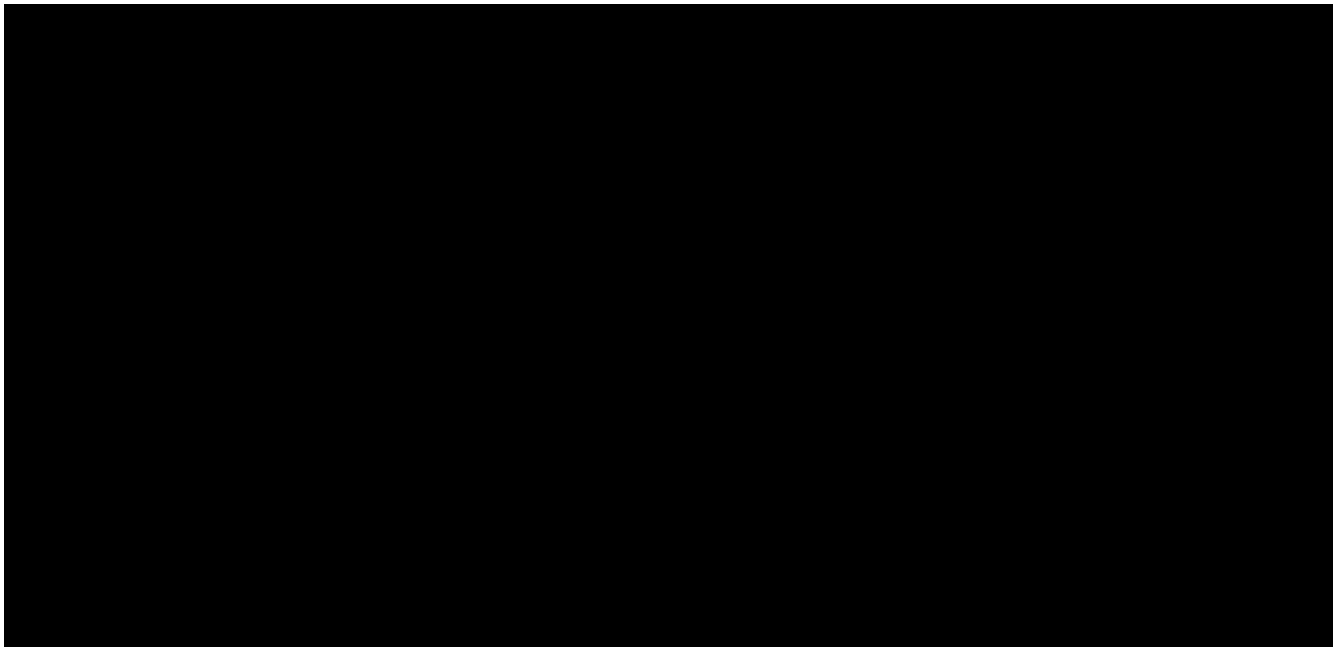

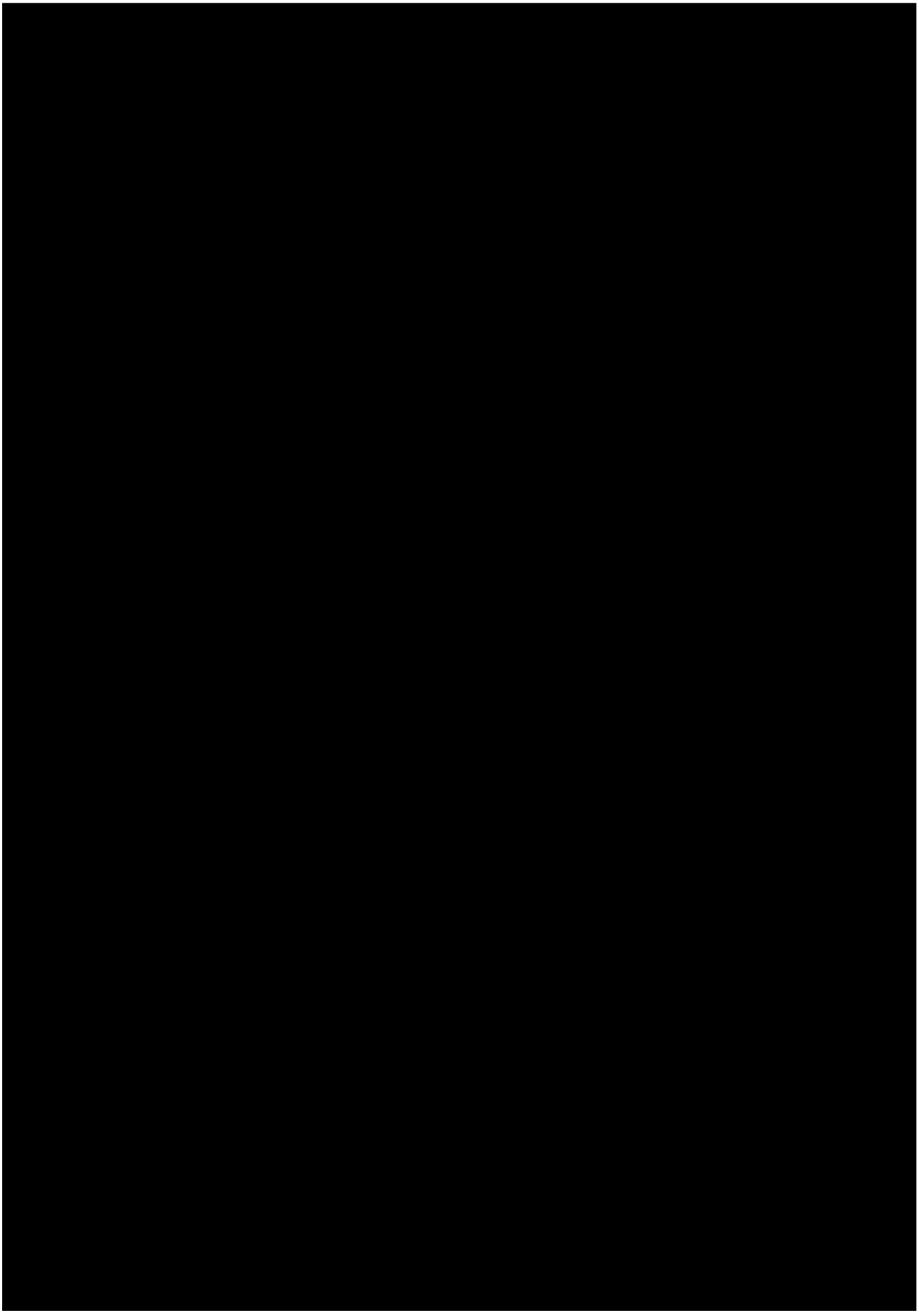

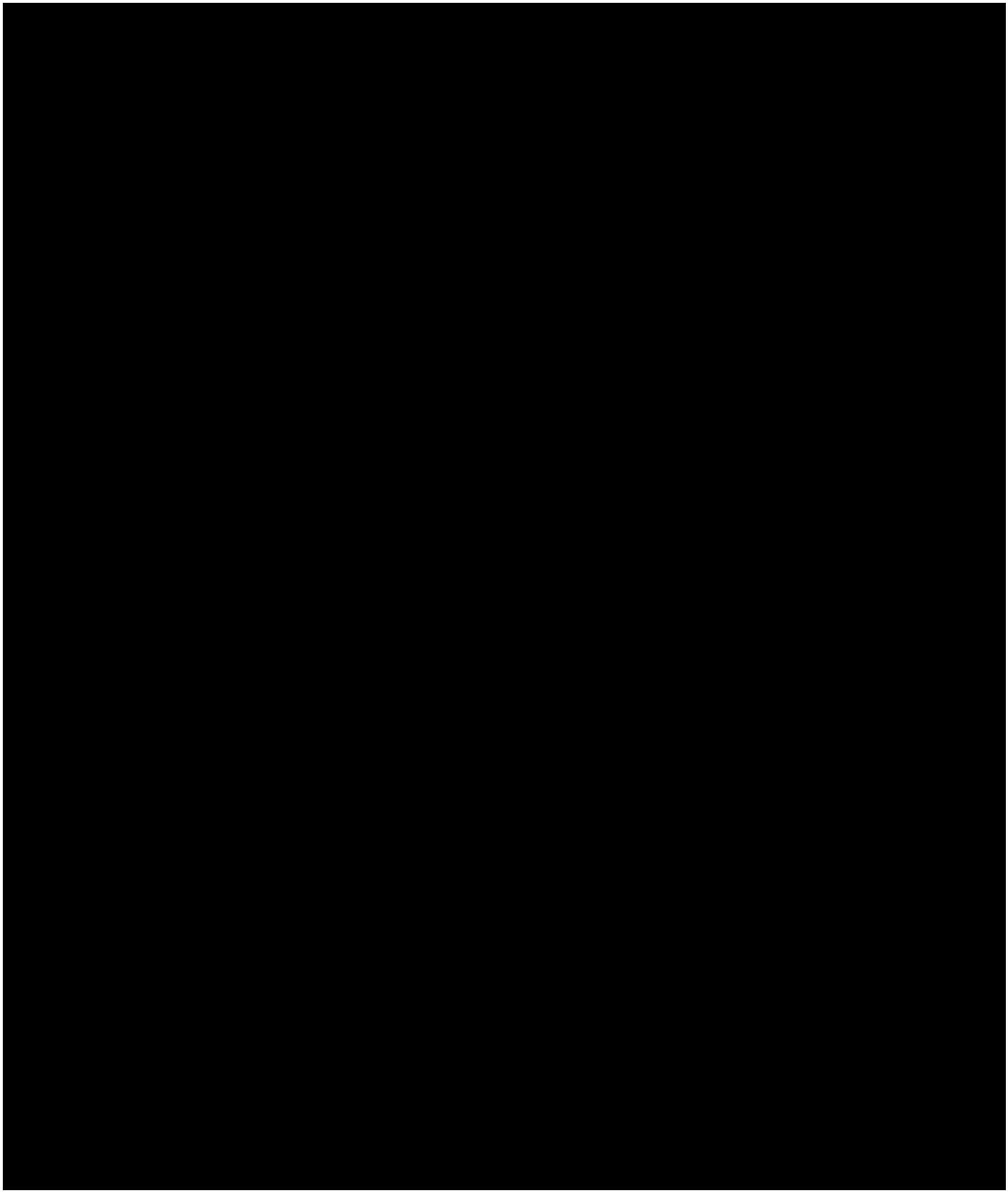

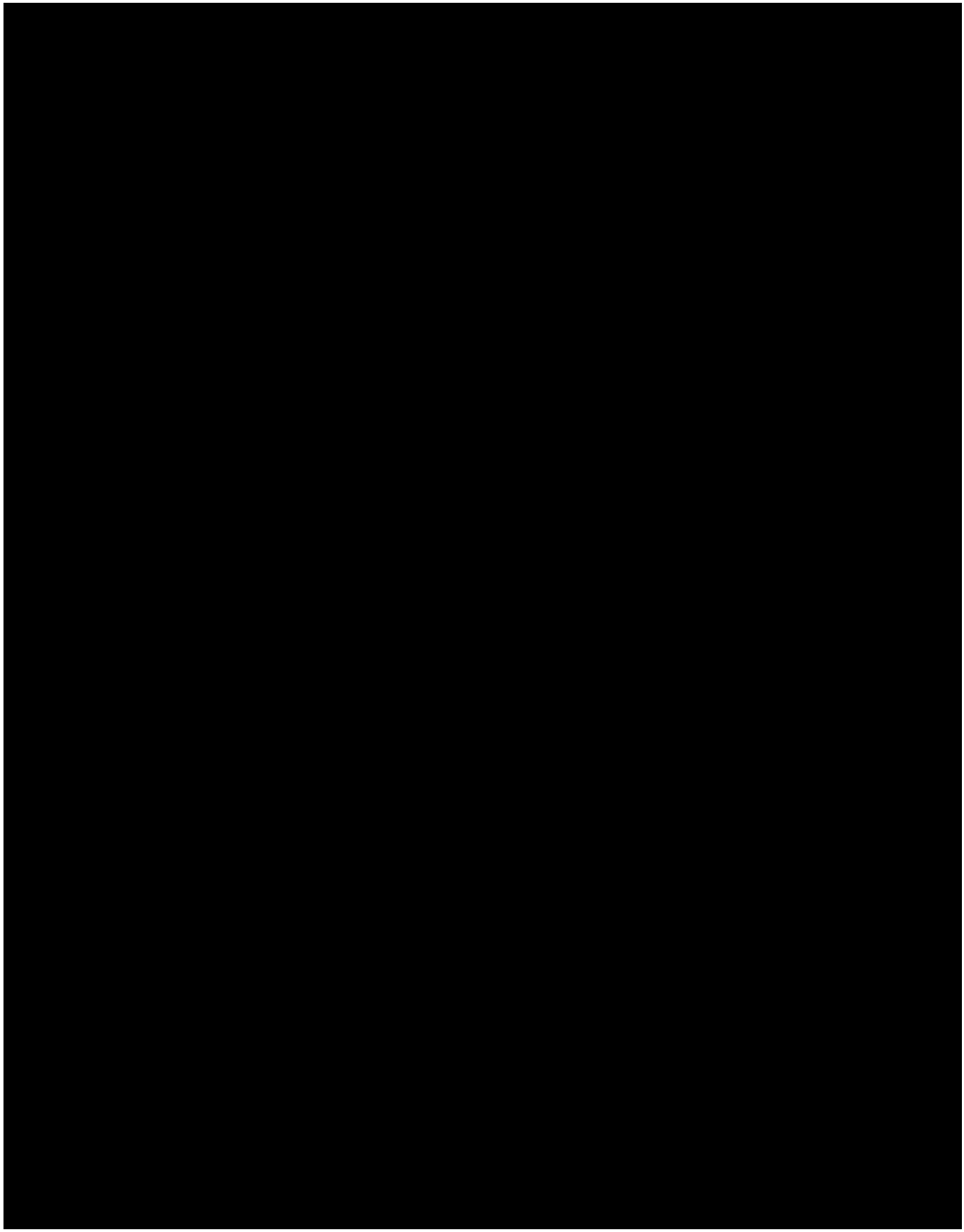

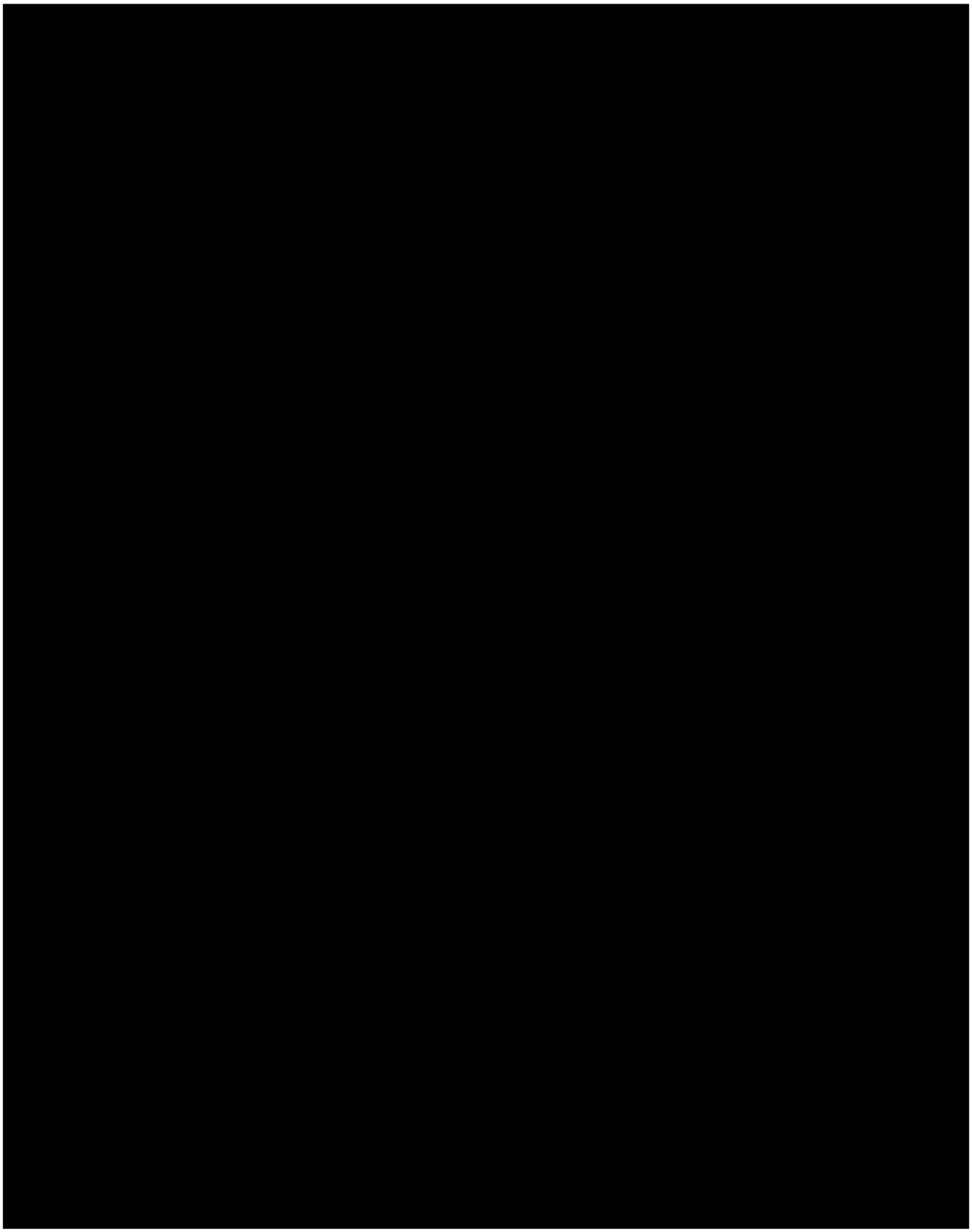

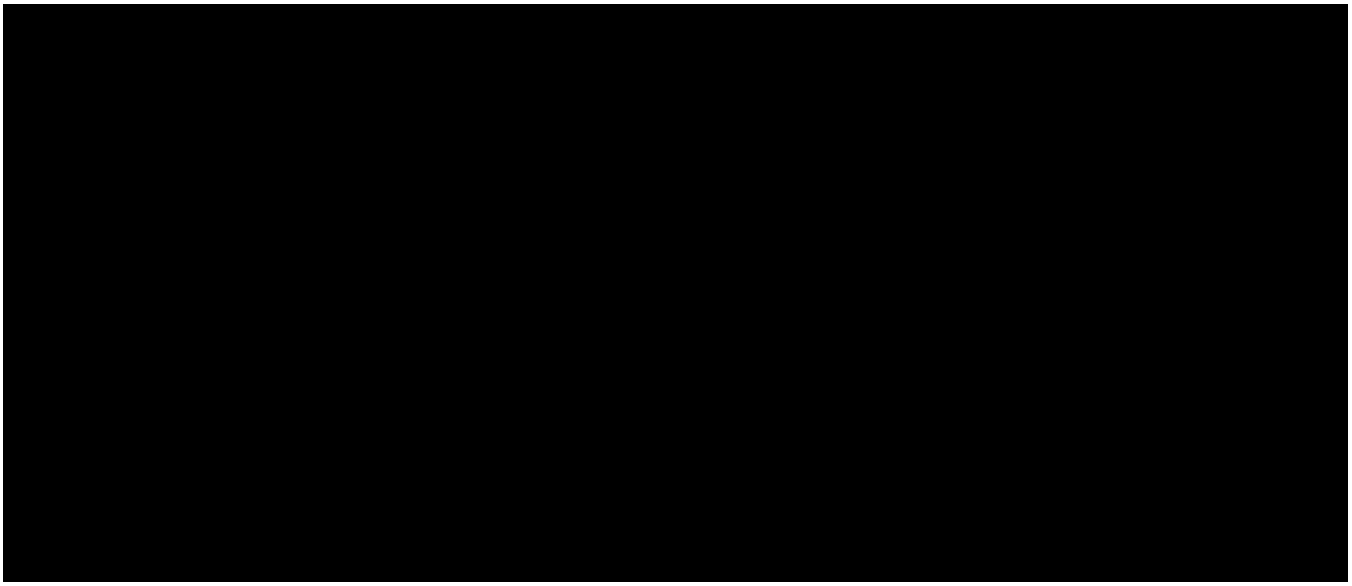

# Appendix J Abbreviations

The following abbreviations and special terms are used in this study Clinical Study Protocol.

| Abbreviation or special term | Explanation |
|---|---|
| ABC | Advanced breast cancer |
| AE | Adverse event |
| AESI | Adverse Event of Special Interest |
| AI | Aromatase inhibitor |
| ALP | Alkaline phosphatase |
| ALT | Alanine aminotransferase |
| AST | Aspartate aminotransferase |
| AUC | Area under the plasma concentration-time curve |
| $AUC_{0-12h}$ | Area under the plasma concentration-time curve from zero to 12 hours |
| BD | Twice-daily |
| BICR | Blinded Independent Central Review |
| *BRCA1/2* | Breast cancer gene 1/2 |
| CBR | Clinical benefit rate |
| CI | Confidence interval |
| $C_{max}$ | Maximum observed plasma (peak) concentration |
| CMH | Cochran-Mantel Haenszel |
| COVID-19 | Novel coronavirus disease 2019 |
| CSP | Clinical study protocol |
| CSR | Clinical study report |
| CT | Computer tomography |
| CTCAE | Common Terminology Criteria for Adverse Events |
| ctDNA | Circulating tumour DNA |
| CYP | Cytochrome P450 |
| DCO | Data cut-off |
| DNA | Deoxyribonucleic acid |
| DoR | Duration of response |
| ECG | Electrocardiogram |
| ECHO | Echocardiography |
| ECOG | Eastern Cooperative Oncology Group |
| eCRF | Electronic case report form |
| EDC | Electronic data capture |

| Abbreviation or special term | Explanation |
|---|---|
| EORTC | European Organisation for Research and Treatment of Cancer |
| EORTC QLQ-BR23 | EORTC Quality of Life Questionnaire-breast cancer specific module |
| EORTC QLQ-C30 | EORTC Quality of Life Questionnaire-Core 30 items |
| ePRO | Electronic patient-reported outcomes |
| EQ-5D-5L | European Quality of Life 5-Domain 5-Level Scale |
| ER | Estrogen receptor |
| FAS | Full Analysis Set |
| FFPE | Formalin-fixed paraffin-embedded |
| FSH | Follicle stimulating hormone |
| GCP | Good Clinical Practice |
| HbA1c | Glycosylated haemoglobin |
| HER2 | Human epidermal growth factor receptor 2 |
| HIV | Human immunodeficiency virus |
| HOSPAD | Hospital Admission |
| HR | Hazard ratio |
| HR+ | Hormone receptor positive |
| HRQoL | Health-related quality of life |
| IA | Interim analysis |
| ICF | Informed consent form |
| ICH | International Conference on Harmonisation |
| IDMC | Independent Data Monitoring Committee |
| IEC | Independent Ethics Committee |
| IHC | Immunohistochemistry |
| IMP | Investigational medicinal product |
| IRB | Institutional Review Board |
| ITT | Intent-to-treat |
| IV | Intravenous |
| IVD | In-vitro diagnostic |
| IVRS | Interactive Voice Response System |
| IWRS | Interactive Web Response System |
| KM | Kaplan-Meier |
| LHRH | Luteinising-hormone releasing hormone |
| LTED | Long-term estrogen deprivation |
| LMWH | Low molecular weight heparin |
| LPFV | Last patient first visit |

| Abbreviation or special term | Explanation |
|---|---|
| LVEF | Left ventricular ejection fraction |
| MRI | Magnetic resonance imaging |
| MTD | Maximum tolerated dose |
| mTOR | Mammalian target of rapamycin |
| MTP | Multiple testing procedure |
| MUGA | Multiple-gated acquisition |
| NCCN | National Comprehensive Cancer Network |
| NCI | National Cancer Institute |
| NGS | Next-Generation Sequencing |
| NL | New lesion |
| NMPA | National Medical Product Administration |
| NTL | Non-target lesion |
| ORR | Objective response rate |
| OS | Overall survival |
| PFS | Progression-free survival |
| PFS2 | Time from randomisation to second progression or death |
| PGIC | Patient Global Impression–Change |
| PGIS | Patient Global Impression–Severity |
| PGI-TT | Patient Global Impression–Treatment Tolerability |
| PI3K | Phosphatidylinositol-3-kinase |
| PK | Pharmacokinetics |
| PR | Partial response |
| PRO | Patient-reported outcome |
| PRO-CTCAE | Patient-reported outcomes version of the Common Terminology Criteria for Adverse Events |
| PTEN | Phosphatase and tensin homolog |
| QoL | Quality of life |
| QT | ECG interval measured from the onset of the QRS complex to the end of the T wave |
| QTc | Corrected QT interval |
| QTcF | QT corrected by Fridericia's formula |
| RECIST | Response Evaluation Criteria in Solid Tumours. This study will use RECIST version 1.1. |
| RNA | Ribonucleic acid |
| SAE | Serious adverse event |

| Abbreviation or special term | Explanation |
|---|---|
| SAP | Statistical analysis plan |
| SARS-CoV-2 | Severe acute respiratory syndrom cornoavirus 2 |
| SBGM | Self-blood glucose monitoring |
| SERD | Selective estrogen receptor degrader |
| SoA | Schedule of Activities |
| TFSC | Time to first subsequent chemotherapy |
| TL | Target lesion |
| $t_{max}$ | Time to reach peak or maximum observed concentration following drug administration |
| TNBC | Triple-negative breast cancer |
| TSC | Trial Steering Committee |
| ULN | Upper limit of normal |
| vs | Versus |
| WHO | World Health Organisation |

# Appendix K Changes Related to Mitigation of Study Disruptions Due to COVID-19 Outbreak - Temporary Measures

Note: Changes below should be temporarily implemented only during study disruptions due to COVID-19 outbreak (eg, during quarantines and resulting site closures, regional travel restrictions and considerations if site personnel or study patients become infected with COVID-19) during which patients may not wish to or may be unable to visit the study site for study visits. These changes should only be implemented if allowable by local/regional guidelines and following agreement from the Sponsor.

Study sites may continue to recruit new patients into ongoing studies provided the following activities to preserve study integrity can be met:

- Upon discussion with the site monitor, the study site has confirmed the ability to enroll and manage new subjects effectively and in compliance with the protocol
- Data will continue to be entered into the eCRF and queries resolved in a timely manner.

## K 1 Reconsent of Study Patients During Study Interruptions

During study interruptions, it may not be possible for the patients to complete study visits and assessments on site and alternative means for carrying out the visits and assessments may be necessary, eg, remote visits. Reconsent should be obtained for the alternative means of carrying out visits and assessments and should be obtained prior to performing the procedures described in Section 1.1. Local and regional regulations and/or guidelines regarding reconsent of study patients should be checked and followed. Reconsent may be verbal if allowed by local and regional guidelines (note, in the case of verbal reconsent the ICF should be signed at the patient's next contact with the study site). Visiting the study sites for the sole purpose of obtaining reconsent should be avoided.

## K 2 Rescreening of Study Patients to Reconfirm Study Eligibility

Additional rescreening for screen failure due to study disruption can be performed in previously screened patients. The investigator should confirm this with the designated AstraZeneca study physician. In addition, during study disruption there may be a delay between confirming eligibility of a patient and either enrolment into the study or commencing of dosing with study treatment. If this delay is outside the screening window specified in Section 1.1, the patient will need to be rescreened to reconfirm eligibility before commencing study procedures. This will provide another opportunity to re-screen a patient in addition to that detailed in Section 5.4. The procedures detailed in Section 1.1 must be undertaken to confirm eligibility.

## K 3 Home or Remote Visit to Replace On-site Visit (where applicable)

A qualified health care professional from the study site or third party vendor service may visit the patient's home/or other remote location as per local standard operating procedures, as applicable. Supplies will be provided for a safe and efficient visit. The qualified health care professional will be expected to collect information per the CSP.

## K 4 Telemedicine Visit to Replace On-site Visit (where applicable)

In this appendix, the term telemedicine visit refers to remote contact with the patients using telecommunications technology including phone calls, virtual or video visits, and mobile health devices.

During the COVID-19 outbreak, visits may be replaced by a telemedicine visit if allowed by local/regional guidelines. Having a telemedicine contact with the patients will allow adverse events, concomitant medication and other relevant data to be collected according to study requirements to be reported and documented.

## K 5 At-home or Remote Location Study Treatment Administration Instructions

If a site visit is not possible, at-home or remote location administration of study treatment may be performed, where available on a case by case basis by a qualified health care professional, provided this is acceptable within local regulation/guidance. Please contact your monitor. The option of at-home or remote location study treatment administration ensures patients safety in cases of a pandemic where patients may be at increased risk by traveling to the site/clinic. This will also minimise interruption of study treatment administration during other study disruptions, eg, site closures due to COVID-19.

### K 5.1 At-home or Remote Location Study Treatment Administration by a Qualified Health Care Professional or Third Party Vendor Service

A qualified health care professional from the study site or third party vendor service may administer the study treatment at the patient's home or a remote location according to the CSP. All necessary supplies and instructions for administration and documentation of study treatment administration will be provided. Additional information related to the visit can be obtained via a telemedicine or home visit.

## K 6 At-home or Remote Delivery of Capivasertib/Placebo

Alternative secure delivery methods for oral drug supply (capivasertib/placebo) may be permitted if the patient is unable to attend the site, but only provided the critical safety assessments have been performed and the delivery methods are in line with local regulatory requirements.

## K 7 Data Capture During Telemedicine or Remote Visits

Data collected during telemedicine or remote visits will be captured in the source documents by the qualified health care professional from the study site or third party vendor service, or from the patient themselves.

# Appendix L Guidance during the COVID-19 Outbreak

## L 1 COVID-19 Risk Assessment

The safety of participants is of primary importance. Any potential risks of participating in the study, particularly with the added challenges due to COVID-19 outbreak, should be weighed against the anticipated benefit (see also principle 2.2 of ICH GCP). Investigators are advised to use clinical judgment in determining infection prevention precautions for study participants.

The emergence of SARS-CoV-2 presents a potential safety risk for cancer patients. Participants enrolling in this study may require more frequent visits to the site for study treatment administration and for study assessments compared to participants receiving standard of care. Therefore, several risk mitigation factors have been implemented related to study conduct during the COVID-19 outbreak, for patient management in an event of COVID-19, and actions to be taken on study treatment (see Appendix L 4). With these measures in place, it is considered that the anticipated potential benefits for the participants enrolled in this study outweigh the potential risks. All implemented measures prioritise trial participant safety and data validity; in case these two conflict with each other, trial participant safety should always prevail (see also EMA 2020).

Notably, participants with active COVID-19 infection confirmed by local laboratory testing will not be eligible for study enrolment (see Section 5.2, Exclusion Criterion 12).

## L 2 Potential Risks during COVID-19

Every effort should be made to follow the CSP. This appendix provides a dose modification and management plan for participants with confirmed or suspected COVID-19 who are being treated with study intervention capivasertib/placebo.

The risk-benefit assessment should be carefully considered for each participant enrolling in the study based on the known safety risks related to COVID-19, individual needs, and local guidelines and restrictions. Investigators must continue to use their best clinical judgment in determining the most optimal care for participants and utmost diligence in determining their eligibility for study participation, continued study treatment, and overall assessment of benefit/risk of study treatment or participation.

The sponsor must be promptly notified of a site's inability to perform study activities due to COVID-19 outbreak in order to minimise any potential risks.

## L 3 New Participant Enrolment

Study sites may continue to recruit new participants into the study provided the following activities to preserve study integrity can be met:

- Upon discussion with the site monitor, the study site has confirmed the ability to enrol and manage new participants effectively and in compliance with the protocol.
- Data will continue to be entered into the eCRF and queries resolved in a timely manner.

Per CSP Exclusion Criterion 12 (see Section 5.2), participants with evidence of severe or uncontrolled systemic diseases, including but not limited to, ongoing or active infection are not eligible for the study participation and hence such participants (including those who have confirmed COVID-19) should not be included for study participation.

Per Exclusion Criterion 26 (see Section 5.2), patients who have circumstances that could limit compliance with study requirements should also be excluded. Please consider this criterion carefully considering evolving circumstances, travel restrictions and health care delivery in your local area that may impact the continued treatment in the study.

The Study Physician should be contacted if any additional guidance or clarification is needed via the local monitor or directly.

## L 4 Study Treatment Administration

If an AE or SAE is associated with COVID-19, the investigator should determine whether the participants' treatment with investigational product should continue, be interrupted, or be discontinued in accordance with the CSP.

Adverse events, SAEs, cycle delays and/or treatment suspensions associated with COVID-19 along with logistical issues should be reported according to the eCRF Completion Guidelines.

For dosing discontinuations, where applicable, the dosing discontinuation guidelines should be followed, and the End of Treatment Form(s) completed.

## L 5 Vaccination against COVID-19

Protocol restrictions applying to live attenuated vaccines are relevant for live attenuated COVID-19 vaccines as well. Investigators should apply their discretion assessing the risk benefit of other types of COVID-19 vaccines for participants in clinical trials. Ideally, administration of the vaccine should be done on a different day other than the day of study drug administration to differentiate any potential AEs seen from the vaccine and study drug. The administration of the vaccine and any potential AEs associated with the vaccine are to be documented on the concomitant medication and AE eCRFs, respectively.

## L 6 Capivasertib/Placebo: Product Specific Guidance In Relation To The Ongoing And Emerging Novel Coronavirus (COVID-19) Pandemic

For Ongoing Patients:

- Patients must continue to have safety blood tests as per protocol schedule. Alternative methods for safety assessments include using local laboratories and follow up by phone contact, virtual visits can be used (see Appendix K for mitigation procedures)
- If it becomes unfeasible to perform the required safety blood tests for a patient, then study treatment should be interrupted until this can resumed and the reason clearly documented, with reference to COVID-19.
- If a patient tests positive for the COVID-19 virus, interrupting capivasertib/placebo treatment for 14 days or until symptoms resolve should be considered. Factors that should be taken into consideration might include:
    - Severity of COVID-19 symptoms
    - Status of safety blood results, particularly haemoglobin, neutrophils, and lymphocytes
    - Benefit risk for the individual patients including curative vs. palliative intent of treatment and response to capivasertib/placebo
- If patients present with new or worsening respiratory symptoms such as dyspnoea, cough and fever, or an abnormal chest radiologic finding is observed, capivasertib/placebo treatment should be interrupted and prompt investigation initiated to determine whether symptoms are due to COVID-19 or potentially drug-induced pneumonitis.
- The use of capivasertib/placebo with the concomitant use of some antibiotics and antivirals (eg, telithromycin, clarithromycin, protease inhibitors boosted with ritonavir or cobicistat, indinavir, saquinavir, nelfinavir, boceprevir and telaprevir) is not recommended. Please review Appendix D for details.

## REFERENCES

**Curigliano et al 2020**
Curigliano G, Banerjee S, Cervantes A, Garassino M, Garrido P, Girard N. Managing cancer patients during the COVID-19 pandemic: an ESMO multidisciplinary expert consensus. Ann Oncol 2020;31(10):1320-35.

**EMA 2020**
EMA, Clinical Trials Facilitation and Coordination Group, European Commission. Guidance on the Management of Clinical Trials during the COVID-19 (Coronavirus) pandemic, Version 2, 27 March 2020. Available from: URL: https://ec.europa.eu/health/sites/health/files/files/eudralex/vol-10/guidanceclinicaltrials_covid19_en.pdf. Accessed: 17 December 2020.

# SIGNATURE PAGE

*This is a representation of an electronic record that was signed electronically and this page is the manifestation of the electronic signature*

| **Document Name:** d3615c00001-csp-v4 | | |
|---|---|---|
| **Document Title:** | D3615C00001 Clinical Study Protocol version 4 | |
| **Document ID:** | Doc ID-004589372 | |
| **Version Label:** | 3.0 CURRENT LATEST APPROVED | |
| **Server Date** **(dd-MMM-yyyy HH:mm 'UTC'Z)** | **Signed by** | **Meaning of Signature** |
| 11-Feb-2022 09:43 UTC | [redacted] | [redacted] |
| 11-Feb-2022 12:08 UTC | [redacted] | [redacted] |
| 11-Feb-2022 18:40 UTC | [redacted] | [redacted] |

Notes: (1) Document details as stored in ANGEL, an AstraZeneca document management system.
