# Supplementary material for: Capivasertib plus fulvestrant in patients with HR-positive/HER2-negative advanced breast cancer: phase 3 CAPItello-291 study extended Chinese cohort
Source: Nat Commun. 2025 May 9;16:4324. doi: 10.1038/s41467-025-59210-6 (PMC12064754; doi:10.1038/s41467-025-59210-6)
Supplement: Supplementary file 2 — Reporting Summary [file 41467_2025_59210_MOESM2_ESM.pdf]

nature portfolio

Corresponding author(s): Xichun Hu

Last updated by author(s): Mar 19, 2025

Reporting Summary

Nature Portfolio wishes to improve the reproducibility of the work that we publish. This form provides structure for consistency and transparency in reporting. For further information on Nature Portfolio policies, see our Editorial Policies and the Editorial Policy Checklist.

Statistics

For all statistical analyses, confirm that the following items are present in the figure legend, table legend, main text, or Methods section.

n/a	Confirmed	
☐	☒	The exact sample size (*n*) for each experimental group/condition, given as a discrete number and unit of measurement
☒	☐	A statement on whether measurements were taken from distinct samples or whether the same sample was measured repeatedly
☐	☒	The statistical test(s) used AND whether they are one- or two-sided<br>*Only common tests should be described solely by name; describe more complex techniques in the Methods section.*
☐	☒	A description of all covariates tested
☐	☒	A description of any assumptions or corrections, such as tests of normality and adjustment for multiple comparisons
☐	☒	A full description of the statistical parameters including central tendency (e.g. means) or other basic estimates (e.g. regression coefficient) AND variation (e.g. standard deviation) or associated estimates of uncertainty (e.g. confidence intervals)
☒	☐	For null hypothesis testing, the test statistic (e.g. *F*, *t*, *r*) with confidence intervals, effect sizes, degrees of freedom and *P* value noted<br>*Give P values as exact values whenever suitable.*
☒	☐	For Bayesian analysis, information on the choice of priors and Markov chain Monte Carlo settings
☒	☐	For hierarchical and complex designs, identification of the appropriate level for tests and full reporting of outcomes
☒	☐	Estimates of effect sizes (e.g. Cohen's *d*, Pearson's *r*), indicating how they were calculated

Our web collection on statistics for biologists contains articles on many of the points above.

Software and code

Policy information about availability of computer code

Data collection	Medidata Rave (Medidata, NY, USA) versions: Medidata Classic Rave® 2021.2.0, 2022.2.0, 2023.2.0, SAS Enterprise (SAS Institute, NC, USA) version 7.13 HF8 (7.100.3.5532), Origin Study Modeller (Formedix, UK) version 3.7.2 2017117-1048, Medidata Coder Module (Medidata, NY, USA) version 2018.2.0, RAVE Lab Admin Module (Medidate, NY, USA) version 2017.2.3, Xcellerate Data Review (Covance, NJ, USA) version 11Jun2018, and Xcellerate Risk and Issue Management (Covance, NJ, USA)
Data analysis	SAS (SAS Institute, NC, USA) version 9.4

For manuscripts utilizing custom algorithms or software that are central to the research but not yet described in published literature, software must be made available to editors and reviewers. We strongly encourage code deposition in a community repository (e.g. GitHub). See the Nature Portfolio guidelines for submitting code & software for further information.

Data

Policy information about availability of data

All manuscripts must include a data availability statement. This statement should provide the following information, where applicable:

- Accession codes, unique identifiers, or web links for publicly available datasets
- A description of any restrictions on data availability
- For clinical datasets or third party data, please ensure that the statement adheres to our policy

Data underlying the findings described in this manuscript may be obtained in accordance with AstraZeneca's data sharing policy described at https://astrazenecagrouptrials.pharmacm.com/ST/Submission/Disclosure. Anonymized datasets (to General Data Protection Regulation standards, with link to patient code

destroyed) would be available on request. Data could be requested through Vivli at https://vivli.org/members/enquiries-about-studies-not-listed-on-the-vivli-platform/. AstraZeneca's Vivli member page is also available, outlining further details: https://vivli.org/ourmember/astrazeneca/. Some patients/countries may need to be excluded based on the informed consent form or country-level legislation (e.g. Chinese patients would be excluded based on Human Genetic Resources Regulations). Patients who have withdrawn consent for data use will also be removed from the shared dataset. Any data submitted to this journal will have elements removed to reduce risk of patient reidentification while meeting the journal's needs for data reproducibility and transparency. Only clinical trial data may be shared. Patient-level image or genetic data are not available for access in our repository, in the interest of protecting patient privacy. Available documents include the clinical trial protocol, statistical analysis plan, informed consent form and clinical study report. Data can be available until the expiry of the Retention and Disposal Schedule of the data, based upon trial milestones at AstraZeneca. The data will be available upon approval of the request and signature of the Data Usage Agreement until, typically, one year starting on the date access was granted. Use of the data is restricted to the named users approved for the request and is made available to the requestor for one year from the date access was granted. Please refer to the Data Usage Agreement (https://vivli.org/resources/vivli-data-use-agreement/; non-negotiable contract for data accessors) for more information.

Research involving human participants, their data, or biological material

Policy information about studies with human participants or human data. See also policy information about sex, gender (identity/presentation), and sexual orientation and race, ethnicity and racism.

Reporting on sex and gender	Biological sex was reported at baseline and is presented in Table 1 of the manuscript. In the capivasertib–fulvestrant group, 100% of the patients were female and in placebo–fulvestrant group, 98.4% of the patients were female (only one male patient). This is expected as approximately 99% of all breast cancer cases occur in women (https://www.who.int/news-room/fact-sheets/detail/breast-cancer). No analyses have been conducted according to sex.
Reporting on race, ethnicity, or other socially relevant groupings	The study recruited a Chinese cohort of patients from sites in mainland China and Taiwan. All patients with known race were Asian. Data for race were not available for four patients (two in each treatment group). No analyses have been conducted according to race or ethnicity.
Population characteristics	Patient characteristics at baseline are outlined in Table 1 of the manuscript.
Recruitment	Patients were recruited by participating investigators in 25 sites in mainland China and three National Medical Products Administration-certified sites in Taiwan between 13 October 2020 and 3 January 2023. Patients were selected based on the uniform inclusion/exclusion criteria outlined in the study protocol. Tumor assessments were evaluated by Response Evaluation Criteria in Solid Tumours (RECIST) v1.1 to minimize bias.
Ethics oversight	The study was designed and overseen by an academic steering group that included representatives from AstraZeneca, the sponsor. An institutional review board and independent ethics committee reviewed the study protocol, amendments, and other relevant documents.<br>The study was also approved by the following local independent ethics committees (ECs) /institutional review boards (IRBs): China: Hubei Cancer Hospital, The Third Hospital of Nanchang, The First People's Hospital of Foshan, Affiliated Hospital of Hebei University, IRB EC of Linyi Cancer Hospital, The Second People's Hospital of Neijiang, IRB Shantou Central Hospital Ethics Committee; Taiwan: Research EC (REC) National Taiwan University, REC China Medical University Hospital, IRB Chang Gung Medical Foundation, IRB Taipei Veterans General Hospital, IRB National Cheng Kung University Hospital, IRB Koo Foundation Sun Yat-Sen Cancer Center, IRB E-DA Hospital, IRB Chi Mei Medical Center.<br>The study was approved by the Human Genetics Resources Administration of China and China's Ministry of Science and Technology, and was conducted in accordance with the applicable International Council for Harmonisation of Technical Requirements for Pharmaceuticals for Human Use and Good Clinical Practice guidelines and the principles of the Declaration of Helsinki. All patients gave informed consent prior to enrollment.

Note that full information on the approval of the study protocol must also be provided in the manuscript.

Field-specific reporting

Please select the one below that is the best fit for your research. If you are not sure, read the appropriate sections before making your selection.

☒ Life sciences ☐ Behavioural & social sciences ☐ Ecological, evolutionary & environmental sciences

For a reference copy of the document with all sections, see nature.com/documents/nr-reporting-summary-flat.pdf

Life sciences study design

All studies must disclose on these points even when the disclosure is negative.

Sample size	All statistical analyses in the Chinese cohort were exploratory, and only performed if sufficient numbers of events or patients were available (eg, ≥ 20 overall survival or progression-free survival events). No formal sample size/power calculation was performed. Recruitment of patients was planned to continue until approximately 134 patients had been randomized, irrespective of whether or not the overall study enrolment had been reached, to ensure adequate participation to satisfy China Regulatory Authority requirements. Analysis of the dual primary endpoints was planned at approximately 77% maturity in both the overall Chinese cohort population (when 103 events of progression or death had occurred) and in the population of patients with PIK3CA/AKT1/PTEN-altered tumors (when approximately 41 events of progression or death had occurred, assuming a prevalence of 40%).
Data exclusions	Participants were excluded from randomization if they failed to meet the inclusion/exclusion criteria outlined in the study protocol. Efficacy analyses included all the patients who underwent randomization. Patients who received at least one dose of capivasertib, fulvestrant or placebo were included in the safety analyses.

Replication	No replication attempts were taken at this study. The detailed study protocol provided with submission would allow a similar study to be conducted.
Randomization	Fulvestrant was allocated to be dispensed to each patient using the interactive web response system (IWRS) system (if centrally sourced) or via the local pharmacy (if locally sourced) at each patient dispensing visit. Routines for this were described in the interactive voice response system (IVRS)/IWRS user manual that will be provided to each centre. Eligible patients were randomized in a 1:1 ratio (capivasertib–fulvestrant: placebo–fulvestrant). The planned treatment given to individual patients was determined by a randomization scheme that was loaded into the IWRS database. The randomization scheme was produced by a process called AZRand (AZ Global Randomisation process) that incorporates a standard procedure for generating random numbers. A blocked randomization was generated, and all centres used the same list in order to minimize any imbalance in the number of patients assigned to each treatment group. Eligibility was established before treatment randomization. It was recommended that patients commence study treatment as soon as possible after randomization and ideally on the same day of randomization.
Blinding	This was a double-blind study, i.e. neither the patients nor the investigators were aware of the treatment received by the patients. Capivasertib and placebo film-coated tablets were identical in appearance and presented in the same packaging to ensure blinding of capivasertib. Blinded analysis was carried out and unblinding occurred for data interpretation following analysis.

Reporting for specific materials, systems and methods

We require information from authors about some types of materials, experimental systems and methods used in many studies. Here, indicate whether each material, system or method listed is relevant to your study. If you are not sure if a list item applies to your research, read the appropriate section before selecting a response.

Materials & experimental systems

n/a	Involved in the study
☒	☐ Antibodies
☒	☐ Eukaryotic cell lines
☒	☐ Palaeontology and archaeology
☒	☐ Animals and other organisms
☐	☒ Clinical data
☒	☐ Dual use research of concern
☒	☐ Plants

Methods

n/a	Involved in the study
☒	☐ ChIP-seq
☒	☐ Flow cytometry
☒	☐ MRI-based neuroimaging

Clinical data

Policy information about clinical studies

All manuscripts should comply with the ICMJEguidelines for publication of clinical research and a completedCONSORT checklist must be included with all submissions.

Clinical trial registration	NCT04305496
Study protocol	The study protocol has already been published as supplementary material of Turner NC et al. N Engl J Med 2023;388:2058-2070; doi:10.1056/NEJMoa2214131 and has also been provided with the present submission.
Data collection	Patients were recruited from 25 sites in mainland China and three National Medical Products Administration-certified sites in Taiwan between 13 October 2020 and 3 January 2023 as part of the global CAPItello-291 study or the extended study with the same protocol in mainland China.
Outcomes	The dual primary endpoint was investigator-assessed progression-free survival by Response Evaluation Criteria in Solid Tumours (RECIST) v1.1 of capivasertib–fulvestrant versus placebo–fulvestrant in the overall population and in patients with PIK3CA/AKT1/PTEN-altered tumors. Tumor assessments were performed per RECIST v1.1 by computed tomography and/or magnetic resonance imaging scans at screening (within 4 weeks before randomization), every 8 weeks for the first 18 months, and then every 12 weeks until disease progression.<br><br>Secondary efficacy endpoints included overall survival (defined as the length of time from randomization until the date of death due to any cause), objective response rate (defined as the percentage of patients with at least one complete or partial response per RECIST v1.1, as assessed by the investigator at the local site) and clinical benefit rate (defined as the percentage of patients who have a complete response, partial response or stable disease per RECIST v1.1 [without subsequent cancer therapy] maintained ≥24 weeks after randomization).<br><br>Secondary outcomes also included safety and tolerability. Adverse events were recorded continuously until 30 days after treatment discontinuation and were graded using the National Cancer Institute (NCI) Common Terminology Criteria for Adverse Events (CTCAE) v5.0. European Organisation for Research and Treatment of Cancer Quality of Life Core (EORTC QLQ-C30) global health status/quality of life scores were evaluated, including change from baseline and time to deterioration (defined as the time from the date of randomization until the date of the first clinically meaningful deterioration [a sustained decrease of ≥ 10 points in the score from baseline]).

Plants

Seed stocks	n/a
Novel plant genotypes	n/a
Authentication	n/a
